# Histology and transcriptomic analyses of barnacles with different base materials and habitats shed lights on the duplication and chemical diversification of barnacle cement proteins

**DOI:** 10.1186/s12864-021-08049-4

**Published:** 2021-11-01

**Authors:** Hsiu-Chin Lin, Yue Him Wong, Chia-Hsuan Sung, Benny Kwok Kan Chan

**Affiliations:** 1 Department of Marine Biotechnology and Resources, National Sun Yat-sen University, 80424 Kaohsiung, Taiwan; 2 Institute for Advanced Study, Shenzhen University, Shenzhen, China; 3 Planning and Information Division, Fisheries Research Institute, Keelung, Taiwan; 4 Biodiversity Research Center, Academia Sinica, 115 Taipei, Taiwan

**Keywords:** Barnacle, Cement protein, Cement gland, Transcriptome

## Abstract

**Background:**

Barnacles are sessile crustaceans that attach to underwater surfaces using barnacle cement proteins. Barnacles have a calcareous or chitinous membranous base, and their substratum varies from biotic (e.g. corals/sponges) to abiotic surfaces. In this study, we tested the hypothesis that the cement protein (CP) composition and chemical properties of different species vary according to the attachment substrate and/or the basal structure. We examined the histological structure of cement glands and explored the variations in cement protein homologs of 12 barnacle species with different attachment habitats and base materials.

**Results:**

Cement gland cells in the rocky shore barnacles *Tetraclita japonica formosana* and *Amphibalanus amphitrite* are eosinophilic, while others are basophilic. Transcriptome analyses recovered CP homologs from all species except the scleractinian coral barnacle *Galkinia* sp. A phylogenomic analysis based on sequences of CP homologs did not reflect a clear phylogenetic pattern in attachment substrates. In some species, certain CPs have a remarkable number of paralogous sequences, suggesting that major duplication events occurred in CP genes. The examined CPs across taxa show consistent bias toward particular sets of amino acid. However, the predicted isoelectric point (pI) and hydropathy are highly divergent. In some species, conserved regions are highly repetitive.

**Conclusions:**

Instead of developing specific cement proteins for different attachment substrata, barnacles attached to different substrata rely on a highly duplicated cementation genetic toolkit to generate paralogous CP sequences with diverse chemical and biochemical properties. This general CP cocktail might be the key genetic feature enabling barnacles to adapt to a wide variety of substrata.

**Supplementary Information:**

The online version contains supplementary material available at 10.1186/s12864-021-08049-4.

## Background

How marine sessile invertebrates permanently attach to surfaces is a fascinating issue that has attracted attention from both evolutionary and applied scientists for more than a century. Cementation of sessile organisms typically involves three major components: (1) the surface of the sessile organism attaching to the substratum, (2) bioadhesive substances and (3) the substratum to which organisms are attaching. Bioadhesive substances are the key components responsible for gluing an organism’s attachment surface to a substratum. Bioadhesion is therefore highly dependent on the strength of the interactions between the material that makes up of the attachment surfaces and the bioadhesives, and between the bioadhesive substance and the substratum materials. Barnacles, mussels, bryozoans and ascidians are common sessile organisms capable of attaching to a wide range of substrata. However, it remains unclear whether or to what extent the evolution of bioadhesive substances in these sessile organisms are influenced by the proximal bottom structure and/or the substratum materials.

**Fig. 1:**
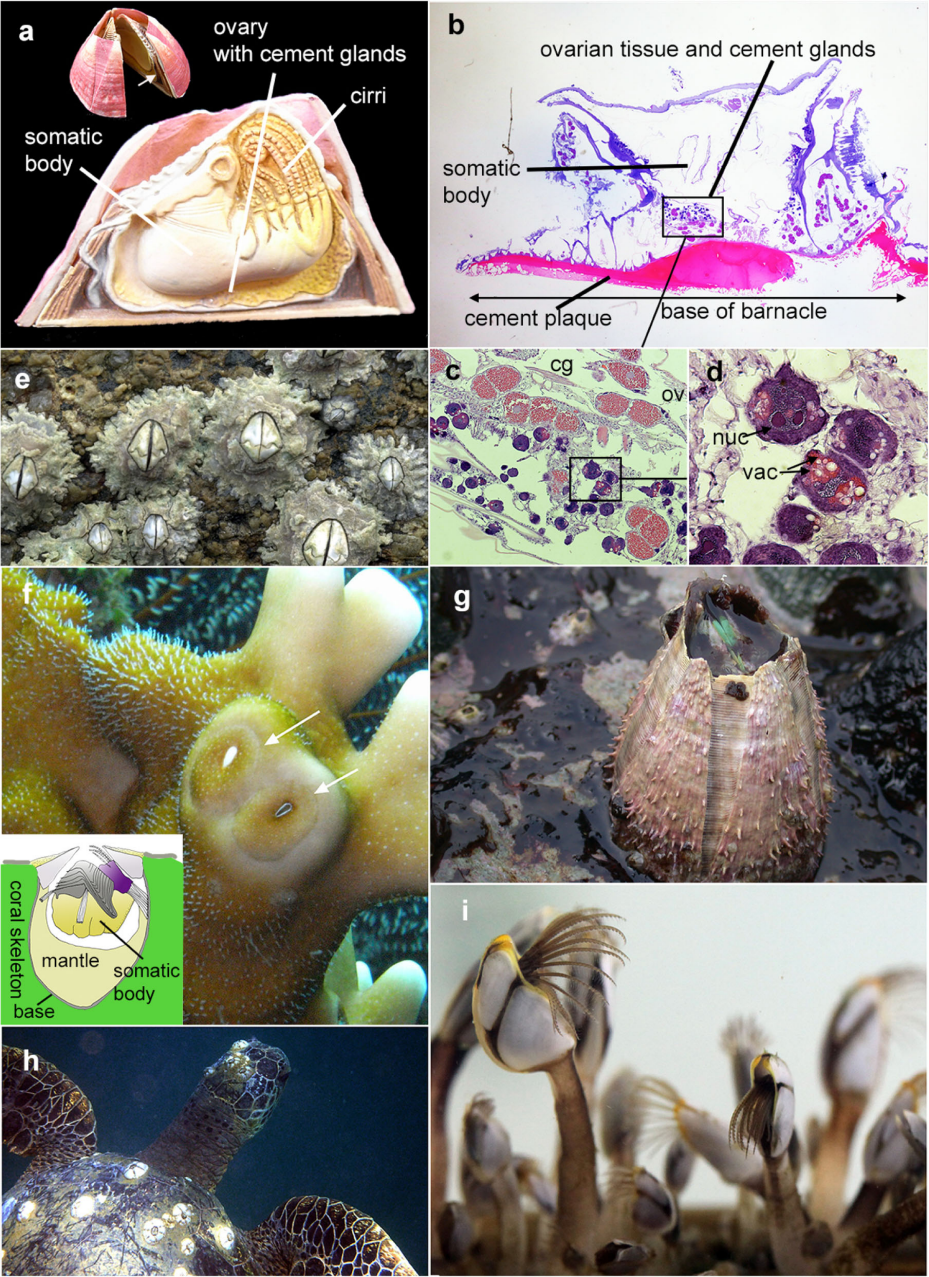
Barnacle cement gland and variations in barnacles’ habitat and attachment substratum.** a.** Plastic toy of the barnacle *Megabalanus rosa*, showing the cross section of the barnacle. Cement glands are located among the ovarian tissues at the base of the barnacle. **b**. Histological cross section of the acorn barnacle *Chelonibia testudinaria* showing the plaque of cement stained red at the base and the location of cement glands among the ovarian glands (boxes correspond to magnified views in **c** followed by **d**). A plaque of cement stained red at the base. **e**. *Chthamalus malayensis* is a membranous based barnacle that lives on intertidal rocks. **f. ***Wanella milleporae* is a membranous based barnacle that lives exclusively on fire corals. **g. ***Megabalanus volano* is a barnacle that lives on intertidal rocks with calcareous bases. **h**. *Chelonibia testudinaria* are epibiotic on sea turtles (photo by Ceri Lewis). **i. ***Lepas* species often attach on floating woods or other marine animals

Barnacles are sessile crustaceans that are infamous for being irreversibly affixed to an impressive range of biotic and abiotic surfaces, ranging from whales, sea turtles, jellyfish, crustaceans, mussels, corals, sponges, rocky shores and a variety of man-made objects, including ships [1–3] (Fig. 1). They are also considered key marine biofoulers because they significantly impact both the population dynamics of their biological hosts [4, 5] and the costs of antifouling measures. The underlying structural and molecular mechanisms of barnacle attachment is poorly studied, particularly from an evolutionary and functionally comparative standpoint.

Initial barnacle attachment occurs during cypris larvae settlement. After a serious of complex substratum exploration behavior, barnacle cyprids would determine the most suitable substratum and commit to permanent attachment by secreting larval bioadhesives from a pair of cement glands [2, 6, 7]. Once the cyprid is firmly attached, the larva immediately commits to metamorphosis and transforms into the juvenile stage [8]. The juvenile and adult remain attached to their substratum by continuously secreting adult cement proteins [9–12]. Some acorn barnacles (balanomorphan species) possess tubiferous calcareous bases that are physically cemented to the substratum [13]. This strategy is widely seen in species that adhere to ships and rocks and is believed to facilitate the strongest and firmest surface attachment [10, 12]. These calcareous bases remain intact on the substratum, even after the barnacles die. Other balanomorphan and stalked barnacles produce membranous and chitinous bases, which are only indirectly attached to the substratum via the cement [12]. The shell of these membranous-based species may detach from the substratum after they die [14]. Barnacles inhabiting biotic surfaces, such as corals and sponges, exhibit different attachment strategies. In coral-associated barnacles, their bases are embedded into and fused with the coral skeleton [15]. For sponge barnacles, their bases are cup-shaped and in contact with the living sponge tissue [16]. The underlying molecular mechanisms that have allowed barnacles to invade such a mesmerizing array of substrata have, however, remained elusive and poorly studied.

Adult barnacle adhesives are generated from cement glands, which are located close to the ovarian tissue and deliver cement to the basal region (Fig. 1a–d). A set of interconnecting principal and secondary ducts leads from the cement glands to the narrow interface between the barnacle base and the attachment substratum [17–20]. Barnacle adhesives are mainly composed of cement protein complex. A number of barnacle cement proteins identified from the fouling acorn barnacle species *Megabalanus rosa* and categorized based on their molecular weight; CP19k, CP20k, CP52k, CP68k and CP100k (CP denotes cement protein) are by far the five most studied cement proteins [21–27]. Their homologs have been identified in a range of rock-attaching barnacle species. However, functions of these CPs were only predicted based on protein primary structure, predicted pI (isoelectric point) and hydrophobicity of the homologs from *M. rosa* and *A. amphitrite* [7, 12]. In these two species, CP19k and CP43k (identical to CP68k) homologs were found to be hydrophobic and believed to be located at the barnacle cement and substrate boundary, thus making them responsible for the removal of the surface-bound water layer; CP20k was proposed to be responsible for interfacial adhesion [12], but its exact function is still controversial [7]; and CP52k and CP100k were considered bulk insoluble cement proteins that play important roles in internal cohesion [7].

As adult barnacles are structurally specialized to cement onto different substrata in different habitats and lifestyles [1–3, 28], whether and to what extent their adhesives display cross-species variation in their chemical composition and make-up are important questions [29]. In fact, the Raman spectrum of the cement of *Lepas anatifera*, which has a membranous base and attaches to a wide range of floating objects, is different from that of the acorn barnacle *Balanus crenatus*, which possesses a calcareous base and mainly attaches to rocky surfaces in the intertidal area [30]. Lin et al. [31] interestingly found signatures of interspecific variation in adhesive components of tetraclitid barnacles. These previous studies suggest that the compositions of cement proteins differ across species. Yet, it remains unclear if the gene expression level of key adult cement proteins varies across taxa and whether such signatures adaptively evolved to accommodate barnacle life on different substrata.

Although studies on the adhesive strategies of the stalked lepadid and pollicipedid barnacles have recently increased, they have traditionally focused on a few closely related biofouling model species, e.g., the balanid *A. amphitrite*, *A. eburneus* and *M. rosa* [29, 30, 32–35]. Understanding the diverse adhesive components of barnacles may shed further light on how barnacles achieve underwater attachment. In the present study, we used both histological and transcriptomic data to explore the cement glands and cement proteins on an unprecedented diversity of barnacles inhabiting both fire- and scleractinian corals, sponges, rocky shores, sea turtles, crabs and floating objects. We hypothesized that the composition and chemical properties of cement proteins from different species might vary according to the attachment substratum and/or the basal structure.

## Results

### Histological examination of cement glands

Cement glands (CGs) were examined from free living species (mostly attached to rocks), including the acorn barnacles *Amphibalanus amphitrite*, *Tetraclita japonica formosana*, *Chthamalus malayensis*; the stalked barnacle *Capitulum mitella*; epibiotic barnacles *Chelonibia testudinaria* (on sea turtles or crabs), *Megabalanus ajax* (on fire corals), *Galkinia* sp. (on corals), and *Membranobalanus longirostrum* and *Pectinoacasta* sp. (in sponges). Table 1 summarizes the coverage of the taxa, their lifestyle and habitat preferences.

**Table.1:** Characters of barnacle species examined in this study [14, 15, 36]

Species name	Abbr.	Morphology	Family	Primary substratum	Base material	Diameter of CGCs (µm)	Histochemistry of CGCs
*Amphibalanus amphitrite*	Aamph	Acorn	Balanidae	Intertidal, rocky shores	Calcareous	Up to 110	Eosinophilic
*Chelonibia testudinaria*	Ctest	Acorn	Chelonibiidae	Epibiotic, crabs/sea turtles	Membranous	34 to 70	Basophilic
*Chthamalus malayensis*	Cmala	Acorn	Chthamalidae	Intertidal, rocky shores	Membranous	Up to 80	Basophilic
*Galkinia* sp.	Galk	Acorn	Pyrgomatidae	Coral reefs, corals	Membranous	Not detected	Not detected
*Megabalanus ajax*	M*ajax*	Acorn	Balanidae	Coral reefs, fire corals	Calcareous	15 to 20	Basophilic
*Membranobalanus longirostrum*	Mlong	Acorn	Balanidae	Coral reefs, sponges	Membranous	Not detected	Not detected
*Pectinoacasta* sp.	Pect	Acorn	Balanidae	Coral reefs, sponges	Calcareous	Not detected	Not detected
*Tetraclita japonica formosana*	Tform	Acorn	Tetraclitidae	Intertidal, rocky shores	Calcareous	128 to 214	Eosinophilic
*Wanella milleporae*	Wmill	Acorn	Balanidae	Coral reefs, fire corals	Calcareous	Not detected	Not detected
*Capitulum mitella*	Cmite	Stalked	Pollicipedidae	Intertidal, rocky shores	Membranous	14 to 70	Basophilic
*Conchoderma hunteri*	Chunt	Stalked	Lepadidae	Neuston, floating objects	Membranous	Up to 135	Basophilic
*Lepas anatifera*	Lanat	Stalked	Lepadidae	Neuston, floating objects	Membranous	Up to 150	Basophilic

The CGs of the acorn barnacles *A. amphitrite*, *T. j. formosana*, *C. testudinaria*, *C. malayensis* and *M. ajax* are located in the basal mantle region and closely associated with the ovarian tissue. CG could be identified by the presence of unicellular cement gland cells (CGCs), which are structurally distinct from the ovarian follicle cells. The CGs of *M. ajax* are highly reduced and barely observable in histological sections. Cement gland structure could not be detected in the coral-associated barnacles *Galkinia* sp. and *Wanella milleporae* or the sponge-associated barnacles *M. longirostrum* and *Pectinoacasta* sp. through repeated sectioning of multiple specimens. The CG in these four coral- or sponge-associated species might be absent or highly reduced, or located in mantle tissue closer to the distal (upper) operculum plates.

On the other hand, the locations of CGs in the three examined stalked barnacle species (*Capitulum mitella, L. anatifera*, and *C. hunteri*) are variable. In the lepadid stalked barnacles *C. hunteri* and *L. anatifera*, the cement glands are located in the mantle and top region of peduncle; in the pollicipedid stalked barnacle *Capitulum mitella*, the cement glands are located in the ovary inside the peduncle.

**Fig. 2:**
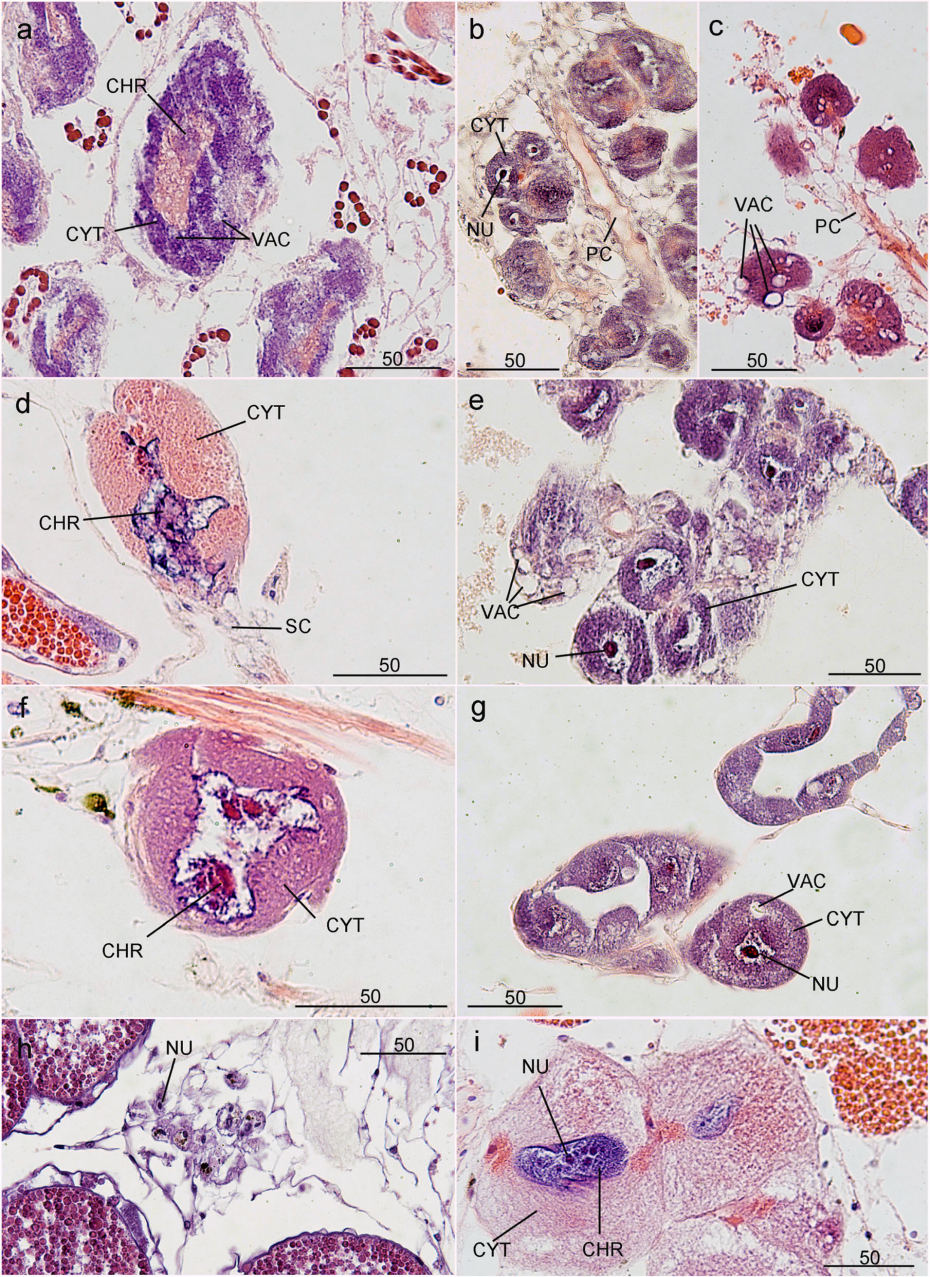
Hematoxylin and eosin staining of histological sections of barnacle cement glands.** a**. *Lepas anatifera*, **b** and **c**. *Capitulum mitella*, **d**. *Amphibalanus amphitrite*, **e**. *Chelonibia testudinaria*, **f. ***Chthamalus malayensis*, **g**. *Conchoderma auritum*, **h**. *Megabalanus ajax*, **i. ***Tetraclita japonica formosana*. CHR – chromatin, VAC – vacuole, NU – nucleolus, PC – principal canal, CYT – cytoplasm. Scale bars = 50 μm

The histochemical properties and the cellular diameter of CGCs are variable among the examined species. The CGCs of *L. anatifera* are up to 150 μm in diameter and have vacuoles in the basophilic cytoplasm (Fig. 2A); the CGCs of *C. mitella* occur in clumps and are linked by principal and secondary canals, ranging from 14 to 70 μm in diameter, and contain vacuoles among the basophilic cytoplasm and large single nucleolus in the nuclei (Fig. 2B and C); the CGCs of *A. amphitrite* have diameters up to 110 μm and eosinophilic cytoplasmic contents (Fig. 2D); the CGCs of *C. testudinaria* range from 34 to 70 μm in diameter, with single distinct nucleolus and basophilic cytoplasmic contents (Fig. 2E); the CGCs of *C. malayensis* reach up to 80 μm in diameter, with small vacuoles inside the basophilic cytoplasm (Fig. 2F); *C. hunteri* has large CGCs with diameter up to 135 μm, and large sized vacuoles inside the basophilic cytoplasm (Fig. 2 G); the diameter of CGCs of *M. ajax* range from 15 to 20 μm and are only found scarcely scattered in the basal mantle tissue (Fig. 2 H), and the cytoplasmic contents are basophilic; the CGCs of *T. j. formosana* are the largest, with a diameter ranging from 128 to 214 μm with multiple nucleoli in the eosinophilic cytoplasm (Fig. 2I). The diameter and the histochemical property of the CGCs of different species are summarized in Table 1.

### Phylogenomic analysis

**Fig. 3:**
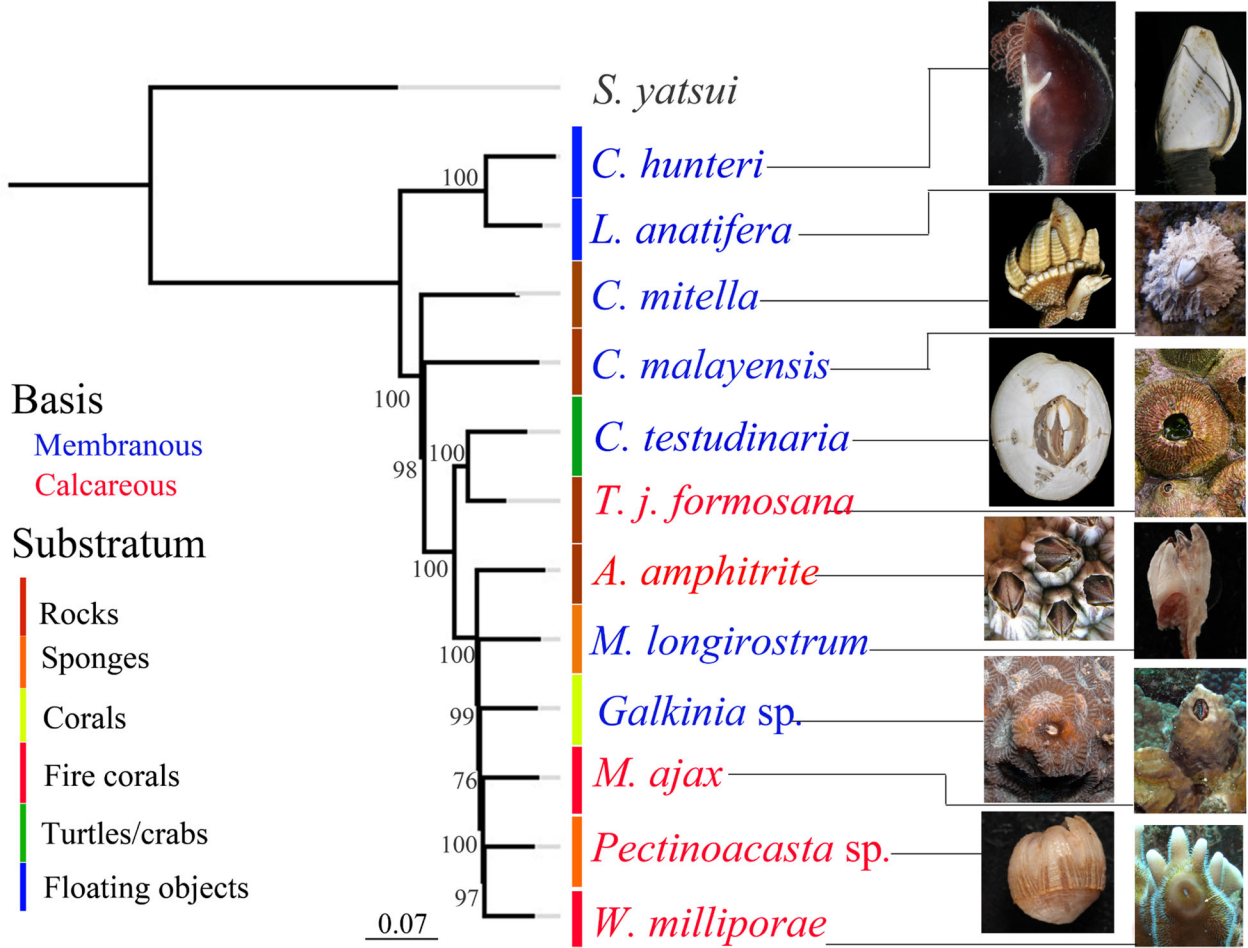
Phylogeny, attachment substratum and base feature of the examined barnacle species. Maximum-likelihood phylogenetic tree constructed from 278 single-copy orthologs in the 14 examined Cirripedia species as identified by Orthofinder. The number on each node signifies the bootstrap support value out of 100 replications. The attachment substratum of each barnacle species is indicated in the figure. Species with membranous and calcareous bases are indicated in blue and red, respectively

For each examined barnacle species, a transcriptome assembly was generated using Illumina reads from the prosoma and base (see Additional file 1). The statistics of the assemblies are shown in Table 2. Based on the translated protein database generated from the transcriptomes of thirteen barnacle species, 45,913 orthogroups were identified, among which 6,348 were detected in all examined species and 278 were single copy orthologs (see Additional file 2 for detailed statistics). A maximum likelihood phylogenetic tree was generated based on the alignment of these single copy ortholog sequences (Fig. 3). Each node of the ML tree was supported by bootstrap values of 78–100 % (out of 100 replicates). With the parasitic rhizocephalan barnacle *S. yatsui* as the outgroup, two major sister clades were identified. The first clade included the two stalked barnacle lepadid species, *L. anatifera* and *C. hunteri*, and the second included the remaining species. Within the second clade, *C. mitella* (Pollicipedidae), which morphologically is considered a stalked barnacle, was the sister to the remaining acorn barnacles. Here, *C. malayensis* (Chthamalidae) was sister to two additional two sub-clades. The first sub-clade included *C. testudinaria* (Chelonibiidae) and *T. formosana* (Tetraclitidae), and the second included *Galkinia* sp. and *W. milleporae* (Balanidae), *A. amphitrite* and *M. ajax* (Balanidae), and *Pectinoacasta* sp. and *M. longirostrum* (Balanidae).

**Table.2:** Statistics on transcriptome assemblies of the 12 species examined in this study

Species name	Min. length (bp)	Max. length (bp)	N50 (bp)	Total number of transcripts	Total transcript bases (bp)
*Amphibalanus amphitrite*	201	20,231	749	215,314	1.22E + 08
*Tetraclita j. formosana*	201	15,218	810	164,141	1.01E + 08
*Chelonibia testudinaria*	201	23,466	2,207	52,981	7.79E + 07
*Chthamalus malayensis*	201	18,500	2,100	65,373	9.48E + 07
*Galkinia* sp.	201	23,999	2,189	70,524	1.05E + 08
*Megabalanus ajax*	201	24,170	2,202	49,211	7.26E + 07
*Membranobalanus longirostrum*	201	29,797	2,105	67,494	9.83E + 07
*Pectinoacasta* sp.	201	23,167	1,921	80,139	1.06E + 08
*Wanella milleporae*	201	14,491	2,030	83,406	1.18E + 08
*Capitulum mitella*	201	25,750	2,137	56,425	8.06E + 07
*Conchoderma hunteri*	201	21,142	1,126	187,935	1.70E + 08
*Lepas anatifera*	201	34,433	1,958	165,076	2.02E + 08

We mapped the primary substratum choice (corals, fire corals, floating objects, rocks, sponges, sea turtles/crabs) and the base material of the examined barnacles as listed in Table 1 onto the phylogenetic tree (Fig. 3). Symbioses and base material arose independently in our tree and are thus not monophyletic.

### Identification of barnacle adult cement proteins homologs

We first concluded the presence or absence of five CPs identified from *Megabalanus rosa*—CP19k, 20k, 43k, 52k, and 100k homologs—in our transcriptome dataset (Table 3). The transcript expression level of CP homologs and biochemical properties of the translated protein such as estimated molecular weight, protein isoelectric points (pI) and GRAVY hydropathy index are summarized in Additional file 3. Absence of CP homologs in our dataset indicated failure in detecting the mRNA expression signal by RNAseq or the lack of expression of the particular CP homologs, but does not necessarily imply that the CP gene was lost from the genome.

**Table.3:** A summary of the cement proteins detected in this study

Species name	CP19k	CP20k	CP43k^+^	CP52k	CP100k
**Acorn barnacles**					
*Amphibalanus amphitrite*	V	V	V	V	V
*Chelonibia testudinaria*	V		V	V	V
*Chthamalus malayensis*	V	V	V	V	V
*Galkinia* sp.					
*Megabalanus ajax*	V	V	V		V
*Megabalanus rosa*	V	V	V^+^	V	V
*Membranobalanus longirostrum*				V	V
*Pectinoacasta* sp.			V		
*Tetraclita j. formosana*	V			V	V
*Wanella milleporae*			V	V	V
**Stalked barnacles**					
*Capitulum mitella*	V	V	V	V	V
*Conchoderma hunteri*		V	V	V	
*Lepas anatifera*					V

^+^: So et al. [27] suggested that the 68KDa band identified by Kamino et al. [25] is homologous to AaCP43

No cement protein was identified from the transcriptome assembly of the coral barnacle *Galkinia* sp. CP100k homologs were detected in 10 of the sample species—all except the coral barnacle *Galkinia* sp. and the sponge barnacle *Pectinoacasta* sp.; CP52k homologs were detected in *C. testudinaria*, *C. malayensis*, *C. mitella*, *C. hunteri*, *L. anatifera*, *M. longirostrum*, *T. j. Formosana*, and *W. milleporae*, but were not detected in *M. ajax*, *Galkinia* sp. or *Pectinoacasta* sp. CP43k homologs were detected in *C. testudinaria*, *C. malayensis*, *C. mitella, C. hunteri*, *Pectinoacasta* sp., *L. anatifera, M. ajax*, and *T. j. formosana*, but not in *M. longirostrum*, *Galkinia* sp., or *W. milleporae*; CP20k homologs were detected in *A. amphitrite, C. hunteri, C. mitella*, and *C. malayensis*; CP19k homologs were detected in *A. amphitrite, C. hunteri, C. mitella*, and *C. malayensis* (Table 3).

### Cement protein homologs

There were 33 CP19k, 17 CP20k, 28 CP43k, 57 CP52k and 39 CP100k homologs detected from the transcriptomes of the 12 examined barnacle species (Additional file 3). Alignment files of all detected homolog sequences of each CP were provided in Additional file 4. Among the CP homologs, we distinguished transcript isoforms and paralogues based on (1) the TRINITY transcript ID information, (2) protein sequence alignment data, and (3) the orthogroup assignment. For example, CP114k is a known paralogue of CP100k [37]. Although the two proteins were assigned to the same orthogroup (OG OG0001681, see Additional file 3), they only shared about 58 % and did not share any identical region in the alignment (refer to Additional file 3 in [37]).

After removing transcript isoforms based on the sequence similarity and transcript ID information (see Materials and Methods), there were 29 CP19k, 16 CP20k, 20 CP43k, 45 CP52k and 39 CP100k homologs. The CP homolog sequences were assigned to four orthogroups of CP19k, three of CP20k, four of CP43k, six of CP52k, and three orthogroups of CP100k by Orthofinder (Additional files 3 and 5). Certain species have remarkable numbers of CP paralog sequences. For example, 11 CP19k paralog sequences were detected in the *C. malayensis* transcriptome, and 22 and 14 CP52k were detected from *M. longirostrum* and *C. testudinaria* transcriptomes, respectively (Additional file 5).

### mRNA expression pattern of cement protein homologs

**Fig. 4:**
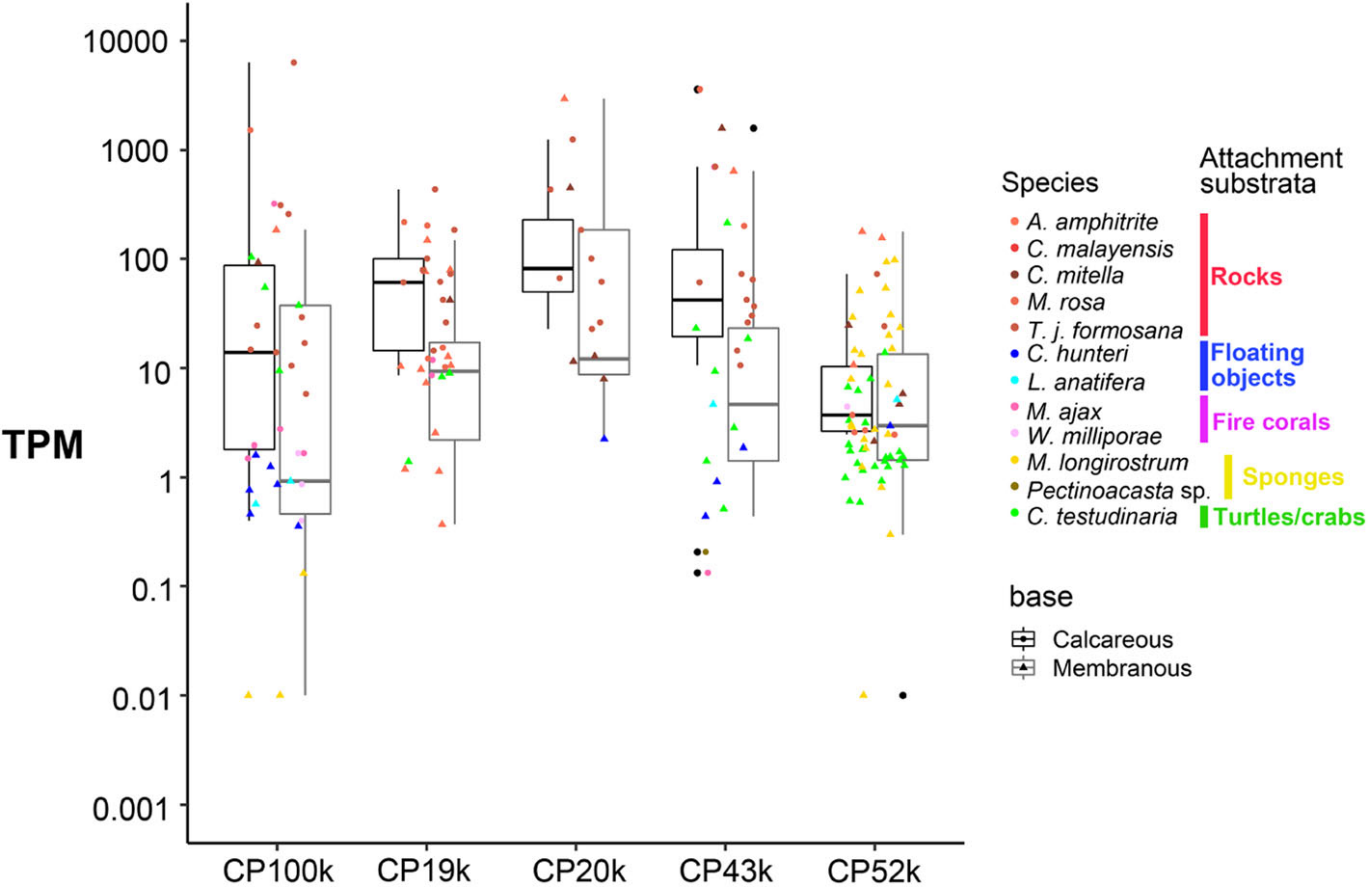
mRNA expression level of CP homologs. The vertical dot plot presents the mRNA expression level of CP homologs in the base or the peduncle. The corresponding species of each CP homolog is indicated by different colors. Grouping of the attachment substratum refers to Table 1. The grey box plot and black box plot illustrate the 75th percentile, the median (50th percentile) and 25th percentile of the expression level (in TPM) of different CPs of calcified-based and membranous-based barnacles examined in this study, respectively

The transcripts of CP homologs were generally highly expressed at the base compared to its prosoma counterpart in each species (Additional file 6). The mRNA expression CP homologs were often 10 times or higher in the base or peduncle compared to the prosoma, but a few exceptions were found. The mRNA expression level of different CP homologs in different species could vary by over 1000-fold (Fig. 4). In the majority of the examined acorn barnacles, the transcript expression level of at least one of the CP homologs exceed 10 TPM (Transcript Per Million read). In *C. hunteri* and *L. anatifera*, however, none of the CP homolog transcript expression levels exceeded 10 in the peduncle (Fig. 4), even though the expression levels were generally higher in the peduncle (Additional files 3 and 6). The mRNA expressions of CP homologs in calcareous based species were not significantly different from membranous-based species (Fig. 4).

### Amino acid composition of CP homologs

**Fig. 5:**
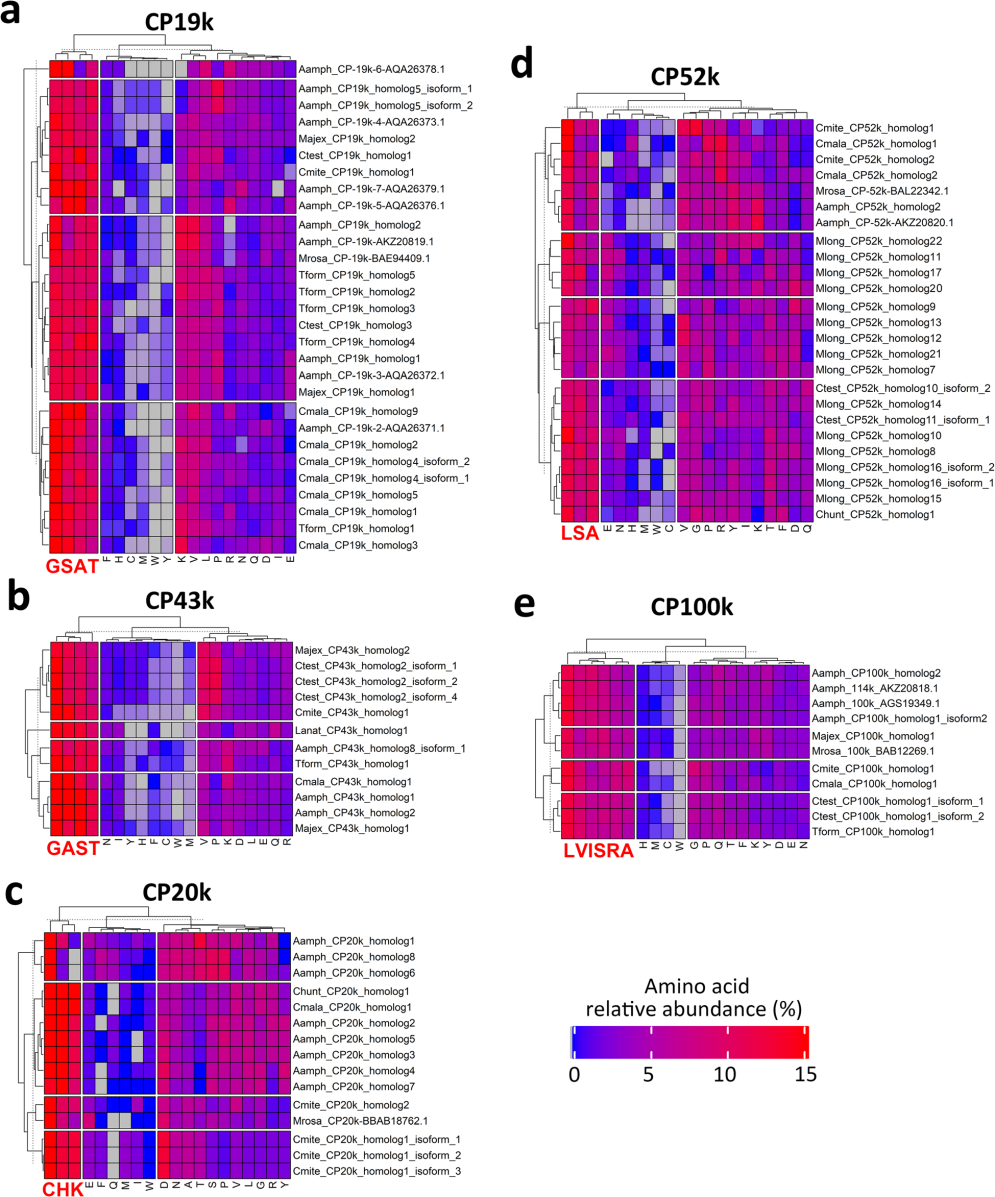
Amino acid (AA) composition of all full-length CP homolog sequences.** a**. CP19k homologs, **b**. CP43k homologs, **c**. CP20k homologs, **d**. CP52k homologs, **e**. CP100k homologs. The color scale shows the relative abundance of the amino acids in percentage with respect to the total number of amino acids of the CP homologs. The amino acid columns are three clusters based on the k-mean clustering method (k-mean cluster = 3) to visualize amino acid bias among CP homologs. Enriched amino acids are highlighted in enlarged red characters. K-mean clustering of CP homologs was also performed (row clustering) to observe similarity in amino acid composition among homologs. Abbreviations: Aamph – *Amphibalanus amphitrite*, Ctest – *C. testudinaria*, Cmite – *C. mitella*, Cmala – *C. malayensis*, Chunt – *C. hunteri*, Mrosa – *Megabalanus rosa*, Majax – *Megabalanus ajax*, Tform – *Tetraclita japonica formosana*

We only included full length CP homologs in the amino acid composition analysis, pI and hydropathy index predictions. The amino acid composition of CP homologs revealed similarities between CP19k and CP43k, CP52k and CP100k, and the unique property of CP20k homologs. Except for CP20k, all examined CP homologs were low (relative abundance below 1 %) in Cysteine (C), Histidine (H), Methionine (M), and Tryptophan (W). Different CP homologs showed clear bias (average relative abundance > 10 %) towards different amino acids (Fig. 5). CP19k (Fig. 5A) and CP43k (Fig. 5B) homologs shown a clear bias towards Glycine (G), Serine (S), Alanine (A), and Threonine (T); the biochemical profile of CP20k homologs was different from the other CPs, in which the homologs in general bias towards Cysteine (C), Histidine (H), and Lysine (K) (Fig. 5C); CP52k (Fig. 5D) and CP100k (Fig. 5E), on the other hand, were biased towards Leucine (L), Serine (S), and Alanine (A); CP100k homologs also showed clear bias towards Valine (V), Isoleucine (I), and Arginine (R) (Fig. 5E).

The CP homologs of certain species showed remarkable diversification in terms of the amino acid composition. For example, CP19k homologs of *A. amphitrite* were divided into four different clusters, among which one of the previously reported CP19k homolog, CP19k-6 (accession no. AQA26378, reported in [38]), was distinct from the other CP19k homologs (Fig. 5A); CP20k homologs of *A. amphitrite* were divided into two clusters, one of which was rich in Cysteine (C), Histidine (H), and Lysine (K), while the other was rich in Cysteine (C) only (the uppermost row cluster in Fig. 5C); CP52k homologs of *M. longirostrum* were divided into three clusters (Fig. 5D).

### Chemical properties and motif structures of CP homologs

**Fig. 6:**
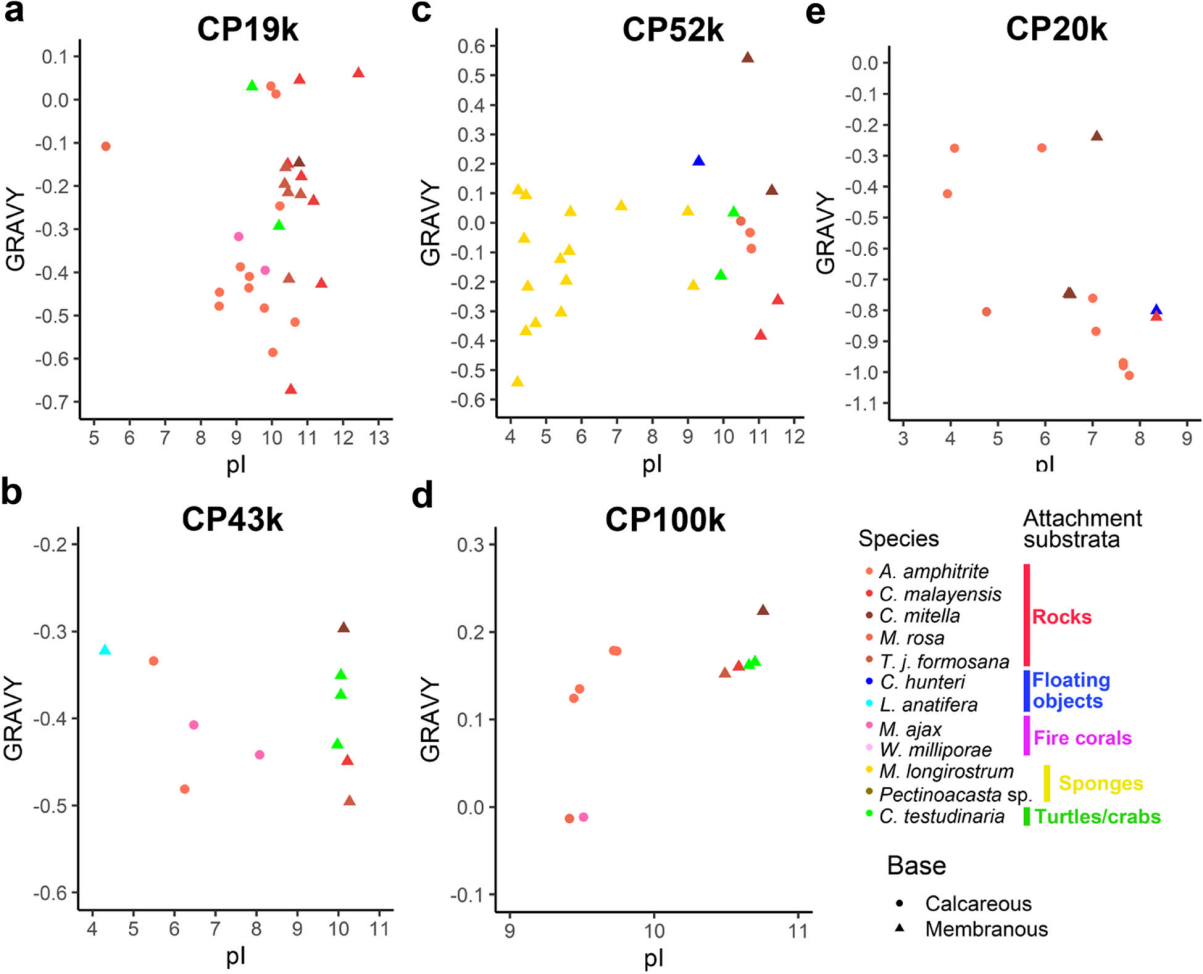
The predicted chemical properties of CP homologs. The predicted pI and GRAVY index of **(A)** CP19k homologs, **(B)** CP43k homologs, **(C)** CP52k homologs, **(D)** CP100k homologs, and **(E)** CP20k homologs. The corresponding species of each CP homolog is indicated by different colors. Grouping of attachment substratum refers to Table 1. Species with calcareous and membranous bases are indicated with circular and triangular nodes in the scatterplot

**Fig. 7:**
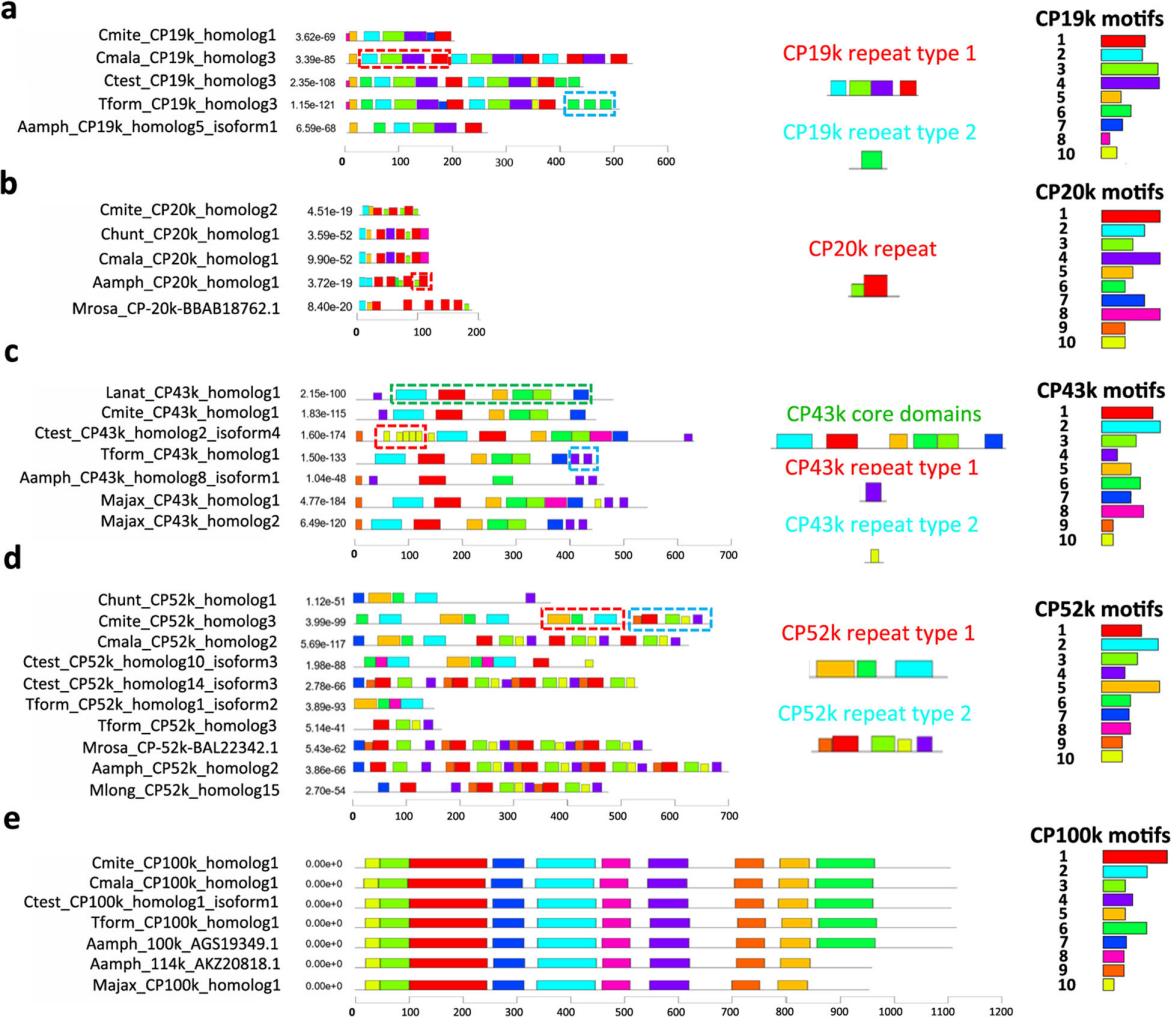
Schematic diagram for MEME motif structure of selected CP homologs.** A**. CP19k homolog, **B**. CP20k homologs, **C**. CP43k homologs, **D**. CP52k homologs, **E**. CP100k homologs. MEME was set to present the top 10 most significant motif regions among the homolog of each CP. Each motif region was numbered 1 to 10, where motif 1 has the lowest P-value in MEME. Protein repeats are highlighted and the motifs regions within these repeats are shown in each subfigure. Abbreviations: Aamph – *Amphibalanus amphitrite*, Ctest – *C. testudinaria*, Cmite – *C. mitella*, Cmala – *C. malayensis*, Chunt – *C. hunteri*, Lanat – *Lepas anatifera*, Mrosa – *Megabalanus rosa*, Majax – *Megabalanus ajax*, Tform – *Tetraclita japonica formosana*

Correlations between the chemistry of CP with the attachment substrata, predicted protein pI, and protein hydropathy index of the recovered CP homologs (full length proteins only) were analyzed (Fig. 6). The internal conserved motif structures of paralog sequences were identified for all CPs (Fig. 7, Additional file 4: Files S1–5). The paralogous sequences within the top 10 MEME motif regions were aligned for each CP homolog (Additional file 4: Files S6–10) and the motif consensus sequences of all CPs were summarized in Additional file 7: Figure S6.

#### CP19k homologs

Full-length CP19k homologs were successfully recovered from the transcriptome of *C. malayensis, C. testudinaria, C. mitella*, *T. j. formosana, A. amphitrite, L. anatifera*, *M ajax* and, from NCBI, *M. rosa*. The GRAVY index of full-length CP19k homologs from rock-attaching barnacles (*C. malayensis* and *C. mitella*), crab/sea turtle attaching *C. testudinaria*, and the fire coral inhabiting barnacle *M. ajax* ranged from a highly hydrophilic value − 0.67 to 0.06, and the predicted pI of all except *M. rosa* CP19k ranged from 8.51 to 12.4 (Fig. 6A, Additional file 3). The predicted pI of CP19k in the rock-attaching *M. rosa* was 5.43. The predicted pI of full-length CP19k homologs of membranous-based species—including *C. malayensis, C. testudinaria, C. mitella*, and *T. j. formosana*—ranged from 9.4 to 12.4, while those of calcareous-based species—including *A. amphitrite. M. ajax*, and *M. rosa—*ranged from 5.4 to 10.6. Internal repeating motif regions were observed within the paralogs (Additional file 7: Figure S1).

#### CP20k homologs

Full-length CP20k homologs were successfully recovered from the transcriptome of *C. malayensis, C. mitella*, *C. hunteri*, *A. amphitrite* and, from NCBI, *M. rosa*. The predicted pI of CP20k homologs ranged from 3.9 to 8.3 and the GRAVY index ranged from − 1 to -0.2, indicating that these proteins are hydrophilic and can be slightly acidic in certain species (Fig. 6E, Additional file 3). The predicted pI of CP20k homologs of membranous-based species (*C. malayensis, C. mitella*, and *C. hunteri*) ranged from 6.5 to 8.3, while those of the calcareous-based species (*A. amphitrite* and *M. rosa)* ranged from 3.9 to 7.8. Internal repeating motif regions were observed within the paralogs (Additional file 7: Figure S2).

#### CP43k homologs

Full-length CP43k homologs were successfully recovered from the transcriptome of *C. malayensis, C. testudinaria, C. mitella*, *T. j. formosana, A. amphitrite, L. anatifera*, and *M. ajax*. The GRAVY index of full-length CP43k homologs were from − 0.49 to -0.29 and the predicted pI ranged was 4.3 to 10.2, indicating that the homologs were in general hydrophilic. The CP43k homolog from *L. anatifera* was the most acidic (predicted pI: 4.3) (Fig. 6B, Additional file 3). The predicted pI of full-length CP43k homologs of all the membranous-based species except *L. anatifera*—i.e., *C. malayensis, C. testudinaria, C. mitella*, and *T. j. formosana—*exceeded 10, while those of the calcareous-based species—including *A. amphitrite* and *M. ajax—*ranged from 5.5 to 8. Internal repeating motif regions were observed within the paralogs (Additional file 7: Figure S3).

#### CP52k homologs

Full-length CP52k homologs were successfully recovered from the transcriptome of *C. malayensis, C. testudinaria, C. mitella*, *C. hunteri*, *M. longirostrum, A. amphitrite* and, from NCBI, *M. rosa*. The GRAVY index of full-length CP52k homologs ranged from − 0.55 to 0.55 and predicted pI ranged from 4.2 to 11.5 (Fig. 6C, Additional file 3). The predicted pI of CP52k from rock-attaching species (*C. malayensis, C. mitella, A. amphitrite*, and *M. rosa*) ranged from 10.5 to 11.5, exhibiting a potential bias towards basic pI. The predicted pI of CP52k homologs of the sponge-inhabiting barnacle *M. longirostrum*, on the other hand, ranged from 4.2 to 9.2. The GRAVY index of *M. longirostrum* CP52k homologs ranged from − 0.6 to 0.1, while in the rock-attaching barnacles, the CP52k homologs ranged from − 0.4 to 0.6, indicating a more diverse chemical property. Hence, the chemical properties of *M. longirostrum* CP52k homologs were distinct from those of other species. Internal repeating motif regions were observed within the paralogs (Additional file 7: Figure S4).

#### CP100k homologs

Full-length CP100k homologs were successfully recovered from the transcriptome of *C. malayensis, C. testudinaria, C. mitella*, *T. j. formosana*, *M. ajax*, *A. amphitrite* and, from NCBI, *M. rosa*. The majority of the homologs from different species were hydrophobic, except the homolog from fire coral-inhabiting *M. ajax* and the congeneric rock-attaching species *M. rosa* (Fig. 6D, Additional file 3). The GRAVY index of the CP100k homolog of these two *Megabalanus* species were slightly below zero, indicating that the CP100k homologs were somewhat amphiphilic. The predicted pI of all full-length CP100k homologs were basic, ranging from 9.4 to 10.75, but the homologs of membranous-based species (*C. malayensis, C. testudinaria, C. mitella*, and *T. j. formosana*) were more basic (pI: 10.5 to 10.75) than the calcareous-based species (*M. ajax*, *A. amphitrite*, and *M. rosa*, pI: 9.4 to 9.7). Unlike the other CPs, no internal repeat motif region was observed within these paralogs (Additional file 7: Figure S5).

## Discussion

Our histological and molecular data supported our hypothesis that cement protein composition and their chemical properties in different species vary according to the attachment substratum and/or the barnacle basal attachment surface. Based on the histological results, we found that the diameter and the histochemistry of CGCs in the examined species were highly variable. While the diameter of CGC could vary in relation to the body size of the species, it is intriguing that not all CGCs showed the same histochemical properties. Cytoplasmic content typically contains a mixture of intracellular proteins with different chemical properties. However, given that CGCs should be dedicated to the production of CP, the histochemical properties of CGCs could serve as a preliminary indicator that reflect the average chemical properties of the barnacle adhesives, where basophilic and eosinophilic staining indicate the acidic and basic nature of the CGC cellular content. It is intriguing that the CGCs of the calcareous-based rock-attaching barnacles *A. amphitrite* and *T. j. formosana* were eosinophilic, while other species with observable CGCs were basophilic. Our histological section data in general support our hypothesis that the chemical properties of cement of different species vary according to the attachment substratum and/or the basal structure, although how the chemistry of cement is related to cementation on different surfaces remains unclear.

It is worth noting that the failure to observe CG in the histological sections of four coral- or sponge-associated barnacle species may not necessarily indicate the absence of CG in these species. CGs are considered a major feature of barnacle ecological success in space and time, and it is not clear what coral symbiotic barnacles gain by losing them. One explanation may revolve around the coral and sponge barnacles that inhabit the interior skeleton or tissues of their hosts—in this case, the barnacle is more or less physically fixed to the coral skeleton or within sponges. Compared to other examined species, the morphologies of these four species are different and their basal structures are not flat but modified into a cup-shape convex surface (see Fig. 1F). The CGs of these species might be distributed more distally in the mantle region. The presence of CG in the sponge barnacles *M. longirostrum* and *Pectinoacasta* sp. and the fire coral inhabiting *W. milliporae* was supported by the transcriptomic data, in which CP transcript expressions were detected. However, we do not rule out the possibility that CG is absent in *Galkinia* sp., as CG and CP were not detected in histological or transcriptomic analyses.

Based on the transcriptomic results, we observed a diversity of CP homologs. Based on sequence alignment, a remarkable number of conspecific CP sequences were found to be paralogues. The presence of CP paralogues were previously reported in *A. amphitrite* [38]. In this study, we observed more paralogous sequences of CP19k, 20k, 43k, and 52k compared to CP100k. Interestingly, the mRNA expression level of these paralogs exhibited remarkable variations, suggesting that their requirement in cementation might vary from each other. Species that attached to similar substrata also showed major variations in their cement protein expressions. For instance, only one CP43k homolog was detected in the transcriptome of a sponge inhabiting the barnacle *Pectinoacasta* sp., and its mRNA expression was below 1 TPM (Transcript Per Million read), while in another sponge barnacle—*M. longirostrum—*mRNA expressions of CP52k and CP100k homologs were detected, and the mRNA expression levels were 10 to 100 times higher. Such variation suggests that the composition of cement proteins can be highly variable even for species that attach to similar substrata.

In addition to variations in gene expression level, the predicted pI and hydrophobicity of CP19k, CP20k, CP43k, and CP52k homologs/paralogs exhibited remarkable divergence. CP100k, on the other hand, exhibited remarkable convergence in terms of amino acid composition, estimated pl, and hydrophobicity, suggesting that CP100k is a core component with indispensable function in barnacle adhesives. In *A. amphitrite*, hydrophobic CP19k and CP43k paralogs were suggested to serve as a water-repellent to remove non-polar substances on the surface of the substratum and subsequently mediate the formation of fibrous cement protein plaque [7, 38]; CP20k was suggested to play an important role in interfacial adhesion [12]. However, in this study, most of the examined full length CP homologs were recovered from rock-attaching species, and our results hint to a potential linkage between barnacle base material (calcareous/membranous) and the overall charge (predicted pI) of CP19k, CP20k, CP43k, and CP52k. On the other hand, we are aware that this result is, at this stage, insufficient to conclude such a correlation, owing to the limited number of examined species. Further studies with increased taxa coverage are necessary to elucidate the possible linkage between the overall charge of CP and base material.

In certain species, multiple sequences of CP19k, CP20k, CP43k and CP52k were assigned to more than one orthogroups. For example, 22 CP52k homologs from *M. longirostrum* were assigned to six different orthogroups, with two to six CP52k homologs assigned to each orthogroup. The results suggest that (1) there were significant duplications of the CP52k gene and (2) the resulting CP52k paralogous sequences have more than one ortholog within the examined species. Remm and colleagues referred to such a phenomenon as an “in-paralog” in which gene duplication occurred after a speciation event in one genome but not the orthologous counterpart in the other genome [39]. More interestingly, internal repeating motif regions were observed within the paralogues of CP19k, CP20k, CP43k, and CP52k (Fig. 7), suggesting that intragenic domain duplication occurred in addition to gene duplication. These repetitive motifs were conserved across the examined taxa but the number of motifs vary in different species, suggesting a common ancestral origin but random duplication of these motif regions in different species. Protein repeats could arise from exon duplications and/or transitions from existing genes within proteins that process regular secondary structures. The variations in numbers of repeats indicate the rapid loss and/or gain of repeats in the evolution of CP19k, CP20k, CP43k, and CP52k in different barnacle species. On the other hand, relatively fewer paralogs and a lack of internal repeat structures among CP100k sequences of the examined species suggested a completely different evolutionary constraint on this CP.

Protein repeats possess regular secondary structures and form multirepeat assemblies in three dimensions of diverse sizes and functions. They often have specific roles, such as protein-protein interaction, protein-biomolecule binding, and formation of fibrous structures. Examples of important protein repeats include Leucine-rich repeats in pattern recognition proteins, PPxxPxPPx repeats in Homeobox protein (summarized in [40]), GXY (where X and Y are frequently occupied by Pro and Hyp, respectively) or PXG repeats in collagen [41] and GA repeats in silk proteins [42]. In squid teeth, the mechanical properties of squid ring teeth proteins increase as a function of the number of tandem repeats [43]. For barnacle cement proteins, those repetitive regions may directly relate to substrate binding capacity because the presence of internal repetition increases the available binding surface area of the protein.

While the exact functions of those repetitive motifs in CPs remain unclear, the remarkable CP gene duplication and domain duplication of CP19k, 20k, 43k, and 52k have resulted in a pool of CPs with diverse chemical and biochemical properties. This is best illustrated by *M. longirostrum* CP52k paralogs, which are highly diverse in term of the overall protein charge and hydrophobicity. *M. longirostrum* is embedded and attached to sponge tissue, which is a composite of organic matrix and inorganic sponge spicules. Development of a highly diverse cement protein cocktail may enable the barnacle to accommodate environments with a variety of substrata. A remarkable repertoire of CP paralogs was also recovered from the rock-attaching species *C. malayensis* and *A. amphitrite*, and the sea turtle-inhabiting barnacle *C. testudinaria*, suggesting that duplications of CP19k, 20k, 43k, and 52k gene might be a common genetic feature in thoracican barnacles.

## Conclusions

In conclusion, our results suggest that cement protein composition and its chemical properties might vary according to the base material of the barnacle species. Instead of developing highly specific cement proteins, barnacles seem to attach to different substrata by relying on a highly duplicated genetic toolkit that generates paralogous CP sequences with diverse chemical and biochemical properties. Such versatility may explain why many barnacle species are extremely cosmopolitan and diverse in their substratum choice.

## Methods

### Sample collection

A total of 12 barnacle species were collected from a wide variety of taxonomic groups covering diverse morphologies, host substrata, and basic materials (Fig. 1; Table 1). Specimens of the intertidal stalked barnacle *Capitulum mitella*, acorn barnacle *Amphibalanus amphitrite*, and membranous base high-shore barnacles *Chthamalus malayensis* and *Tetraclita japonica formosana* were collected from rocks in Shen-Ao-Kang, Taiwan (25°12’36.59 N, 121°81’97.66E). Specimens of coral reef barnacles, including *Megabalanus ajax* (on fire corals), *Membranobalanus longirostrum* (in sponges), *Pectinoacasta* sp. (in sponges), *Wanella milleporae* (on fire corals) and *Galkinia* sp. (on scleractinian corals) were collected from Green Island, Taiwan (22°40’45.03 N, 121°30’11.98E). Specimens of the oceanic epibiotic barnacles *Chelonibia testudinaria*, *Conchoderma hunteri* and *Lepas anatifera* were collected from crabs (*C. testudinaria*) at fish markets, ropes underneath buoys (*C. hunteri*) and floating pieces of wood (*L. anatifera*) in Taiwan.

### Histological examination of cement glands

The somatic body (= prosoma) and ovarian tissue (which often mix with cement glands), located at the base region of barnacles, were fixed in 10 % formalin in seawater for one week, and rinsed in freshwater for 10 min before dehydration. The tissues were dehydrated in ascending concentrations of ethanol (75, 95, and 100 % for serial dehydration for 1 h for each concentration), then immersed in xylene (3 h) and embedded in paraffin. The specimens were then cut at a sagittal angle into 10-µm sections using a microtome (Leica). Hematoxylin (4 min) and eosin (2 min) (HE) staining were performed on distinct paraffin sections (see Kiernan 2008 for details on the histological preparation methods). The HE-stained sections were observed using a compound microscope, and cement glands were identified following the morphological description in [18].

### RNA extraction, cDNA library preparation, and sequencing

For ten of the 12 species (all except *A. amphitrite* and *T. j. formosana*, for which transcriptome data had already been published), tissue samples (single individual of each species) of the prosoma and base/peduncle (where the cement gland is located) were carefully isolated without contaminating each other. The total RNA of the prosoma and base was extracted using TRIzol® Reagent (Invitrogen, Camarillo, CA) and High Pure RNA Isolation Kit (Roche Applied Science, Germany), respectively. RNA quality assessment was conducted by a Bioanalyzer 2100 with RNA 6000 labchip kit (Agilent Technologies, Santa Clara, CA, USA). cDNA libraries for both parts of all 10 species were prepared using Illumina TruSeq RNA Sample Prep Kits v2 and subsequently sequenced by HiSeq™ 2500 High-Throughput Mode v4 with paired-end 125 base-pair reads located at the High throughput Genomics Core, Biodiversity Research Center, Academia Sinica, Taiwan according to the manufacturer’s instructions (Illumina, San Diego, CA).

### Sequence de novo assembly, annotation, and mRNA expression pattern

For cross-species comparison, RNA reads from both prosoma and base/peduncle samples in each species were combined into a single input to obtain a consolidated set of contigs. The sequence read quality was checked with FastQC v0.11.5 (Babraham Bioinformatics, Cambridge, UK) and filtered using the Trimmomatic v0.35 [44] to discard adaptor sequences and low-quality reads. The trimmed reads were then applied to do *de novo* assemblies with Trinity v2.2.0 [45]. Contigs with amino acid sequence similarity higher than 80 % were clustered and regarded as reference sequences, or transcripts. Transcripts with a sum of read counts from base/peduncle and prosoma of no more than 1 were discarded. Gene annotation of each transcript was performed by BLASTx via information from the NCBI Arthropoda database and non-redundant protein database with an *E* value of 1e-6 as the cutoff point. Gene expression level was quantified by mapping all short reads of paired-end data to the transcripts in Bowtie [46]. Then RSEM (RNA-Seq by Expectation Maximization) [47] was used to calculate FPKM (fragments per kilobase of transcripts per million mapped reads) values. The FPKM measure normalized the raw reads of each contig to avoid bias from transcript length and sequencing level. The differential expression level between base and prosoma was tested with DESeq2 [48] using negative binomial generalized linear models. Salmon [49] was used to calculate the TPM (Transcripts Per Million read) values of transcripts for subsequent comparative analyses.

### Ortholog clustering

Prior to the *in-silico* protein translation step, transcripts with FPKM below 0.01 in both base and prosoma were removed. From the transcriptome assembly of each examined species, a translated protein database was generated from candidate coding regions within contigs using Transdecoder v3.0.0 [45]. BLASTp was performed on the translated protein databases against an assembly of translated protein sequences predicted from the genome data of the acorn barnacle *Amphibalanus amphitrite* [50] using Diamond v0.9.30.131 [51], with the default *E* value setting and -max_target_seqs set to 1. The BLASTp tubular output file was used as the reference file for the parameter --retain_blastp_hit in the TransDecoder.Predict step. Completely identical protein sequences in the translated protein database of each species were removed by cd-hit with an identity threshold of 1 (-c 1). Ortholog identification was performed using Orthofinder with default settings [52]. Single copy orthologs were aligned and a maximum-likelihood (ML) phylogenetic tree was constructed (with 100 bootstraps) as described in Lan et al. [53]. Transcriptome data of parasitic barnacle *Sacculina yatsui* (BioProject no. PRJDB8012) was incorporated as the outgroup to the examined thoracican barnacle species.

### Barnacle cement protein identification and bioinformatics

Barnacle cement proteins were recovered from the NCBI webpage interface for protein sequences (https://www.ncbi.nlm.nih.gov/protein/) using the search command “(cement protein [Protein Name] OR (cement[All Fields] AND protein[All Fields])) AND “Thoracica“[ORGN]”. Barnacle cement protein sequences from *Amphibalanus amphitrite* and *Megabalanus rosa* were selected and downloaded for the subsequent analyses. The barnacle cement proteins were named based on Kamino et al. [23], who labeled the identified protein bands based on the estimated molecular weight in the SDS-PAGE. These *M. rosa* cement proteins included CP19k, CP20k, CP52k, CP68k (not deposited to NCBI) and CP100k, in which the number and “k” reflect the estimated molecular weight of the protein in kiloDa (kDa). This naming system was adopted for all subsequent studies relating to these reported barnacle cement proteins. So et al. [38] reported an extensive list of *M. rosa* cement protein homolog sequences. Their proteomic-transcriptomic approach identified three cement protein sequences from a 43 kDa protein band in their SDS-PAGE analysis. These 43 kDa proteins were reportedly homologous to a *M. rosa* 68 kDa protein (CP68k) [27]. Since *M. rosa* CP68k was not deposited into NCBI, we referred to the protein sequences homologous to *A. amphitrite* 43 kDa and *M. rosa* CP68k as “CP43k” homologs in the present study.

CP19k, CP20k, CP43k, CP52k, CP100k and CP114k (from *A. amphitrite*, reported by [38]) were used as the query in a tBLASTn search against the transcriptome (cDNA) databases, with an *E* value cutoff of 1e-5. Alignment of homologous barnacle cement protein sequences was performed using MUSCLE in MEGA7 [54]. The amino acid composition of each CP homolog sequence was computed using the function “readAAStringSet” in the R package “Biostrings” (Pages, [55]). Protein isoelectric points (pI) were predicted using the web-based interface of an IPC–isoelectric point calculator [56]. “Average pI” was taken as the predicted pI of the protein sequence. The GRAVY hydropathy index prediction was performed using GRAVY Calculator (http://www.gravy-calculator.de/index.php). Conserved motif identification was performed using MEME v5.2.0 [57], with the number of expected motifs set to 10 (-nmotifs 10), motif length range from 6 to 150 amino acid (-minw 6 -maxw 150), motif distribution set as “any number of repetition (anr)” (-mod anr) and the background model as zero order (-markov_order 0).

## Supplementary information

**Additional file 1**

**Additional file 2**

**Additional file 3**

**Additional file 4**

**Additional file 5**

**Additional file 6**

**Additional file 7**

## Data Availability

The datasets generated and/or analysed during the current study are available in this published article and its supplementary information files. The datasets are also available from the GenBank repository, Biosample account numbers SAMN13956698-701, SAMN16953520-23, SAMN16963732-34, SAMN17491171-74, SAMN17525968-71, SAMN17573517-20, SAMN17573696-99, SAMN17573706-09, SAMN17598410-13, SAMN17598515-18, SAMN18042803-06. https://www.ncbi.nlm.nih.gov/genbank/.
