# Supplementary material for: Histology and transcriptomic analyses of barnacles with different base materials and habitats shed lights on the duplication and chemical diversification of barnacle cement proteins
Source: BMC Genomics. 2021 Nov 1;22:783. doi: 10.1186/s12864-021-08049-4 (PMC8561864; doi:10.1186/s12864-021-08049-4)
Supplement: Supplementary file 1 — Additional file 1 [file 12864_2021_8049_MOESM1_ESM.docx]

Additional file 1. RNAseq sample information.

| **Species name** | **Base R1** | | **Base R2** | | **Prosoma R1** | | **Prosoma R2** | | |
| --- | --- | --- | --- | --- | --- | --- | --- | --- | --- |
|  | **no. of raw reads** | **Biosample acc no.** | **no. of raw reads** | **Biosample acc no.** | **no. of raw reads** | **Biosample acc no.** | | **no. of raw reads** | **Biosample acc no.** |
| *Amphibalanus amphitrite* | 13,181,352 | SAMN18042803 |  |  | 25,842,361 | SAMN18042804 | |  |  |
| *Tetraclita j. formosana* | 27,121,002 | SAMN18042805 |  |  | 26,658,879 | SAMN18042806 | |  |  |
| *Chelonibia testudinaria* | 21,286,012 | SAMN13956701 | 21,208,556 | SAMN13956700 | 22,476,472 | SAMN13956699 | | 22,452,606 | SAMN13956698 |
| *Chthamalus malayensis* | 20,447,498 | SAMN17573706 | 20,434,060 | SAMN17573707 | 20,639,112 | SAMN17573708 | | 20,589,694 | SAMN17573709 |
| *Galkinia* sp. | 23,142,504 | SAMN17573520 | 23,065,916 | SAMN17573519 | 21,664,310 | SAMN17573518 | | 21,632,528 | SAMN17573517 |
| *Megabalanus ajax* | 19,065,484 | SAMN16953520 | 19,023,398 | SAMN16953521 | 20,914,008 | SAMN16953522 | | 20,875,656 | SAMN16953523 |
| *Membranobalanus longirostrum* | 19,783,242 | SAMN16963732 | 19,741,630 | SAMN16963733 | 22,121,028 | SAMN16963734 | | 22,014,260 | SAMN16963734 |
| *Pectinoacasta* sp. | 21,081,616 | SAMN17491171 | 21,005,322 | SAMN17491172 | 19,686,100 | SAMN17491173 | | 19,636,484 | SAMN17491174 |
| *Wanella milleporae* | 20,616,904 | SAMN17525968 | 20,570,366 | SAMN17525969 | 18,192,154 | SAMN17525970 | | 18,192,466 | SAMN17525971 |
| *Capitulum mitella* | 17,868,008 | SAMN17573696 | 17,811,890 | SAMN17573697 | 22,517,214 | SAMN17573698 | | 22,446,300 | SAMN17573699 |
| *Conchoderma hunteri* | 21,116,722 | SAMN17598515 | 21,023,424 | SAMN17598516 | 21,807,484 | SAMN17598517 | | 21,730,578 | SAMN17598518 |
| *Lepas anatifera* | 20,899,894 | SAMN17598410 | 20,786,652 | SAMN17598411 | 21,669,994 | SAMN17598412 | | 21,577,866 | SAMN17598413 |
