# Supplementary material for: Histology and transcriptomic analyses of barnacles with different base materials and habitats shed lights on the duplication and chemical diversification of barnacle cement proteins
Source: BMC Genomics. 2021 Nov 1;22:783. doi: 10.1186/s12864-021-08049-4 (PMC8561864; doi:10.1186/s12864-021-08049-4)
Supplement: Supplementary file 2 — Additional file 2 [file 12864_2021_8049_MOESM2_ESM.docx]

**Additional file 2: Orthofinder results and statistics.**

| Number of species | 13 |
| --- | --- |
| Number of genes | 554,298 |
| Number of genes in orthogroups | 435,698 |
| Number of unassigned genes | 118,600 |
| Percentage of genes in orthogroups | 78.6 |
| Percentage of unassigned genes | 21.4 |
| Number of orthogroups | 45,914 |
| Number of species-specific orthogroups | 17,805 |
| Number of genes in species-specific orthogroups | 57,730 |
| Percentage of genes in species-specific orthogroups | 10.4 |
| Mean orthogroup size | 9.5 |
| Median orthogroup size | 4 |
| G50 (assigned genes) | 19 |
| G50 (all genes) | 15 |
| O50 (assigned genes) | 6,750 |
| O50 (all genes) | 103,08 |
| Number of orthogroups with all species present | 6,348 |
| Number of single-copy orthogroups | 278 |
