# Supplementary material for: Histology and transcriptomic analyses of barnacles with different base materials and habitats shed lights on the duplication and chemical diversification of barnacle cement proteins
Source: BMC Genomics. 2021 Nov 1;22:783. doi: 10.1186/s12864-021-08049-4 (PMC8561864; doi:10.1186/s12864-021-08049-4)
Supplement: Supplementary file 3 — Additional file 3 [file 12864_2021_8049_MOESM3_ESM.pdf]

Additional file 3. Summary of all CP homologs. (page 1)

Assigned name	Species	NCBI accession no.	Orthogroup	CP	Base	Substrate	Full_lengthpartial	GRAVY
Aamph_CP100k_homolog1_isoform1	Aamph	MW462722	OG0001681	100k	Calcareous	rock	partial	0.186778399
Aamph_CP100k_homolog1_isoform2	Aamph	MW462723	OG0001681	100k	Calcareous	rock	Full_length	0.178787879
Aamph_CP100k_homolog2	Aamph	MW462724	OG0001681	100k	Calcareous	rock	Full_length	0.1241
Aamph_CP100k_homolog3	Aamph	MW462717	OG0001681	100k	Calcareous	rock	partial	0.214450867
Aamph_CP100k_homolog4	Aamph	MW462715	OG0001681	100k	Calcareous	rock	partial	0.025423729
Aamph_CP100k_homolog5	Aamph	MW462718	OG0001681	100k	Calcareous	rock	partial	-0.081451613
Aamph_CP100k_homolog6	Aamph	MW462719	OG0001681	100k	Calcareous	rock	partial	-0.2025
Aamph_CP100k_homolog7	Aamph	MW462720	OG0001681	100k	Calcareous	rock	partial	-0.089285714
Aamph_CP100k_homolog8	Aamph	MW462721	OG0001681	100k	Calcareous	rock	partial	-0.284545455
Aamph_CP100k_homolog9	Aamph	MW462716	OG0001681	100k	Calcareous	rock	partial	0.211620795
Aamph_100k_AGS19349.1	Aamph	AGS19349.1		100k	Calcareous	rock	Full_length	0.178287197
Aamph_114k_AKZ20818.1	Aamph	AKZ20818.1		100k	Calcareous	rock	Full_length	0.135
Mrosa_100k_BAB12269.1	Mrosa	BAB12269.1		100k	Calcareous	rock	Full_length	-0.013293051
Chunt_CP100k_homolog1	Chunt	MW462695	OG0032961	100k	Membranous	Floating objects	partial	0.002145923
Chunt_CP100k_homolog2	Chunt	MW462696	OG0001681	100k	Membranous	Floating objects	partial	0.311881188
Chunt_CP100k_homolog3	Chunt	MW462697	OG0001681	100k	Membranous	Floating objects	partial	-0.063829787
Chunt_CP100k_homolog4	Chunt	MW462698	OG0001681	100k	Membranous	Floating objects	partial	0.207017544
Chunt_CP100k_homolog5	Chunt	MW462699	OG0001681	100k	Membranous	Floating objects	partial	0.134177215
Chunt_CP100k_homolog6	Chunt	MW462700	OG0034915	100k	Membranous	Floating objects	partial	0.053763441
Cmala_CP100k_homolog1	Cmala	MW462714	OG0001681	100k	Membranous	rock	Full_length	0.16030025
Cmite_CP100k_homolog1	Cmite	MW462704	OG0001681	100k	Membranous	rock	Full_length	0.224010327
Ctest_CP100k_homolog1_isoform_1	Ctest	MW462689	OG0001681	100k	Membranous	Turtles/crabs	Full_length	0.161989796
Ctest_CP100k_homolog1_isoform_2	Ctest	MW462690	OG0001681	100k	Membranous	Turtles/crabs	Full_length	0.165617021
Ctest_CP100k_homolog1_isoform_3	Ctest	MW462691	OG0001681	100k	Membranous	Turtles/crabs	partial	0.05255102
Ctest_CP100k_homolog2	Ctest	MW462688	OG0001681	100k	Membranous	Turtles/crabs	partial	0.184878049
Lanat_CP100k_homolog1	Lanat	MW462686	OG0034915	100k	Membranous	Floating objects	partial	0.226315789
Lanat_CP100k_homolog2	Lanat	MW462687	OG0032961	100k	Membranous	Floating objects	partial	0.057345972
Majax_CP100k_homolog1	Majax	MW462705	OG0001681	100k	Calcareous	Corals	Full_length	-0.011666667

Additional file 3. Summary of all CP homologs. (page 2)

Assigned name	pI	estimated Mw	transcript ID	Base_R1	Base_R2	soma_R1	soma_R2
Aamph_CP100k_homolog1_isoform1	9.837	61622.8657	Aamph_140108_CL2143.Contig1_Ba_mix	258.9825	NA	1.76269	NA
Aamph_CP100k_homolog1_isoform2	9.719	129598.0823	Aamph_140108_CL2143.Contig2_Ba_mix	311.281	NA	0.18628	NA
Aamph_CP100k_homolog2	9.443	114550.7733	Aamph_140108_CL3031.Contig1_Ba_mix	6316.361	NA	1.864716	NA
Aamph_CP100k_homolog3	9.77	96605.0665	Aamph_170312_TRINITY_DN119856_c0_g7_i2	13.91756	NA	0.120103	NA
Aamph_CP100k_homolog4	9.802	34215.2541	Aamph_170312_TRINITY_DN12011_c0_g1_i1	16.99424	NA	0	NA
Aamph_CP100k_homolog5	10.129	14174.5631	Aamph_170312_TRINITY_DN124352_c0_g1_i1	29.30488	NA	0	NA
Aamph_CP100k_homolog6	9.125	13822.9374	Aamph_170312_TRINITY_DN130017_c0_g1_i1	5.79208	NA	0	NA
Aamph_CP100k_homolog7	7.773	16128.7249	Aamph_170312_TRINITY_DN130724_c0_g1_i1	24.58072	NA	0	NA
Aamph_CP100k_homolog8	10.98	12786.0460	Aamph_170312_TRINITY_DN134975_c0_g1_i1	10.49296	NA	0	NA
Aamph_CP100k_homolog9	9.7	37326.9938	Aamph_170312_TRINITY_DN99279_c0_g1_i1	14.78316	NA	0.164666	NA
Aamph_100k_AGS19349.1	9.741	129477.8654	AGS19349	NA	NA	NA	NA
Aamph_114k_AKZ20818.1	9.483	114233.4133	AKZ20818	NA	NA	NA	NA
Mrosa_100k_BAB12269.1	9.413	113640.6968	BAB12269.1	NA	NA	NA	NA
Chunt_CP100k_homolog1	9.768	25820.6228	Chunt_TRINITY_DN139064_c0_g1_i1	0.917876	0.614368	0.1607	0.322699
Chunt_CP100k_homolog2	10.53	11175.4597	Chunt_TRINITY_DN173691_c0_g1_i1	1.59757	1.602928	0	0
Chunt_CP100k_homolog3	4.696	15691.7192	Chunt_TRINITY_DN249746_c0_g1_i1	1.722511	0	0	0
Chunt_CP100k_homolog4	6.59	12742.7668	Chunt_TRINITY_DN261218_c0_g1_i1	1.883122	0.630175	0	0
Chunt_CP100k_homolog5	9.319	17172.7882	Chunt_TRINITY_DN286513_c0_g1_i1	0.354575	0.355907	0.370711	0
Chunt_CP100k_homolog6	10.771	30043.4837	Chunt_TRINITY_DN302817_c0_g1_i1	0.152979	0.76796	0.3214	0.161349
Cmala_CP100k_homolog1	10.587	132967.6531	Cmala_TRINITY_DN14904_c0_g1_i1	185.23981	184.81695	0.479209	0.423828
Cmite_CP100k_homolog1	10.756	124390.5552	Cmite_TRINITY_DN22920_c0_g1_i3	91.512434	94.041425	0.026666	0
Ctest_CP100k_homolog1_isoform_1	10.658	130478.4600	Ctest_170615_TRINITY_DN109122_c0_g1_i4	8.958273	9.926426	0	0
Ctest_CP100k_homolog1_isoform_2	10.699	130006.5554	Ctest_170615_TRINITY_DN109122_c0_g1_i5	37.093795	38.329667	0	0
Ctest_CP100k_homolog1_isoform_3	11.835	44605.3645	Ctest_170615_TRINITY_DN109122_c0_g1_i6	51.163706	59.532229	0	0
Ctest_CP100k_homolog2	11.63	46024.9202	Ctest_170615_TRINITY_DN86401_c0_g1_i1	102.21711	105.91798	0.044645	0
Lanat_CP100k_homolog1	8.354	18649.4147	Lanti_TRINITY_DN115714_c0_g1_i1	0.568251	0.571356	0	0
Lanat_CP100k_homolog2	10.52	23057.4096	Lanti_TRINITY_DN53854_c0_g1_i1	0.820068	1.03044	0	0
Majax_CP100k_homolog1	9.511	115784.2530	Majex_TRINITY_DN14369_c0_g1_i1	324.66281	318.65213	1.327022	1.297987

Additional file 3. Summary of all CP homologs. (page 3)

Assigned name	Species	NCBI accession no.	Orthogroup	CP	Base	Substrate	Full_lengthpartial	GRAVY
Majax_CP100k_homolog2	Majax	MW462706	OG0001681	100k	Calcareous	Corals	partial	0.183666667
Majax_CP100k_homolog3	Majax	MW462707	OG0001681	100k	Calcareous	Corals	partial	0.004587156
Majax_CP100k_homolog4	Majax	MW462708	OG0001681	100k	Calcareous	Corals	partial	-0.009561753
Majax_CP100k_homolog5	Majax	MW462709	OG0001681	100k	Calcareous	Corals	partial	0.029957806
Mlong_CP100k_homolog1	Mlong	MW462692	OG0001681	100k	Membranous	Sponges	partial	0.125984252
Mlong_CP100k_homolog2	Mlong	MW462693	OG0001681	100k	Membranous	Sponges	partial	-0.092241379
Mlong_CP100k_homolog3	Mlong	MW462694	OG0001681	100k	Membranous	Sponges	partial	0.082198953
Tform_CP100k_homolog1	Tform	MW462710	OG0001681	100k	Calcareous	rock	Full_length	0.152364574
Wmill_CP100k_homolog1	Wmill	MW462701	OG0001681	100k	Calcareous	Corals	partial	0.086335404
Wmill_CP100k_homolog2	Wmill	MW462702	OG0001681	100k	Calcareous	Corals	partial	-0.108571429
Wmill_CP100k_homolog3	Wmill	MW462703	OG0001681	100k	Calcareous	Corals	partial	-0.042718447
Aamph_CP-19k-AKZ20819.1	Aamph	AKZ20819.1		19k	Calcareous	rock	Full_length	0.013300493
Aamph_CP-19k-2-AQA26371.1	Aamph	AQA26371.1		19k	Calcareous	rock	Full_length	-0.246506986
Aamph_CP19k_homolog3	Aamph	MW462848	OG0008889	19k	Calcareous	rock	partial	-0.747297297
Aamph_CP19k_homolog4	Aamph	MW462842	OG0008889	19k	Calcareous	rock	partial	-0.838053097
Aamph_CP-19k-3-AQA26372.1	Aamph	AQA26372.1		19k	Calcareous	rock	Full_length	-0.387280702
Aamph_CP-19k-4-AQA26373.1	Aamph	AQA26373.1		19k	Calcareous	rock	Full_length	-0.482938389
Aamph_CP19k_homolog6	Aamph	MW462845	OG0008889	19k	Calcareous	rock	partial	-0.270833333
Aamph_CP19k_homolog7	Aamph	MW462846	OG0008889	19k	Calcareous	rock	partial	-1.646017699
Aamph_CP19k_homolog8	Aamph	MW462847	OG0008889	19k	Calcareous	rock	partial	-0.6
Aamph_CP19k_homolog9	Aamph	MW462841	OG0008889	19k	Calcareous	rock	partial	-0.608465608
Aamph_CP-19k-5-AQA26376.1	Aamph	AQA26376.1		19k	Calcareous	rock	Full_length	-0.515322581
Mrosa_CP-19k-BAE94409.1	Mrosa	BAE94409.1		19k	Calcareous	rock	Full_length	-0.108080808
Ppoll_CP19k-ATB53755.1	Pollicipes pollicipes	ATB53755.1		19k	Membranous	rock	Full_length	
Mvolc_CP19k-QDO67070.1	Megabalanus volcano	QDO67070.1		19k	Calcareous	rock	Full_length	
Falbi_CP19k_BAE94410.1	Fistulobalanus albicostatus	BAE94410.1		19k	Calcareous	rock	Full_length	

Additional file 3. Summary of all CP homologs. (page 4)

Assigned name	pI	estimated Mw	transcript ID	Base_R1	Base_R2	soma_R1	soma_R2
Majax_CP100k_homolog2	10.258	34121.7117	Majex_TRINITY_DN38903_c0_g1_i1	3.210984	2.276104	0.110505	0
Majax_CP100k_homolog3	9.509	24572.4559	Majex_TRINITY_DN464_c0_g1_i1	1.496419	1.497268	0	0
Majax_CP100k_homolog4	9.979	28739.3514	Majex_TRINITY_DN5038_c0_g1_i1	1.645289	2.263717	0	0
Majax_CP100k_homolog5	9.676	26399.5720	Majex_TRINITY_DN524_c0_g1_i1	2.003636	1.336556	0	0
Mlong_CP100k_homolog1	10.976	13616.5285	Mlong_TRINITY_DN37885_c0_g1_i1	0	0	1.01778	0
Mlong_CP100k_homolog2	9.246	12899.9067	Mlong_TRINITY_DN56399_c0_g1_i1	0	0	3.601815	0
Mlong_CP100k_homolog3	9.716	20976.9871	Mlong_TRINITY_DN79458_c0_g1_i1	0	0.262566	1.309984	1.316189
Tform_CP100k_homolog1	10.491	130793.7580	TF_160309_TR54333|c0_g1_i1	1524.1987	NA	0	NA
Wmill_CP100k_homolog1	8.366	18563.5979	Wmill_TRINITY_DN41843_c0_g1_i1	0.39848	0.399786	0	0.469793
Wmill_CP100k_homolog2	10.382	12626.1285	Wmill_TRINITY_DN86332_c0_g1_i1	1.664322	1.667399	0	0
Wmill_CP100k_homolog3	9.612	11507.4890	Wmill_TRINITY_DN88483_c0_g1_i1	0.86314	0.864725	0	0
Aamph_CP-19k-AKZ20819.1	10.111	20166.3094	AKZ20819		NA	NA	NA
Aamph_CP-19k-2-AQA26371.1	10.224	47614.1873	AQA26371		NA	NA	NA
Aamph_CP19k_homolog3	11.2	14881.0680	Aamph_140108_Unigene15903_Ba_mix	26.2324	NA	0	NA
Aamph_CP19k_homolog4	9.038	23249.5429	Aamph_170312_TRINITY_DN102386_c0_g1_i3	184.8325	NA	0.058346	NA
Aamph_CP-19k-3-AQA26372.1	9.112	45314.2236	AQA26372	NA	NA	NA	NA
Aamph_CP-19k-4-AQA26373.1	9.788	42351.4690	AQA26373	NA	NA	NA	NA
Aamph_CP19k_homolog6	9.985	16459.2246	Aamph_170312_TRINITY_DN117451_c0_g11_i4	42.4856	NA	0.209345	NA
Aamph_CP19k_homolog7	4.705	12331.6066	Aamph_170312_TRINITY_DN117451_c0_g14_i1	73.2622	NA	0.362033	NA
Aamph_CP19k_homolog8	9.944	20125.0059	Aamph_170312_TRINITY_DN117451_c0_g15_i6	14.4802	NA	0	NA
Aamph_CP19k_homolog9	9.913	18675.1196	Aamph_170312_TRINITY_DN96062_c0_g1_i1	61.4518	NA	0	NA
Aamph_CP-19k-5-AQA26376.1	10.651	38252.7604	AQA26376	NA	NA	NA	NA
Mrosa_CP-19k-BAE94409.1	5.33	19516.0000	BAE94409.1	NA	NA	NA	NA
Ppoll_CP19k-ATB53755.1			ATB53755.1	NA	NA	NA	NA
Mvolc_CP19k-QDO67070.1			QDO67070.1	NA	NA	NA	NA
Falbi_CP19k_BAE94410.1			BAE94410.1	NA	NA	NA	NA

Additional file 3. Summary of all CP homologs. (page 5)

Assigned name	Species	NCBI accession no.	Orthogroup	CP	Base	Substrate	Full_lengthpartial	GRAVY
Cmala_CP19k_homolog1	Cmala	MW462829	OG0008889	19k	Membranous	rock	Full_length	-0.426933333
Cmala_CP19k_homolog3	Cmala	MW462831	OG0008889	19k	Membranous	rock	Full_length	-0.672928177
Cmala_CP19k_homolog5	Cmala	MW462834	OG0008889	19k	Membranous	rock	Full_length	-0.149234694
Aamph_CP-19k-6-AQA26378.1	Aamph	AQA26378.1	OG0008889	19k	Calcareous	rock	partial	-0.585526316
Aamph_CP-19k-7-AQA26379.1	Aamph	AQA26379.1	OG0008889	19k	Calcareous	rock	partial	-0.445945946
Cmala_CP19k_homolog9	Cmala	MW462838	OG0008889	19k	Membranous	rock	Full_length	0.06
Ctest_CP19k_homolog3	Ctest	MW462820	OG0008889	19k	Membranous	Turtles/crabs	Full_length	-0.293095768
Majax_CP19k_homolog2	Majax	MW462823	OG0008889	19k	Calcareous	Corals	Full_length	-0.395137421
Tform_CP19k_homolog3	Tform	MW462826	OG0008889	19k	Calcareous	rock	Full_length	-0.415250965
Aamph_CP19k_homolog2	Aamph	MW462850	OG0016354	19k	Calcareous	rock	Full_length	0.031034483
Aamph_CP19k_homolog5_isoform_1	Aamph	MW462843	OG0016354	19k	Calcareous	rock	Full_length	-0.478277154
Aamph_CP19k_homolog5_isoform_2	Aamph	MW462844	OG0016354	19k	Calcareous	rock	Full_length	-0.435907336
Cmala_CP19k_homolog6	Cmala	MW462835	OG0008889	19k	Membranous	rock	partial	-0.246494465
Cmala_CP19k_homolog7	Cmala	MW462836	OG0008889	19k	Membranous	rock	partial	-0.747731397
Cmala_CP19k_homolog8	Cmala	MW462837	OG0008889	19k	Membranous	rock	partial	-0.292207792
Ctest_CP19k_homolog1	Ctest	MW462818	OG0016354	19k	Membranous	Turtles/crabs	Full_length	0.030526316
Cmala_CP19k_homolog10	Cmala	MW462839	OG0019175	19k	Membranous	rock	partial	-1.031067961
Cmala_CP19k_homolog11	Cmala	MW462840	OG0020443	19k	Membranous	rock	partial	-0.461904762
Tform_CP19k_homolog1	Tform	MW462824	OG0016354	19k	Calcareous	rock	Full_length	-0.219583333
Tform_CP19k_homolog2	Tform	MW462825	OG0016354	19k	Calcareous	rock	Full_length	-0.195338983
Ctest_CP19k_homolog2	Ctest	MW462819	OG0019175	19k	Membranous	Turtles/crabs	partial	-0.194623656
Tform_CP19k_homolog5	Tform	MW462828	OG0016354	19k	Calcareous	rock	Full_length	-0.2152
Aamph_CP19k_homolog1	Aamph	MW462849	OG0019175	19k	Calcareous	rock	Full_length	-0.409247312
Majax_CP19k_homolog1	Majax	MW462822	OG0019175	19k	Calcareous	Corals	Full_length	-0.317371938
Tform_CP19k_homolog4	Tform	MW462827	OG0019175	19k	Calcareous	rock	Full_length	-0.1569869
Cmala_CP19k_homolog2	Cmala	MW462830	OG0020443	19k	Membranous	rock	Full_length	0.045177665
Cmala_CP19k_homolog4_isoform_1	Cmala	MW462832	OG0020443	19k	Membranous	rock	Full_length	-0.234883721

Additional file 3. Summary of all CP homologs. (page 6)

Assigned name	pI	estimated Mw	transcript ID	Base_R1	Base_R2	soma_R1	soma_R2
Cmala_CP19k_homolog1	11.39	37373.0345	Cmala_TRINITY_DN12977_c0_g1_i1	80.862944	78.344133	0.139626	0.069982
Cmala_CP19k_homolog3	10.529	54291.9773	Cmala_TRINITY_DN16562_c0_g1_i1	146.49349	150.38427	0.061789	0.123876
Cmala_CP19k_homolog5	10.443	38154.5930	Cmala_TRINITY_DN19565_c0_g1_i1	78.856795	75.751441	0.220362	0.294529
Aamph_CP-19k-6-AQA26378.1	10.028	7813.4289	AQA26378	NA	NA	NA	NA
Aamph_CP-19k-7-AQA26379.1	8.525	10718.4725	AQA26379	NA	NA	NA	NA
Cmala_CP19k_homolog9	12.43	19171.8641	Cmala_TRINITY_DN52758_c0_g1_i1	2.533418	2.534406	0	0
Ctest_CP19k_homolog3	10.196	44747.0867	Ctest_170615_TRINITY_DN96511_c0_g1_i1	8.134553	8.543407	0	0
Majax_CP19k_homolog2	9.81	47837.9398	Majex_TRINITY_DN56716_c0_g1_i1	11.926699	11.823429	0	0
Tform_CP19k_homolog3	10.475	51945.9081	TF_160309_TR63530|c0_g1_i1	10.18752	NA	0	NA
Aamph_CP19k_homolog2	9.969	20152.2361	Aamph_140108_CL9507.Contig1_Ba_mix	436.6977	NA	1.928043	NA
Aamph_CP19k_homolog5_isoform_1	8.518	27035.8779	Aamph_170312_TRINITY_DN110324_c0_g1_i4	100.3071	NA	0	NA
Aamph_CP19k_homolog5_isoform_2	9.35	26187.9547	Aamph_170312_TRINITY_DN110324_c0_g1_i8	62.2018	NA	0.059016	NA
Cmala_CP19k_homolog6	11.364	26338.1891	Cmala_TRINITY_DN23239_c0_g1_i1	9.858707	10.834553	0.659299	0.13219
Cmala_CP19k_homolog7	10.741	55454.0028	Cmala_TRINITY_DN23239_c1_g1_i1	10.221543	9.21525	0	0.2876
Cmala_CP19k_homolog8	10.182	15349.1323	Cmala_TRINITY_DN34884_c0_g1_i1	0.45386	1.816261	0	0.372505
Ctest_CP19k_homolog1	9.439	27645.9753	Ctest_170615_TRINITY_DN86728_c0_g1_i1	1.351408	1.441685	0.333108	0.133316
Cmala_CP19k_homolog10	9.595	10191.7915	Cmala_TRINITY_DN60481_c0_g1_i1	0.792482	1.584729	1.298913	0
Cmala_CP19k_homolog11	10.881	20892.1545	Cmala_TRINITY_DN61689_c0_g1_i1	0.739983	0	0.603598	0.605103
Tform_CP19k_homolog1	10.806	23943.4866	TF_160309_TR57313|c0_g1_i1	202.92416	NA	0	NA
Tform_CP19k_homolog2	10.358	23547.0111	TF_160309_TR63517|c0_g1_i1	217.57175	NA	0	NA
Ctest_CP19k_homolog2	10.518	45263.3960	Ctest_170615_TRINITY_DN87774_c0_g1_i1	9.236846	8.748168	0	0
Tform_CP19k_homolog5	10.452	25373.9929	TF_160309_TR71863|c0_g1_i1	12.212923	NA	0	NA
Aamph_CP19k_homolog1	9.365	46262.3135	Aamph_140108_CL14151.Contig1_Ba_mix	79.525	NA	0.102128	NA
Majax_CP19k_homolog1	9.065	45030.1729	Majex_TRINITY_DN16151_c1_g1_i1	8.193488	9.006183	0	0
Tform_CP19k_homolog4	10.379	45068.5550	TF_160309_TR66686|c0_g1_i1	15.444005	NA	0	NA
Cmala_CP19k_homolog2	10.775	19192.0051	Cmala_TRINITY_DN13810_c0_g1_i1	11.243317	9.885822	0	0
Cmala_CP19k_homolog4_isoform_1	11.17	21140.9611	Cmala_TRINITY_DN19296_c0_g1_i2	8.362521	6.237293	0	0

Additional file 3. Summary of all CP homologs. (page 7)

Assigned name	Species	NCBI accession no.	Orthogroup	CP	Base	Substrate	Full_lengthpartial	GRAVY
Cmala_CP19k_homolog4_isoform_2	Cmala	MW462833	OG0020443	19k	Membranous	rock	Full_length	-0.177927928
Cmite_CP19k_homolog1	Cmite	MW462821	OG0020443	19k	Membranous	rock	Full_length	-0.146086957
Falbi_CP20k_BAF96022.1	Fistulobalanus albicostatus	BAF96022.1		20k	Calcareous	rock	Full_length	
Aamph_CP20k_homolog1	Aamph	MW462864	OG0024973	20k	Calcareous	rock	Full_length	-0.274418605
Aamph_CP20k_homolog2	Aamph	MW462862	OG0019302	20k	Calcareous	rock	Full_length	-1.011029412
Aamph_CP20k_homolog3	Aamph	MW462863	OG0019302	20k	Calcareous	rock	Full_length	-0.979389313
Aamph_CP20k_homolog4	Aamph	MW462859	OG0019302	20k	Calcareous	rock	Full_length	-0.867669173
Aamph_CP20k_homolog5	Aamph	MW462860	OG0019302	20k	Calcareous	rock	Full_length	-0.969465649
Aamph_CP20k_homolog6	Aamph	MW462861	OG0024973	20k	Calcareous	rock	Full_length	-0.42311828
Aamph_CP20k_homolog7	Aamph	MW462858	OG0019302	20k	Calcareous	rock	Full_length	-0.760909091
Aamph_CP20k_homolog8	Aamph	MW462857	OG0024973	20k	Calcareous	rock	Full_length	-0.275423729
Mrosa_CP-20k-BBAB18762.1	Mrosa	BBAB18762.1		20k	Calcareous	rock	Full_length	-0.804455446
Chunt_CP20k_homolog1	Chunt	MW462851	OG0035244	20k	Membranous	Floating objects	Full_length	-0.800806452
Cmala_CP20k_homolog1	Cmala	MW462856	OG0035244	20k	Membranous	rock	Full_length	-0.821774194
Cmite_CP20k_homolog1_isoform_1	Cmite	MW462852	OG0035244	20k	Membranous	rock	Full_length	-0.7486
Cmite_CP20k_homolog1_isoform_2	Cmite	MW462853	OG0035244	20k	Membranous	rock	Full_length	-0.746017699
Cmite_CP20k_homolog1_isoform_3	Cmite	MW462854	OG0035244	20k	Membranous	rock	Full_length	-0.746017699
Cmite_CP20k_homolog2	Cmite	MW462855	OG0035244	20k	Membranous	rock	Full_length	-0.23889
Aamph_CP43k_homolog1	Aamph	MW462751	OG0015331	43k	Calcareous	rock	Full_length	-0.333854167
Aamph_CP43k_homolog2	Aamph	MW462752	OG0015331	43k	Calcareous	rock	Full_length	-0.334015345
Aamph_CP43k_homolog3	Aamph	MW462750	OG0013232	43k	Calcareous	rock	partial	-0.705825243
Aamph_CP43k_homolog4	Aamph	MW462746	OG0016429	43k	Calcareous	rock	partial	-0.35915493
Aamph_CP43k_homolog5	Aamph	MW462747	OG0016429	43k	Calcareous	rock	partial	-0.469047619
Aamph_CP43k_homolog6_isoform_1	Aamph	MW462748	OG0015331	43k	Calcareous	rock	partial	0.101785714
Aamph_CP43k_homolog6_isoform_2	Aamph	MW462749	OG0015331	43k	Calcareous	rock	partial	0.108571429
Aamph_CP43k_homolog7	Aamph	MW462743	OG0032098	43k	Calcareous	rock	partial	-0.194339623

Additional file 3. Summary of all CP homologs. (page 8)

Assigned name	pI	estimated Mw	transcript ID	Base_R1	Base_R2	soma_R1	soma_R2
Cmala_CP19k_homolog4_isoform_2	10.825	21969.9154	Cmala_TRINITY_DN19296_c0_g1_i4	12.937772	12.576289	0	0.16291
Cmite_CP19k_homolog1	10.759	22387.0459	Cmite_TRINITY_DN44054_c0_g1_i1	42.512098	41.967864	0.157025	0.078767
Falbi_CP20k_BAF96022.1			BAF96022.1	NA	NA	NA	NA
Aamph_CP20k_homolog1	5.929	14375.2554	Aamph_140108_CL917.Contig1_Ba_mix	67.2732	NA	0.041378	NA
Aamph_CP20k_homolog2	7.776	16035.2806	Aamph_140108_Unigene33081_Ba_mix	1254.4271	NA	0	NA
Aamph_CP20k_homolog3	7.651	15395.5684	Aamph_140108_Unigene33082_Ba_mix	434.9338	NA	15.051436	NA
Aamph_CP20k_homolog4	7.072	15367.3693	Aamph_140108_Unigene4944_Ba_mix	26.2324	NA	0	NA
Aamph_CP20k_homolog5	7.646	15332.5067	Aamph_140108_Unigene4945_Ba_mix	184.8325	NA	1.372366	NA
Aamph_CP20k_homolog6	3.932	13094.5606	Aamph_140108_Unigene5084_Ba_mix	100.3071	NA	0	NA
Aamph_CP20k_homolog7	7.002	12446.0499	Aamph_170312_TRINITY_DN101384_c1_g2_i1	62.2018	NA	0.153968	NA
Aamph_CP20k_homolog8	4.08	13083.6270	Aamph_170312_TRINITY_DN92857_c1_g1_i2	22.9096	NA	0.040726	NA
Mrosa_CP-20k-BBAB18762.1	4.76	22466.0000	BBAB18762	NA	NA	NA	NA
Chunt_CP20k_homolog1	8.347	14272.7018	Chunt_TRINITY_DN34424_c0_g1_i1	2.126876	2.348988	0.445921	0
Cmala_CP20k_homolog1	8.347	14242.6143	Cmala_TRINITY_DN13935_c0_g1_i1	2986.3189	2940.0957	2.105288	1.535172
Cmite_CP20k_homolog1_isoform_1	6.527	13040.8832	Cmite_TRINITY_DN20195_c0_g1_i1	8.475655	7.454707	33.27796	29.92353
Cmite_CP20k_homolog1_isoform_2	6.527	13040.8832	Cmite_TRINITY_DN20195_c0_g1_i2	12.73147	12.940874	20.653553	21.454052
Cmite_CP20k_homolog1_isoform_3	6.49	13017.8500	Cmite_TRINITY_DN20195_c0_g1_i3	11.72539	11.259887	11.33727	9.693778
Cmite_CP20k_homolog2	7.09	12004.8900	Cmite_TRINITY_DN32162_c0_g1_i1	446.76404	453.98749	0.516906	0.518628
Aamph_CP43k_homolog1	5.492	36138.8512	Aamph_140108_CL217.Contig2_Ba_mix	65.2	NA	1.694899	NA
Aamph_CP43k_homolog2	5.492	36808.6729	Aamph_140108_CL217.Contig4_Ba_mix	704.8715	NA	1.316715	NA
Aamph_CP43k_homolog3	10.5	20555.3472	Aamph_140108_Unigene11709_Ba_mix	30.3851	NA	0	NA
Aamph_CP43k_homolog4	10.531	20919.8431	Aamph_170312_TRINITY_DN102661_c1_g6_i1	10.6104	NA	0	NA
Aamph_CP43k_homolog5	6.894	12045.7988	Aamph_170312_TRINITY_DN106445_c0_g2_i1	42.4856	NA	0	NA
Aamph_CP43k_homolog6_isoform_1	9.878	10751.3940	Aamph_170312_TRINITY_DN116225_c2_g3_i1	73.2622	NA	0	NA
Aamph_CP43k_homolog6_isoform_2	9.878	10053.5185	Aamph_170312_TRINITY_DN116225_c2_g3_i3	14.4802	NA	0.237331	NA
Aamph_CP43k_homolog7	10.268	10222.2747	Aamph_170312_TRINITY_DN5689_c0_g1_i1	61.4518	NA	0	NA

Additional file 3. Summary of all CP homologs. (page 9)

Assigned name	Species	NCBI accession no.	Orthogroup	CP	Base	Substrate	Full_lengthpartial	GRAVY
Aamph_CP43k_homolog8_isoform_1	Aamph	MW462744	OG0015331	43k	Calcareous	rock	Full_length	-0.481127983
Aamph_CP43k_homolog8_isoform_2	Aamph	MW462745	OG0015331	43k	Calcareous	rock	partial	-0.324867725
AQA26370.1_CP-43k-1-AQA26370.1	Aamph	AQA26370.1	OG0015331	43k	Calcareous	rock	partial	-0.435940803
AQA26374.1_CP-43k-2-AQA26374.1	Aamph	AQA26374.1	OG0015331	43k	Calcareous	rock	partial	-0.46971831
AQA26377.1_CP-43k-3-AQA26377.1	Aamph	AQA26377.1	OG0015331	43k	Calcareous	rock	partial	-0.174537037
Chunt_CP43k_homolog1	Chunt	MW462734	OG0013232	43k	Membranous	Floating objects	partial	-0.393193717
Chunt_CP43k_homolog2	Chunt	MW462735	OG0016429	43k	Membranous	Floating objects	partial	-0.675
Chunt_CP43k_homolog3	Chunt	MW462736	OG0016429	43k	Membranous	Floating objects	partial	-0.575862069
Cmala_CP43k_homolog1	Cmala	MW462742	OG0016429	43k	Membranous	rock	Full_length	-0.448994975
Cmite_CP43k_homolog1	Cmite	MW462737	OG0013232	43k	Membranous	rock	Full_length	-0.296636771
Ctest_CP43k_homolog1	Ctest	MW462733	OG0015331	43k	Membranous	Turtles/crabs	partial	-0.575107296
Ctest_CP43k_homolog2_isoform_1	Ctest	MW462727	OG0013232	43k	Membranous	Turtles/crabs	Full_length	-0.373166927
Ctest_CP43k_homolog2_isoform_2	Ctest	MW462728	OG0013232	43k	Membranous	Turtles/crabs	Full_length	-0.350535988
Ctest_CP43k_homolog2_isoform_3	Ctest	MW462729	OG0013232	43k	Membranous	Turtles/crabs	partial	-0.517467949
Ctest_CP43k_homolog2_isoform_4	Ctest	MW462730	OG0013232	43k	Membranous	Turtles/crabs	Full_length	-0.429923664
Ctest_CP43k_homolog3_isoform_1	Ctest	MW462731	OG0016429	43k	Membranous	Turtles/crabs	partial	-0.612962963
Ctest_CP43k_homolog3_isoform_2	Ctest	MW462732	OG0016429	43k	Membranous	Turtles/crabs	partial	-0.636382536
Lanat_CP43k_homolog1	Lanat	MW462726	OG0013232	43k	Membranous	Floating objects	Full_length	-0.322384937
Majax_CP43k_homolog1	Majax	MW462738	OG0032098	43k	Calcareous	Corals	Full_length	-0.441685649
Majax_CP43k_homolog2	Majax	MW462739	OG0012121	43k	Calcareous	Corals	Full_length	-0.407564576
PECT_CP43k_homolog1	PECT	MW462725	OG0013232	43k	Calcareous	Sponges	partial	-0.362411348
Tform_CP43k_homolog1	Tform	MW462740	OG0015331	43k	Calcareous	rock	Full_length	-0.495555556

Additional file 3. Summary of all CP homologs. (page 10)

Assigned name	pI	estimated Mw	transcript ID	Base_R1	Base_R2	soma_R1	soma_R2
Aamph_CP43k_homolog8_isoform_1	6.249	46303.9488	Aamph_170312_TRINITY_DN73624_c0_g1_i1	26.2324	NA	0	NA
Aamph_CP43k_homolog8_isoform_2	7.945	19098.4179	Aamph_170312_TRINITY_DN73624_c0_g1_i3	36.9579	NA	0	NA
AQA26370.1_CP-43k-1-AQA26370.1	9.537	45948.1570	AQA26370	NA	NA	NA	NA
AQA26374.1_CP-43k-2-AQA26374.1	6.412	28199.8304	AQA26374	NA	NA	NA	NA
AQA26377.1_CP-43k-3-AQA26377.1	6.924	20690.6797	AQA26377	NA	NA	NA	NA
Chunt_CP43k_homolog1	3.974	19141.4915	Chunt_TRINITY_DN14078_c0_g2_i1	0.525262	0.351572	0.918868	1.400273
Chunt_CP43k_homolog2	10.605	13167.3228	Chunt_TRINITY_DN221884_c0_g1_i1	0.909913	0.913555	0.951248	0
Chunt_CP43k_homolog3	9.164	10811.5346	Chunt_TRINITY_DN74589_c0_g1_i1	2.483551	1.246549	0	0.651509
Cmala_CP43k_homolog1	10.218	37981.4993	Cmala_TRINITY_DN17572_c0_g1_i1	647.00258	632.62595	1.290451	1.724804
Cmite_CP43k_homolog1	10.126	42300.4482	Cmite_TRINITY_DN21715_c0_g1_i1	1587.2917	1593.5089	3.095425	2.883612
Ctest_CP43k_homolog1	9.567	45021.8189	Ctest_170615_TRINITY_DN101681_c1_g1_i2	211.07971	217.08541	0	0.045599
Ctest_CP43k_homolog2_isoform_1	10.057	63155.3474	Ctest_170615_TRINITY_DN97391_c0_g1_i3	22.992403	23.571822	0	0
Ctest_CP43k_homolog2_isoform_2	10.057	64267.6848	Ctest_170615_TRINITY_DN97391_c0_g1_i4	19.141563	18.250644	0	0
Ctest_CP43k_homolog2_isoform_3	9.604	63226.9191	Ctest_170615_TRINITY_DN97391_c0_g1_i6	3.082854	2.57027	0.037458	0
Ctest_CP43k_homolog2_isoform_4	9.973	64692.7013	Ctest_170615_TRINITY_DN97391_c0_g1_i7	2.245011	0.582479	0	0
Ctest_CP43k_homolog3_isoform_1	9.975	27105.3774	Ctest_170615_TRINITY_DN97456_c0_g1_i1	1.024502	0	0	0
Ctest_CP43k_homolog3_isoform_2	10.578	47582.7443	Ctest_170615_TRINITY_DN97456_c0_g1_i2	10.041454	8.600118	0	0.045537
Lanat_CP43k_homolog1	4.302	46377.9310	Lanti_TRINITY_DN84167_c0_g1_i1	3.934922	5.36151	0	0
Majax_CP43k_homolog1	8.076	42085.2604	Majex_TRINITY_DN11586_c0_g1_i1	695.05269	705.26123	0.740159	0.740408
Majax_CP43k_homolog2	6.469	54358.7404	Majex_TRINITY_DN22249_c0_g1_i3	0.265468	0	0	1.070742
PECT_CP43k_homolog1	9.558	13217.0625	PECT_TRINITY_DN74810_c0_g1_i1	0	0.413007	1.241185	0.414222
Tform_CP43k_homolog1	10.267	43541.0646	TF_160309_TR52047|c0_g1_i1	3599.5395	NA	0	NA

Additional file 3. Summary of all CP homologs. (page 11)

Assigned name	Species	NCBI accession no.	Orthogroup	CP	Base	Substrate	Full_lengthpartial	GRAVY
Tform_CP43k_homolog2	Tform	MW462741	OG0013232	43k	Calcareous	rock	partial	-0.492899408
Aamph_CP52k_homolog1	Aamph	MW462816	OG0007385	52k	Calcareous	rock	partial	0.094571429
Aamph_CP52k_homolog2	Aamph	MW462817	OG0007385	52k	Calcareous	rock	Full_length	-0.033560709
Aamph_CP52k_homolog3	Aamph	AKZ20820.1		52k	Calcareous	rock	Full_length	-0.087281399
Mrosa_CP52k_homolog4	Mrosa	BAL22342.1		52k	Calcareous	rock	Full_length	0.005496454
Ppoll_CP52k-ATB53756.1	Pollicipes pollicipes	ATB53756.1		52k	Membranous	rock	Full_length	
Mvolc_CP52k-QDO67067.1	Megabalanus volcano	QDO67067.1		52k	Calcareous	rock	Full_length	
Chunt_CP52k_homolog1	Chunt	MW462801	OG0003309	52k	Membranous	Floating objects	Full_length	0.207258065
Cmala_CP52k_homolog1	Cmala	MW462813	OG0003309	52k	Membranous	rock	Full_length	-0.263476071
Cmala_CP52k_homolog2	Cmala	MW462814	OG0003309	52k	Membranous	rock	Full_length	-0.383254344
Cmala_CP52k_homolog3	Cmala	MW462815	OG0003309	52k	Membranous	rock	partial	0.410759494
Cmite_CP52k_homolog1	Cmite	MW462803	OG0003309	52k	Membranous	rock	Full_length	0.557004831
Cmite_CP52k_homolog2	Cmite	MW462804	OG0003309	52k	Membranous	rock	Full_length	0.108022923
Cmite_CP52k_homolog3	Cmite	MW462805	OG0003309	52k	Membranous	rock	partial	0.369732938
Cmite_CP52k_homolog4	Cmite	MW462806	OG0003309	52k	Membranous	rock	partial	0.79
Cmite_CP52k_homolog5	Cmite	MW462807	OG0003309	52k	Membranous	rock	partial	0.527
Ctest_CP52k_homolog1	Ctest	MW462777	OG0003309	52k	Membranous	Turtles/crabs	partial	-0.117647059
Ctest_CP52k_homolog2	Ctest	MW462754	OG0003309	52k	Membranous	Turtles/crabs	partial	-0.060194175
Ctest_CP52k_homolog3	Ctest	MW462755	OG0003309	52k	Membranous	Turtles/crabs	partial	-0.283236994
Ctest_CP52k_homolog4	Ctest	MW462756	OG0003309	52k	Membranous	Turtles/crabs	partial	-0.066666667
Ctest_CP52k_homolog5	Ctest	MW462757	OG0003309	52k	Membranous	Turtles/crabs	partial	0.026857143
Ctest_CP52k_homolog6	Ctest	MW462758	OG0003309	52k	Membranous	Turtles/crabs	partial	-0.261290323
Ctest_CP52k_homolog7	Ctest	MW462759	OG0003309	52k	Membranous	Turtles/crabs	partial	-0.024180328
Ctest_CP52k_homolog8	Ctest	MW462760	OG0003309	52k	Membranous	Turtles/crabs	partial	0.076377953
Ctest_CP52k_homolog9	Ctest	MW462761	OG0003309	52k	Membranous	Turtles/crabs	partial	-0.03968254
Ctest_CP52k_homolog10_isoform_1	Ctest	MW462762	OG0003309	52k	Membranous	Turtles/crabs	partial	-0.445251397
Ctest_CP52k_homolog10_isoform_2	Ctest	MW462763	OG0003309	52k	Membranous	Turtles/crabs	Full_length	-0.178988327
Ctest_CP52k_homolog10_isoform_3	Ctest	MW462764	OG0003309	52k	Membranous	Turtles/crabs	partial	-0.136946903

Additional file 3. Summary of all CP homologs. (page 12)

Assigned name	pI	estimated Mw	transcript ID	Base_R1	Base_R2	soma_R1	soma_R2
Tform_CP43k_homolog2	10.267	49555.6826	TF_160309_TR64719|c0_g1_i1	201.46619	NA	0	NA
Aamph_CP52k_homolog1	10.919	40828.8161	Aamph_170312_TRINITY_DN106899_c0_g1_i3	73.2622	NA	0	NA
Aamph_CP52k_homolog2	10.751	84267.9429	Aamph_170312_TRINITY_DN106899_c1_g1_i1	24.2185	NA	0.257487	NA
Aamph_CP52k_homolog3	10.788	73015.7166	AKZ20820	#N/A	NA	#N/A	NA
Mrosa_CP52k_homolog4	10.494	64132.7221	BAL22342.1	NA	NA	NA	NA
Ppoll_CP52k-ATB53756.1			ATB53756.1	NA	NA	NA	NA
Mvolc_CP52k-QDO67067.1			QDO67067.1	NA	NA	NA	NA
Chunt_CP52k_homolog1	9.301	41095.1101	Chunt_TRINITY_DN14710_c0_g1_i1	3.081727	2.812818	0.885624	0.592982
Cmala_CP52k_homolog1	11.537	47389.4797	Cmala_TRINITY_DN16752_c0_g1_i1	174.88232	181.94245	0.269692	0.630821
Cmala_CP52k_homolog2	11.048	73569.5496	Cmala_TRINITY_DN24475_c0_g1_i1	154.52834	157.37918	0.348719	0.624373
Cmala_CP52k_homolog3	12.204	35317.1276	Cmala_TRINITY_DN9305_c0_g1_i1	10.404582	10.92377	0.442924	0.063427
Cmite_CP52k_homolog1	10.684	43236.7663	Cmite_TRINITY_DN12453_c0_g1_i3	7.858084	6.21726	0	0
Cmite_CP52k_homolog2	11.365	39514.3386	Cmite_TRINITY_DN14519_c0_g1_i2	5.951893	5.635441	0	0.08895
Cmite_CP52k_homolog3	11.56	72893.7284	Cmite_TRINITY_DN21231_c0_g1_i2	23.920135	25.507866	0	0
Cmite_CP52k_homolog4	11.619	20072.6720	Cmite_TRINITY_DN21231_c1_g1_i1	5.844905	3.462679	0	0
Cmite_CP52k_homolog5	11.573	21682.8250	Cmite_TRINITY_DN67826_c0_g1_i1	2.38544	1.86011	0.221202	0
Ctest_CP52k_homolog1	9.114	17819.7751	Ctest_170615_TRINITY_DN194197_c0_g1_i1	0.991934	0.995454	0	0
Ctest_CP52k_homolog2	9.499	11476.3746	Ctest_170615_TRINITY_DN33210_c0_g1_i1	1.396868	2.101932	0	0
Ctest_CP52k_homolog3	11.506	19658.0534	Ctest_170615_TRINITY_DN56201_c0_g2_i1	1.610999	1.078006	0.206579	0
Ctest_CP52k_homolog4	10.6	21140.0867	Ctest_170615_TRINITY_DN82489_c0_g1_i2	1.675609	1.201494	0.184825	0
Ctest_CP52k_homolog5	10.916	20204.9004	Ctest_170615_TRINITY_DN87731_c0_g1_i1	1.586336	2.388391	0	0
Ctest_CP52k_homolog6	7.125	31478.4527	Ctest_170615_TRINITY_DN87731_c0_g3_i2	1.491857	1.604848	0	0
Ctest_CP52k_homolog7	9.795	28169.0607	Ctest_170615_TRINITY_DN94376_c0_g2_i1	0.785935	0.428563	0	0
Ctest_CP52k_homolog8	9.114	14439.7486	Ctest_170615_TRINITY_DN94376_c0_g6_i1	1.386878	0.927452	0	0.349814
Ctest_CP52k_homolog9	9.637	21659.6188	Ctest_170615_TRINITY_DN94376_c0_g8_i1	1.297175	1.277417	0	0
Ctest_CP52k_homolog10_isoform_1	7.533	19260.6155	Ctest_170615_TRINITY_DN94376_c0_g9_i1	0.859039	1.660979	0.122185	0.122252
Ctest_CP52k_homolog10_isoform_2	9.927	28537.8813	Ctest_170615_TRINITY_DN94376_c0_g9_i2	1.601987	1.208206	0	0
Ctest_CP52k_homolog10_isoform_3	10.398	50831.0091	Ctest_170615_TRINITY_DN94376_c0_g9_i3	1.763001	1.268042	0	0

Additional file 3. Summary of all CP homologs. (page 13)

Assigned name	Species	NCBI accession no.	Orthogroup	CP	Base	Substrate	Full_lengthpartial	GRAVY
Ctest_CP52k_homolog11_isoform_1	Ctest	MW462765	OG0007385	52k	Membranous	Turtles/crabs	Full_length	0.034033613
Ctest_CP52k_homolog11_isoform_2	Ctest	MW462766	OG0007385	52k	Membranous	Turtles/crabs	partial	-0.031746032
Ctest_CP52k_homolog12	Ctest	MW462767	OG0007385	52k	Membranous	Turtles/crabs	partial	-0.082159624
Ctest_CP52k_homolog13	Ctest	MW462768	OG0007385	52k	Membranous	Turtles/crabs	partial	0.219631902
Ctest_CP52k_homolog14_isoform_1	Ctest	MW462769	OG0007385	52k	Membranous	Turtles/crabs	partial	0.170776256
Ctest_CP52k_homolog14_isoform_2	Ctest	MW462774	OG0007385	52k	Membranous	Turtles/crabs	partial	0.10982659
Ctest_CP52k_homolog14_isoform_3	Ctest	MW462775	OG0007385	52k	Membranous	Turtles/crabs	partial	-0.009090909
Ctest_CP52k_homolog14_isoform_4	Ctest	MW462770	OG0007385	52k	Membranous	Turtles/crabs	partial	0.131636364
Ctest_CP52k_homolog14_isoform_5	Ctest	MW462771	OG0007385	52k	Membranous	Turtles/crabs	partial	0.128125
Ctest_CP52k_homolog14_isoform_6	Ctest	MW462772	OG0007385	52k	Membranous	Turtles/crabs	partial	0.047005445
Ctest_CP52k_homolog14_isoform_7	Ctest	MW462773	OG0007385	52k	Membranous	Turtles/crabs	partial	-0.042307692
Ctest_CP52k_homolog15	Ctest	MW462776	OG0007385	52k	Membranous	Turtles/crabs	partial	0.201538462
Lanat_CP52k_homolog1	Lanat	MW462753	OG0003309	52k	Membranous	Floating objects	partial	-0.257792208
Mlong_CP52k_homolog1	Mlong	MW462778	OG0019083	52k	Membranous	Sponges	partial	-0.040425532
Mlong_CP52k_homolog2	Mlong	MW462779	OG0019084	52k	Membranous	Sponges	partial	-0.093835616
Mlong_CP52k_homolog3	Mlong	MW462780	OG0021274	52k	Membranous	Sponges	partial	-0.0785
Mlong_CP52k_homolog4	Mlong	MW462781	OG0030110	52k	Membranous	Sponges	partial	-0.552719665
Mlong_CP52k_homolog5	Mlong	MW462782	OG0030110	52k	Membranous	Sponges	partial	-0.454491018
Mlong_CP52k_homolog6	Mlong	MW462783	OG0003309	52k	Membranous	Sponges	partial	0.227338129
Mlong_CP52k_homolog7	Mlong	MW462784	OG0019083	52k	Membranous	Sponges	Full_length	-0.304020101
Mlong_CP52k_homolog8	Mlong	MW462785	OG0021274	52k	Membranous	Sponges	Full_length	0.092805755
Mlong_CP52k_homolog9	Mlong	MW462786	OG0019084	52k	Membranous	Sponges	Full_length	-0.542081448
Mlong_CP52k_homolog10	Mlong	MW462787	OG0007385	52k	Membranous	Sponges	Full_length	0.109964413
Mlong_CP52k_homolog11	Mlong	MW462788	OG0019084	52k	Membranous	Sponges	Full_length	-0.095530726

Assigned name	pI	estimated Mw	transcript ID	Base_R1	Base_R2	soma_R1	soma_R2
Ctest_CP52k_homolog11_isoform_1	10.287	53914.8606	Ctest_170615_TRINITY_DN97758_c3_g2_i3	0.633494	0.543224	0.051197	0
Ctest_CP52k_homolog11_isoform_2	10.256	57598.1575	Ctest_170615_TRINITY_DN97758_c3_g2_i5	1.227711	1.855951	0	0
Ctest_CP52k_homolog12	9.325	23973.6043	Ctest_170615_TRINITY_DN97758_c3_g3_i1	1.440206	2.168966	0	0
Ctest_CP52k_homolog13	10.18	18468.8617	Ctest_170615_TRINITY_DN99265_c0_g1_i1	8.614037	7.327284	0	0
Ctest_CP52k_homolog14_isoform_1	10.336	24951.4843	Ctest_170615_TRINITY_DN99265_c0_g2_i1	3.560897	3.010529	0	0.145786
Ctest_CP52k_homolog14_isoform_2	9.602	19875.1752	Ctest_170615_TRINITY_DN99265_c0_g2_i10	1.65241	0.848362	0.208463	0
Ctest_CP52k_homolog14_isoform_3	10.556	62237.0458	Ctest_170615_TRINITY_DN99265_c0_g2_i11	1.62564	1.800204	0	0
Ctest_CP52k_homolog14_isoform_4	9.933	31589.9892	Ctest_170615_TRINITY_DN99265_c0_g2_i3	7.227202	6.156328	0	0
Ctest_CP52k_homolog14_isoform_5	10.35	14622.1863	Ctest_170615_TRINITY_DN99265_c0_g2_i4	1.366688	0.485944	0	0
Ctest_CP52k_homolog14_isoform_6	10.593	62263.1367	Ctest_170615_TRINITY_DN99265_c0_g2_i5	7.034696	5.379341	0	0
Ctest_CP52k_homolog14_isoform_7	10.633	42141.3051	Ctest_170615_TRINITY_DN99265_c0_g2_i6	13.494533	14.19349	0	0
Ctest_CP52k_homolog15	10.13	14807.4120	Ctest_170615_TRINITY_DN99265_c0_g4_i1	3.540674	2.73256	0	0
Lanat_CP52k_homolog1	10.614	17836.4030	Lanti_TRINITY_DN24997_c0_g1_i1	6.897093	3.381745	0	0
Mlong_CP52k_homolog1	4.422	15574.6295	Mlong_TRINITY_DN11150_c0_g1_i1	15.049546	15.101557	0	0
Mlong_CP52k_homolog2	4.91	16447.5239	Mlong_TRINITY_DN14009_c0_g1_i1	25.756529	36.005332	0.690603	0.346974
Mlong_CP52k_homolog3	8.473	22693.0074	Mlong_TRINITY_DN15653_c0_g1_i1	1.067084	1.42397	0.352454	1.416022
Mlong_CP52k_homolog4	10.151	27064.1598	Mlong_TRINITY_DN1830_c0_g1_i1	2.4603	1.231205	0.174101	0
Mlong_CP52k_homolog5	9.618	19656.4050	Mlong_TRINITY_DN20086_c0_g1_i1	2.232396	3.531767	0.131098	0.131613
Mlong_CP52k_homolog6	4.018	15015.7840	Mlong_TRINITY_DN20508_c0_g1_i1	22.50997	24.61387	0.227447	0.342596
Mlong_CP52k_homolog7	5.416	22930.0499	Mlong_TRINITY_DN22409_c0_g1_i2	20.46897	19.450582	0.3082	0.618951
Mlong_CP52k_homolog8	4.425	15145.0676	Mlong_TRINITY_DN23404_c0_g1_i1	93.895559	93.737249	1.219866	1.137357
Mlong_CP52k_homolog9	4.191	24049.3848	Mlong_TRINITY_DN23544_c0_g1_i1	14.863442	14.194812	0.865604	0.48287
Mlong_CP52k_homolog10	4.209	30354.3883	Mlong_TRINITY_DN24074_c5_g7_i1	0	14.088322	0.267326	0.104363
Mlong_CP52k_homolog11	5.649	20435.4208	Mlong_TRINITY_DN24434_c0_g1_i1	51.373622	51.637722	0.9028	1.201079

Additional file 3. Summary of all CP homologs. (page 15)

Assigned name	Species	NCBI accession no.	Orthogroup	CP	Base	Substrate	Full_lengthpartial	GRAVY
Mlong_CP52k_homolog12	Mlong	MW462789	OG0019083	52k	Membranous	Sponges	Full_length	-0.196590909
Mlong_CP52k_homolog13	Mlong	MW462790	OG0019083	52k	Membranous	Sponges	Full_length	-0.055248619
Mlong_CP52k_homolog14	Mlong	MW462791	OG0019084	52k	Membranous	Sponges	Full_length	-0.123121387
Mlong_CP52k_homolog15	Mlong	MW462792	OG0007385	52k	Membranous	Sponges	Full_length	0.037006237
Mlong_CP52k_homolog16_isoform_1	Mlong	MW462793	OG0021274	52k	Membranous	Sponges	Full_length	0.055592105
Mlong_CP52k_homolog16_isoform_2	Mlong	MW462794	OG0021274	52k	Membranous	Sponges	Full_length	0.035526316
Mlong_CP52k_homolog17	Mlong	MW462795	OG0019084	52k	Membranous	Sponges	Full_length	-0.21686747
Mlong_CP52k_homolog18	Mlong	MW462796	OG0021274	52k	Membranous	Sponges	partial	-0.305217391
Mlong_CP52k_homolog19	Mlong	MW462797	OG0030110	52k	Membranous	Sponges	partial	-0.784827586
Mlong_CP52k_homolog20	Mlong	MW462798	OG0019083	52k	Membranous	Sponges	Full_length	-0.340883978
Mlong_CP52k_homolog21	Mlong	MW462799	OG0019083	52k	Membranous	Sponges	Full_length	-0.368527919
Mlong_CP52k_homolog22	Mlong	MW462800	OG0019084	52k	Membranous	Sponges	Full_length	-0.214012739
Tform_CP52k_homolog1_isoform_1	Tform	MW462809	OG0003309	52k	Calcareous	rock	partial	0.21559633
Tform_CP52k_homolog1_isoform_2	Tform	MW462810	OG0003309	52k	Calcareous	rock	partial	0.108496732
Tform_CP52k_homolog2	Tform	MW462811	OG0003309	52k	Calcareous	rock	partial	-0.0203125
Tform_CP52k_homolog3	Tform	MW462808	OG0007385	52k	Calcareous	rock	partial	-0.261676647
Wmill_CP52k_homolog1	Wmill	MW462802	OG0007385	52k	Calcareous	Corals	partial	-0.41

Additional file 3. Summary of all CP homologs. (page 16)

Assigned name	pI	estimated Mw	transcript ID	Base_R1	Base_R2	soma_R1	soma_R2
Mlong_CP52k_homolog12	5.565	19842.4893	Mlong_TRINITY_DN25508_c0_g1_i2	8.968987	6.847241	0.300541	0
Mlong_CP52k_homolog13	4.374	20219.9578	Mlong_TRINITY_DN25582_c0_g1_i1	2.861773	3.15847	0	0
Mlong_CP52k_homolog14	5.397	19063.6638	Mlong_TRINITY_DN26441_c1_g5_i3	55.333809	53.934533	22.161039	22.646725
Mlong_CP52k_homolog15	8.995	52873.5394	Mlong_TRINITY_DN27848_c0_g1_i2	13.058591	13.766924	1.477883	1.691943
Mlong_CP52k_homolog16_isoform_1	7.116	33351.7338	Mlong_TRINITY_DN30790_c11_g1_i1	99.962085	95.730919	0.238123	0.426407
Mlong_CP52k_homolog16_isoform_2	5.681	33413.7584	Mlong_TRINITY_DN30790_c11_g1_i2	26.220633	32.284203	1.401491	0.976459
Mlong_CP52k_homolog17	4.479	18769.0697	Mlong_TRINITY_DN32972_c3_g29_i1	2.845657	2.616252	2.239351	1.800179
Mlong_CP52k_homolog18	9.423	13150.0351	Mlong_TRINITY_DN3921_c0_g1_i1	0.596893	0	0	0
Mlong_CP52k_homolog19	9.781	17014.4494	Mlong_TRINITY_DN56954_c0_g1_i1	0.403596	1.21102	1.222827	0.40969
Mlong_CP52k_homolog20	4.699	21041.0107	Mlong_TRINITY_DN62463_c0_g1_i1	0	0	3.159617	3.90511
Mlong_CP52k_homolog21	4.427	21980.5470	Mlong_TRINITY_DN62806_c0_g1_i1	2.296248	2.121409	0	0
Mlong_CP52k_homolog22	9.152	18163.2119	Mlong_TRINITY_DN6982_c0_g2_i1	2.36054	2.557547	0	0.332156
Tform_CP52k_homolog1_isoform_1	9.469	12655.8262	TF_160309_TR47370|c0_g1_i1	2.444312	NA	0	NA
Tform_CP52k_homolog1_isoform_2	9.852	17816.8515	TF_160309_TR47370|c0_g1_i2	2.570802	NA	0	NA
Tform_CP52k_homolog2	9.873	14793.1378	TF_160309_TR47370|c2_g2_i2	3.707175	NA	0	NA
Tform_CP52k_homolog3	11.058	19123.8283	TF_160309_TR5993|c0_g1_i1	2.684122	NA	0	NA
Wmill_CP52k_homolog1	9.562	19038.0798	Wmill_TRINITY_DN16510_c0_g1_i1	4.658498	4.191092	0	0
