# Supplementary material for: Histology and transcriptomic analyses of barnacles with different base materials and habitats shed lights on the duplication and chemical diversification of barnacle cement proteins
Source: BMC Genomics. 2021 Nov 1;22:783. doi: 10.1186/s12864-021-08049-4 (PMC8561864; doi:10.1186/s12864-021-08049-4)
Supplement: Supplementary file 4 — Additional file 4 [file 12864_2021_8049_MOESM4_ESM.docx]

**File S1.** Alignment of all CP19k homologs.

>Aamph CP19k homolog3

----------------------------------------------------------------------------------------------------------------------------------------------------------------------------------------GSKGTR---------------------------------------------------RAQVVTGTKGQSISKGNAGTVQKAGANVGFQGAQAVRFTQPGQGQEIAVSVDK-KAGASSSSGHQSATRGSGSIGVENVGGTELR----------------------------RVNPARDPTDP------------------------DDSLSSDSQLRQTG-----------KTTGT-SSLTASGSTQGGGGARFNLWSPT--FNNSR----------------------------------------------------------------------------------------------------------------------------------------------------------------------------------------------------------------------------------------------------------------------------------------------------------

>Aamph CP19k homolog4

--------------------------------------------------------------------------------------------------------------------------------------------------------------D---SQLRQTGKTTGTSSLTASGSTQGGGGARFNLWSP-TFNNSRDFAGNTNVAGNGIASGN--GFFVQGVQANTELVSTKD-GLKVKTGTRGAGTTGGNAGLIEKAGANGKATDVAIITL---ADGSKQVQLVNSQKTTASTSSGLAASSKGDGRFRVDDKRETEVR-------------------VKPVDLNSEALKVPPAPQPGEGSKYI------------------DQYIPSDPKSGPSP-----------QPKPD-PQPKPDPQPKPEPEPEPKPEPEP-----------------------------------------------------------------------------------------------------------------------------------------------------------------------------------------------------------------------------------------------------------------------------------------------------------------

>Aamph CP19k homolog6

--------------------------------------------------------------------------------------MLS--LHLLTVCAAVAAAAALPVDP--------KTVEQPPPPPSTTPAPSKEGSGQ------LKPDFGFEIK----SRQSQSGTTSGGASVSSTGSSQGAVTGALNLATE-GYKLDLSAVGNSGVSGSGVSIGD--SGFRQKTQTNSEAGSKGTRRAQVVTGTKGQSISKGNAGTVQKAGANVGFQGAQAV---------------------------------------------------------------------------------------------------------------------------------------------------------------------------------------------------------------------------------------------------------------------------------------------------------------------------------------------------------------------------------------------------------------------------------------------------------------------------------------

>Aamph CP-19k-4-AQA26373.1

--------------------------------------------------------------------------------------MLS--LHLLTVCAAVAAAAALPVDP-------KTVEQPPPPPPPTTPAPSKEGSGQ------LKPDFGFEIK----SRQSQSGTTSGGASVSSTGSSQGAVTGALNLATE-GYKLDLSAVGNSGVSGSGVSIGD--SGFRQKTQTNSEAGSKGTKRAQVVTGTKGQSISKGNAGTVQKAGANVGFQGAQAVRFTQPGQGHEIAVSVDK-KAGASSSSGHQSATRGSGSIGVENVGGTELR----------------------------RVNPARDPTDPDD------------------------SLSSDSQLRQTG-----------KTTGT-SSLTASGSTQGGGGARFNLWSPT--FNNSRDFAGNTNVAGNGIASGNGFFVQGVQANTELVSTKDGLKVKTGTRGAGTTGGNAGLIEKAGANGKA--TDVAIITLADGSKQVQLVNSQKTTASTSSGLAASSKGDGRFRVDDKRETEVRVKPVDLNSEALKVPSAPQPEEGSKYIDQYI-----------------------------------------------------------------------------------------------------------------------------------------------------

>Aamph CP19k homolog7

-----------------------------------------------------------------------------------------------------------------------------------------------------------------------------------------------------GL--------------AASSKGD--GRFRVDDKRETEVRVKPV---------------------------DLNSEALRVPSAPQPEEGSKYIDQYIPSDPKSGPSPQPKPNPQPKPDPQPKPEPEPEPK-----------------------------PEPEPEPEPEPEP-------------------------------------------------------KPDPSPKGGYDKKA*----------------------------------------------------------------------------------------------------------------------------------------------------------------------------------------------------------------------------------------------------------------------------------------------------------------------

>Aamph CP19k homolog8

---------------------------------------------------------------------------------------------------ELRRVNPARDP--------------------------------------TDPDDSLSSD----SQLRQTGKTTGTSSLTASGSTQGGGGARFNLWSP-TFNNSRDFAGNTNVAGNGIASGN--GFFVQGVQANTELVSTKD-GLKVKTGTRGAGTTGGNAGLIEKAGANGKATDVAIITL---ADGSKQVQLVNSQKTTASTSSGLAASSKGDGRFRVDDKRETEVR-------------------VKPVDLNSEALKVPSAPQP------------------------------------------------------------------------------------------------------------------------------------------------------------------------------------------------------------------------------------------------------------------------------------------------------------------------------------------------------------------------------------------------

>Aamph CP19k homolog9

----------------------------------------------------------------------------------------------------------EGSGQ-------------------------------------LKPDFGFEIK----SRQSQSGTTSGGASVSSTGSSQGAVTGALNLATE-GYKLDLSAVGNSGVSGSGVSIGD--SGFRQKTQTNSEAGSKGTKRAQVVTGTKGQSISKGNAGTVQKAGANVGFQGAQAVRFTQPGQGHEIAVSVDK-KAGASSSSGHQSATRGSGSIGVENVGGTELR----------------------------RVNPARDPTDPDDS--------------------------------------------------------------------------------------------------------------------------------------------------------------------------------------------------------------------------------------------------------------------------------------------------------------------------------------------------------------------------------------------

>Cmala CP19k homolog1

--------------------------------------------------------------------------------------MLS--ARIFLLCMAVAVAVSVPVPG------------------RKTPKPG------------RRPDSSGAVK----SKLSQSGHTTGGATVSTQGSTKGSFRIKATIKGP-GKTVDQNSAANAGVSGSSVSAKE--GVFAQRSGAKTEITNKKG-RLSAETTSQGTGITGGGAGTMQNTGANGGANQRTTVTR-SLPAGKKVLRVKAAEKASVSSSSGHKSSTTGSGSFKVINKGGTDIK--------------------------------LELPEL---------------------------DIEVASSQQQAG-----------GTTRG-GSVSAEGATRGSAVGSSGLDLGK--IKGSKATAANAGASATSVSTGLGAFKHRTTGRTRVASSKDKVKVTSRTGGRGSTGGGAGIVEKSAARGRARHRKAIIVTLPDGKKRVRIVGSDRSSAKASSKHEASSSGLGDFKSATEVGTEIKLDPLKL*----------------------------------------------------------------------------------------------------------------------------------------------------------------------------

>Cmala CP19k homolog3

--------------------------------------------------------------------------------------MLL--RFTLLLCVALAVAVPTPSKK-----------------------------------GDKDDKSGLTTS----SEVSQSGITQGGGVVSSKGSSKGSTKSSSSFKAP-DVKIKRNLRASSGVSGSGASSGD--SAFGQKAGSKSVVSIDKD-EIIVRTGTKGKGFSTGDAGAIQSAGATAGGKQSTIIKL---PGGKKLPTIIDSSKGAAKSSSGHDASTAGEGTFKTINIGGTEVR-----------------------------LDDL-SP---------------------------DLDIEVKSKQGQAG-----------RTTRG-GNVNSHGSTQGSADSKSGFKAGK-KVTNRNNAAVNAGAAAQAASTDNGAFRQNAKSFSGAKTDKDGISVRTGNIGEGNTRGHSGVQQKTGANAGANKKQAIIITLPDGKKTVRIVDSGKGSAQSSSGQEASSTGKGSFRALNVGGTDVKLNGVKPGIDLSIESRQKQAGVTSKGGAVSSQGKSRGAGSDRFKLKAVDLKLTEKGAGNAGTSAQAHSAGNGAFKQDAKAKTDIKSNKDGLTVATETGGRGKTAGDTQIAQGTAANGKADQKKDSRQKYPVKSKHSEQGSASSSSGHDASSSDHGSFQTKNKGKTTIKSGDVKVSPKKG*

>Cmala CP19k homolog5

-------------------------------------------------------------------------------------MMRS--PLALLLCAAAVFAAPVPDHK-------GIIWLPTTTTPPPLPI--------------VVGRSGTAIN----SKLTQVGHTSGGAVVSSTGSTQGSSRFTRIVAGP-GGTVQQAGAGSAGVSGTSVSSGH--GVFVQRGQAKTTIKAGPD-GIDVKTGTQGEGFTDGTAGNIQKAGAGGGATQKQTVVT-VVPKGHQLVAVVASEESKATSSSGHEASSTGPGSFKTINLAGTGIA-----------------------------LTPLPNPLGGGT------------------------DVTITSKQKQGG-----------RTTHG-GALSATGATKGSVKTDSSRSGGG--VQERKAAAASSGAAGTAASAGNGDFFQDTVAKTQIVNSEDGLVVKTGTKGTGKSGGKSGIQQTAGAAGVGALSRVVIVTLPNGKRVVRIVKTDEAKASSNQGHQASSTGDGAFSTINLGGTEIKLDDPLKG*---------------------------------------------------------------------------------------------------------------------------------------------------------------------------

>Cmala CP19k homolog6

--------------------------------------------------------------------------------------MLA--RLPLLICAAAAAVAAPTVKH-----DASTVAPATTAAPATTSAPAKAATSDWELPSGGSARSGVRIK----SRLKQEGSTSGSGSVSSKGATRGSSKFKTSLTTP-DIKIDFSGAANAGLSGNSVSTGR--GAFSQKSQSTSGVSVSKT-GTDVTTGTKSLGATSGGAGSISKAGANAAARQKQAVVV-----GKKAARIADSAQGSARSTSRHDASSTGDGTFGISNVGGTSLR-----------------------------LGGVGSPDL---------------------------DFSVVSRRRQAG-----------STSAG-GSVASKGATRGSSKFKTSLTTPDIK---------------------------------------------------------------------------------------------------------------------------------------------------------------------------------------------------------------------------------------------------------------------------------------------------------------

>Cmala CP19k homolog7

SKGATRGSGRAKSGVSARQLKLKGASAANAGASTEAASAGNGKFQQKVAAKTDVRNNAKGLSVKTSTRSRGKTAGKVGISQKAGANGAANQRKAIKIAAPKGSAGSETSGA----ASAKSSSAHDAASTGDGTFSGKSTGGTEIDINGLDPEIDFSVE----SKQDQAGFSTSGGTLNTRGGTRGSARAKSRLSGP-NLKLQESGAANAGASGTGSASRGSLSAFVNKASSKSDVVSGKE-GLSVITTTNGTGRTLGGGSMAHKTGGNSHARQRKVVLV-TLPDGKKSVRLSESGEGSAKSSSGQEASSSGKGTFGTFNIRGTKVK--LSAFEPDLDLSLKSQQSQSSLSTSGTILRSLKNPKPKILPRKVSHRRVSKAKEDSESDGGKDSEEPVKGTKTAVNAHPVKINLITKSEPNTKAGLSTAAKDSGDDGQKVARAAQSVQERTAIKKTGPTVSSGGQGASSTSSTGEGEDPKSGVRSTQGRLSVESETEGRGKTDGGGGVNVRSRTRGNMQERTEV--------KTPGVVSSSREAVRAASALEASSSGKGDFNTVNRQGVDLKDKK*-------------------------------------------------------------------------------------------------------------------------------------------------------------------------------

>Cmala CP19k homolog8

------------------------------------------------------------------------------------------------------------------------------------------------------------------PILQSRHSDKSTHILFMCSTVVGLGRN----------------------------------------------------ALVVKTGTKGKSVTTGGAGTVQGAGADAGGTQEVNITL---PGGKKVARIVDVNKGSATSSSGHEASSTGEGRLKTINIGGTKVD-----------------------------LDGLRHDV----------------------------DIGVKSKQGQIG-----------RTADG-ASLNARGATQGEAVSSTGLN--------------------------------------------------------------------------------------------------------------------------------------------------------------------------------------------------------------------------------------------------------------------------------------------------------------------

>Cmala CP19k homolog9

--------------------------------------------------------------------------------------LSS--HVLLLFCVAVALTVPVPRRP------------------------------------------GASVT----SRLRQTGRTGGGAVVSSSGSSSGSVSTSSSSRRAAGSSLQVAGAANAGVSGTAVSRGV--GAFAQRAKAKTVIKANKK-GLSVKTSTAGRARTRGKAGAVQQAGANGGLTLKRVVIV-TLPNGLKTIRLVDAASGAASSSSGHKASSAAHGTFKIANFGGTEIK-----------------------------LKGPFDF*-------------------------------------------------------------------------------------------------------------------------------------------------------------------------------------------------------------------------------------------------------------------------------------------------------------------------------------------------------------------------------------------------

>Ctest CP19k homolog3

--------------------------------------------------------------------------------------MLG--VRLLLACVAAACAGPVPTKL-QTYIPSTPTAAPTSTATPPTPAPPAD----------LRPDLGVQIR----SRQTQTGSTSGGASVSATGSTQGSASNSIKLAGL-GYLLEKASIGNGGVSGSSVSSGD--ATFTQKAEADTVSNSAGTRAAKVGTSTKGLATTTGKAATVQKTGANAGFRGSQGVIF-NQPNSLKALKVRVAKKAGASASSGHRGSTKDVGAFTIENLGGTKLR----------------------------RVNTVLNPTNP------------------------NDSLSSDSGLKQVG-----------KTTGT-SSLSATGSTQGSGSAQVGLWTPS--LDRKKDVSGNTGVSGNGVSTGNGFFVQGVQAQTELVSTKDGLKVKTGTRGEGSTEGDAGIVEKAGADGKA--TDVAIVTLADGTKEVRLVNNKKATAVSTSGFSGSGSGKSTLNVVNEGETEVKLAKIDLNTSMPKFPSVPEALPPPELPNFPMTPTSMPSTTPEPTLAPLPVYKKGGK*---------------------------------------------------------------------------------------------------------------------------

>Tform CP19k homolog3

--------------------------------------------------------------------------------------MLS--VRLLLVWVAVATAGPLPSKL----RPEEYTKYAPTSAPTTTAAPATTKAPEA-----PKPDFGFQIR----SRQSQSGSTSGGASVSSTGSTQGSASNSVELSGI-GFVLKKSAVGNGGASGSSVSSGD--AAFNQNAATRTVAVSSGTRQAEVLTGTKGQAVTAGKAATVQKTGANIGFLGTQGIVF-NQPNGLKAVKVRVDKRAGASSSTGHQGSTKGTGAFGIDNFGSTELK----------------------------RANPAPEPTNPND------------------------SLSSDSQLKQVG-----------KTTGT-SSLSATGSTQGSGGAKFGFLTPI--VKRKKDFAGNSGVSGNGASSGNGFFVQGVQANTELVSTKDGLKVRTGTRGEGTTGGNAGIVEKAGANGKA--TDVAIVTLADGTKEVRLVNNKKATAVSSSGFTGSGSGDSALNVVNEGETEVKLNKIDLNAGIPTFPSVAAPASNPKLPKYPTPSPTTTPAPTTTPAPPTSSPKYLKNPKKYFAPAPTATSAPTTTLATPTNSLKYPKYHKHPSPAPTTTPAPTTTPAPTSAPSPKYSKGKKLFS*--------------------------------------------------------

>Majax CP19k homolog2

--------------------------------------------------------------------------------------MLS--AHRLIACAAIAAAAALPIEQ----KYVHYDPPAATTAAPSTAAPSSSPQPDGKEEK-LKPDFGFAIE----SKQLQTGSTSGGASISSTGSTQGSVSSIMDLSTD-IYNLNISAVGNGGLSSSSASSGV--GSFSQKAQINTDAGSIGTRRADLGTGTRGKASSRGNAGTVHKTAANVGLQGLETITFAEP--GLQFMTRVAK-RAGASSSTGHQGSTKDNGALTIDNKGATQLR----------------------------RLKPDLDPTDP------------------------NESLSSSSQLKQTG-----------RTTGM-SSLSATGSTQDSGGTRLTRWSPSQGLNNSRDLSGNTGVSGNGAATGNGFFVQGVQANTELISTKDSLKVRTGIRGGGTTGGSAGIVEKAGAKGKA--TDVRIVTLADGSKQVRLVNNQKTTAATSSGLSVSSSGNGTFNAQNSRETEVKVVPLNLDANVLKVSVLGSPAEPIPTAMPVVMPTGGPQYNVRYISTELPSTEKQVPTAAPTESLPEAKGKPTPE*---------------------------------------------------------------------------------------------------------

>Aamph CP-19k-7-AQA26379.1

------------------------------------------------------------------------------------------------------------------------------------------------------PLCGNLETA---AEMKQLGFSRGTGVFSSSSSSRGSANTKCRSRSD-TSENALAAAGNAAVTGAGNSVGG--GAFKQGARANTEVKQTPD-GVSVNTATGGSGVTAGSSATNQTTDAN------------------------------------------------------------------------------------------------------------------------------------------------------------------------------------------------------------------------------------------------------------------------------------------------------------------------------------------------------------------------------------------------------------------------------------------------------------------------------------------------

>Aamph CP-19k-6-AQA26378.1

----------------------------------------------------------------------------------------------------------------------------------------------------------------------------------------------------------------------------------------------------------------------QTTDANGGVNVRLLRNALRQGSDGGLVTFSSILRGRGTSSSGHSGSSSGDGELDVSNESGTQII-----------------------------FSQLTTPRPRGH---------------------------------------------------------------------------------------------------------------------------------------------------------------------------------------------------------------------------------------------------------------------------------------------------------------------------------------------------------------------------------------------

>Aamph CP-19k-5-AQA26376.1

---------------------------------------------------------------------------------------MIGAVHLLLVCLSASFAAENVTAP-----------------SAAAPSPA------------PLPDVRLRVE----SKQSHRGSTRGGASVSSRSEARGGASERLRLTDL-DLSLEKVARAETRAVTRSTSSGA--GAYDQFADTKGDVNSIAARKATSSTHSISYGNTTGGAYTGVVTGANARFSGELNATTSQEGGA-----IRRSKRTDEAANIVQSGASKNKGFFTFRNLADSEVK-----------------------------RSAEAGPAPSRE------------------------RETVASRAVQTG-----------RSEGS-AVVFTNGTAANLASSTEDS------PGSRRDLVGRSNVAGNASSIGHGFFRHSVKSGSDLAANRTALRAQAATSGDGETRRRAILHQSADSGGAGRHAGRTSSTPGADS----TISERMSVAEARQSHLASSRGQGNFSVSNEAQMVTEVQKPNA-----------------------------------------------------------------------------------------------------------------------------------------------------------------------------

>Aamph CP-19k-3-AQA26372.1

--------------------------------------------------------------------------------------MVS--ASLLLLCAAAASAVPLKATV--------------STTSPSTPPPSS-----------SGPVIDID------SKLASAVVTGGGAQVTTSGGTSGAASVTSTVRGP-GSVSTLTAVGQGGISGSSATAGN--SASLQKGRSKTVVEATTE-GTQVKTGTQGKGITSGEAVANQKAGAEGGAQRVEAVKY-VESDGKNLYKVEKVDRTAVKSASGHEASSRRFGTFNVLNLGSTAINNPGLIALPAQAPSSEPEPEPTPVYWNPKNPQPEPEPQPEPHP-EPEPEPEPHPKPTKPAAPQPKKELSSESKAKQAG-----------KTTGL-GAVSSTGATQGAAQSQTSVETPN--GSAKQAAVVNSGVGLTGVSSGTGLFNQKAHGKTAVTRTGESIDVTSISSGLGETDGAAGSIQSVATNGGFTAVDTLNLNLPG----LDLGSKAKASGTSSSGHKASSSGPGRFITSNEVGTEIKLTTPELDLETIHVPLAAPTTKPPKVKRGKW-----------------------------------------------------------------------------------------------------------------------------------------------------

>Aamph CP-19k-2-AQA26371.1

--------------------------------------------------------------------------------------------------------------------------------------------------------LGLSSK----SRQRQTGYTRGGAAVSSTGATQGAGSLDLAIDGPGGFKARSKALVNSGVSGAAVAAGN--GGFKQKSESETVGTVGLQ-GLDISTSSRGAGKSFGTAGVTQKQGANGGVSGAARV-----GGKYGHAKVAGALKGSATSSSGLDATSSGDSKFKGANAGKTSVK---------------------------FNVPSPDDALPSGVA--------------------PNIGVDSDSLLKQQG-----------GAVGD-ASISTNGGTQGGGSLVAGLRAPG--LQIDGSAAAKSGVSGAGALVGDGGFAQESIAGTSSSGRSDGLSVKSRSNGRGSAVGKVATRQGGSTGGGISGSIVTGLGTPDGK--FSIKGKHKGAAKGSIGHSASTDGTGSFSAINAVGTDVKFDVPSPDLALPGQGGLPSAPADTPVFKLTSTSDILQKGGSQGGAAVSGAGSTQGSGLGTVDLDTPHLQLDGEVVANSGVSGTAGSQGHGLFGQDANARTDAIANADGIETRTRTGGRGVTEGRAATAQKTGANGAIKARRDIVAQLPGEALGVSLPKGK--------------------

>Aamph CP-19k-AKZ20819.1

--------------------------------------------------------------------------------------MVS--PRILLAWAAVGIAVLSSPTL---------------------------------GAP-VPPPCDLKIK----SKVGQAAVTKGGAAVSTTGSSGGTGTVHCVVVGP-NKIVKKAAVGNTGVTGAGATAGD--GILKNLVKGVTEVKTTKD-GTKVKTKTAGKAGTGGTATIFQVADANGGVTEKSIKVDHLLTDDFEVIKIKEKKQGTATSSSGHKGSGVGDSLLKVVNEAETELK-----------------------------LKGLKLD--------------------------------------------------------------------------------------------------------------------------------------------------------------------------------------------------------------------------------------------------------------------------------------------------------------------------------------------------------------------------------------------------

>Aamph CP19k homolog1

--------------------------------------------------------------------------------------MVS--PQILLAWAAVGIAVLSSPTL---------------------------------GAP-VPPPCDLKIK----SKVGQAAVTKGGAAVSTTGSSGGTGTVHCVVVAP-NQIVKKAAVGNTGVTGAGATAGD--GILKNLVKGVTEVKTTKD-GTKVKTKTAGKAGTGGTATIFQVADANGGVTEKSIKVDHLLTDDFEVIKIKEKKQGTATSSSGHKGSGVGDSLLKVVNEAETELK-----------------------------LKGLKLD*-------------------------------------------------------------------------------------------------------------------------------------------------------------------------------------------------------------------------------------------------------------------------------------------------------------------------------------------------------------------------------------------------

>Aamph CP19k homolog5 isoform 1

------------------------------------------------------MQLPTVLLVWVAATTAAPWPQDPGTLPLSPPTARP--PIIITLPRRILRPTPQPTPE-------PTPEPTPQPTPQPTPEPTPQPTPEPTSPP-ALPLCGNLETA---AEMKQLGFSRGTGVFSSSSSSRGSANTKCRSRSD-TSENALAAAGNAAVTGAGNSVGG--GAFKQGARANTEVKQTPD-GVSVNTATGGSGVTAGSSATNQTTDANGGVNVRLLRNALRQGSDGGLVTFSSILRGRGTSSSGHSGSSSGDGELDVSNESGTQII-----------------------------FSQLTTPRPRGD*--------------------------------------------------------------------------------------------------------------------------------------------------------------------------------------------------------------------------------------------------------------------------------------------------------------------------------------------------------------------------------------------

>Aamph CP19k homolog5 isoform 2

------------------------------------------------------MQLPTVLLVWVAATTAAPWPQDPGTLPLSPPTARP--PIIITLPRRILRPTPQPTPE---------------PTPEPTPQPTPQPTPEPTSPP-ALPLCGNLETA---AEMKQLGFSRGTGVFSSSSSSRGSANTKCRSRSD-TSENALAAAGNAAVTGAGNSVGG--GAFKQGARANTEVKQTPD-GVSVNTATGGSGVTAGSSATNQTTDANGGVNVRLLRNALRQGSDGGLVTFSSILRGRGTSSSGHSGSSSGDGELDVSNESGTQII-----------------------------FSQLTTPRPRGD*--------------------------------------------------------------------------------------------------------------------------------------------------------------------------------------------------------------------------------------------------------------------------------------------------------------------------------------------------------------------------------------------

>Ctest CP19k homolog1

----------------------------------------------------------------------SDWVQRVVPATSRPATMLSSAAHLLLAYCTVATAVPLPAFELGLAYSGLHSTPAPPTSAPTTPAPTTQAPTVKRPA--ALPFCDDLKTS---SVVQQGGFTSGGAALSSRSATQGSAAVKCVVRGP-QLAAQLAAAGNSGVSGTGVSAGN--GIFQQGVKAATEVKSGGN-GVDVTTASAGTGKSGGGAAINQSSGANVGANLKIVGAGLLQTSELGLATIKNILQGGAKSSSGHKGSGSGNSALDVVNQSGTSIV--------------------------FDKLSPPAAPPTTPAP----------------------------------------------------TTTPAPSTPAPDTGLPDYFKFG*-----------------------------------------------------------------------------------------------------------------------------------------------------------------------------------------------------------------------------------------------------------------------------------------------------------------

>Tform CP19k homolog1

-----------------------------------------------------------------VKRRDMKRSRRVDPASPSPATMLS---ARLLVCVAIATAVPLPSGK----R--------------------------------KATPCGLLTI----SNLTQKAITKGGAAVSSTGATQGSGSVRCITVTP-VSVEKLAAVGNSGVSGSGVSAGN--GILKHIVTSGTVAKRTKD-GFKAKSGTSGSGASGGGAGILQDAAANGGTKLEIGELA-VLTDGKNLLKIKQVKKGSATTSSGHKASGSRESVFGVENRGGTEIT-----------------------------LDELDVPKIP--------------------------TLPKPNSSKSKG-----------KKKSG*----------------------------------------------------------------------------------------------------------------------------------------------------------------------------------------------------------------------------------------------------------------------------------------------------------------------------------------

>Tform CP19k homolog2

---------------------------------------------------------------------------------------MS--VRLLLVCVAVATAVPLPSGK---------LEPPTTKAPPTKPP--------------TLPYCDDLKTI---SKLKQAGFTKGGAAVSSSSSTQGSASVKCIVRTP-KSQTKLNAAGNSGVSGAGVSASG--GIYKQGVEAATEVKTSNE-GVEVKTESQGTGGSAGGAAINQNAGANGGAKLNIVGVDLLKNGKLGLAVIKRVLKGGTTSSSGHKGSGSEDSVFKVANQGGTKIV-----------------------------FDKLTPPTLPPT--------------------------------------------------------KPAPTTQAPKLKHLKLH*-------------------------------------------------------------------------------------------------------------------------------------------------------------------------------------------------------------------------------------------------------------------------------------------------------------------

>Tform CP19k homolog5

------------------------------------------RVNPPTPSRGQRVNPPTPSRGQRVNPPTPSRGQRVNPLSPSPAIMLS--FRLVLVPVAMAITVLLASGK----------------------------------PATALPPCDLKIV----SKLKQAGITEGGAAVSTTGSTQGSGVIKCVFKSP-TSVVKKAATGNSGVSGASISADN--GAFKNLVEALTDVKTTKK-GTKVKTESAGEGATTGKATTLQNAAANGAATLTTAEID-LLTSGKGLFKVKEVKRGSATSSNSHKASGSGKSLFKVLNLGETELK-----------------------------LEGTLSDELDD*---------------------------------------------------------------------------------------------------------------------------------------------------------------------------------------------------------------------------------------------------------------------------------------------------------------------------------------------------------------------------------------------

>Aamph CP19k homolog2

-----------------------------------------------------------------------------SAAPSEPNRMVS--ASLLLLCAAAASAVPLKATV--------------STTSPSTPSPSP-----------SGPVIDID------SKLASAVVTGGGAQVTTSGGTSGAASVTSTVRGP-GSVSTLTAVGQGGISGSSATAGN--SASLQKGRSKTVVEATTE-GTQVKTGTQGKGITSGEAVANQKAGAEGGAQRVEAVKY-VESDGKNLYKVEKVDRTAVKSASGHEASSRRFGTFNVLNLGSTAINNPGLIALPAQAPSSEPEPEPTPVYWNPKNPQPEPEPQPEPHP-EPEPEPEPHPKPTKPVAPQPKKELSSESKAKQAG-----------KTTGL-GAVSSTGATQGAAQSQTSVETPN--GSAKQAAVVNSGVGLTGVSSGTGLFNQKAHGKTAVTRTGEGIDVTSISSGLGETDGAAGSIQSVATNGGFTAVDTLNLKLPG----LDLGSKAKASGTSSSGHKASSSGPGRFITSNEVGTEIKLTTPELDLETIHVPLPAPTTKPPKVKRGKW*----------------------------------------------------------------------------------------------------------------------------------------------------

>Ctest CP19k homolog2

------------------------------------------------------------------------PAPLSPVRGTQPAAMLL--AHLLLACVAFASAVPVPSNY-----LPRTTTTTTTAAPATSRAPPKAKVPVA-----SNPLVGVD------SKLTSNVVTSGGAKVQTTGSTSGSGSLTTVITGP-GLTSRTTASGSGGVSGTSASAGN--GLSKQKSRAKTVVTDSNG-GPKVQTATEGQGNTVGPAAATQSAAADGNVNTLKAIKF-VQSDGKNLFQVEQIRKSAAKSGSKHEASTKGLGSFRTLNLGSSSIQ--------------------------GSGLGSLPLPGVPAAPVTLD----------------PKGSISSDSSLKQSG-----------KTTGG-GSISSLGATQGSTSLEAAGNSPG--GGIKVSGVANSGVSGAGVSSGNGVYNQGAQGKTVVDGNKNGGKVTTGSTGFGFTDGTAGALQEVAANGGFTGLGTLDLKLPSGNNLIHVQSVKKGSGTSSSGHKASSTGLGKFGTTNIASTDIKLQTPDLDLSVPLLPPVVPATTTTTTTTTTTTTAATTTTHRPYKKYSK------------------------------------------------------------------------------------------------------------------------------------

>Cmala CP19k homolog10

-------------------------------------------------------------------------------------------------------------------------------------------------------------------------------------------------------------------------------------AGKTDVKSTDN-GISVSTGSAGKGKTDGDAAVLQKTGANGGARQQRSLSG-SQGAGKGAIRAEQSEDGTASSTSGHDASSTGDGRFKTRNEASTEVK-----------------------------QNAEHAPK*------------------------------------------------------------------------------------------------------------------------------------------------------------------------------------------------------------------------------------------------------------------------------------------------------------------------------------------------------------------------------------------------

>Majax CP19k homolog1

--------------------------------------------------------------------------------------MSS--ARLLLLCAAAASAVPLPSGF--------------RPPTPTTPAP-------------PLPSSSSESLIGIGSKLSSSIVTGGGAVVNTRGSTSGSATLHTSYKGP-GESSETTTVGKTGLSETSAAASG--GVSIQKNRAKTVVDSTEE-GIEVKTGTEGKGITDGKAAGTQKAGAEGGAKTVDTTKF-VYTDGENSFQVQHVERTAAESTSGHEASIEGFGTFGVLNFGTTITK-----------------KMRVPKVPKSPAPAPTSAPALSPEPDYWHDEPKPVTTTAAPTTV-SPQELSSKSKMKQAG-----------KTSGT-GAVTASGATQGSTTSETDVKTPT--GNAKQAAVANSGVSGTGVSSGESGFMHVAVGGTVVFKTKDGAKVVTTSDGFGNTKGASGTIQTVAVNGGLSVLDNIDLDLPG----FNIRSKKKVSGSSSTGHEASSAGDGEFVAKNLAGTEIKLLSPELSLDEISMPTLPPTTTTVKPPKEPKFKLGKW*----------------------------------------------------------------------------------------------------------------------------------------------

>Cmala CP19k homolog2

--------------------------------------------------------------------------------------MRL--PISLVCLAALVTTAPASGGK-------------------------------------SKRKCGVSIL----SKLHQSGSTSGGGAVSAHGKTQGSARFRCRVQGP-GFLVDSAGVGSAGVSGTGVSSGL--GAFDQDAAALTEIISKPG-GSKVLTLTEGKGSSGGTAGVLQKAGASGGVSKKKIGIL-QKNFLKGLVTAVKKVKAQGTSSSGHTGSSTGFGDFKFKGGSVTDIK-----------------------------LPSAL*---------------------------------------------------------------------------------------------------------------------------------------------------------------------------------------------------------------------------------------------------------------------------------------------------------------------------------------------------------------------------------------------------

>Tform CP19k homolog4

-----------------------------------------------------------------------------PACPAQPLTMLS--AHLLLVCVAVATAVPLPYKH-----------YTTTTAAPATSTAAP-----------ARSSRLIDVD----SKLTSNVITGGGAVVQTSGSTSGAGSVTRVVSGP-GLVFRTTAVGSGGVSGQSASSGD--GLAAQLSRARTVVVDTED-GTKVQTGTEGKGTTLGTAAAAQKTGANGGVNTLKAIKF-VETDGKNLFQVEQVQRAAAKSASGHEASSEGLGSFNVLNLGSTEIK-----------------------HSDFPSLPPPAGPAPTKKPKHWKPTAAPTTTTEAPTTASPKFGISTNSKLKQAG-----------KTTGS-GAISSTGATQGSASSKSGLKTRA--GGAKQSAVANSGVTGAGVSSGDGAYKQGAEGKTVVAATKEGVKVTTGSTGFGFTDGTAGTLQNVAANGGFTGLSTLNIKLPTGKHLVNIQGIQKAAGTSSTGHKASSTGPGKFATTNIAGTDIQLKTPDISLVAPLIPTPAPTTTAAPSTAAPKLKFHKW*----------------------------------------------------------------------------------------------------------------------------------------------

>Cmala CP19k homolog4 isoform 1

---------------------------------------------------------------------LKGSPLRDSSYSSVKSATMR--FFVILLCVAVALAVPTGNRR---------------------------------RPA-PKRRCNLTTL----SKLGQKGHTSGGGAVSASTSTQGSGSINCVVKGP-NFKVDTAAAGNSGVAGTGVAAGD--GAFGQKVGAGSGVKNVPG-GTKVTTGTAAGGATRGGAAVTNKAGANAGAKTIAEVKR-----RLKNIKIGQKQEASGTSSSSHEASSTGDGTFAVDQKGKTVIK-----------------------------LKGPLGR*-------------------------------------------------------------------------------------------------------------------------------------------------------------------------------------------------------------------------------------------------------------------------------------------------------------------------------------------------------------------------------------------------

>Cmala CP19k homolog4 isoform 2

---------------------------------------------------------------------LKGSPLRDSSYSSVKSATMR--FFLILLCVAVALAVPTGNRR---------------------------------RPR-PKPICNPSTL----SKLGQKGHTSGGGAVSASTSTQGSGSINCVFKGP-NLKVDTGAAANSGVAGTGVSAGE--GAFGQNVRAGSGVKSVPG-ITKVTTGTAAGGATGGGAAVTNKAGANAGAKTSV-----EFGRRLKKIKILEKQERSGTSSSGHEASSTGDGTFSVDQKGKTVIKLKGP-------------------------LVGVISPLDN*----------------------------------------------------------------------------------------------------------------------------------------------------------------------------------------------------------------------------------------------------------------------------------------------------------------------------------------------------------------------------------------------

>Cmala CP19k homolog11

--------------------------------------------------------------------------MERRKLWNRKTGNYV--FDNKKSVEPISNLAPSGNRR---------------------------------RPK-SKPTCNVSTL----SKLGQRGHTSGGGAVSGTTSSQGSGSIACVFRGP-GLSIDAREAARSGVAGSAASNGH--GAFGQTAGANSGVAFVPG-LASVTSGTGGDGTTLGGAAIAQEAGAHAGAKIGSQIDL-----HLRKVDIGQAAQAAGTSSSNHRGSGNGDSKINVKQQSETKVK-----------------------------IKGPLAG*-------------------------------------------------------------------------------------------------------------------------------------------------------------------------------------------------------------------------------------------------------------------------------------------------------------------------------------------------------------------------------------------------

>Cmite CP19k homolog1

-------------------------------------------------------------RGRPSPIRPRRRPARRVAAPDLAAAMLS--ARLFWICLGAAAAVPLQLPS-------------------LTPVLG------------TPPNCNMSSH----SGLKQSGQTTGTGSVSTTGSTTGSVSGLCAFTGP-NSGTQDTGAGNSGVSGSGVSAGN--GIFGQTVDAGAGVGTAPD-KTVVQTATGGSGGSAGTAGTVQKAGANVGAEKKKTVIV-TLSDGTKVVKIVDTGKGTSTGSTGHDGSTTGFGTFHVQQNTTSVIK-----------------------------LKPPLQG*-------------------------------------------------------------------------------------------------------------------------------------------------------------------------------------------------------------------------------------------------------------------------------------------------------------------------------------------------------------------------------------------------

**File S2.** Alignment of all CP20k homologs.

>Aamph_CP20k_homolog2

-MKSSVLALCLATVLAVLYVSVDAHEDHGHDHGHRSRHCTPSHRCYYC------HYDCECNHLHDQCKPS-------HPCYRKLPGS-----HSDCDCNH--VKPCNPKHPCWHKYTVKKGHKQHKRIYGCNCNHLR-CNRKHPCWHRHCDCYCKHQH*

>Aamph_CP20k_homolog5

-MKSSVLALCLATVLAVLYVSVDAHEDHGHDHGHKSRPCGPGHRCYYC------HYDCECNHLHDQCKPS-------HPCYRKLPGS-----HFDCDCNH--VKPCNPKHPCWNKYTVKKGHKQR-----CNCDHLR-CNRKHPCWHRHCDCYCKHQH*

>Aamph_CP20k_homolog3

-MKSSVLALCLATVLAVLYVSVDAHEDHGHDHGHKSRHCGPGHRCYYC------HYDCECHHLHDQCKPS-------HPCYRKLPGS-----HFDCDCNH--VKPCNPKHPCWNKYTVKKGHKQR-----CNCDHLR-CNRKHPCWHRHCDCYCKHQH*

>Aamph_CP20k_homolog4

-MKSSVLALCLATVLAVLYVSVDAHEDHGHDHGHKSRPCGPGHRCYYC------HYDCECNHLHDQCKPN-------HPCYHK-HGS-----DYDCNCNH--LKPCNSKHPCWHGHKV-KGSKKI--IYDCDCDHLGYCNAKHPCWHRHCDCYCKHQH*

>Aamph_CP20k_homolog7

-MKSSVLALCLATVLAVLYVSVDAHEGHGHDHGHKSRPCGPGHRCYYC------HYDCECNHLHDQCKPS-------HPCYHK-HGS-----DYDCNCNH--LKPCNSKHPCWHG---------------HKVKGSK-------KIVYDCDCDH-----

>Aamph_CP20k_homolog8

-MQSTLFVLFLAAASTVFYAAGQGQGP----------PCNPGNPNFNC------TDSCDCAELRF-CMPA-------FPCFRLPSGN---ETEPSCDCNQ--LTPCDSSHPCWRTTVV-NGTLTD---NNCDCDSIA-CSISHPCRHRQCSCD*-----

>Aamph_CP20k_homolog6

-MQSTLFVLFLAAASTVFYAAGQGQGP----------PCNPGNPNFNC------TDSCDCAELRY-CMPA-------FPCFRLPSGN---ETEPSCDCNQ--LTPCDSSHPCWRTTVV-NGTLTD---NNCDCDSIA-CSTSHPCYHRQCSCD*-----

>Aamph_CP20k_homolog1

-MKRTQLALCLTAVIVAVFVAADARGQK--------RNCNPGNPCFHC------QDTCDCSSEGLFCTPA-------HRCFHIHVSNMSTTREVECNCNH--LTPCDSSHPCWHPTVV-NETLTT---YDCDCDSIE-CSIRHPCWHRECGCNCTHTA*

>Chunt_CP20k_homolog1

-MKLSVLVVMLSLVVVAL-AHKDYHKKHNPKN---HGVCNADAPCWHCKCNKKGVRSCNCSCKDMKCIGKSTRWYKRHPCYHC-HGK---KRRCDCDCTHEDELPCNRRHPCFH----------------CHGKGKK---------YCHCGCEHSHTG*

>Cmala_CP20k_homolog1

-MKLSVLVVTLSLVVVAL-AHKDYHKKHNPKN---HGVCNADAPCWHCKCNKKGVRSCNCSCKDMKCIGKSTRWYKRHPCYHC-HGK---KRRCDCDCTHEDELPCNRRHPCFH----------------CHGKGKK---------YCHCGCEHSHTG*

>Cmite_CP20k_homolog1_isoform_2

MMKTTRVLLLFFVVVAAISATAEAHEDH---------ECNNKTKCWNC-ITKDGKEDCNCDCNRMKCDDK-------HPCYHCYKDA-NGKMHFDCDCHH---IKCDKRHACYH----------------CHCKGKS-------CDHCHCDCTHSPD*-

>Cmite_CP20k_homolog1_isoform_3

MMKTTRVLLLFFVVVAAISATAEAHEDH---------ECNNKTKCWNC-ITKDGKEDCNCDCNRMKCDDK-------HPCYHCYKDA-NGKMHFDCDCHH---IKCDKRHACYH----------------CHCKGKS-------CDHCHCDCTHSPD*-

>Cmite_CP20k_homolog1_isoform_1

MMKTTRVLLLFFVVVAAISATAEAHEDH---------ECNNKTKCWNC-ITKDGKEDCNCDCNRMKCDDK-------HPCYHCYKDA-NGKMHFDCDCHH---IKCDKRHACYH----------------CHCKGKS-------CDDCHCDCTHSPN*-

>Cmite_CP20k_homolog2

--MKTVRVLFLLVVVVAISATAEAHEVP---------VCNALAKCWNC-ITKTGKKDCNCDCDRITCAKGI------HPCYHCHKNG-KGKNECDCDCGH---IECDKHHPCYH----------------CHS-----------CFDCHCDCTHSPQ*-

**File S3.** Alignment of all CP43k homologs.

>Aamph_CP43k_homolog3

---------------------------------------------------------------------------------------------------------------------------------------------------------------------------------------------------------------------------------------------------------------------------------------------------------------------------------------------------------------------------------------------------------------------------SASRRASRTEAANKGEVETRGDGAGAVGESSSKATATESGTSAAESSA-NNRLAATRTGRAETSIFTEGGSTLGLDQFSGDV------VGAVKSLTSGSQSSLGSANSKVNAASSAQNA-LGGGAALGDSTFRAQTSGTGAVSGTLETRAQNRLKADAQPAQLSLVERIRQQQQQKEAEQGKGSVLPSAGKGKGPRPTQPPPPPTLPSPPRGKH*--------------------------------------------------------------------------

>Chunt_CP43k_homolog1

---------------------------------------------------------------------------------------------------------------------------------------------------------------------------------------------------------------------------------SSDSTTDADASSDSTTDADASSDSTTDADASSDSAKSANAD---------------------------------------------------------------------------------------------------------------AVATSAPQAAERKQVGEAISQPTDDVI-------PASTAITRTTAYAEGQQKTSGDAVTISNTQGKSAVISPLGVIETDAQF--GGLLASNDASLQGSIFTQGGLAQLPQTVPS--------FPAFQARPLGNLNIFQKIAAKREAASRDAKA----------ADQGAEVIGQGSTE*-------------------------------------------------------------------------------------------------------------------------------------------------

>Cmite_CP43k_homolog1

-----------------------------KVWLLIPAVLCAA-------------------------------------RAAPTGRGRGPPLVVPPGPLPGPPVVPPPPP-------------------------------------PPPVVVPVPPPPPPV------------------------VPVLPVPAPSAEGTKSKAVTGGSLQTTGNARADVSGGSTSAEQTGDQKTKAHVRTELTSQEHGLAATKAKSVGQSAKVAGGG-VEDATSQAKTLTQSVSAGQATSQTDAVADAATVGLGTKTKAEGGTSSASRGNGQAGGGSKVVSSAGSQAVPGGEGSSSVVSTETEGQSTGAGGTQSAGRGASAGVTG-GTSSISRAQTGSDTLGDGVAAARKTVYSKGAVGGDGAGVGTATKAGAKGDVATNGPGAASAAEGNTKGRARAGDSLTSKSTA-NTQLRSTNTGAASVEVKSSSGSTVASDDLESF-------VTATQARVAGQQSSDGSASTRANTAGTSGVR-TPSGGGVADSAYRAETGGTAAVKGGQEAGGVTVVRRGPSVVDDLVKKVLPKGG*-------------------------------------------------------------------------------------------------------------------

>Ctest_CP43k_homolog2_isoform_1

-MRCVAGRRAPVAARHRRPPTSVAANTMMTCWIALSAALGAL-CAYTNGAPTPGGKGGGYRHVGLGTPSHLGTYPV------VTGTVPQVPVAPVYPGVPGGPVVPGQPGVPGGPVGPGYPGVPVGPVG------------PVNPGVPVGPVVPVNPGVPVGPVVPVNPGVPSVVTPGHPGVPDVPVNAVLPEGKRNLGTKSQTDSAGKIISTGNAKAFASGGARASDMLPDQKSGADVRLTLKTQREGASEGVAGSVGRSDAAIPDG-RQHSSSQGDAHTEVRSTGQSSTEADANARGATLLKNAKTDATGGSSSATVNEGTAAGASETLGSAGRK---GPHSTDAGSGAASQSTSTGSGGSKSVGRGRGAAFGG-GTTSLAGSQSETDTIGDGKAQSVQRGGAGGELDGNGVLRVGGSKARSQGDVKTLGSGASGSGESTSKGTAGKDSKTSSESVA-RNQLKSTRTGRAAADVITEGLSALGLDTFTKDL------VVDTKSKAASNQRSVGSASSAANGASTAGST-LAGGASLTDSTVQGKTSKAGEIRSVLETRGQARVSEGKPVV-----------QPLPEAPSILSKPSAAPVIPPPPPTPPPTSPPYVPPPPVQPSHVPPYVDLPQSQESRAPVYVPVPSPAPVPYVPPHVGHKPVYHYPKKAPYVSQKKGYRHYG*--------------

>Ctest_CP43k_homolog2_isoform_2

-MRCVAGRRAPVAARHRRPPTSVAANTMMTCWIALSAALGAL-CAYTNGAPTPGGKGGGYRHVGLGTPSHLGTYPV------VTGTVPQVPVAPVYPGVPGGPVVPGQPGVPGGPVGPGYPGVPVGPVGPVNPGVPVGPVVPVNPGVPVGPVVPVNPGVPVGPVVPVNPGVPSVVTPGHPGVPDVPVNAVLPEGKRNLGTKSQTDSAGKIISTGNAKAFASGGARASDMLPDQKSGADVRLTLKTQREGASEGVAGSVGRSDAAIPDG-RQHSSSQGDAHTEVRSTGQSSTEADANARGATLLKNAKTDATGGSSSATVNEGTAAGASETLGSAGRK---GPHSTDAGSGAASQSTSTGSGGSKSVGRGRGAAFGG-GTTSLAGSQSETDTIGDGKAQSVQRGGAGGELDGNGVLRVGGSKARSQGDVKTLGSGASGSGESTSKGTAGKDSKTSSESVA-RNQLKSTRTGRAAADVITEGLSALGLDTFTKDL------VVDTKSKAASNQRSVGSASSAANGASTAGST-LAGGASLTDSTVQGKTSKAGEIRSVLETRGQARVSEGKPVV-----------QPLPEAPSILSKPSAAPVIPPPPPTPPPTSPPYVPPPPVQPSHVPPYVDLPQSQESRAPVYVPVPSPAPVPYVPPHVGHKPVYHYPKKAPYVSQKKGYRHYG*--------------

>Ctest_CP43k_homolog2_isoform_3

------SKRGPSVDSDLR-FVALLTEEITLCYETGAGYLKCVEEAYTNGAPTPNAKDWDSHPFG-GSFNNRMAYPTGLPSVQLNQHVPHVNLHPITHSGTGRTHLPTVSHLPSAPYVHGV---------------------RHAPNLPSYSSLPRVPGVSHTPLAPQQHGLPAV-----SGIPD---NLLALRRRLNLGTKSRANSAGKVISTGNAKVLVSGGAQVSERLPDQKSGADARLTLKSQREGVGEGIAGSLGRSDAAIPDG-RQHASSQGDAHTEVRGTGESVTEADANARGATLLKNAKTDATGGSSSATVNEGTAAGASETLGSAGRK---GPHSTDAGSGAASQSTSTGSGGSKSVGRGRGAAFGG-GTTSLAGSQSETDTIGDGKAQSVQRGGAGGELDGNGVLRVGGSKARSQGDVKTLGSGASGSGESTSKGTAGKDSKTSSESVA-RNQLKSTRTGRAAADVITEGLSALGLDTFTKDL------VVDTKSKAASNQRSVGSASSAANGASTAGST-LAGGASLTDSTVQGKTSKAGEIRSVLETRGQARVSEGKPVV-----------QPLPEAPSILSKPSAAPVIPPPPPTPPPTSPPYVPPPPVQPSHVPPYVDLPQSQESRAPVYVPVPSPAPVPYVPPHVGHKPVYHYPKKAPYVSQKKGYRHYG*--------------

>Ctest_CP43k_homolog2_isoform_4

-MRCVAGRRAPVAARHRRPPTSVAANTMMTCWIALSAALGAL-CAYTNGAPTPGGKGGGYRHVGLGTPSHLGTYPV------VTGTVPQVPVAPVYPGVPGGPVVPGQPGVPGGPVGPGYPGVPVGPVG------------PVNPGVPVGPVVPVNPGVPVGPVVPVNPGVPSVVTPGHPGVPDVPVNAVLPEGKRNLGTKSQTDSAGKIISTGNAKAFASGGARASDMLPDQKSGADVRLTLKTQREGASEGVAGSVGRSDAAIPDG-RQHSSSQGDAHTEVRSTGQSSTEADANARGATLLKNAKTDATGGSSSATVNEGTAAGASETLGSAGRK---GPHSTDAGSGAASQSTSTGSGGSKSVGRGRGAAFGG-GTTSLAGSQSETDTIGDGKAQSVQRGGAGGELDGNGVLRVGGSKARSQGDVKTLGSGASGSGESTSKGTAGKDSATSSDSVA-RNQLKASRTGRAKADVITEGLSALGLDSFIDDL------VVDTKSKSASNQQSVGSASSAVNGGSTAGSS-LTGGAAVADTTVQGKTSKAGEIRSVLETNGQARVGDGKPVV-----------QPSQPPPKIIKTPSSAPIIPPPAPTEAPTSPPYVAPPPAQPSHSPPYVPLHQSQKSHVPGYFPHSSPSPVPYVPQHADHSPVYQSPHKAPRASPKKSYGYYPSKKDRRVPQPWELH*

>Lanat_CP43k_homolog1

--------------------------MRTLVVLAALAVLASG----------------------------------------APTNKRQRQLPTVPPRVPVAPVKIQIQ--------------------------------------VPTPVQPAAPQIQQP------------------QVVVVQAPQQEPIDANNIASESTAIGSASISTNGNARSAVSGGTQATQSIPSQRTDAILKLSLESQEEGDVTAAGRSDSRTDIASAGF-SEGSSSQTDALTNVFSSGQAQGAIDANAAGATTGAATRTDADSGSSSASRGDGEAQGASQSIGTGGFGIAGSGEQSDAGGSSTSTAESFGDAGTKTVGRGRAVSTGGQDTTAAAGAQTGVNTIGDGAGTSQETSLSSGISVGDGVGQAVSSDSSTIGAVQTFGPGAEALGEGTGQATAERDGLQTSKSTT-SNQLNSAGDGAAVAETGSIGASALSLDDI-GAP------NTRTQAAAQGFQSSTGNANSRANTVGEGTVL-TPLGGSTSDATFRGVTAGDGSIEGSILTEGGNRLDLALP-------------GSLPGLGDSSKAKSVLEQIAGGPRQPSDPNNPKNPTSLKG*----------------------------------------------------------------------------

>Majax_CP43k_homolog2

---------------------------MHPWVVVLAATLGVA----------------------------------------T--------------AAPCVTCVVKD---------------------------------------------VISGSLPRPVPVRQTHSADGVVVVGAPGHAQ--QSDIVRVEKRGVGTKTRADVRGSIISIGNAQAVISGGARAAEQMADQNAGADVRLKLDAQRNGLGAGKAAAVGRSDMS-PAG-L-AGSSQGDAITETRSTGEVSTEADAVGGGATLSGGVRSDAHGDSSSATYGDGTAAGITQTVGTGGQQ---PGHATDAGTGSVSQVESSGSGGSKSAGRGRGVAIGS-DTTSASGSQTKVDVMGDGKAQAFERGTARGELAAGGPVRCGSTQVGSEGDVKTLGDGASGAGETTVKSTADQNGTTSSDSVA-RNQLKASRDGVAKAEVFTTGMSALGLDEFSKDL------VVDTKSTTRGSQTTAGSAAAAANGGSTSGST-LVGAGTVSDTTFTGKTSQAGHIKAQLTARGQGRVTDGEPVVVAQNTANPDEDEPEPPVPVEHPVPY-----VPPQPSPEPYVPPYVPPQPSPAPYVLPFVRSRPNPALHVPPYVR-PQPNPAPYVPPYVRPQPSHGPPSEQYNQPYHRSGAYKKPHYVTY*--------

>Tform_CP43k_homolog2

----WAAGRWRQWPPVTSCRQHISQQLGMSFWIVLVAALSAA------------------------------------------------------GAAPCHKCGAPGGRPSAVVVV------------------------------ATPVRPPYPPGLPPK---------------------------------LSLGTKTKADVAGKVISTGNAQVVVSGGAKAAERLPDQKSGANARVTLRTQENGLGAGSAGSVGRSDVGRPHG-HEHASSQGDAHTETRSTGQASTEADANSSGKTTGSGAKTDASGGSSSATTGEGAAAGASQTLGTAGKH---GAHSTDAGSGSASQAESSGSGGSKVAGRGRGAALGG-GTTSVSGSHTRVDVIGDGKAQAVQRGGAGGELAGDALVRVGHTHAGSQGDVKTQGPGASGTGGSASKGTAGKKGTTSSDSVA-RSQLKASKDGAAKASVFSTGLSALGLDSFTKDL------VVDTKSKTTGTQTTLGSGSSAANGASTAGSS-LVGGDAVADSTFQGKTSGSGHIKGILESRGQGRVADGKPVVQPQKPAPDH--IKKPGPPPYAPPHPAPKPYVPPHPSPKPYVPPYVPPPPSPSPYVPPYAPPGPRNSPPYKP---------------------------------------------------------

>PECT_CP43k_homolog1

--------------------------------------------------------------------------------------------------------------------------------------------------------------------------------------------------------------------------------------------------------------------------------------------------------------------------------------------------------------------------------ASAGVTG--GTSSISRAQTGSDTLGDGVAAARKTVYSKGAVGGDGAGVGTATKAGAKGDVATNGPGAASAAEGNTKGRARAGDSLTSKSTA-NTQLRSTNTGAASVEVKSSSGSTVASDDL-ESF------VTATQARVAGQQSSDGSAST-------------------------------------------------------------------------------------------------------------------------------------------------------------------------------------

>AQA26374.1_CP-43k-2-AQA26374.1

--------------------------------------------------------------------------------------------------------------------------------------------------------------------------------------------------------------------------------------------------------------------------------------------------------------------------------------------------------------------------------GRGAAFGS-DTTSLSGSQTRSDVIGDGQARSVETGGAAGQKGDG----ISQTQATSKGKVHTLGNGASGDGETSAKGTAGTNSTTSSDSTA-SNRLKATRGGVAESDVFTTGLSELGMDSFTKGL------AVKTKSVTTGEQTTAGSARAKSNGASAVGSTLLGAGAAITDTTFRGESVTAGSIKGALQSSGVAGVADGQPVVQAAEPAADAGNTVVVVDKPATSETPVVVAQPAPAPEPYVPPVPAPEPYVPRPRPEPYTPPYSHKQSTGPYIPPYAHKQSPEPYTPPYVPKQS------------------------------------

>AQA26377.1_CP-43k-3-AQA26377.1

---------------AASAPGRRPRTSAMHCWILAVACLGAA------------------------------------------------------AAAPCINCGVRGAG-------------------------------------PPYGAVVVGPPPPEP----------------------------VLVEKHSLGTRTKADVSGTIISTGNAKAVVSGGSRATQLLPDQRGGAAARVKLYTQRNGLGSGSAASVGRSDVDAG---LNAASSQGDAITETRSTGEATTEADANGKGETLGEGSRTDANGGSSSATISDG----------------------------------------------------------------------------------------------------SSAGAAQTVGTAGQAPGIGTDAGSGAASHVE-------------------------------------------------------------------------------------------------------------------------------------------------------------------------------------------------------------------------------------------------------

>Aamph_140108_CL217

----------------------------MLPAAILLLSLGAA------------------------------------------------------LSAPAPGVTPP-----------------------------------------------VSPPLPPK----------------------------------RAATDADAVTVGTLKTVGTAIGKSSGGAVSLEQTADQGSKAGVKVDLYSQRAGATEGSAASTSATKVKCKPG-FSKGASVTGQQTSGASVGEATSTSDATGEAGTICDDTKSDVQGGASTSTISDGKAASEALNTAASETLTVPGGSSTVSNANSKTGGTSSGSAGTDAAGLASSRGIGD-GTTSRADSQTKISTTGDGRSEADQRSTGTGTTGRKRGALGAETSAQTTGTSSTVGGGSASKGESSAGGTANQGSNVAAESDS-NQKIGSTRTGSAAVDAKSGSAGVLG--AIKDKL------IGKSDAASGGSAESVGSAKTDFNTGGSAGHS-AGEGSGFAETSVGGQTRQTGAVEGSQII*--------------------------------------------------------------------------------------------------------------------------------------------

>Aamph_140108_CL217

----------------------------MLPAAILLLSLGAA------------------------------------------------------LSAPAPGVTPP----------------------------------------VSPPLPPVPPPLPPK----------------------------------RAATDADAVTVGTLKTAGTAIGKSSGGAVSLEQTADQGSKAGVKVDLYSQRAGATEGSAASTSATKVKCKPG-FSKGASVTGQQTSGASVGEATSTSDATGEAGTICDDTKSDVQGGASTSTISDGKAASEALNTAASETLTVPGGSSTVSNANSKTGGTSSGSAGTDAAGLASSRGIGD-GTTSRADSQTKISTTGDGRSEADQRSTGTGTTGRKRGALGAETSAQTTGTSSTVGGGSASKGESSAGGTANQGSNVAAESDS-NQKIGSTRTGSAAVDAKSGSAGVLG--AIKDKL------IGKSDAASGGSAESVGSAKTDFNTGGSAGHS-AGEGSGFAETSVGGQTRQTGAVEGSQII*--------------------------------------------------------------------------------------------------------------------------------------------

>Aamph_CP43k_homolog6_isoform_1

----------------------------MLPAAILLLSLGAA------------------------------------------------------LSAPAPGVTPP----------------------------------------VSPPLPPVPPPLPPK----------------------------------RAATDADAVTVGTLKTAGTAIGKSSGGAVSLEQTADQGSKAGVKVDLYSQRAGATEGSAASTSATKVKCKPG------------------------------------------------------------------------------------------------------------------------------------------------------------------------------------------------------------------------------------------------------------------------------------------------------------------------------------------------------------------------------------------------------------------------------------------------

>Aamph_CP43k_homolog6_isoform_2

----------------------------MLPAAILLLSLGAA------------------------------------------------------LSAPAPGVTPP-----------------------------------------------VSPPLPPK----------------------------------RAATDADAVTVGTLKTAGTAIGKSSGGAVSLEQTADQGSKAGVKVDLYSQRAGATEGSAASTSATKVKCKPG------------------------------------------------------------------------------------------------------------------------------------------------------------------------------------------------------------------------------------------------------------------------------------------------------------------------------------------------------------------------------------------------------------------------------------------------

>AQA26370.1_CP-43k-1-AQA26370.1

---SIKPPAALTRRSAAHPSADAPSDPTMLPAAILLLSLGAA----------------------------------------L--------------SAPAPGVTPP----------------------------------------VSPPLPPVPPPLPPK----------------------------------RAATDADAVTVGTLKTAGTAIGKSSGGAVSLEQTADQGSKAGVKVDLYSQRAGATEGSAASTSATKVKCKPG-FSKGASVTGQQTSGASVGEATSTSDATGEAGTICDDTKSDVQGGASTSTISDGKAASEALNTAASETLTVPGGSSTVSNANSKTGGTSSGSAGTDAAGKASSRGIGD-GTTSRADSQTKTSTTGDGRSEADQRSTGTGTTGRKRGALGAETSAQTTGSSATVGGGSDSKGESSAGGTANQGSNVAAESDS-NQKIRSTRTGSSAVDAKSGSAAALGAI--KDKL------VGKSDAASGGSAESVGSAKTDFNTGGSAGHS-AGEGSGFAETSVGGQTRQTGAVEGSQTSSASGSVTLKRPV--------------WPCRLPSKAPKDWLHGWVPGTKLVWHCVFPHKIPAKYSQLYKPKW----------------------------------------------------------------------

>Aamph_CP43k_homolog8_isoform_1

----------------------------MLAVVLLLLVAGGA------------------------------------------------------LCAPAPGESYP------------------------------------------TVPPTVPPTKPPK----------------------------------KDIAKTNAYTGGGVKTTGDSSSKLKGEVLSVVETEGSQIFNKVDLEVESQRDGGGGGAVTSNTVAKNKCQPD-FKKGVSVTGQEGKSGSIGEATTSTFAGGFANTRCNETRTSAQGQTKTGTSDFGQAGSESLNKASSKTVKLPEGISAVSSTKSRIEGESVGNSSTLALGDNSAEGGGT-GAKTVSTSGNQGSTDGDGQVKSVQGSAATATLARVAETLGAESSTGTIGAARSAGDKSSITGNAAATGEANEGGDVRANTKS-NQETKTDGDGASVTDTKSRAATL--RVAFTDDLTGAKKEIGKSAGSSAAESESVGSASAKLDTSSDIGFS-LDGGFGTGGTSIEGETR-NGTLVGSLRTSFDSKIASVPTRL---------KKPKWPCEKPSAPPHGWLDDWPITPNPVWPCELPLSPIDPFKPKASKSKDMHGF*----------------------------------------------------------------

>Aamph_CP43k_homolog8_isoform_2

----------------------------MLAVVLLLLVAGGA------------------------------------------------------LCAPAPGESYP------------------------------------------TVPPTVPPTKPPK----------------------------------KDIAKTNAYTGGGVKTTGDSSSKLKGEVLSVVETEGSQIFNKVDLEVESQRDGGGGGAVTSNTVAKNKCQPD--------------------------------------------------------------------------------------------------------------------------------------------FKKGVSISEQEGKSGSIGEATTSTEGRGIVSTKCNETFSAVQGATLTRTSN---------DGRAGSEALNKASSKT---------------VKLPEGISAVSN---------------------------------------------------------------------------------------------------------------------------------------------------------------------------------------------

>Ctest_CP43k_homolog1

----------------PVGPVHPVGPVHPVGPVHPVGPVGPV--------------------------------------------------------HPVYPVSPGQPPVYI----------------------------------PPPPPTTRQPPPPPG----------------------------VSLGQSPGGTGSDTKSSGGVVTTGHARSVVQGGGKAYEKGDDQQTASQSSVKLTSQKEGEAEAAAASTSGSKIACVPKLGSQGESQAATRAQGASVGQATTKSDATGNAGTVCESTKTDVDTGSSSATRGDGKADADSKTKSVAKTINTGSGSTTRVATGAEGRSQSAGGAGTNTAGRARGASIGK-SGTSKSGSQTQTETTGDGSAVGGQQGTATGTTGKGPNLAGADTSTTAVGESNTVGTGSSSGGETSSEATANQGSNVASESEA-NESVKATRTGAAKAAVGTGGSGVLGKD--GQDI------VGDSRAKSSVNQGSLGSGSTKTNGGSTAGHS-LGLGSGVAQSRSEATTAGTGTASGTHQAEAQGRQKPV---------------VQWPCSLPGKPK----AGWQFKDGDVWPCVFPVNFKAPFKPKVAFKG*---------------------------------------------------------------------

>Tform_CP43k_homolog1

--------------------------MRVVLLLLLLGVLGSL------------------------------------------------------DGAPVASPSKG-----------------------------------------------KKPPLPPP----------------------------------PKGTTTDTVSSGGVRTSGHAKAVVSGGSKAVENAQNQKTGSKAAVKLTSQATGQGEASAGSTSGTKVACVPPVGSKGTSQAGTRTSGASVGQATTQSDATGRATTVCKSTKTDADTGTSTATHGAGKAEADSKTKSTAKTINTGSGSSTQTTTGSESSGQSGGAAGTLGASRARAGGVGD-GTTAQSGSQVKTETTGDGAATGSQKGEAGGTTAVGVGAIGAETNADAKGESSSVGGGSSSAGETTAGATAGQGDNVAAESDA-NEGVQTTRTGVAAAKVGSKASAVLGKD--GKDI------VGDSQAKSSLDQISAGSGSTKTNAASTGAHN-LSGGSGAADTTIKAKTAGTGAAEGSHQARAQGRVTPAKKPK-----------HVWPCKFPTKAPKGWYKKYGTIKHPIWPCVLPIKPIKPFKPKPKKDPFKW*------------------------------------------------------------------

>Aamph_CP43k_homolog7

-----------------------------LFAVVLVAMLASL------------------------------------------------------QAAPAPGKAV------------------------------------------------------------------------------------------TVGTDSSTVATGSVTTGGNANGSAKGGSVTTVQADNQKSNTASKVNIYGQRYSKGEATAASSSGTGAKCRPN------------------------------------------------------------------------------------------------------------------------------------------------------------------GSKGTSVTGQQ-------------------------------------------------------------------------------------------------------------------------------------------------------------------------------------------------------------------------------------------------------------------

>Majax_CP43k_homolog1

----------------------------MLFAVVLVATLAGL----------------------------------------H--------------AAPAPSKSV----------------------------------------------------LPPK----------------------------------VVGTTSDTISTGSIVTGGNSKGTAKGGGVAAQQSDSQRSNTASKVNLYSQRYGKAKASSASSSGTGTKCGPY-GGKGTSVSGQQVSGASAGEATTESDATGGAATVCDSTSTEVEGGASSATRSDGTAASQTKNTATSETLIAPRGSSTVTNTQSNAEGTSSGSGGTDGAGRAGATGTGD-GVTSNTKSQTKISTTGDGRSAADQKAIGSASQGSGVGSAGVETDSRSTGSSQSVGGGSSSEGGSASKATANKGSVVAAESKT-NEEVGTTRTGSASSDVKAGSGSVVGKD--GSDI------VGDSQASTAVDAESAGSGFTKSNGQSTGGHS-IGSGSGVADSTIGGESAGTGFVKGSQESRAQGRTTVADKK------------PKWPCQLPSAPSKDWLKLLPNVKGTDWPCVVPGKAEEPYKAVALGSLF*--------------------------------------------------------------------

>Aamph_CP43k_homolog4

----------------------------MKFVVLLAAALSVA------------------------------------------------------SAVPTGFVRPNPVRTT-----------------------------------TTTTTTTTTTPAQPE----------------------------------GLATETKALAGGKLKTVGNALAQVAGGSNTVERQESQESAANARVQIDAQRAAIGQAKAASKGRSDVLSQAN----------------------------------------------------------------------------------------------------------------------------------SEQASSDTRAAARGVSVGEARTQADANGAAATRGRGTKTDGNSGISTATESDGASSGLSQALGTASRQTVGGVSESRADTISGT-------------------------------------------------------------KGQSRGSAGVAG-------------------------------------------------------------------------------------------------------------------------------------------------

>Aamph_CP43k_homolog5

----------------------------------------------------------------------------------------------------------------------------------------------------------------------------------------------------------------------------------------------------AGVAGAGRARSAAVQA---------------------------------------------------------------------------------------------------------------------NTTALSGSQTKVDVIGDGRATAAEAGGALGQSGGSASRRASRTEAANKGEVETRGDGAGAVGESSSKATATESGTSATESSA-NNRLAATRTGRAETSIFTEGGSTLGLDQ--------------------------------------------------------------------------------------------------------------------------------------------------------------------------------------------------------------------

>Chunt_CP43k_homolog2

-------------------------------------------------------------------------------------------------------------------------------------------------------------------------------------------------------------------------------------------------------EGKSRGRAAAIGAR--------------------------------------------------------------------------------------------------------------------GSTSISGSQTEGGVIGDGKVKSRQVAGAATRTDRGPLGKSAQSRAASEGEVQTAGPGASGGGESNAQARAG-DGVTNSKSQT-GQKLKSARDGAAAAKTETEAKSTLGLDAFGGTL------FGTTKTKA-------------------------------------------------------------------------------------------------------------------------------------------------------------------------------------------------

>Chunt_CP43k_homolog3

--------------------------------------------------------------------------------------------------------------------------------------------------------------------------------------------------------------------------------------------------IKSQEEGIGQASAASQGTSGVKCVPG-FSKGLSKGDAATEGASAGQATTEADANGRAATIC----------------------------------------------------------------------------------------------------------------KGGKTDANSGSSSATRGDG-KAKGVSRSVGQSGSAGDS-TNSNS-GAEVKGKAVGSAGT----------------------------------------------------------------------------------------------------------------------------------------------------------------------------------------------------------------------------------

>Cmala_CP43k_homolog1

----------------------------MNVWILLLAALGAV----------------------------------------N--------------AAPLVGPAVVLNP--------------------------------------------------------------------------------------PVATKSKAKAAGDLKTVGNAGASVSGGSITFETAADQKTDANARVSIKSQEEGIGQASAASQGTSGVKCVPG-FSKGLSKGDAATEGASAGQATTEADANGRAATICKGGKTDANSGSSSATRGDGKAKGVSRSVGQSGS----AGDSTNSNSGAEVKGKAVGSAGTEGKSRGRAAAIGARGSTSISGSQTEGGVIGDGKVKSRQVAGAATRTDRGPLGKSAQSRAASEGEVQTAGPGASGGGESNAQARAG-DGVTNSKSQT-GQKLKSARDGAAAAKTETEAKSTLGLDAFGGTL------FGTTKTKASGKQQSLGNANSDANAASTGVSK-AGGGGTKSDSAYKGKTAGDGAVSGSQQSQGEGVVKLGDNKASSILKKLG*-----------------------------------------------------------------------------------------------------------------------

>Ctest_CP43k_homolog3_isoform_1

-----------------------RGASRHIDAGVAPVRTTKS---------------------------------------------------QDRQLRPTPKAFPSGVGDGWYYNP------------------------------TTTTTTTTTTTTEKP----------------------------------GYGTRSKTEVGGKLKTVGNVIASVGGGSNAIETLADQQSKANSRVVINAQQAAIGEANAAAKAKSDIARQAD-AEQASSRGLAAAKGISVGQARTQSDANADAKTIGKGTKTDGSSGISTATEGDGSSSGISE-------------------------------------------------------------------------------------AIGASSRQTVGGVRQSQSGSLAATGESSSKSTASEKNSTASDSIA-QNKLKASRNGRGDATLFSESQSTLGLDQFTGDV------VG-------------------------------------------------------------------------------------------------------------------------------------------------------------------------------------------------------

>Ctest_CP43k_homolog3_isoform_2

-----------------------RGASRHIDAGVAPVRTTKS---------------------------------------------------QDRQLRPTPKAFPSGVGDGWYYNP------------------------------TTTTTTTTTTTTEKP----------------------------------GYGTRSKTEVGGKLKTVGNVIASVGGGSNAIETLADQQSKANSRVVINAQQAAIGEANAAAKAKSDIARQAD-AEQASSRGLAAAKGISVGQARTQSDANADAKTIGKGTKTDGSSGISTATEGDGSSSGISEAIGASSRQTVGGVRQSQSGSLAGTKGKSTGGAGVTGAARGRGASVNS-GTTSVSGSQTSVDVIGDGKAAAIEGGGATGESGSSSILRSSRTEAGSKGSVRTQGPGAGATGESSSKSTASEKNSTASDSIA-QNKLKASRNGRGDATLFSESQSTLGLDQFTGDV------VGAVGSLTSASQSTSGSANSAQNGASSATNA-LGGGAAAADSTFNAKSAGDGSVAGTIESRGQNRIKSQSSPVQLSLVERIRQQRLREQQQKGKGSVLPPKGQKLPKGQRPTQKPPTPTLPPSLPRGKND*----------------------------------------------------------------------

**File S4.** Alignment of all CP52k homologs.

>Chunt_CP52k_homolog1

--------------------------------------------------------------------------------------------------------------------------------------------------------------------------------------------------------------------------------------------------------------------------------------------------------------------------------------------------MFRILLLAAVLAVATPHGYHSLYTYLLPSDIAPALSLVRSRYVGISQPCAYSIIEHALTHPHNGFLGR----------------------INLSFPSL---RSQ-----------RLGYLRTLFS----ALPSQVFYT-----SYIDPVSAAL-GARGIH----------------------LPRYRLVD-----SLA----LSDYFLGLNAV------------------RPVSTGL-------FSQFYTGQLLGLTQ----------PFYNSFTFP--------------------------------------------------------------------------------------AYSTILGRLSSYGFPGQYFAPLVSVFTPYTG------LGHQLLPIFSGLGVRPSLPA--ITLPGVYNYYTQHNIAIPSFVARIRGVFNSLYQPVSYASVRSYGPGLGFITQD-----VF-EHDLIPALYSRYYAFPSGVRQNLFLHSTRARFAPLATAFFKDAFTGNLLTGHSTYSTQFVSQT----------LESFDTFCRGKSDSRYSGSFWNSGILGGIFNKLGKLG*-------------------------------------------------------------------

>Cmala_CP52k_homolog1

-------------------------------------------------------------------------------------------------------------------------------------------------------------------------------------RNRGAMLRVLVLTWLAAAAAGCRFKSCHPPHPHPHPLPHPRPHPLPRPYPRPFPRPH-------------PYPRPLPIT---------------------------------------------------------------------------------------------DPAWVILRGLLPRKHYRLALKLVLPRYPGLDRDGAAYVIRYLL--TH-RRLPI----FG-------R--------VKYHTVPA---LPL-----------RLAFIRKVWP----LLPP-VFYR----GKYHASLLSYL-RRLHFP----------------------HKYSYLIR-----PLS----LLDFHLACHFY------------------RPVSKLK-------FNKFFVRYIL---------------RFNGKVFPPLPRPS--------------------------------------------------------------------------------KLFDLFDRYPIKRYTHVFRHIHGHPL-KVRVTELPL-NEETLPLVLSRLGLPKFYRP-HTSLAGLLRVLKSASISQHRFVAHLRQLQ-GQLQRLIHRLTKQYSGL---------------RRDLLKLCALRYYAFPLRIRRNVHFHDVFRRYLS----------SIKVPTVYTKYTPTYVYRF----------IIRFERYVRTHALKKYSGKLWWSTG*-------------------------------------------------------------------------------

>Cmala_CP52k_homolog2

-----------------------------MLRSVLLAVLALAAAAHNDDDFFSSPVVVRHPSPEDLVLTDILAPAHRGKAIVLVQRRYPGLSRLAAAHGIRYLLSQHRLPIYQRIKMQGVPPLSDRLPYLQRLVAAMPPVFYTRYQNGLLHYLKKYGFSHGANALVAPLALLDMRLGSHLYRPVNSHYFNKFFARMILGSNGKYVGAIPPLQKLYTTIMRIKIKSLPDYFERITSPRPVRVVPFHGVVQPHGTKLIYILH-------------------------------------------NMGLPRLYKPRTSLAGLVAYLDRVGIPQTRFVDRVRKLRGDIQRLIHNAKKQYDGVRSDLHMLAALRYYQFPKTTRVNIRFPDAFSRYLGQSQ--SPSRYSPEYVSAFLRGFSDYVR--------RYRPQKPAH--GDY-----------RVHHLEKVLSSL--GLPR-LYRP----RESLSGLEEYL-QHAQINHDNFVDRIRKM---RQRIKGIIHNIRKHYD-----GVR----ADLMQLSALRYYQ----------------LPEDTRS--------DISFDQAFS--------------KYLSQRKNPG-------------------------------------------------------------------------------------QYSRHYVRDFLQGFGSFIRQRRRSAP-------ITD-EVHGLARALRRLGLPRFSQP-DASLGGLIVFLRGQRIPRHQFVARLATLS-SNVRSLISGLTPSCRGL---------------RSDLLKLSAMRYYGLPKRIRVNVRFPDAFRGYLR----------RYKIPR---HWNSDAAYQY----------LTGFDKFVRTHKLRKYSGKMWWSW*--------------------------------------------------------------------------------

>Cmala_CP52k_homolog3

------------------------------------------------------------------------------------------------------------------------------------------------------------------------------------------------------------------------------------------------------------------------------------------------------------------------------------------------------------------------------------------------REAALDVLRLLLARPH-RRLLR-------------A--------VRLQTVPV---LEQ-----------RLAFLRAVFG----ALPP-VFYR----ANYRRALVTYV-RRLGVH----------------------VTPQALVE-----PLA----LIDARLAASFV------------------TPVSVPT-------FCNFVGSQILHA------------VGGRRVSLPPL-H----------------------------------------------------------------------------------TLYSRLVSLRLNPVPVGFQRVTLRVG---PSSLTLT-AVRTLRPVLARAGLSQLLHP-ATVIGGLADYLGAQRIPVSRFAHCVSLLRIPSVAAVVSRLQVRAVVRRLHFLPA-----PLVRRDILPLLALRFFTLPVQLTRRLRFARIVPGFLA----------SLRVKR-AVVFDRSFVSGL----------LTSFDSYLHSHVKGFRGRLWTPQFYRVSGGVKY*-----------------------------------------------------------------------

>Cmite_CP52k_homolog1

---------------------------------------------------------------------------------------------------------------------------------------------------------------------------------------------GTPAAEMLRLVLVAALVARSLATFGPGYGFHDVLGGLGSAGVVVD---------------------------------------------------------------------------------------------GGLVSAGLHVGSGLGSVVVPGVGGVVGGGVLDLGGLLSAGDVGQAIELVQTRYSGISSPAAVQIIRLAVSQPPAAILPR----------------------IHFRTVPS---LGR-----------RLVYLRRLIA----ALPPPIAL-----QSYLPSVEAQL-GALGLH----------------------VPRAALVD-----PLW----LTDFRVAGSLL------------------APISPPV-------FSKFFVNRVLNVH-----------RGLPSHAVPVIPSLH--------------------------------------------------------------------------------VIDSRLGGLDLGGLRTVFRSIVLAGGGLSG-------AGEAIAPVISRIGLPRLLQP-AVTLGGLADYLHVQHVPVRAFVRHIKVYRAPQITRLVRPIQIQPIVGRLSFVSA-----PLIRRDILPTLALRYLSLPPILQSSLNFDRIVPGYFG----------SSLIPG-GHSLSRGFVSGL----------LNGFDSYIHGLLNDYDGSLWGEGMCYGK*----------------------------------------------------------------------------

>Cmite_CP52k_homolog2

---------------------------------------------------------------------------------------------------------------------------------------------------------------------------------------------------MFRAFLVVALAAVAAAHGGHHPGPIYVRP---------------------------------------------------------------------------------------------------------------------------------NPLTPILGGLLNPSDYGVAIRLVQRRYSNLQSVAAAHILRYIL--RP-QRLPI----YG-------R--------IKYRTVPI---ISR-----------RVAFLRSYFG----ALPG-VFYH----PTYYGGLRSYL-SRYHFK----------------------YNPSSLLA-----PLA----FCDIRLASHLY------------------RPVSVSS-------FNGFFGRHIL------GFNGK---VFR---GFPSL-G----------------------------------------------------------------------------------RLDTILGGISLSAYPRIFQSIVSVSV--------SY-PGSSLLPIWSGIGLPALSQP-QATLGGLYSYLASANIRTVDFIRHCRTIRLPNINRLIHSLLKLYSGVY--------------RTDLLPLLALRYYSFPTGVRRNLNFVRSTRGFLR----------SIKIPG---RLSSRYVRGF----------LGRYSKYIRHHYLKKYSGKLWW*----------------------------------------------------------------------------------

>Cmite_CP52k_homolog3

LGIGQIPAVSGRLEFLRLYFSRLPVVVYNQGISNDLASFLAGLKIRTSPSDLLAPLAISDIYLASNLVRPTSPSLFNRFFLRNILGIKGRVAGRPSLPSIDVISSNLASLPLTSGFSGTLQPIAIGGSSQQQIIISHSQPVVGTVKTAVVSPVVKILGGLLSPRDIVPATRLVQTRYPGVSSLAAAQIISYILRSKQLPVFGQLGIGQIPAVSGRLEFLRLYFSRLPVVVYNQGISNDLASFLAGLKIRTSPSDLLAPLALSDIYLASNLVRPTSLSLFNRFFLRNILGIKGRVVGRPSLPSIDVISSNLASLPLTSGFSGILQPITIGGSSHQQIIISHSQPVVGTVKTAVVSPVVKILGGLLSPRDIVPATRLVQTRYPGVSSLAAAQIISYILRS---QQLPIFGK-------------------IKLKKVPA---VAA-----------RLPFLRSVFR----ALPAPVLVS----RVYSGGLVSHL-QRHGLR----------------------TSAAALTA-----PLA----IIDIHLACQLL------------------QPVSLSA-------FRDFFIGQIL---------GISGKIIGSQYSLPSVG-----------------------------------------------------------------------------------RLNTILSRLQYSSFVPAVFQRIGY-------------AGSSLLPIWSRIGLPALSQP-QATLGGLRSYLASTNIPRPTFVQYISSLKPHSINALIHPLQKLYSGAY--------------RTDLLPLLALRYYSLPLRIQRNVNFGAVTRAYLS----------SIKVPQ---RLSASYVRGF----------LRGYSDYIRHRVPKKYTGRLWRW*---------------------------------------------------------------------------------

>Ctest_CP52k_homolog6

----------------------------------------------------------------------------------------------------------------------------------------------------------------------------------------------------------------------------------------------------------------------------------------------------------------------------------------------------------------------------------------------------------------------------------------------------------------------------LFP----ALPP-VFYP----SQYQGSLASYL-RELKFP----------------------YSKEELVD-----PLS----LIDIRLVSYKY------------------RTLSVKT-------FNEFFVINIL------KYDGE---RFQ---RIPPL-Q----------------------------------------------------------------------------------TLDNMLNASLFSSH-PTVFETTRPALTTLSSDTVTQ-PSSSLQSLVFSIGLPAFSRP-RASLGGLEQFLRSADIPYSQFLSRASQVG-RRIRAAFDTMKSLYTGV---------------QSDLLKLAALQYFAMSASTKSHVEFSDAFASYIE----------ETQIPD---SYSADYASRF----------LTGFEDHVHQLHLKNHVHDHGHVHDHAHHHSHGHVPSPVSSHSYGPR*---------------------------------------------------------

>Lanat_CP52k_homolog1

-----------------------------------------------------------------------------------------------------------------------------------------------------------------------------------------------------------------------------------------------------------------------------------------------------------------------------------------------------------------------------------------VTLPGFYNYLTRQQIA----------------------------------------------IPS-----------FVSQFRSSFG----NLYRPISYS---TVRRYGSHLGFI-NSRIFA--------------QDLQPLLALRYYSFPS-----DLR-----SNIYLQNVRANF----------------SPLVSSF-------FRNIFSGSLL--------------SSYR-------------------------------------------------------------------------------------------TYDSRFVSAALNSFDKYCR------------------------------GMSHGRYRGPYWVGGLLKALKHKYKKG*-----------------------------------------------------------------------------------------------------------------------------------------------------------------------------------------------------------------------------

>Mlong_CP52k_homolog6

----------------------------------------------------------------------------------------------------------------------------------------------------------------------------------------------------------------------------------------------------------------------------------------------------------------------------------------------------------------------------------------------MFHAAFVFVLVAV--------------------------------------------AAG-----------QQTSISSAVG----QITPLSSFS--DSTTSLEGLESFV-KQSTVSESQFIANIQGS---STQLQIFLNAFKSEYS-----GTL----SDLAQISVLGY----------------------IGT-------VTNWLPPLLFS-------------GFFQQTEVPA-------------------------------------------------------------------------------------EYTYSNAARYIKDFIDNAQS*-------------------------------------------------------------------------------------------------------------------------------------------------------------------------------------------------------------------------------------------------------------------------------------

>Cmite_CP52k_homolog4

------------------------------------------------------------------------------------------------------------------------------------------------------------------------------------------------MADVLRALLLTALAAAAAAHFGGGDGGGTVVVRVAG-----------------------------------------------------------------------------------------------------------------------------HGAGALPDGLLAPTQVGPAFQLVQARYPGISSLAAGNVISFLLSASA-QQLPI----FS-------R--------LALGPIPA---ISG-----------RLAYLTSVFS----RLPAPVVFG----VRFSGALASHL-AGHGLR----------------------VPAAALLS-----PLA----VTDIRLVANLL------------------KPVSPQV-------FGGFFRSKIL-------------------------------------------------------------------------------------------------------------GLGGKIVGTPTLPSIDVI----------------------------------------------------------------------------------------------------------------------------------------------------------------------------------------------------------------------------------------------------------------------------------------

>Cmite_CP52k_homolog5

---------------------------------------------------------------------------------------------------------------------------------------------------------------------------------------------------------------------------------------------------------------------------------------------------------------------------------------------------------------------------------------------------------------------------------------------------------------------------------------------------LNGLHGYLGSHLGLQ----------------------IPVGALVD-----PLS----LADIYFSGHLV------------------YPVSVAA-------FNKFFVSHIWGGHGGSVYDHVL--GIGGGVGIRPGAG----------------------------------------------------------------------------------LYGIPYINGLISRIRKISLTPIPAGFRQIVLPGSSLVAGGSLLPTLSKIGLPVPVQP-TVTIPGLVDYLVGQRVPVQRFISRVRVIR-IHFRRLIRRIKLRRIVRFLPYVSP-----TVIRQDLLPLL--------------------------------------------------------------------------------------------------------------------------------------------------------------------------

>Wmill_CP52k_homolog1_isoform_1

------------------------------------------------------------------------------------------------------------------------------------------------------------------------------------------------------------------------------------------------------------------------------------------------------------------------------------------------------------------------------------------------MLRVVLLVTLMVVASA-KRDER----------------------------------AK-----------QKEQLRTALKSI--EMPD-FIRP----ERALAGLDFFM-TDNGITYQSFVSRIKKW---RYQIKELYDDHEANHY-----GWW----KDFLQLAAVEYYYY---------------RPTTYKA--------KISFIDAFK--------------QYSE--TLRPKKK----------------------------------------------------------------------------------GEIFLYSAANVKLF-----------------------------------------------MNGFIEFINSKQTGR*-----------------------------------------------------------------------------------------------------------------------------------------------------------------------------------------------------------------------------

>BAL22342.1_Mrosa_52k

---------------------------------------------------MLLRPVLLLAALAALAAATGSRPYFPVSSLKPVLSGIGLPAFYKPDYALSGLVGYLNTRPKIVTQAQFTARIQKYAPVIKRIVLPMRRKYSGILGDLIQVAVIRYYGCEPVIGSSIHLDRIFGEYIKRQSMPSPYKYNAKYVTGFIRGFMGYMHKNYKPSQ---------------LVVPVVKPTYP-------------GYGLLTVLQ-------------------------------------------SVGLPALTNPRMSLGGVVAYLQLANIQQAVFISRIRSQRKAIRRLVSKYRSRYSGVQLDLLCLAALRYYGVPRTARYVVDFDYALEHSLKSTAIV---HYNPSYVRTFL-------S--------RFSTKLVT---LPY-----------PGYDMITIFRGF--GLPK-LYQP----RYTLGGLVSYL-KVAKISQPTFIGQIKKY---AKKIKKFIRKYKKKYS-----GYR----SDLLQLAAIRYCL----------------YPRSYPI------KFSTVFQRALS--------------SYS--------------------------------------------------------------------------------------------TYSVSSVSSFLGLFTQYLKKP-------------AY-VGYNLKPVLLQAGLPKLSMP-QYSLSGLMSYIHGNKYSDSSLIGLIRVYG-PKIKRIVHQYKSRYSGI---------------QADLLQLCAIRYYSLPVVFRSSYSFGTIFQQYLG----------SQNLK----VYNASTVKRF----------INGFVSYIRKRQSNKYSGPMWVCRRC-------------------------------------------------------------------------------

>AKZ20820.1_CP52k

-----------------------------------------------------------------------------------------------------------------------------------------MKQYKGVLADLLQLAVVRYYGCEPVINKKVPLSTYFGRYVKKTKIPSPYRYNTKYVRTFLDGFKVYLRRHLQRPQKISIPSLPPKDIFPIFPVRPPKPSYP-------------GYEILATLK-------------------------------------------SLGLPPLVNPRASLGGIVAYLKVVGVSKTTFISRIQVYRQAIIKVVLKYKKIYTGYRLDLLQVAALRYYGLKKNTKYEVSFGYALRKILKPSI--KI-KISVTYVRQFL-------V--------RFSRVLVA---VPY-----------PGYSLLPVLRGI--GLPK-LYRP----KYALGGIVAYL-KIKKISQVTFIARIKKY---RLKIKKLVLKYKRQYK-----GYQ----LDLVQIAALRYYCISKKSKYSVSFGYALKKTIKLSIRQKYSIKYARIFLERFSKVLVTLPYPGYNLIRVFRGIGLPKLYRPKYSVGGLVAYLKVVKIKQVTFISQINKYSTSIRQLVLKYKKQYSGFRVDLLQLAALRYYGIKQDYSVKFIVAFRKALVPSFKISTRSIKAFLVRFSKYLLKPAYISP-AYKPRPATY-PGYQLKPLLTTVGLPKLTKP-QYSLPGLVGYLQSNKYPLPSLVGRIQKYG-PKIRSTVYRYRPNYSGV---------------LTDLLQLCAIRYYSLPPVIRSNVGFGSTFQRYIR----------SQKLSR---QYSPAFVKKF----------LSGFIKYIRKNKFQKYSGKWW------------------------------------------------------------------------------------

>Ctest_CP52k_homolog1

-------------------------------------------------------------------------------------------------------------------------------------------------------------------------------------------------------------------------------------------------------------------------------------------------------------------------------------------------------------------------------ASQEAIKLVTNRYNGLSKIAAAYILRFLL--TN-KQLPI----FN-------G--------MKYRSVPV---LAS-----------RLKFLEELFL----ELPP-VFYS----GKYQRNLISYL-KALHLP----------------------HDLDKLID-----PMA----LVDIRLASYQY------------------QTPRVED-------FAHFLVVYAL------KYNGN---KFE---DVQPQ-E----------------------------------------------------------------------------------TIDRLLSQKLTEHF-P------------------------------------------------------------------------------------------------------------------------------------------------------------------------------------------------------------------------------------------------------------------------------------------

>Ctest_CP52k_homolog2

-----------------------------------------------------------------------------------------------------------------------------------------------------------------------------------------------------------------------------------------------------------------------------------------------------------------------------------------------------------------------------SELYEALNSNMMSRQPAVFKPTKRAAAPRPRATEKPQAAPIII-------------------------------IPD-----------PMHEVLH-------GLLP-VSYY---------GVANDL-VVSRYP-----------------------------------GLS----RD------------------------------------------GAAYVLHFL-------------------------------------------------------------------------------------------------------------LTSRQLPVFTLIKYQT------------------------------------------------------------------------------------------------------------------------------------------------------------------------------------------------------------------------------------------------------------------------------------------

>Ctest_CP52k_homolog3

------------------------------------------------------------------------------------------------------------------------------------------------------------------------------------------------------------------------------------------------------------------------------------------------------------------------------------------------------------------------------------------RYPGLSRRAAAYILRFLL--TK-KQLPFNR-----------H--------MKRHTIPA---LAG-----------RLEFLQLLFR----ALPA-VFYS----GRYQSSLTARL-KQLNIA----------------------YDQNKLIT-----SLS----LIDIRLAVNRR------------------KPVSPQT-------FNQFFVQHILKA------------GGENNGGIRPL-N----------------------------------------------------------------------------------ELYDALNSTMMSRH-PTVFKPT------------------------KRAAAPRPQAT-EKPQAAPIIIIPDP----------------------------------------------------------------------------------------------------------------------------------------------------------------------------------------------------------------------------------

>Ctest_CP52k_homolog4

-------------------------------------------------------------------------------------------------------------------------------------------------------------------------------------------------------------------------------------------------------------------------------------------------------------------------------------------------------------------------------------------------MAAGYILRFLLTS---KQLPV----FG-------R--------IKYNGVPI---LSR-----------RLNFLKLLFP----QLPP-VFYS----GKYRKSLISYL-RTIKAP----------------------YDMHKLID-----PMG----LVDIRLACNQY------------------QPPSIED-------CSKFVLKNVLKH------------HKDNFEGVRPL------------------------------------------------------------------------------------RTIDTVLNQSLATFFPTVTKPSHPV------------VQRRVTNADLGISQHPKQAP-IIVKPDPVQVLLRGLLPT------------------------------------------------------------------------------------------------------------------------------------------------------------------------------------------------------------------------------

>Ctest_CP52k_homolog5

---------------------------------------------------------------------------------------------------------------------------------------------------------------------------------------------------------------------------------------------------------------------------------------------------------------------------------------------------------------------YRKNELLAPLSLTDIRLATYIYKPRSIQNFSRFFTRFILKIRG-RPFPE----FP---------------------------PLN-----------KLDTILSSVRIT--GYPA-VFRPLQTSQRYTTPVTRYP-RRPQLV--------------PANKPDLVYVMLHSLL-----PIT--TYETAIKLVSIRY------------------PGISRTA--------AAYVIRFLL------------------TNQLPVYRGM---------------------------------------------------------------------------------QYDVVPALSGRLGFLQLLFPALP-----------------------------------------------------------------------------------------------------------------------------------------------------------------------------------------------------------------------------------------------------------------------------------

>Ctest_CP52k_homolog7

--------------------------------------------------------------------------------------------------------------------------------------------YSVKYQKNLTSYLRTVNFYDNINKLIDHMSLVDIRLVCQQYQTPSVEEFGRFITKYALQYRQREFEGIRPLKTIDTVLNRNLATVLPTVFRPSQPA--------------------------------------------------------------------------------------------VQKQVTVPVLGIPRHPKPASIIVKPDPVHAILNGLLPTTAYPAAIKLVTDRYTGLSRMAAAYILRFLITN---KQLPIYRQ-------------------MKYNAVPV---SSG-----------RLQFLRQLFE----ALPP-VFYF----SQYQKKLTAYL-EALNFP----------------------YAVTMLIV-----PLS----FIDIRLASYQY------------------QP---------------------------------------------------------------------------------------------------------------------------------------------------------------------------------------------------------------------------------------------------------------------------------------------------------------------------------------------------------------------------------------------------------------------------------------------

>Ctest_CP52k_homolog8

----------------------------------------------------------------------------------------------------------------------------------------------------------------------------------------------------------------------------------------------------------------------------------------------------------------------------------------------------------------------------------------------------------------------------------------------------------------------------LFQ----VLPP-VFYV----SPYRESLASYL-ETLDFP----------------------YDVDRLID-----HLS----VVDIRLASYQY------------------QIPSTQA-------FGQFVIKQIF------KYSGN---EFR---AIRPV------------------------------------------------------------------------------------GAVDVLLNQNLATFFPAVLTPLP-------------------RPVQKRATVPVSHKP-QYPKSAPVI---------------------------------------------------------------------------------------------------------------------------------------------------------------------------------------------------------------------------------------

>Ctest_CP52k_homolog9

------------------------------------------------------------------------------------------------------------------------------------------------------------------------------------------------------EHFPTVFRPNQPSAQKMVTVAVLGIPQHPKPAPIIVKP---------------------------------------------------------------------------------------------------------------------DPVYAMLNGLLPTTAYHAAIKLVTDRYHGLSRIAAAYILRFLL--TN-KQLPV----FG-------R--------IKYNEAPT---LPG-----------RLDFLKQLFH----ELPP-VFYS----DKYHNKLVYYL-RTLKVP----------------------YNLDKLID-----PMG----LVDIRLACYQY------------------QPPTVED-------VGQFLIKYVL-------------------------------------------------------------------------------------------------------------KYNGVKGVRLLRT---------------------------------------------------------------------------------------------------------------------------------------------------------------------------------------------------------------------------------------------------------------------------------------------

>Ctest_CP52k_homolog10_isoform_2

-------------------------------------------------------------------------------------------------------------------------------------------------------------------------------------------------------------------------------------------------------------------------------------------------------------------------------------------------------------------------------------------------MAAAYILRFLLTN---KQLPVYRH-------------------TKYGAVPA---MPG-----------RLQFLQHLFR----ALPP-VFYT----GQYQRNLLTYL-KALSFP----------------------HSKNKLIM-----PLS----LLDIRLAAGGH------------------YPVDMDS-------FNQFFIRKLM---------------KFNGKTFQALPSAD--------------------------------------------------------------------------------QLSKALSMKLIADHPRVFGAV-------------------------QRAGQPAAAEAVTTSIGGLDQFLHCTRVARSQMSSSVSWVG-GQTPIVFTTMKDDYTGG---------------YSELPQTTTAPQRTQPTAAQGDVKVPTN---YFG----------VIGNTKMSDISTPQPQSRF----------QDAFEGYF*------------------------------------------------------------------------------------------------

>Ctest_CP52k_homolog10_isoform_3

-----------------------------------------------------------------------------------------------------------------------------MKFLQLLFKSLPPVFYHGQFQANMLAHFKRLNVPYNKDEIMIPLSFIDNRLISYQYQPLNAQTFVRFFVKYVLKYGGEDLQDISTISKFDKIINLALIVRYPTVFRPIEP----------------ATQRPATVP-------------------------------------------GFSRPRHLKPIPNMGRP---------------------------------DSVHVILKGLLPANSYGAAIRLVVNRYPGLSRTGAAYVIRFLLTN---KQLPVYRH-------------------TKYGAVPA---MPG-----------RLQFLQHLFR----ALPP-VFYT----GQYQRNLLTYL-KALSFP----------------------HSKNKLIM-----PLS----LLDIRLAAGGH------------------YPVDMDS-------FNQFFIRKLM---------------KFNGKTFQALPSAD--------------------------------------------------------------------------------QLSKALSMKLIADHPRVFGAV-------------------------QRAGQPAAAEAVTTSIGGLDQFLHCTRVARSQMSSSVSRAG-GHTPTDFTTMKDDYTGG---------------YSELSHTTTAPLRTQPTAVQGDVKVPTN---YFG----------VIGNTKMSDMSTPQPQSRF----------QDAFEGYF*------------------------------------------------------------------------------------------------

>Ctest_CP52k_homolog10_isoform_1

------------------------------------------------------------------------------------------------------------------------------------------------------------------------------------------------------------------------------------------------------------------------------------------------------------------------------------------------------------------------QCLTTSDPKSHSRCRESTRDPTPCCCSMKYICPPLQALPSAGQLSR----------------------ALSMKLIT-----------------DHPSVFGAVQRA--GQPAAAEAV----TTSIGGLDQFL-HCTRVARSQMSSSVSRA---GGHTPTDFTTMKDDYT----GGYS---ELSHTTTAPLRT------------------QPTAVQG--------DVKVPTNYF--------------GVIGNTKMSD-------------------------------------------------------------------------------------MSTPQPQSRFQDAFEGYF*---------------------------------------------------------------------------------------------------------------------------------------------------------------------------------------------------------------------------------------------------------------------------------------

>Tform_CP52k_homolog1_isoform_1

-------------------------------------------------------------------------------------------------------------------------------------------------------------------------------------------------------------------------------------------------------------------------------------------------------------------------------------------------------------------------------------------------MGAAYVLRFLL--TN-RQLPI----YS-------R--------IKYRLVPV---LSG-----------RLEFLQLLFR----ALPP-VFYS----GQYQSGLFAYL-RTLNYP----------------------YSQDSLIA-----PLC----LTDIRLATYQY-----------------------------------------------------------------------------------------------------------------------------------------------------------------------------------------------------------------------------------------------------------------------------------------------------------------------------------------------------------------------------------------------------------------------------------------------------------------

>Tform_CP52k_homolog1_isoform_2

-------------------------------------------------------------------------------------------------------------------------------------------------------------------------------------------------------------------------------------------------------------------------------------------------------------------------------------------------------------------------------------------------MGAAYVLRFLL--TN-RQLPI----YS-------R--------IKYQAVPV---LSG-----------RLEFLQLLFR----ALPP-VFYS----GQYQSDLFAYL-RTLNFP----------------------YSQDKLIV-----PLS----LTDIRLAAYQY------------------QPLNVQR-------FNQFFIRNVL------KFNGK---VFQ---DIPPL-N----------------------------------------------------------------------------------KLDRILNVDLITG---------------------------------------------------------------------------------------------------------------------------------------------------------------------------------------------------------------------------------------------------------------------------------------------

>Aamph_CP52k_homolog1

-------------------------------------------------------------------------------------------------------------------------------------------------------------------------------------------------------------------------VTYVRQFLVRFSRVLVAVPYP-------------GYSLLPVLR-------------------------------------------GIGLPKLYRPKYTLGGIVAYLKIKKISQVAFIARIKKYRLKIKKLVLKYKKIYTGYRLDLLQVAALRYYGLKKNTKYEVSFGYALRKILKPSI--KI-KISVTYVRQFL-------V--------RFSRVLVA---VPY-----------PGYSLLPVLRGI--GLPK-LYRP----KYALGGIVAYL-KIKKISQVTFIARIKKY---RLKIKKLVLKYKRQYK-----GYQ----LDLVQIAALRYYC----------------ISKKSKY--------SVSFGYALK--------------KTI---KLSIR-Q----------------------------------------------------------------------------------KYSIKYARIFLERFSKVL-------------VTLPY-PGYNLIRVFRGIGLPKLYRP-KYSVGGLVAYLKVVKIKQVTFISQINKYS-TSIRQLVLKHKKQYSGF---------------RVDLLQLA--------------------------------------------------------------------------------------------------------------------------------------------------------------------------

>Aamph_CP52k_homolog2

--------------------------------------------------------MFRPVLLLALAAAAAAVGTYPGFTLKPVFSEIGLPSLYKPQYSLGGLYGYLQQ--ADIPKTQFVSRVRKYGPYISRTVLPLTKQYKGVLADLLQLAVVRYYGCEPVINKKVPLSTYFGRYVKKTKIPSPYRYNTKYVRTFLDGFKVYLRRHLQRPQKISIPSLPPKDIFPIFPVRPPKPSYP-------------GYEILATLK-------------------------------------------SLGLPPLVNPRASLGGIVAYLKVVGVSKTTFISRIQVYRQAIIKVVLKYKKIYTGYRLDLLQVAALRYYGLKKNTKYEVSFGYALRKILKPSIRI---KYSVTYVRQFL-------V--------RFSRVLVA---VPY-----------PGYSLLPVLRGI--GLPK-LYRP----KYTLGGIVAYL-KIKKISQVTFIARIKKY---RLKIKKLVLKYKRQYK-----GYQ----LDLVQIAALRYYCISKKSKYSVSFGYALKKTIKLSIRQKYSIKYARIFLERFSKVLVTLPYPGYNLIRVFRGIGLPKLYRPKYSVGGLVAYLKVVKIKQVTFISQINKYSTSIRQLVLKHKKQYSGFRVDLLQLAALRYYGIKQDYSVKFIVAFRKALVPSFKISTRSIKAFLVRFSKYLLKPVYISP-VYKPRPATY-PGYQLKPLLTTVGLPKLTKP-QYSLPGLVGYLQSNKYPLPSLVGRIQKYG-PKIRSTVYRYRPNYSGV---------------LTDLLQLCAIRYYSLPLVIRSNVGFGSTFQRYIR----------SQKLSR---QYSPALVKKF----------LSGFIKYIRKNNFQKYSGKWW*-----------------------------------------------------------------------------------

>Ctest_CP52k_homolog11_isoform_1

--------------------------------------------------------------------------------------------------------------------------------------------------------------------------------------------------MLCTVALITVLTATAASQKSYAPGF--------------------------------DRRLERVMA-------------------------------------------DIRLPKLSRPYTSLNGLLEYLQQTHVPEFKFLSRIQLHGADISHIVHSNKGQYTGHRADLWQLSALFFYGVPKASRTVSFEAAFKRSFIEPVL-----RYAKYSVVEFL-------G--------RFSQALVT---PTY-----------PAHKLLAVLRRV--GLLH-FHNT----ANAVGGTVAYL-ELANVNYAAFLRRISTY---RSIIVQLVHKHRHSAR-----GYQ----TDLLQLAAMRYFAVPD-------------SPPHIVS-------FPEAFEKALQ--------------------VYEPT------------------------------------------------------------------------------------KYTVRYATQFLVRFNHTLTAPPY--------------PGYKLTPILKKMGLPALKDV-ETSLGGVVVYLKAAKIRMATFINFIQAFS-IGIKKIVSKYIKRYSGY---------------QADLLQLAVMRHFIVYETDHYAVSFRVAFRQALK----------ANPIRQYSVSYVRQFLVSFSR--------IHITQWYPGVGLEPHLKIIVPSGLDYAKITLMGLDSYLLVTQNKVGEVAQRLASWSLGPRVPGSRPGWSSADWTRMG*---------------------------

>Ctest_CP52k_homolog11_isoform_2

0

>Ctest_CP52k_homolog12

-----------------------------------------------------------------------------------------------------------------------------------------------------------------------------------------------------------------------------------------------------------------------------------------------------------------------------------------------------------------KNYSGNQYDLLQVAALRYYGISETSEYPISFIAAFKRALGGTKDIPEDQYNASYVKAFL-------V--------RFSAFLMAPVPPSG-----------PGHEVQAFLQLT--KLPE-LHKS----EVSLAGLVEYL-KTINISPAAFASRVKTY---SSEIKAMVTKLEGQYA-----GAE----ADLLQLCVLRYFS----------------FPPAVQQ--------RVQFSSVFR--------------RFL---GMQSM-K----------------------------------------------------------------------------------NYSSNYVSQFLTDFVAHVRKHHLKDS---------------------------------KKFAGVLW*--------------------------------------------------------------------------------------------------------------------------------------------------------------------------------------------------------------------------------------

>Ctest_CP52k_homolog13

------------------------------------------------------------------------------------------------------------------------------------------------------------------------------------------------------------------------------------------------------------------------------------------------------------------------------------------------------------------------------------------------------------------------------------------------ILIT---PPY-----------PGYKMAPLLQRI--GLPG-LKYP----QYTLSGIVSYL-KIVKISQTTFISSIRAH---ASIIKKTVLEHRKHFT-----GYR----LDLLQVAVLRY------------------YGMSQTS------QYAVSFSVAFR--------------QALAVHTFH--------------------------------------------------------------------------------------QYNVAYVSQFLVHFSKILVTPPY--------------PGYKMAPLLQRIGLPGLTYP-RYTLSGIV----------------------------------------------------------------------------------------------------------------------------------------------------------------------------------------------------------------------------------------

>Ctest_CP52k_homolog14_isoform_1

-----------------------------------------------------------------------------------------------------------------------------------------------------------------------------------------------------------------------------------------------------------------------------------------------------------------------------------------------------------------------------------------------HQYSVAYVSQFLV--------------------------------HFSKILIT---PPY-----------PGYKMAPLLQRI--GLPG-LKYP----QYTLSGIVSYL-KVVKISQTTFISSIQSH---AAIIKKTVLQHQKHFT-----GYR----LDLLQVAVLRYYG----------------MSQTSQY--------AVSFNVAFR--------------QALEVHTLH--------------------------------------------------------------------------------------QYSVAYVSQFLVHFSKILITPPY--------------PGYKMAPILQRIGLPGLKYP-QYTLSGIVSYLKVVKISQTIFISNIQAHS-AIIKKTVLKHLKHFTGYR------------------------------------------------------------------------------------------------------------------------------------------------------------------------------------------------

>Ctest_CP52k_homolog14_isoform_2

--------------------------------------------------------------------------------------------------------------------------------------------------------------------------------------------------------------------------------------------------------------------------------------------------------------------------------------------------------------------TGYRLDLLQVAVLRYYGMSQTSQYA--VSFSVAFRQALEVHTLH-QYSVTYVSQFL-------V--------HFSKILIT---PPY-----------PGYKMAPLLQRI--GLPG-LKYP----QYTLSGIVSYL-KIVKISQTTFISSIQSH---AASIKTMVYKHRKHYT-----GYR----LDLLQVAVLRYY-------------------------------------------------------------GMSQI-S----------------------------------------------------------------------------------RYTVSFSVAFTQALKVSVYHQYT-----------------------------------------------------------------------------------------------------------------------------------------------------------------------------------------------------------------------------------------------------------------------------------

>Ctest_CP52k_homolog14_isoform_3

-----------------------------------------------MLRTVLVAALTVTAIVSAGNGVKQASHPYHGGNLRHVLASMGLPEFAQPNDSLNGLMSYLQQVH--VSRAEFLGRVQMYAPFISGIVHSLKKRYRGVAGDLLQLAALRHYGCEPQVGNHLWLGQHFQRYLQRMKMPHSKNYNIKFVISFLNGLKGYMSKLRQPPKVDIAP-------------VPTPAPYP-------------GYELLTVLK-------------------------------------------SAGLPGFHNPRFSLGGLISYMKITKISHTTFIVGIRAHISSIKQMIIKYRTQYTGYKADFLQLAALRYYGMPRTSPYL--VSFITAFKKSLAGPSQF-SNGIAFVSQFL-------I--------KFSKTLIT---PPY-----------PGYKMAPLLQRI--GLPG-LKYP----QYTLSGIVSYL-KVVRISQTTFISNIQTH---ADSIKKMVLKYRKHFT-----GYR----LDLLQVAVLRYY-G---------------MSQTSQY--------AVSFSVAFR--------------QAL---KVHTL-D----------------------------------------------------------------------------------QYSVTYVSQFLVHFSKIL-------------ITPPY-PGYKMAPILQRIGLPGLKYP-QYTLSGIVSYLKIVKIGQTTFINSIQAHA-ASIKTMVYKYRKHFTGY---------------RLDLLQVAVLRYYGMSQTSQYAVSFSVAFRQALE----------VHTL----HQY---------------------------------------------------------------------------------------------------------------------------

>Ctest_CP52k_homolog14_isoform_4

-------------------------------------------------------------------------------------------------------------------------------------------------------------------------------------------------------------------------------------------------------------------------------------------------------------------------------------------------------------------------------------FRQALEVHTFHRYSVAYVSQFLV--------------------------------HFSKILVT---PPY-----------PGYKMAPLLERI--GLPG-LKYP----QYTLSGVVSYL-KVVKITQTTFISSIQAH---AAIIKKTVLKHWKHYT-----GYR----LDLLQVAVLRYYG----------------MSQTSQY--------AVSFSVAFR--------------QALQVHTFH--------------------------------------------------------------------------------------QYSIAYVSQFLVHFSKILVTPPY--------------PGYKMAPLLQRIGLPGLKYP-QFTLSGIVSYLKIVKISQTTFISSIQSHA-ASIKTMVYKHRKHYTGY---------------RLDLLQVAVLRYYGMSQTSQYAVSFSVAFRQALE----------VHTFHRYSVAYVS-------------------------------------------------------------------------------------------------------------------------

>Ctest_CP52k_homolog14_isoform_5

--------------------------------------------------------------------------------------------------------------------------------------------------------------------------------------------------------------------------------------------------------------------------------------------------------------------------------------------------------------------------------------------------------------------------------------------HFSKILIT---PPY-----------PGYKMAPLLQRI--GLPG-LKYP----QYTLSGIVSYL-KIVRISQTTFINSIQAH---SAIIKKTVLKHRKHFT-----GYR----LDLLQVAALRY------------------YGMSQTS------QYAVSFSVAFR--------------QALEVHTLH--------------------------------------------------------------------------------------QYSVAYVSQFLV----------------------------------------------------------------------------------------------------------------------------------------------------------------------------------------------------------------------------------------------------------------------------------------------

>Ctest_CP52k_homolog14_isoform_6

-----------------------------------------------MLRTVLVAALTVTAIVSAGNGVKQASHPYHGGNLRHVLASMGLPEFAQPNDSLNGLMSYLQQVH--VSRAEFLGRVQMYAPFISGIVHSLKKRYRGVAGDLLQLAALRHYGCEPQVGNHLWLGQHFQRYLQRMKMPHSKNYNIKFVISFLNGLKGYMSKLRQPPKVDIAPVPTPAPYP--------------------------GYELLTVLK-------------------------------------------SAGLPGFHNPRFSLGGLISYMKITKITHTTFIVGIKAHISSIKQIIIKYRTQFTGYQADFLQLAALRYYGMPRTSPYLVSFITAFKKSLSGPSQF---SHGVAFVSQFLI---------------KFSKILIT---PPY-----------PGYKMVSLLQRI--GLPG-LKYP----QYTLSGIVSYL-KVVKISQTTFISSIQAH---AAIIKKTVLKHWKHYT-----GYR----LDLLQVAVLRYYG----------------MSQTSQY--------AVSFSVAFR--------------QALQVHTFH--------------------------------------------------------------------------------------QYSIAYVSQFLVHFSKILVTPPY--------------PGYKMAPLLQRIGLPGLKYP-QFTLSGIVSYLKIVKISQTTFISSIQSHA-ASIKTMVYKHRKHYTGY---------------RLDLLQVAVLRYYGMSQISRYTVSFSVA------------------------------------------------FTQALKVSVYHQYT----------------------------------------------------------------------------------------

>Ctest_CP52k_homolog14_isoform_7

---------------------------------------------------------------------------------------------------------------------------------------------------------------------------------------------------------------------------------------------------------------------------------------------------------------------------------------KISQTTFISSIQAHAAIIKKTVLQHQKHYTGYRLDLLQVAVLRYYGMSKTSQYTVSFSVAFRQALEVHT--FH-QYSVAYVSQFL-------V--------HFSRILIT---PPY-----------PGYRMAPLLQRI--GLPG-LKYP----KHSLSGIVAYL-KIAKISQTTFITNIQAH---SAIIKKTVYKHTKHYT-----GYR----LDLLQVAVLRYY-----------------GMSQISR-------YTVSFSVAFT--------------QAL---KVSVY-H----------------------------------------------------------------------------------QYTASYVSAFLARFSELLVRP----------VVPSY-PGFEVKAALQRLQLPSLHRE-RSSLSGLVSHLRSIRISPVTFASRIMTYG-DKIREMVNKYKRRYSGM---------------NADLMQLCALRYFSFPLDIQRRVQFGAAFRQFLG----------TQSMK----QYNTRFVSKF----------LTDFIAHVRKFQLKSYKKYTSILW*--------------------------------------------------------------------------------

>Ctest_CP52k_homolog15

------------------------------------------------------------------------------------------------------------------------------------------------------------------------------------------------------------------------------------------------------------------------------------------------------------------------------------------------------------------------------------------------------------------------------------------------------------------------------------------------------------KVVKITQTTFISSIQAH---AAIIKKTVLQHQKHYT-----GYR----LDLLQVAVLRY------------------YGMSQTS------QYAVSFSVAFR--------------QALAVHTFH--------------------------------------------------------------------------------------HYNVAYVSQFLVHFSKILITPPY--------------PGYKMAPLLQRIGLPGLTYP-RYTLSGIVSYLKV-----------------------------------------------------------------------------------------------------------------------------------------------------------------------------------------------------------------------------------

>Mlong_CP52k_homolog10

--------------------------------------------------------------------------------------------------------------------------------------------------------------------------------------------------------------------------------------------------------------------------------------------------------------------------------------------------------------------------------------------------MLGPMLMMVLSGAASAQTSL-----------------------------------------------TGASLQPALTTA--GLSS-FSST----TVALGGLASFL-QQKEISLTTFNSRVTQY---ETKIRSSAGSVLNRIG-----GQV----GDLRALLVLHYYVS---------------EPIVVNN---------IPLLARVA--------------AFAEKQNAATL------------------------------------------------------------------------------------VYTVENVKNFLLSFDGELAETF---------------PPLGLLTELTNIGLPTLKQQ-ENSLNGLLSYLDSARIDRQMFVDSISSNG-DNIKTLFEDNKMLYDE----------------DGDLVQLAAAVYYYGDTSTYFPTFTDAFSEAVPSS---------DENSPAITKNFLDSFVTRLNTPLPELPDLIFG*-----------------------------------------------------------------------------------------------------

>Mlong_CP52k_homolog15

--------------------------------------------------------------------------------------------------------------------------------------------------------------------------MAYRYHLSHGVLATTPPRYKNILRPLVQHCLLPVVSDTAPPVRISQTMMRSMLLLALSVTVAIVASSPATTRLKDRVRQISLQATLA----------------------------------------------DIGLPSFHNPAISLSGLKSYLQRQGVTTEQFNRSLLKHRATVKDIILSLWTKHEGALADLVQLSALSYYGGESTVKNIRLSPLITGYITTTKTPSSA-DFNVRYITRFLNGFKGYVSKNAGQQSVVFPDPPPA---PSY-----------PGSELLATMRSI--GLPT-LTNK----QASLGGVDAYL-SSAGITMETFARRITLF---GEDMNRLVMQHAEQYS-----GSR----GDLLQVAALRYFG----------------AGGSYSV------QFSVAFEEALT----------------------PIS------------------------------------------------------------------------------------EYTVSYVTSFIDRFGQYLSTPMY--------------AGYSLRSRLVSAGLPVMSAP-HICLPGLISYLQSNSYSRDTLVSRIQTQS-SAIVATVNQYGAQYSGV---------------RGDLLQLCVIRYYAMPAAAREEVTFGDTFPAYFR----------TVTLTQFNAAFVSEM--------------LSGFVEYIQNQLDDYSGELW*------------------------------------------------------------------------------------

>Tform_CP52k_homolog3

---------------------------------------------------------------------------------------------------------------------------------------------------------------------------------------------------------------------------------------------------------------------------------------------------------------------------------------------------------------------------------------LITGYPSVFRPIQQVVQQSGVAT-----------------------------------------VTY-----------RSRSLQRVVQQI--GLPA-FSRP----QASLGGLNVLL-RSANIPHRRFYSRVSQV---GGQIRSLVHSMQGQYT-----GVE----SDLLKLAALRYYA----------------LPISIQR--------RVEFADAFA--------------SYLDRTQIPS-------------------------------------------------------------------------------------RYTTQFTSSFLTGFERYIQRYRLK----------------------------------NHNYGQSLGWW-------------------------------------------------------------------------------------------------------------------------------------------------------------------------------------------------------------------------------------

>Tform_CP52k_homolog2

-----------------------------------------------------------------------------------------------------------------------------------------------------------------------------------------------------------------------------------------------------------------------------------------------------------------------------------------------------------------------------------------------------------------------------------------------------------------------------------------FYS----GQYQSGLFAYL-RTLNFP----------------------YNQNSLIV-----PLS----LTDIRLATYQY------------------RPLSVQS-------FNQFFARFVL------KYNGK---VFQ---GIPPL-R----------------------------------------------------------------------------------TLDNILNLDLIARY-PSVFRPIQHVV---------------------------HRQT-TASVSPIRQYPQPVPVVVKPDPVYAI----------------------------------------------------------------------------------------------------------------------------------------------------------------------------------------------------------------------

>Mlong_CP52k_homolog1

----------------------------------------------------------------------------------------------------------------------------------------------------------------------------------------------------------------------------------------------------------------------------------------------------------------------------------------------------------------------------------------------MFRPVLFAVLMAGICSGT-GDSRV-------------P--------GAGDPTPP---RIP-----------DSYWLKKSLY----GFLP-LHHY----NVSLQGLESVL-LEEEVFANDFHNRISAY-----KHATLFEPYNSLLD-----KVD-VTLRDEIQLAVLCYFAL---------------ETSADSH--------SDSFPET-------------------------------------------------------------------------------------------------------------------------------------------------------------------------------------------------------------------------------------------------------------------------------------------------------------------------------------------------------------------------------------------------------------------------

>Mlong_CP52k_homolog7

----------------------------------------------------------------------------------------------------------------------------------------------------------------------------------------------------------------------------------------------------------------------------------------------------------------------------------------------------------------------------------------------MFRPVLFALLTAISCSQR-GHNGV-------------P--------GARELVPP---PPY-----------DHYWLKRGFA---------LFYPFSNEDATLNGFDDFL-MKYNWSAQSFYQRLHEYQINQIQVPNKNMTKKYDVS-----PVR----VDQVQLVVSSFFGV---------------YTSASEY--------DDVFPYIFLE-------------QFLDNTTVPTP-E----------------------------------------------------------------------------------DYTENYVTSFLEEFKKHI--------------------------------LPDPIPP-LSSKTNEQVMLPGRKM*-------------------------------------------------------------------------------------------------------------------------------------------------------------------------------------------------------------------------------

>Mlong_CP52k_homolog12

----------------------------------------------------------------------------------------------------------------------------------------------------------------------------------------------------------------------------------------------------------------------------------------------------------------------------------------------------------------------------------------------MYGPVLFAVLIAGVCSGT-GDNRA-------------P--------GAGDHTPG---NSDSSGTGEVYDVDDVYWLKKSLD----GFPP-LRHY----NASLEGLKKEI-MRKGVYPVDFYSHISAY-----NQDTVLEPYTSLAA-----KYN-ATLHDEIQLAVVCYFVL---------------VTTVDKY---------RKFPMDTV--------------EEVFNHALPPK------------------------------------------------------------------------------------TYNIFYVQEFLKEFLNHVRVPRFS*---------------------------------------------------------------------------------------------------------------------------------------------------------------------------------------------------------------------------------------------------------------------------------

>Mlong_CP52k_homolog13

----------------------------------------------------------------------------------------------------------------------------------------------------------------------------------------------------------------------------------------------------------------------------------------------------------------------------------------------------------------------------------------------MLRLTVLCVLATTVICVASEQSPF-------------K-------------------PDG-----------DVYWLQLSIS----GFQT-FYHI----ANSLEGLRRFL-VKEEVTPRQIYDNIRAY-----KGDAVVDANTAMVE-----KYNAVSIRDQVQLVVLTYLGG---------------FTSAEHY---------VTIPTEVE--------------EFLD--TTEPLS-----------------------------------------------------------------------------------PYGPGTVEDYLDAFFKYMEKSDLA*---------------------------------------------------------------------------------------------------------------------------------------------------------------------------------------------------------------------------------------------------------------------------------

>Mlong_CP52k_homolog20

-----------------------------------------------------------------------------------------------------------------------------------------------------------------------------------------------------------------------------------------------------------------------------------------------------------------------------------------------------------------------------------------------MLQPLLLVLLWAAATVA-DGAP----------------------------------ENY-----------AGKPLAQILDQF--DMSK-LKYQ----MVSLQGLERAL-TEEDISHQQFVDLFKRYKI-GGDIGIVRIPSMHLANRYRGNADKDKIQRDKSQLAVLAYFGVV--------------LTKEEEY---------NFFPYFAV--------------QEFLDTEKKKLST----------------------------------------------------------------------------------TYTVDDAYKFVSRFITYMRDD--------------------------------------------PDYFYKMELDDEDVN*-------------------------------------------------------------------------------------------------------------------------------------------------------------------------------------------------------------------------

>Mlong_CP52k_homolog21

-----------------------------------------------------------------------------------------------------------------------------------------------------------------------------------------------------------------------------------------------------------------------------------------------------------------------------------------------------------------------------------------------LRPVLFAVLMAGISSGTEDNGVPEAGDPTPENSDSSEKGDSKVPGAGEQATPQ---TND-----------TVIWDTLLVKKSLYGFLP-FHHY----NASLEGLEDAI-FEEDVFPNDFHNRISAY-----EHTTLFKPYNSLLD-----KHA-VTLRDEIQLGVLCY------------------FAIETRA-----DRYNDEFPTETV--------------TKFLNKVVAPI------------------------------------------------------------------------------------TYNDEYFSTFVANFLSIVRGKKTP*---------------------------------------------------------------------------------------------------------------------------------------------------------------------------------------------------------------------------------------------------------------------------------

>Mlong_CP52k_homolog2

----------------------------------------------------------------------------------------------------------------------------------------------------------------------------------------------------------------------------------------------------------------------------------------------------------------------------------------------------------------------------------------------MLRSSLLLVVSAVVTLGQ-DSAP----------------------------------ESF-----------PGSEYLSSLSGLNDETKT-LLLS----REMLGAWASYM-EEFGHSKTTFDTRLSEDSV-ISAIQIDVSRYRRDFN---SIDFR----RDLALLLSMHT------------------RALSFGDTSKYRAAINDAFNLNCY-------------------------------------------------------------------------------------------------------------WWTTNSIVDVLRS---------------------------------------------------------------------------------------------------------------------------------------------------------------------------------------------------------------------------------------------------------------------------------------------

>Mlong_CP52k_homolog9

-----------------------------------------------------------------------------------------------------------------------------------------------------------------------------------------------------------------------------------------------------------------------------------------------------------------------------------------------------------------MFLLVLLLGLSLSTITTASPAGKDNKTPLKPDQSNDADIPPADTNSPSSSADISSEAADTPPVGTTSPSSSEAEDTPPDNAAA---PTF-----------SGSEYLPILT----EIQVDLHDE----EPALGGWKRYM-DEHSINKDSFETQLRNY---QHFVRQQISSIQGQYT-----DYQ----KDLAKLFIVHL------------------NGID----------YGEPFDINAS--------------IASVLASAPAG------------------------------------------------------------------------------------QYTADSVFQHLQSFREEIAKD--------------------------------------------IDKVNEKDSKDK*----------------------------------------------------------------------------------------------------------------------------------------------------------------------------------------------------------------------------

>Mlong_CP52k_homolog11

----------------------------------------------------------------------------------------------------------------------------------------------------------------------------------------------------------------------------------------------------------------------------------------------------------------------------------------------------------------------------------ELILTGPTAPHSIMMRAAVLISVSVAIVLG-QEYPL-------------P--------AGCDCQTA---DSF-----------PGSEYLSSLSVLNEETRT-LLFP----REMLGAWASYM-REFNENKTEFDNRLAI----ATVISNIEFDVEKRRRNFNEVDFR----RDLVLLLAMHT------------------RGILFGE--------STKYRAAIN--------------DAL---NLNCY------------------------------------------------------------------------------------SWTSDNILVVLRRFRTQIRRPNEI*---------------------------------------------------------------------------------------------------------------------------------------------------------------------------------------------------------------------------------------------------------------------------------

>Mlong_CP52k_homolog14

------------------------------------------------------------------------------------------------------------------------------------------------------------------------------------------------------------------------------------------------------------------------------------------------------------------------------------------------------------------------------------------------MLPALLLFALTAAAAG-APQPVTVTGVLRPSPPPPP--------GFYPVFPA---QTY-----------SGHEYLSSIKSL--GMDS-LNYP----SISLGGWAKYM-DQYEYCRTQFEDKIAAK-------KTEILQAINGFSNYGTNDYR----RDIAQLMVLHKYGL---------------DYGDSQK-------FAAAYDAVLA--------------LTVTQFDVTTV------------------------------------------------------------------------------------GIALDYFRRQIEPSQSN*----------------------------------------------------------------------------------------------------------------------------------------------------------------------------------------------------------------------------------------------------------------------------------------

>Mlong_CP52k_homolog17

------------------------------------------------------------------------------------------------------------------------------------------------------------------------------------------------------------------------------------------------------------------------------------------------------------------------------------------------------------------------------------------------MFGAALLLSLTA------------------------------------TAAV---ATY-----------PGHEYGKILKEA--GLPD-LIHR----NKTLEGWVDYM-NEHAVDATTAQANARGN---SSNMAALFTQLETVTN-----RTYTDERRDVRQLFVLHRYAL---------------YDVGDAE--------SFFIAERYVMNY-----------YDLGLFAVQVKTD----------------------------------------------------------------------------------EYSAYIVKTLLNLFRKVL------------------------------------------ECAGEEEDDDCE*---------------------------------------------------------------------------------------------------------------------------------------------------------------------------------------------------------------------------------

>Mlong_CP52k_homolog22

------------------------------------------------------------------------------------------------------------------------------------------------------------------------------------------------------------------------------------------------------------------------------------------------------------------------------------------------------------------------------------------------MLRALLRVMLLLSLTA-AKK-----------------------------------EEY-----------YGEEYLQNLQSL--KLPA-LVNP----RATLGGWKKYM-VKYGYSKAAFETEIASAKK-LRSIKKEIPELISRIP-IPPGDFR----RDLAQLFILHKYGI---------------IYGDISR-------INDLAATYLV--------------ETDYFYDSEEV------------------------------------------------------------------------------------LHVLIYLRNAIRKDGEDSRK*-------------------------------------------------------------------------------------------------------------------------------------------------------------------------------------------------------------------------------------------------------------------------------------

>Mlong_CP52k_homolog3

----------------------------------------------------------------------------------------------------------------------------------------------------------------------------------------------------------------------------------------------------------------------------------------------------------------------------------------------------------------------------IQLNNYFIQYIKTANIASSFAYNSIYVHTFLSGFKSYLQTFHILQKQL----------------VPVTPLYK---PAY-----------PGHELLSTLTEM--GLPA-LEYP----ETSLGGIVAHL-KLASVSLKRFIGAIKQY-----GNDIAEKVAESQYH-----GIQ----RDLWQLVVLDYYC----------------GGQQNSD--------NFPLNSYFK--------------EYIKIADIPSPP-----------------------------------------------------------------------------------AYNNLYVQVFLDGFESYLRVS---------------------TKLLRSSSIAQKAR*-------------------------------------------------------------------------------------------------------------------------------------------------------------------------------------------------------------------------------------------------

>Mlong_CP52k_homolog8

----------------------------------------------------------------------------------------------------------------------------------------------------------------------------------------------------------------------------------------------------------------------------------------------------------------------------------------------------------------------------------------------MLRASLLTALAAGMAAAQ-----------------------------------------------------NITTLKTVFS----SVDLNVEFP----AVSLVGFQQYL-EQVGAS-EKFGDRINTH---NHIIRIAAGDLANSFT-----GAK----SDLSKILLLCYYGC---------------DSQVDAF---------NLPSSDIR--------------DFLNGFTEPTAE-----------------------------------------------------------------------------------QYTTGYVNARMDDFF*------------------------------------------------------------------------------------------------------------------------------------------------------------------------------------------------------------------------------------------------------------------------------------------

>Mlong_CP52k_homolog16_isoform_1

----------------------------------------------------------------------------------------------------------------------------------------------------------------------------------------------------------------------------------------------------------------------------------------------------------------------------------------------------------------------------------------MFRFTFLTVLSAAASLAPSSASPV-QQPVI-----------------------------------------------TGSDISTILSAT--PLPT-LSFR----TVSTTTLAKHL-QANGASLHTFRSILQRYGK-VIADEVKKQGYTSVFG-----DLW--------QLINLDFYA----------------GSIDFYYGDVYGINTDLPLNSYFL--------------QYIASLDASALGS----------------------------------------------------------------------------------DYTEEYALARLSGFWSFLLANADLSP-----FRPAY-PGYELISVLQEIGLHALTYP-RPSLGGLVGRLNVAQISRETFNSAVRQYN-STIVSKVAQYQNQYQGV---------------KRDLWKLVVLEYYGTTSEPNVNAALKTSFDTYTT----------GRTIPA-ASLYTDVYVLTF----------LQGFASPSGGK*---------------------------------------------------------------------------------------------

>Mlong_CP52k_homolog16_isoform_2

----------------------------------------------------------------------------------------------------------------------------------------------------------------------------------------------------------------------------------------------------------------------------------------------------------------------------------------------------------------------------------------------MFRFTFLTVLSAAASLAPSSASPV-------------------------QQPVI-----------------TGSDISTILSAT--PLPT-LSFR----TVSTTTLAKHL-QANGASLHTFRSILQRYGKVIAD-----EVKKQGYT-----SVF----GDLWQLINLDFYA----------------GSIDFYYGDVYGINTDLPLNSYFL--------------QYIASLDASALGS----------------------------------------------------------------------------------DYTEEYALARLSGFWSFLLANADLSPFR------PAYPGYELISVLQEIGLHALTYP-RPSLGGLVGRLNVAQISRETFNSAVRQYN-STIVSKVAQYQNQYQGV---------------KRDLWKLVVLEYYGTTSEPNVNAALKTSFDTYTA----------DEHIPA-PSSYTDVYVLTF----------LLGFPSPTGGK*---------------------------------------------------------------------------------------------

>Mlong_CP52k_homolog18

------------------------------------------------------------------------------------------------------------------------------------------------------------------------------------------------------------------------------------------------------------------------------------------------------------------------------------------------------------------------------------------------------------------------------------------------------------------------------------LPA-LEYP----ETSLGGIVAHL-KLASVSLKTFIGAIKQY-----GNDIAEKVKEKKYK-----GIQ----GDLWQLVVLDHFSR---------------RQQNSEN-----IRLNNYFKQYVK-----------------TTYIASPF------------------------------------------------------------------------------------AYNSVYVHSFLSGFKSYLQTSH------------------------------------------------------------------------------------------------------------------------------------------------------------------------------------------------------------------------------------------------------------------------------------

>Mlong_CP52k_homolog4

---------------------------------------------------------------------------------------------------------------------------------------------------------------------------------------------------------------------------------------------------------------------------------------------------------------------------------------------------------------------------------------------MSRLLLLIALVTITGAWSP-PYKRKTPTSFG-------R--------ASYYIPSK---TTY-----------SVDNLESALS----SLGM-SYHY----RSSLSGLISYL-KEKKLPQAKYIAGVRKY---KERIRSGLASAKDQYN-----GVS----GDLLQLSALVYN-----------------SGEDVGS-------YKVSLLYAFK--------------EYLKKNKAPLA-K----------------------------------------------------------------------------------KYKASYVKSFLKSFKKHLGELNKKQE-------------SQLASTLSDLGLYPLKRQ-KASYNGYSDYLKSKKRTEASAVYVLKQYS-NQIKNVVGQYKYNSYNRD------------------------------------------------------------------------------------------------------------------------------------------------------------------------------------------------

>Mlong_CP52k_homolog5

--------------------------------------------------------------------------------------------------------------------------------------------------------------------------------------------------------------------------------------------------------------------------------------------------------------------------------------------------------------------------------------------------SAKNFLDGYK-----ARLPRI---------------------RFHRSEMI---YDN-----------RNYLFNFIDAVEEIGLPS-LECG----RISSAGFNNHL--GSSSRQNQVAGEMRKH---KEAIKELVSKHMDTYV-----DSD----RDLLILSAIRYYL----------------IPADTRS--------RVNFDTTFS--------------AHFAEQRATTY------------------------------------------------------------------------------------RYNAEIARRFCVLYIRYIKR--------------------------------------SLQIRGTLFWIR*-----------------------------------------------------------------------------------------------------------------------------------------------------------------------------------------------------------------------------------

>Mlong_CP52k_homolog19

--------------------------------------------------------------------------------------------------------------------------------------------------------------------------------------------------------------------------------------------------------------------------------------------------------------------------------------------------------------------------------------RASERIPSPESYGASIVPKYMKCFKT-HVEKLNEKMAD---------------------------HDH-----------RSHFASTLEEV---GLYP-LRRQ----KASFNGYSDYL-KGKKSKRVSPINILKQY---ANQIKMVVNQYK--YN-----NYN----RDLLQLSAIEYYS----------------LPFLTRR--------KVSLGYAFQ-------------------------------------------------------------------------------------------------------------GYLSSHRTKTYN----------------------------------------------------------------------------------------------------------------------------------------------------------------------------------------------------------------------------------------------------------------------------------------------

**File S5.** Alignment of all CP100k homologs.

>Aamph_CP100k_homolog1_isoform1

---------------------------------------------------------------------------------------------------------------------------------------------------------------------------------------------------------------------------------------------------------------------------------------------------------------------------------------------------------------------------------------------------------------------------------------------------------------------------------------------------------------------------------------------------------------------------------------------------------------------------------------------------------------------------------------------------QPFGDGYLEFLYLRLEVIIKQVVVIEQQVPIDDYVTQQILSVF-ANVRISVEARRTIIRFIHNS--KLLPKPQKGVS-VVSQYQALLTSLTKRYPLDIFVLNKNILVQIRRQLI-SADIQIKLKVLRDINIIAYISLGLMDRLKGQTTVGYVRQIVYSSIRYFLRTNKVTSILSLEFVQFLL----HKYKVPSPPQI---WWQPPVEQHK-VNIQRDIYILPDIYLPVRSVHQLVVILQKRFVFVSIDNVQTIIVHTILILRANGVTITSDNCYDYLYRYYSGLPANIGVESFDISSVLDSIKRYAAQTTVTEVHVQSALVELYLHMYYLKMPLPS-VKVRDGFLSFVIGAYGKVQVRRQLPFGKLFYDFLQGFLPKLPGYLKPFPIFAGPQVYKVFHSTLK-TPVYPSDIPLYFQLFRR---VTKGSLTMGSLQSSLSG--LSLLPG---LTSEELSSIIDLVKGKQLKVSQTEIRRAFAICRLSLGLSSVKIS--RSKLISIFQEVVISIVQKYKSLLVVSYVEQILLRIRTYGPKYRPVQPITPIKPGYPCKNSK-YIRC*--

>Aamph_CP100k_homolog1_isoform2

---------------------------------------------MLRLPLALALLLAASAYGNVL-FARSGCGCLRNPVAAKLTGEEISHLRGYVKERGVK-----HYDVLSNDALQAIFRFNLINNFPDVVPATRTGVLQIISESLNTLTDAVVPSVPQCGKIAGYLQKSVPGLAAG-GVSLDLRSLVASASVILHQRGVTVNLEQLNVLLKSGLAGYLQSTAYQSSYGSLIQLISALDHIDHNLPNILDQESLIVVRRALESRFNLDR-EIFDKRYKLAIKAFEENRRRLLASFNTLAYRGPNYETNVQLVIKQMLTIFSGISAKTVRIILNILQLTNSAGGKATPKDLLAMITVPKLDVSIRKITEAAANRVYLKLPEHHQGLTIDDIQEAYSIFIIGLASQGVQPLQL--EATYEAFIWHTQRFFLATRIYSVQAYLLYVMRVVVPLIPRGSQSFRLHIFDSSVVIDN-ILVPEGLTSIYEEGRQTIIKRIRGLQGSSSDITNRIIGGQGEKGVIGNDLKFQ-TIVPADVPGYDQFEYQNVILSAVQMREIASVLIQRFNQLKQPSLQLPLMRIMIHANVIPNSGAAAAAAFRRLFRGLPAYSGPTDLSFVLTQLSENRLQLTETQLLAGIQQFYVASRCLGYVIPQQTIPSVFLYTVREYLSTLASVPAQPFGDGFLEFLYLRLEVIIKKVVVIEQQVPIDDYVTQQILSVF-ANVRISVEARRTIIRFIHNS--KLLPKPQKGVS-VVSQYQALLTSLTKRYPLDIFVLNKNILVQIRRQLI-SADIQIKLKVLRDINIIAYISLGLMDRLKGQTTVGYVRQIVYSSIRYFLRINKVSNIPSVEFFRVLL----HQYKVPLPQLP---LPKRPVIQYP-KYTRAPIYILSGISLPVKQVEQLVVILRTRFVFVSIENVQSILAHTVLLLRASGQQIVQKNCYEVLTRYYRGLPKSISVGEFDIEDLVKEIDDQLKDATISGTGVQSALVELYLHMYYLKMPLPS-VKVRDGFLSFVIGAYGKVQVRRQLPFGKLFYDFLQGFLPKLPGYLKPFPIFAGPQVYKVFHSTLK-TPVYPSDIPLYFQLFRR---VTKGSLTMGSLQSSLSG--LSLLPG---LTSEELSSIIDLVKGKQLKVSQTEIRRAFAICRLSLGLSSVKIS--RSKLISIFQEVVISIVQKYKSLLVVSYVEQILLRIRTYGPKYRPVQPITPIKPGYPCKNSK-YIRC*--

>Aamph_CP100k_homolog2

---------------------------------------------MLRLSLPLAVLLAVSAAGDKYPISRFGCGCNRNIIAADLTVQEISQLRVYIQQLGIK-----NHPVLTDDVINAIFRFNLNNNYQGLVPSKRSTLLQIFSESFSSLTDAVLPSVSQCGKFGDYLHKATPLVISG-DRKFDMCSLVASYAVILHQREVTVNFDQLNVILKLGLQKYLKSTAYQSSYSMLTQLLTSLDFIDHDLPTILDYEELIAVRRALLLRYNIKR-ARFDNRFRLAIEEFKLNRHRLLSTFNTIAFRGPYYEIVVQEVIREIIKIFPGLSASSVRIILDVLQLTNAPGGKASPRDLLALITVPRLDAELYVIQQYYIQKYVASLVTYYPSISVEIIKEAYSTFVISLKSQGIQPVNT--IATYKTFYYYLQAYFQSTSSYSVESMLTFYLRMVATSIPRGSPDFRINIFQSTVVIDN-ILVPQPWTSIYRKGKASIIKRIVGPQGNSRNIIIRLKTGRGEKPVIQNDLKFK-NIVPADVPGYDQFEYQNVILSAIQLRQVASALIQRFNLLKQPSLQLSMLRIFIDAQLIKGTGVQAANAFRTLFQGLPAYTLPTDLTFVFSQLTELNLQLTETQIRVALQQFYVVTRSLGYVIPQETIPSVFVYSVREYLSTLTSIPTQPFGDGYLEFLYLRLEVIIKQVVVIEQQVPIDDYVTQQILSVF-ANVRISVEARRTIIRFIHNS--KLLPKPQKGVS-VVSQYQALLTSLTKRYPLDIFVLNKNILVQIRRQLI-SADIQIELKVLRDINIIAYISLGLMDRLKGQTTVGYVRQIVYSSIRYFLRTNKVTSILSLEFVQFLL----HKYKVPSPPQI---WWQPPVEQHK-VNIQRDIYILPDIYLPVRSVHQLVVILQKRFVFVSIDNVQTIIVHTILILRANGVTITSDNCYDYLYRYYSGLPANIGVESFDISSVLDSIKRYAAQTTVTEVHVQSALVELCIHLYSMQLPLPS-VQYRNKFLGYVIDAYGRKYRRHGLPLGARFYKFLKKFLPK----------------------------------------------------------------------------------------------------------------------------------------------------------ARHTKPKY------------ISCEY-D-YFKC*--

>Aamph_CP100k_homolog3

---------------------------------------------MLRLPLALALLLAASAYGNVL-FARSGCGCLRNPVAAKLTGEEISHLRGYVKERGVK-----HYDVLSNDALQAIFRFNLINNFPDVVPATRTGVLQIISESLNTLTDAVVPSVPQCGKIAGYLQKSVPGLAAG-GVSLDLRSLVASASVILHQRGVTVNLEQLNVLLKSGLAGYLQSTAYQSSYGSLIQLISALDHIDHNLPNILDQESLIVVRRALESRFNLDR-EIFDKRYKLAIKAFEENRRRLLASFNTLAYRGPNYETNVQLVIKQMLTIFSGISAKTVRIILNILQLTNSAGGKATPKDLLAMITVPKLDVSIRKITEAAANRVYLKLPEHHQGLTIDDIKGAYSLFIIALASQGVQPLQL--EATYEAFIWHTQRFFLATRIYSVQAYLLYVMRVVVPLIPRGSQSFRLHIFDTSVVIDN-ILVPEGLTSIYEEGRQTIIKRIRGLQGSSSDITNRIIGGQGEKGVIGNDLKFQ-TIVPADVPGYDQFEYQNVILSAVQMREIASVLIQRFNQLKQPSLQLPLMRIMIHANVIPNSGAAAAAAFRRLFRGLPAYSGPTDLSFVLTQLSENRLQLTETQLLAGIQQFYVASRCLGYVIPQQTIPSVFLYTVREYLSTLASVPTQPFGDGFLEFLYLRLASIIRQVTVVDQKVPIDDYVSQKIFSVFGSSVRISVEARRTIIRFIHNS--ELLPKVGQGVS-VVAQYQRLLKSLFKRYPIGTFILSTKELVYIRAELK-KAGISVDIKYLRDANVMAYIGLGLLNRLEKSMTVIRVRQIVLSSIRYFLRINKVSNIPSVEFFRVLL----HQYKVPLPQLP---LPKRPVIQYP-KYTRAPIYILSGISLPVKQVEQLVVILRTRFVFVSIENVQSILAHTVL-------------------------------------------------------------------------------------------------------------------------------------------------------------------------------------------------------------------------------------------------------------------------------------------------------------------

>Aamph_CP100k_homolog4

--------------------------------------------------------------------------------------------------------------------------------------------------------------------------------------------------------------------------------------------------------------------------------------------------------------------------------------------------------------------------------------------------------------------------------------------------------------------------------------------------------------------------------------------------------------------------------------------------------------------------------------------------------------------------PQQKIPSIFLATVGRYLSTLPTIPKQPFDYNFLEYLRYSLASIIEHLPAVGSQSVIDDYAMYKIFSIF-GHTKLSIYAKRIIIKYINEY--KLLPKAAQNVP-LLVQYQQLMESMVSKCSVSSFILSKKQLTTIQSDLYKSRRIRIELSLLVDINYMAYFAVCQSGAYTAVMN-----RYVYQSIISYTQTVRKPNYYSAEFFRILV---------ESSKGSKLPVSRPPLVQYR-RTPKRLCYIVPGIVLYREQLRQLVTLIRPRFTFVSMRNIRSIVAHTILILRAR-YSITQNNCYGHLTKYYNGIPINALG------------------------------------------------------------------------------------------------------------------------------------------------------------------------------------------------------------------------------------------------------------------------------------

>Aamph_CP100k_homolog5

----------------------------------------------------------------------------------------------------------------------------------------------------------------------------------------------------------------------------------------------------------------------------------------------------------------------------------------------------------------------------------------------------------------------------------------------------------------------------------------------------------------------------------------------------RPQPTYDAFEYQNVLLSSQHMQRVAFELAKRFEGLKEPSFRLPLLKILVRANLVTDTGDKAAAAFLRLFQGLPVFSRPSSLSFIVEQLREYRLQTTKAQIKAALDQFFVATKCLGYVIPQQKIP---------------------------------------------------------------------------------------------------------------------------------------------------------------------------------------------------------------------------------------------------------------------------------------------------------------------------------------------------------------------------------------------------------------------------------------------------------------------------------------------------------------------------------------------------------------------------------------------------------

>Aamph_CP100k_homolog6

---------------------------------------------------------------------------------------------------------------------------------------------------------------------------------------------------------------------------------------------------------------------------------------------------------------------------------------------------------------------------------------------------------------------QGIQPLNQ--MATYELFIYHSTIYFRSSCAYTVDDYFLFISRVVRPNIPLGSKHFKIISFDHSVVIEN-ILVPEPWRSNYEKSRDTIIKRLVGLQGSSDQITKRLIEGGGEKGYIKNIVNLKP-----------------------------------------------------------------------------------------------------------------------------------------------------------------------------------------------------------------------------------------------------------------------------------------------------------------------------------------------------------------------------------------------------------------------------------------------------------------------------------------------------------------------------------------------------------------------------------------------------------------------------------------------------------------------------------------------------------------------------------------

>Aamph_CP100k_homolog7

------------------------------------------------------------------------------------------------------------------------------------------------------------------------------------------------------------------------------------------------------------------------KKFDTSV-DIFKNRYQLAIQSYKANRNLLLDSFRTMAYRGPKYEMYLQEAIRETINIFPSISPSTVRIVFNNLQLSNTGSGMVSPLDLLAMVTTPVLDDDLKSITKVYAERLYNKMPGCYMGQEVE-IQEMYFLFLVGILSQ-------------------------------------------------------------------------------------------------------------------------------------------------------------------------------------------------------------------------------------------------------------------------------------------------------------------------------------------------------------------------------------------------------------------------------------------------------------------------------------------------------------------------------------------------------------------------------------------------------------------------------------------------------------------------------------------------------------------------------------------------------------------------------------------------------------------------------------------------------------------

>Aamph_CP100k_homolog8

------------------------------------------------------------------------------------------------------------------------------------------------------------------------------------------------------------------------------------------------------------------------------------------------------------------------------------------------------------------------------------------------------------------------------------------------------------------------------------------------------------------------------------------------------------------------------------------------------------------------------------------------------------------------------------------------------------------------------------------------------------------------------------------------------------------------------------------------------------------------------------------------------------------------------------------------------------------------------------------------PINALGAFDAYNLLQTLRVQPKRAAISSVGIQSAMAELYMHMRHLQMPFPSDNDVRITVLRNCLSAYSSKGMYRNVPFGRRFFAFLNTYLPM------------------------------------------------------------------------------------------------------------------------------------------------------------------------RRTAPNKRCMRYKKSFRC*--

>Aamph_CP100k_homolog9

----------------------------------------------------------------------------------------------------------------------------------------------------------------------------------------------------------------------------------------------------------------------------------------------------------------------------------------------------------------------------------------------------------------------------------------------------------------------------------------------------------------------------------------------------------------------------------------------------------------------------------------------------------------------------------------EYLSTLASVPTQPFGDGFLEFLYLRLASIIRQVTVVDQKVPIDDYVSQKIFSVFGSSVRISVEARRTIIRFIHNS--DLLPKVGQGVS-VVAQYQRLLKSLFKRYPIGTFILSTKELVYIRAELK-KAGISVDIKYLRDANVMAYIGLGLLNRLEKSMTVIRVRQIVLSSIRYFLRINKVSNIPSVEFFRVLL----HQYKVPLPQLP---LPKRPVIQYP-KYTRAPIYILSGISLPVKQVEQLVVILRTRFVFVSIENVQSILAHTVLLLRASGQQIVQKNCYEVLTRYYRGLPKSISVGEFDIEDLVKEIDDQLKDATISGTGVQSALVELYLHMY----------------------------------------------------------------------------------------------------------------------------------------------------------------------------------------------------------------------------------------------

>Aamph_100k AGS19349

---------------------------------------------MLRLPLALALLLAASAYGNVL-FARSGCGCLRNPVAAKLTGEEISHLRGYVKERGVK-----HYDVLSNDALQAIFRFNLINNFPDVVPATRTGVLQIISESLNTLTDAVVPSVPQCGKIAGYLQKSVPGLAAG-GVSLDLRSLVASASVILHQRGVTVNLEQLNVLLKSGLAGYLQSTAYQSSYGSLIQLISALDHIDHNLPNILDQESLIVVRRALESRFNLDR-EIFDKRYKLAIKAFEENRRRLLASFNTLAYRGPNYETNVQLVIKQMLTIFSGISAKTVRIILNILQLTNSAGGKATPKDLLAMITVPKLDVSIRKITEAAANRVYLKLPEHHQGLTIDDIQEAYSIFIIGLASQGVQPLQL--EATYEAFIWHTQRFFLATRIYSVQAYLLYVMRVVVPLIPRGSQSFRLHIFDSSVVIDN-ILVPEGLTSIYEEGRQTIIKRIRGLQGSSSDITNRIIGGQGEKGVIGNDLKFQ-TIVPADVPGYDQFEYQNVILSAVQMREIASVLIQRFNQLKQPSLQLPLMRIMIHANVIPNSGAAAAAAFRRLFRGLPAYSGPTDLSFVLTQLSENRLQLTETQLLAGIQQFYVASRCLGYVIPQQTIPSVFLYTVREYLSTLASVPAQPFGDGFLEFLYLRLAGIIRQVTVVDQKVPIDDYVSQKIFSVFGSSVRISVEARRTIIRFIHNS--ELLPKVGQGVS-VVAQYQRLLKSLFKRYPIGTFILSTKELVYIRAELK-KAGISVDIKYLRDANVMAYIGLGLLNRLEKSMTVIRVRQIVLSSIRYFLRINKVSNIPSVEFFRVLL----HQYKVPLPQLP---LPKRPVIQYP-KYTRAPIYILSGISLPVKQVEQLVVILRTRFVFVSIENVQSILAHTVLLLRASGQQIVQKNCYEVLTRYYRGLPKSISVGEFDIEDLVKEIDDQLKDATISGTGVQSALVELYLHMYYLKMPLPS-VKVRDGFLSFVIGAYGKVQVRRQLPFGKLFYDFLQGFLPKLPGYLKPFPIFAGPQVYKVFHSTLK-TPVYPSDIPLYFQLFRR---VTKGSLTMGSLQSSLSG--LSLLPG---LTSEELSSIIDLVKGKQLKVSQTEIRRAFAICRLSLGLSSVKIS--RSKLISIFQEVVISIVQKYKSLLVVSYVEQILLRIRTYGPKYRPVQPITPIKPGYPCKNSK-YIRC---

>Aamph_114k AKZ20818

---------------------------------------------MLRLSLALAVLLAVSAAGDKYPISRFGCGCNRNIIAADLTVQEISQLRVYIQQLGIK-----NHPVLTDDVINAIFRFNLNNNYQGQVPSKRSTLLQIFSESFSSLTDAVLPSVSQCGKYGDYLHKATPLVISG-DRKFDMCSLVASYAVILHQREVTVNFDQLNVILKLGLQKYLKSTAYQSSYSMLTQLLTSLDFIDHDLPTILDYEELIAVRRALLLRYNIKR-ARFDNRFRLAIEEFKLNRHRLLSTFNTIAFRGPYYEIVVQEVIREIIKIFPGLSASSVRIILDVLQLTNAPGGKASPRDLLALITVPRLDAELYVIQEYYIQKYVASLVTYYPSISVEIIKEAYPTFLISLISQGIQPVNT--IVTYKTFYYYLQAYFQSTSSYSVESMTTFFLRTVVTSIPRGSPHFRINIFQSTVVIDN-ILVPQPWTSIYRKGKASILKRIVGPQGNSRNIIIRLKTGRGEKPVIQNDLKFK-NIVPADVPGYDQFEYQNVILSAIQLSQVASALIQRFNLLKQPSLQLSTLRIMIRAGLIKGTGVQAANAFSTLFQGLPAYSLPTDLTFVFSQLTELNLQLTETQIRGALQQFYVVTRSLGYVIPQETIHSVFVYSVREYLSTLTSIPTQPFGDGFLEFLYLRLEVIIKKVVVIEQHVPIDDYVTQQILSVF-ANVRISVEARRTIIRFIHNS--KLLPKPQKGVS-VVSQYQALLTSLTKRYPLDIFVLNKNILVQIRSQLI-SAGIQIQLKVLRDINIIAYISLGLMDRLKGQNTVGYVRQIVYSSIRYFLRTNKVTSILSLEFVQFLL----HKYKVPSPPQI---WWQPPVEQHK-VNIQRDIYILPDIYLPVRSVHQLVVILQKRFVFVSIDNVQTIIVHTILILRANGVTITSDNCYDYLYRYYSGLPANIGVESFDISSVLDSIKRYAAQTTVTEVHVQSVLVELCIHLYSMELPLPS-VQYRNKFLGYVIDAYGKKYRRHGLPLGARFYKFLKKFLPK----------------------------------------------------------------------------------------------------------------------------------------------------------ARHTKPKY------------ISCKY-D-YFKC---

>Mrosa_100k BAB12269

---------------------------------------------MMRLSLVAVLLVTVSVTGHRPSFERRCCGCLRSPVAADLDDDEIGMLREYVKKQGVM-----HYESLSDISLKAIFRNKLLNNFPEEVPATRDGVLQVITESLGSLTDSVVPSVSQCGQIAGYLQKSVPALAQG-GFNVDLKSLVSSASVLLHQRGVTVNTDELNIFLKYGLINYLKSTVYQSSYSMLRQLIVTLDYLDHELPVILDYEELIAVRLALKKKFDTSV-DIFKNRYQLAIQSYKANRNLLLDSFRTMAYRGPKYEMYLQEAIRETINIFPSISPSTVRIVFNNLQLSNTGSGMVSPLDLLAMVTTPVLDDDLKSITKVYAERLYNKMPGCYMGQEVE-IQEMYFLFLVGILSQGIQPLNQ--MAIYELFIYHSTIYFRSSCAYTVDDYFLFISRVVRPNIPLGSKHFKIISFDHSVVIEN-ILVPEPWRSNYEKSRDTIIKRLVGLQGSSDQITKRLIEGGGEKGYIKNIVNLKPAITPRPQPTYDAFEYQNVLLSSQHMQRVAFELAKRFEGLKEPSFRLPLLKILVRANLVTDTGDKAAAAFLRLFQGLPVFSRPSSLSFIVEQLREYRLQTTKAQIKAALDQFFVATKCLGYVIPQQKIPSIFLATVGRYLSTLPTIPKQPFDYNFLEYLRYSLASIIEHLPAVGSQSVIDDYAMYKIFSIF-GHTKLSIYAKRIIIKYINEY--KLLPKAAQNVP-LLVQYQQLMESMVSKCSVSSFILSKKQLTTIQSDLYKSRRIRIELSLLVDINYMAYFAVCQSGAYTAVMN-----RYVYQSIISYTQTVRKPNYYSAEFFRILV---------ESSKGSKLPVSRPPLVQYR-RTPKRLCYIVPGIVLYREQLRQLVTLIRPRFTFVSMRNIRSIVAHTILILRAR-YSITQNNCYGHLTKYYNGIPIN-ALGAFDAYNLLQTLRVQPKRAAISSVGIQSAMAELYMHMRHLQMPFPSDNDVRITVLRNCLSAYSSKGMYRNVPFGRRFFAFLNTYLPM------------------------------------------------------------------------------------------------------------------------------------------------------------------------RRTAPNKRCMRYKKSFRC---

>Chunt_CP100k_homolog1

------------------------------------------------------------------------------------------------------------------------------------------------------------------------------------------------------------------QPEPLPVPAVEEQVPYFSFIINQYRPTVTGQTLRFGSPFASFLSGYLPKLPAAAGPFSIFNVPALYSGYQPFFSRYSTISDLPLYTQVFRAGSVP--------------------------------------------------------------------------------------------------------------------------------------------------------------------------------------------------------------------------------------------------------------------------------------------------------------------------------------------------------------------SLQGILSAAAALNDDDLSQGFYQNLS---DEETNNVIAVARQFGVPASITGAMFQRAFSPCRLALNLR-----------------------------------------LGGIIRSRGDVIGVIRQLLQRFGKTYTVQNFLSSKLIDDLQKIIQARLQSQQTI-------------------------------------------------------KIYHRPSGFFTNRVVIRKG*------------------------------------------------------------------------------------------------------------------------------------------------------------------------------------------------------------------------------------------------------------------------------------------------------------------------------------------------------

>Chunt_CP100k_homolog2

-------------------------------------------------------------------------------------------------------------------------------------------------------------------------------------------------------------------------------------------------------------------------------------------------------------------------------------------------------------------------------------------------------------------------------------------------------------------------------------------------------------------------------------------------------------------------------------------------------------------------------------------------------------------------------------------------------------------------------------------------------------------------------------------------------------------------LKISIQDLRDANAMALCGLGLFKRLETGLKPAQLRVLIRRAILSFVRVNKVSNIATEDFFRALF----GTVKVPLPELP---VPQAPVYQQPKPYVPPPIMLVPGLSL----------------------------------------------------------------------------------------------------------------------------------------------------------------------------------------------------------------------------------------------------------------------------------------------------------------------------------------------------

>Chunt_CP100k_homolog3

----------------------------------------------------------------------------------------------------------------------------------------------------------------------------------------------------LSLRNIEVAPSQLKVVLQNAVNSFIDGSQFVPSNSALSQLLAYMDSADLRLSQIVQLEELNSVRQALQTQFQIAN-FNFDKRYQAAIQDFETTRFNILNSLNGLVARGPNYEVEVQGAISRLVEQFSGL-------------------------------------------------------------------------------------------------------------------------------------------------------------------------------------------------------------------------------------------------------------------------------------------------------------------------------------------------------------------------------------------------------------KLSDGTARNVLEL--------------------------------------------------------------------------------------------------------------------------------------------------------------------------------------------------------------------------------------------------------------------------------------------------------------------------------------------------------------------------------------------------------------------------------------------------------------------------------------------------------------------------

>Chunt_CP100k_homolog4

----------------------------------------------------------------------------------------------------------------------------------------------------------------------------------------------------------------------------------------------------------------------------------------------------------------------------------------------------------------------------------------------------PEQILEAYAIFIMGITSQGVTPVPTPRDEVLRIFTQQLKMFSDSNLVYDPRNYFLFTSRISVPQIPRGSPAFQIGIFEPQVVVSKVVLVAQPLQSIYVEGRRSFIADVVGIQGN-------------------------------------------------------------------------------------------------------------------------------------------------------------------------------------------------------------------------------------------------------------------------------------------------------------------------------------------------------------------------------------------------------------------------------------------------------------------------------------------------------------------------------------------------------------------------------------------------------------------------------------------------------------------------------------------------------------------------------------------------------------------

>Chunt_CP100k_homolog5

-------------------------------------------------------------------------------------------------------------------------------------------------------------------------------------------------------------------------------------------------------------------------------------------------------------------------------------------------------------------------------------------------------------------------------------------------------------------------------------------------------------------------------SGSISTPKIKNIVKLRPRIESGNLPRFADYVFGDFKISGRDFYRISQIIASRYPAL---------------------------------------------------------------------------------------------------------------------------------------------------------SIRPSQEPVVGLILQALSSGNLPLPSESGALSAYIASYLSNLPAIQLPSPSAL------------------------------EPYLSDIGTFNINLNKAALNIAYQQFVIASQIMGY---------------------------------------------------QAPQTVLPNIFIQS--------------------------------------------------------------------------------------------------------------------------------------------------------------------------------------------------------------------------------------------------------------------------------------------------------------------------------------------------

>Chunt_CP100k_homolog6

---------------------------------------------------------------------------------------------------------------------------------------------------------------------------------------------------------------------------------------------------------------------------------------------------------------------------------------------------------------------------------------------------------------------------------------------------------------------------------------------------------------------------------------------------------------------------------------------------------------------------------------------------------------------------------------RSFGGTISGGPVTELGPQYLRFLQNGLGNVFQQIQLINDRVVLTDDQIRTIFSGI-PGFNPSSEAQRTIITILLGSLPSDTISKLQSSSLAIKTFQDQIKQLLAKYPVASYILSPNAVSQLVGSIN-----QAQAQQIIDANAIAFSSLGVRGILPPAQ---RFQDFVVSNINTFSQIHKPRYLSSPLFGRRVTAARGQRIPIPTFISPSPFTPQPPVVVIPPTPPPAISIPGVDVEIPNRDFVQISNQLTGRFPFVNRGSVPELVGHVVSILRTSGNAQGSISGQVK--------------------------------------------------------------------------------------------------------------------------------------------------------------------------------------------------------------------------------------------------------------------------------------------------

>Cmala_CP100k_homolog1

--------------PTRRAARLKRRGCDVRPQSLAPSLATSRPPSPVSAMKLALLLALLGAASGTIMFPRNGCGCGRNINARPLTPPEIKHLRGYVYNRGVK-----HADVLTDACLQGVFKFSILNNFPDVVPVRRAGMIQIIEQGLSELTDKVVPGADVVAKIVRYLGGAVPELKGS-QLNVDLSSLVPACAVVLHQRGVQLTHQQLQVFLRSALSGYLQSPAYKADYSPQSQLVACLDHFDHQLPSILELAALLPVRRQLEKRFNLES-DIFDHRFRQAVKAFEANRRRILESFNTIAFRGSRYEITIQQVIAKVVKIFPGTSQRTIRAVLNILQLTNTKGGQASPRDLLAMIAFPAVDKSLRDISVEIAHRWRLYFPKH-IDMSYAQVREAYALYIIGLLSQGVQPAKL--SIVHRLFVEHTQRFFLGVPQYNVESYILYVVRVVVPSIPRGSPGFIIRLFENTVVIDN-VLVPEPLKSIYQEGRDTIIPRIFGLQGNSIQISRRLARGEGAKPDVENIVNLRPRIVSGPLPTED-FSYSGVLISAAQLRAVSAVLEARFAALKSRRLYAPIIKVLIDADIIGGSGEKAAASLRRLFDRLPDFDVPSGLDVLVTQLSRKRLQLTRPQLLAGLQQFYVCSRALGHVIPRKALPGVFAAALGRYVQTLPKIPAQPFDIGFLRYLQKRLVSIIQRVVLVGDSVPVLGPQLDKIFSVF-PGLAVSPNAQRAIVRFIESS--NLIKGPIKDSGSAAEVLHRLLSGISNHYPHDAYILRGSQLKLLLTELR-RRSLKISIQDLRDANAMALCGLGLFKRLETGLKPAQLRVLIRRAILSFVRVNKVSNIATEDFFRALF----GTVKVPLPELP---VPQAPVYQQPKPYVPPPIMLVPGLSLTVKKVEYMVSLLRRRFSFVSLDNVQPILAHIISILRSRGEKITQAYLEEALVVYITALPK-FPVKGVDLAALLKRIDEQLVDVTITGIGIQSAFCELYLSLHALKLPLPS-AAVRDDFFTLVVTMYGRTFIRQQIRFGVPFYEFLGKRLPKLVDYVKPFPLFAAPKLFFAFQRELKPAPVPVADIRILIQVVIRGAGLKPDGLTLARLLAVLRDREIKKTAAAGPLSDAELKAILAHVQQKRAKVTQAHVRRAFIACRLALRLTGAPLS--RKVLVQTFSRVVVTVVQRYQKLLVPQLASQLVSALQVGGFGGKLGGDFGSIVIEKEPKKHFGGFFRDD*

>Cmite_CP100k_homolog1

-----------------------------------RTPAAEPLPATMWRPALLTLLLAAAAAGGDLHFNRNGCGCLRNPVAGPLTAAELAQVRKYIGGLGVK-----HVGSLTDQALEAVFRFNLLNNFPGVVPSNRAGVIEVILRGLRELSDSVVPSQKQVDEVVTYLGGNVPELAAG-KVQLDLPSLVGSGAVVLHQRGVGISQQQLGLVLGNGLSGYLQSPAYKVDYNGQSQLVATLDSIDHAIPSILELEQLKPVRAKLESRFNLDS-KIFDSRFRQSVESFERNRQRILDSFNTLASRGAGFEVSIQTVISKVVELFPGLSSASARTILNILQLTNSAGGRARPRNLISMIAIPPIDPSLRVITDVVVNRFQLHMPDK-VGLSAVQVREAYALFIVGLASQGITPLRL--DETHASFIFHTQRFFLASSVFTPEAYILYVIRVVVPSIPRGSPAFSIRLFDPTIVVDE-VLVPEPLRSIYEEGRKTIIPRVFGIQGNSIQITRRLAQGEGDKPPVENPVVLRPPIVNGRLPTAKPFSYGGVALSGVQFQTVSAVLSSRFGALSARSLQEPILQALIEAGVVSGTGDAAAAALSRLFTGLPSFTAPGDLSPLVAELR--RLKLTQEQLLAGLQQFFVTTRSLGHIIPAGALPGVFSASVRRFIDTVPRPPKQPFNVDYLRYLRRRLPAIIERTVLVGNSVPVLGDQLDDIFSTL-GSLRISRRAQRTIVRFIDSS--KLVRGPFQGAGQAVSAFRKVLGGLASRYPRGLFGVGAGELGPLRAALG-RYGPAVSDQQLRDANAIATVGLGLFNRIDAGLKPAGLHQLLRRAILSFLRINKASNIPSADFIRAVY----GAQQAPLPTLP---VPVPDPHQEPRPPTVPPIPIIDDILLPVADVDRVISLLRGRFPFVSFDNVQPILQHVVLARRAAGVSITAGNLLGQLTSFVSSLPADLAVSALDVEALRKAIDGSIHDATVTGNGIQAGVVQLLISMHGLALPLPK-GADRDSFFTFVIVQFARTVRRQQLRFGAPYHAFLGGFLPKLTGLVRPYPLFSAPQLFSLFEKQLSPTLLRIDDLPLLIQAVTAATSKAGGPYSLQRLVKIVSGLRAAPAGLSALTDTEVSSVLTQLSAAQLSGVTGPQVRRAFGVCRLAARLAGGRLAGYRPTLLRLFSQLVVTTVRQHKGLLVPQLVPGLVGILRGGGAGP------------------VNGFITKG*

>Ctest_CP100k_homolog1_isoform_1

----------------------LPAAGAAPRLSGSAAPSAPVTAAMLRVPLALALLLVASANGSFM-FPRNGCGCIRNPVAAQLKPEEIAHLRGYIQQRGVH-----HYKAFTDRALQAIFRFNLLNNFPDVVPSSRAGVLQVVSESLNELTDKVVPSVSQTGEITGYLNGAVPELAGG-GVNIDLDSLVVAVSTILHQRGVALPLDRLNILLKTGLSGYLQSPAYHTNYGRLSQLVATLDHIDHNLPNILDQDLLIDVRRKLETRFNLDS-KIFDKRYHQAIKAFEANRARLMESFNSLAFRGPDYEVTIQTVIREVIRLFPGVSAGTLRSVLNILQLTNTGGGRATPKDLLAMITIPHLDSSIRSITDAVANRIYLKLPQHHQGLNRQHVHEAYALFIIGLASQGVQPLKL--QAAHEAFVWHTQRFFLGTRTYTVEAYILYVIRVVVPSIPRGSSAFRLHLFDSSVVINN-VLVPEPLKSIYKEGRQTIIARIQGLQGSSADITRRLVRGEGEKAVVENIVDLRPPIKNGPLPTYSKFEYDGVVLSATQQRAIFLELQRRFNRLRQPQLQLPLLRVLIRANVVRRN---PAAAFRRLFSGLPSFQAPQNVDGLITQLSQRRLQLTREQLLAGLQQFYVASRCLGIVIPSSSLPGVFIYTVTQYLQTLTTIPAQPFDYRFLQYVYERLASIIEQAVLINRSVPVVGRQVENIFSVF-GNIHISLRAQRTIVRFIDSS--GVLRSPVTGSSSAISVYKGLLASLVQRYPVANFVLSVRELIVLRSQLS-KLGIRVTLPYLRDANIMAFIGLGLFNRLQPTMTAVQFRQMIFVSIRSFVRINKVSNIPTSDFFRIVF----SINGKKVPSLP---VPQPPVIQAP---VPPPIYILSGLTLTVTQVHEVVSVLRVRFPFVSLDNVQGIIAHTVLLLRAKNKVVNQQNAHQALMQYYGGLPKGISIKGVDIDALLTSIDERLVDATISGNGIRSGLVEMYIHMHFLGLPLPG-AEVRDEFFSFIIGAYGQVQVRRQLPFGKYFYKFLSSFLSKLPGYLKPFPLFAGPKLFDAFSSQLK-VRIAPADIPLLIQAIRRTVSVKSSGLSVGSLLQIIAR--AKVSAFTSPLNAEELTSLVAYMKGQRLEVTQSQLSRAFAICQLTVRLSGRSTT--RKQVLQIFRGVIKSTKQQYGSLMVMSLVEQLVVRVRGLKQVHRIV---IPTKYKYQVHK--GLLG*---

>Ctest_CP100k_homolog1_isoform_2

----------------------LPAAGAAPRLSGSAAPSAPVTAAMLRVPLALALLLVASANG-HN-FARHGCGCIRSPVAAELGPEELSQLRGYIQNHGVN-----HYKTFTDQSLQAIYRFNLLNNFPDVVPSSRAGVLQLVSESLNGLTDQVVPSVTQVGQTVDYLRGAVPQLSGS-NVNIDLSSLVAASSAILNNRGLEIRLQELNTLLKTGLTGFLQSAAYHRNYGSLSQLVSSLDYIDSTLPSILDQDLLVDVRRRLVARYNQNS-KIFDQRYDQAIKIFESHRARLLASFNALAFRGPTYEVTIQSVIQAIHRLYPSVTTGALRSGLNVLQLTNTGGGRVTPRDLLAMITVPQLGSGIRSITDIFAGRVVSGLPLRHRGLTISQIREAYGLFIIGLTSQGIQPLQA--QAAYNAFVWHTRRFFGGVTVYSVEAYITYVIRVVVPSIPRDSAHFRLHLFDSNIVINN-VLVPEPLKSIYKEGRQTLLAPIRGLQGSSEDITRRLIRGEGEKPFVKDIVKLAPEIKNGPLPTYSKFEYDGVVLSATQQRAIFLELQRRFNRLRQPQLQLPLLRVLIRANVVRRN---PAAAFRRLFSGLPSFQAPQNVDGLITQLSQRRLQLTREQLLAGLQQFYVASRCLGIVIPSSSLPGVFIYTVTQYLQTLTTIPAQPFDYRFLQYVYERLASIIEQAVLINRSVPVVGRQVENIFSVF-GNIHISLRAQRTIVRFIDSS--GVLRSPVTGSSSAISVYKGLLASLVQRYPVANFVLSVRELIVLRSQLS-KLGIRVTLPYLRDANIMAFIGLGLFNRLQPTMTAVQFRQMIFVSIRSFVRINKVSNIPTSDFFRIVF----SINGKKVPSLP---VPQPPVIQAP---VPPPIYILSGLTLTVTQVHEVVSVLRVRFPFVSLDNVQGIIAHTVLLLRAKNKVVNQQNAHQALMQYYGGLPKGISIKGVDIDALLTSIDERLVDATISGNGIRSGLVEMYIHMHFLGLPLPG-AEVRDEFFSFIIGAYGQVQVRRQLPFGKYFYKFLSSFLSKLPGYLKPFPLFAGPKLFDAFSSQLK-VRIAPADIPLLIQAIRRTVSVKSSGLSVGSLLQIIAR--AKVSAFTSPLNAEELTSLVAYMKGQRLEVTQSQLSRAFAICQLTVRLSGRSTT--RKQVLQIFRGVIKSTKQQYGSLMVMSLVEQLVVRVRGLKQVHRIV---IPTKYKYQVHK--GLLG*---

>Ctest_CP100k_homolog1_isoform_3

------------------------------------------------------------------------------------------------------------------------------------------------------------------------------------------------------------------------------------------------------------------------------------------------------------------------------------------------------------------------------------------------------------------------------------------------------------------------------------------------LKSIYKEGRQTLLAPIRGLQGSSEDITRRLIRGEGEKPFVKDIVKLAPEIKNGPLPTYNKFEYDGVVLSARQQQAIFQALQQRFDRLRQPQLQLPLLRVLIRARVVRRN---PAETFQRLFRGLPSFQAPRNIDGLITQLGQNRLTLTREQLLAGLQQFYVASRSLGIVIPPRSLPGVFIYTIRQYLRTLTTIPAQPFDFRFLQYVQERLASIIQRTVLINRSVPVVGRQVENIFNVF-GNIQVSLRAQRTILRFISSS--GLVRSPVTSSSTAITVYRRLLASLVQRYPVSTFILSVRELIVLRAQLS-QVGIRVSLQYLRDANVMAFVGLGLFNRLQPTVTTVQFRQMIFVSIRSFVRINKVSNIPTSDFFRIVF----SINGKKVPSLP---VPQPPVIQAP---V----------------------------------------------------------------------------------------------------------------------------------------------------------------------------------------------------------------------------------------------------------------------------------------------------------------------------------------------------------------

>Ctest_CP100k_homolog2

------------------------------------------------------------------------------------------------------------------------------------------------------------------------------------------------------------------------------------------------------------------------------------------------------------------------------------------------------------------------------------------------------------------------------------------------------------------------------------------------------------------------------------------------------------------------------------------------------------------------------------------------------------------------------------------------------------------------------------------------------------------------------------------------------------------------------------NVMAFVGLGLFNRLQPTVTTVQFRQMIFVSIRTFARINQVNNIPSGAFFRIVF----STHGKTIPSLP---VPHYPVIQAP---VSPPIYILRGLTLPVRQVHEIVSALRSRFPFVSLDNAQAIIAHTVLLLRSRNSNFNYARAHPALMRYYSGLPRSLAISGVNIDTLLRTIDERLVDATISGAGIRSGLVELYLHMHFLGLPIPT-PAVRDQFFSFILSAYGQMQVRRQLPFGRYFFTFLNSFLGKLPGYVKPFPLFAAPKIYDAFSSQLG-VRISPADMPLLLQAIQRVAPAQGGQFNLGYLLQTIQR--ARPSPFSAPLTSSELTSLLGYFKTHQVAVSQPQLIRAFAICQLTVRLSVQSIT--RAQVLQAFQRVIMSTMRQNHGLFGAGLVNQLVSNFRQGKTIRRPY---Y--KHGFPKRI--NIFH*---

>Lanat_CP100k_homolog1

--------------------------------------------------------------------------------------------------------------------------------------------------------------------------------------------------------------------------------------------------------------------------------------------------------------------------------------------------------------------------------------------------------------------------------------------------------------------------------------------------------------------------------------------------------------------------------------------------------------------------------------------------------------------------------NIFQQSLRLYRGSTSAQQITELGPAYVSFLFNNLGKVFQQVQLINNRLVLSDDQIKVIFSSL-QGLQPSLDAQKAIIYLILGSLPDNTITQLQTSDAAIKAFQDKLKEVIAKYSVSSYILSPDVISKIVSSVN-----GATTQQIIDANAIAFSGLAVRGILPAA-------------------------------------------------------------------PRFEDFIVSNIN-----------------------------------------------------------------------------------------------------------------------------------------------------------------------------------------------------------------------------------------------------------------------------------------------------------------------------------------------------

>Lanat_CP100k_homolog2

-------------------------------------------------------------------------------------------------------------------------------------------------------------------------------------GNVDTDSSVTVTGNGVRAGIAGVVFSHLAQPQPLPVPTAEQQASYFSFLINRYRPTINARFLNFGAPFAGFLGGYLPQLPRATGRFSIFNVPTLYSRYSPFFSRLSRITDLPLFTQVFSRGSSA--------------------------------------------------------------------------------------------------------------------------------------------------------------------------------------------------------------------------------------------------------------------------------------------------------------------------------------------------------------------SLSAVLKAAANLNDEDLSQGFFQLLS---DDELAKIVELGKSLGISSSITGPMYRRAFSPCRLALKLQLSGV-----------------------------------------------------------------------------------LRSRSDVLGFMRQLLQRH-----------------------------------------------------------------------------------------------------------------------------------------------------------------------------------------------------------------------------------------------------------------------------------------------------------------------------------------------------------------------------------------------------------------------

>Majax_CP100k_homolog1

--------------------MGTGTSNSRSHCQLGSAPTASSTGTMMRLPLVAVLLLVASANGYRPSFARRGCGCLRSPVAADLDDKEIAILRGYVKERGVK-----HYESLSDISLKAIFRNKLINNFPDEVPATRDGLLQVIAESFGSLTDSVVPSVAQCDQIAGYLKKSVPALVGA-GVSIDLRSLVSSAAVLLHQRGVTVNTDELNIFLKYGLINYLKSTVYQSSYSMLRQLIVTLDYLDNDLPIILDYKELIAVRLALKKRFNTSV-NIFKNRYQLAIQSYKANRNLLLASFRTLAYRGPKYEIYLQQVIRETIKIFPSISAATVRKVFNSLQLSNTGSGMGSPKDLLTMITVPSLDASLRSITRMYANNLYKKLPACYMDQKIE-IQEIYFLFLVSILSQGIQPLNQ--VATYELFIYHSTSYFQSSCTYTVDDYFLFISRVVRSNIPLGSKHFKIISFDHSVVIEN-ILVPEPWTSIYKEGRDTIIKRLVGLQGSSDQITKRLIEGGGEKDYVKNIVNLKPAITAGPVPTYDAFEYKNVLLSSQHMQSIALELEKRFEGLKKPSLRLPLLQILVRADIITDTGDKAAAAFLRLFQGLPKFSRPSSLSYIMTQLSAYRLQATKAQIKAALDQFFVATKCLGYVIPQQQIPSIFVAVVGQYLSTLPTIPKQPFDYNFLEFLHFRLASIIEQLSAVGSQSVIDDYAIYEIFSVF-GRTGLSVYAKRIIIKYINEY--EILPKEAQ-----IAEYQQLIKSVMTKCSVNSLILNEKQLNSILSNLYKSKGIKIELSMLIDINYMAYFAVCQSGSYRPAM-----HNYLYRSIISYNLTVRKPKYHSAEFFRILIEQAKPSQVKIQQKLP---VSRLPLIQYR-RKPERPCYIIPGIILYKEQLRQLVTVILPRFAFVNMYNIRSIVAHTILILRAR-YRVTQENCYERLKEYYKSLPVN-ALKAFDSYKLLETLEVRPKRAAISSAGIQSAMAELYMHMHHLKMPFPSDDEVRIAVLRDCLSAYSSKGMYRSVPFGRRFHGFLKDYLPK------------------------------------------------------------------------------------------------------------------------------------------------------------------------RRTAPNKRCKLYKKSFRC*--

>Majax_CP100k_homolog2

------------------------------------------------------------------------------------------------------------------------------------------------------------------------------------------------------------------------------------------------------------------------------------------------------------------------------------------------------------------------------------------------------------------------------------------------------------------------------------------------------------------------------------------------------------------------------------------------------------------------------------------------------------------------------------------------------------------------------------------------------------------------------------------------------------------------------------------------------------------------------------------------------------------------------------------------------------VHSILVQSVLLLRASGQKVVQANCYHLLSRYFRSLPKSIAIGALDIDPLVKEIDDQLVDATISGTGIQAAMVELYIHMYYLKLPIPS-VEVRDKFLSFVLGAYGKIHVRRQLPFGKPFYEFLSGFLPRLPDYLQPFPLFAAPQLHSMLYSKLK-TPIYASDIPLYLRVVRK---MQKGGLTLGGLKRTLSN--VDLIPV---LQAKEITRLLDFVKDRKIKVIRGEIIRAFSLCRLTLGLSSVKIS--RANLIGVFERVMLTIVTKYNSLLLVGYVDEIVSRIRNYKPRI--IVPNRPKISMPRCKHSDKHFQC*--

>Majax_CP100k_homolog3

------------------------------------------------------------------------------------------------------------------------------------------------------------------------------------------------------------------------------------------------------------------------------------------------------------------------------VAHFPGLSSSTARSVLNLLQLTNSAGGKATPKDLLAMITVPKLDASLRSISEVYAQRALLKMPDQQRDLTVVQIREAYTLFIMALMSQGIRPVDQ--LKTFEAFIKYTQWFFLGNHVYSVEAYLLYALRVVIAYIPRGSEFFRSHIFDSTIVVDN-ILVPKPWKSIYEEGRDSIIQRIRSLQGSSDQITNRLKSSIGERGYVKNIVNLKPTITAAQVPTYD------------------------------------------------------------------------------------------------------------------------------------------------------------------------------------------------------------------------------------------------------------------------------------------------------------------------------------------------------------------------------------------------------------------------------------------------------------------------------------------------------------------------------------------------------------------------------------------------------------------------------------------------------------------------------------------------------------------------------

>Majax_CP100k_homolog4

------------------------------------------------------------------------------------------------------------------------------------------------------------------------------------------------------------------------------------------------------------------------------------------------TRERSLER-----------------------------------------------------------------------------------------------------------------------------------------------------------------------------------------------------------------------------------------------PSWSFFQHS--------------------------------------------SGDKAAAAFRRLFQGLPAYSRPSSLRYILTQLSEHRLQLTESQLEAALDQFYVFSRCFGYIIPKPAIPSIFMYAVRQYLLTQSKIPAQPFDDDFLTFLDSHLPGIIRQVAVIDNQVPIDDYLSQKILSVF-KDVRISVEARRVIIRFIQSS--NLLGKSVQGVS-VVARYQKLLRSLFKRYPINTFIFGAKDLASLSSQLQ-EVGLGVDLKYLRDVNIMTYIGLGLTNRFPKPMTAVRF------------------------------------------------------------------------------------------------------------------------------------------------------------------------------------------------------------------------------------------------------------------------------------------------------------------------------------------------------------------------------------------------------------------------------

>Majax_CP100k_homolog5

-------------------------------------------------------------------------------VAAELTVKETTQLRGYLQQRGVK-----HYGALSDLALKAMFHFNLINNFPNVVPATRSGMIQVVSGSLSSLTDEVLPSVSQCGQIAGYLQQSVPELIGG-RVNIDLKSLVASASVILHQRGVKVSLAQLNILLKTGLMAYLKSTAYQSSYSSLIQLISSLDHIDHNLPNILDQKSLLIVRRALENRFNLDR-EIFDKRYKQAVKSFEANRRRILASFNALAYRGPDYEVNMQAVIRTMVVHFP------------------------------------------------------------------------------------------------------------------------------------------------------------------------------------------------------------------------------------------------------------------------------------------------------------------------------------------------------------------------------------------------------------------------------------------------------------------------------------------------------------------------------------------------------------------------------------------------------------------------------------------------------------------------------------------------------------------------------------------------------------------------------------------------------------------------------------------------------------------------------------------------------------------------------------------------------

>Mlong_CP100k_homolog1

--------------------------------------------------------------SSFAVFQRRSCGCRGNPVSRRLSAEELSQLRGLVRQQGVT-----HYRTLSDRALQAVFRFSLLNNFPAVVPSSRSGVLQLVAESLSSLTDAVVPSQNQVSHVVNYLKGSIPVLSSG-SVSIDLKSLVASASI--------------------------------------------------------------------------------------------------------------------------------------------------------------------------------------------------------------------------------------------------------------------------------------------------------------------------------------------------------------------------------------------------------------------------------------------------------------------------------------------------------------------------------------------------------------------------------------------------------------------------------------------------------------------------------------------------------------------------------------------------------------------------------------------------------------------------------------------------------------------------------------------------------------------------------------------------------------------------------------------------------------------------------------------------------------------------------------

>Mlong_CP100k_homolog2

---------------------------------------------------------------------------------------------------------------------------------------------------------------------------------------------------------------------------------------------------------------------------------------------------------------------------------------------------------------------------------------------------------------------------------------------------------------------------------------------------------------FHGLQGSSKEITDRIVSGQGEKGYVKNIVDLKPEIVPGSVPTYNTFEYEGVILSALELQEVAGTLARRFHYLKKPSLQLPLLRILVRAKIVTDTDTRAAVAFQRLFQGLPLYQSPS------------------------------------------------------------------------------------------------------------------------------------------------------------------------------------------------------------------------------------------------------------------------------------------------------------------------------------------------------------------------------------------------------------------------------------------------------------------------------------------------------------------------------------------------------------------------------------------------------------------------------------------------------

>Mlong_CP100k_homolog3

------------------------------------------------------------------------------------------------------------------------------------------------------------------SHVVNYLEGSIPVLSSG-SVSIDLKSLVASASIVLHQRGVRVDVSQLNVLLGLSLESYLQSSVYQSSLSSLAQLVAALDHIDHSLPTVLDHKELVQVRRRLQAQFNNNV-KLFDARYRLAIQEYRTNRHRLLSSFHSLAYRGAGYEVQLLQAARQLVSYLPGLTTATARAVLDVLRIGNSFGGRATPKDLLAS----------------------------------------------------------------------------------------------------------------------------------------------------------------------------------------------------------------------------------------------------------------------------------------------------------------------------------------------------------------------------------------------------------------------------------------------------------------------------------------------------------------------------------------------------------------------------------------------------------------------------------------------------------------------------------------------------------------------------------------------------------------------------------------------------------------------------------------------------------------------------------------------------------------------

>Tform_CP100k_homolog1

---------------------------------------------MMRVPLALTLLLAAFASGSIM-FPRNGCGCLRNPVAAELKTEEITQLRGYIKQRGVT-----HYEAFTDYSLQAIYRFNLLNNFPDVVPSSRSGVIQVVSESLKELTDEVVPSAPTCGKIADYLQGAVPELAGG-GVNIDLKSLVAASAVILHQRGVAIQLSQLNILLKTGLSGYLQSTAYKTSYSALSQLIATLDHIDHNLPNILDQDLLIAVRRKLETRFNLDS-KIFDQRFQQAIRVFEANRQRILESFNSLAFRGPDYEVTIQVVIRETIRLFPGISKTTLRSVLNILQLTNTRGGKATPKDLLAMITIPHLDKSLRTITDVIANRIFLKLPYHHKGLTRVEVHEAYVLFIIGLASQGVQPVQL--QACHEAFVWHTQRFFLGTRSYTVEAYILYVIRVVVPSIPRGSVGFRLHLFDASIVIDN-VLVPEPLQSIYKEGRQTIIERVRGLQGSSEDITRRLLTGEGEKPFVKNIVDLRPPIKTGPLPTYSKFEYEGVILSVAHLQAIAYELQRRFDQLKQPQLQLPLLRVLIRAHVVRGSGDKAAAAFRRLFSGLPRYVAPQDVAGIITQLSERRLQLTRTQVLAGLQQFFVASRCLGHVIPPKSLPGVFVYTITQYIQTLSKIPAQPFDYRFLQYLYQRLASIIQQVVLINRSVPVVGRQVESIFSVF-GRVRISLRCQRTIVRFIDNS--GLIKRPMKGSSTAVVVYRQLLATMLKRYPVGVFVLSEKELTILRVELSTKYRISITTQYLRDANIMSFVGLGLLGRLKPSLTVVQYRQVLVVSIRSFLRINKVSNIPTSDYFRVLF----RTQHVKTPSLP---VPQPPVIQTPKVYVPPPIYILRGLTLTVVQVREIVAVLRVRFTFVSLDNVQAILAHTVLLLRANGKPVDQKNAYEVLSSYYSSLSKNLAISGVDIDALLKTIDERLVDATISGTGIQSGLVELFLHMSFLKMPIPG-PEVRNEFFSFCIGAYGQVQVRRQLPFGKSFYEFLSGFLPKLPGYLKPFPLFSGPKIFDAFSAQLK-TRIAPSDIRLLIQVIRRTTRVKSSSFNLGYLLKIISR--AKPTPITAPLDPKELTGLLDYVKAQKLKVTQAELTRAFAICRLTVRLSVRKVT--RVQVLKIFQRLVQTTVKKYNSLLVVSLVEQLVVSFRSVKVIRPVIPFPRPTR--FPVKRAGGFFD*---

>Wmill_CP100k_homolog1

-------------------------------------------------------------------------------------------------------------------------------------------------------------------------------------------------------------------------------------------------------------------------------------------------------------------------------------------------------------------------------------------------------------------------------------------------------------------------------------------------------------------------------------------------------------------------------------------------------------------------------------------------------------------------------------VVGQYLSTLPTIPKQPFDYNFLEFLHFRLASIIEQLSAVGSQSVIDDYAIYEIFSVF-GRTGLSVYAKRIIIKYINEY--EILPKEAQ-----IAEYQQLIKSVMTKCSVNSLILNEKQLNSILSNLYKSKGIKIELSMLIDINYMAYFAVCQSGSYRPAMHNYLYRRS---------------------------------------------------------------------------------------------------------------------------------------------------------------------------------------------------------------------------------------------------------------------------------------------------------------------------------------------------------------------------------------------------------------------------

>Wmill_CP100k_homolog2

------------------------------------------------------------------------------------------------------------------------------------------------------------------------------------------------------------------------------------------------------------------------------------------------------------------------------------------------------------------------------------------------------------------------------------------------------------------------------------------------------------------------------------------------------------------------------------------------------------------------------------------------------------------------------------------------------------------------QVKIQQKLP--------------------------------------------------------------------------------------------------------------------------------------------------------------------------VSRLPLIQYR-RKPERPCYIIPGIILYKEQLRQLVTVILPRFAFVNMYNIRSIVAHTILILRAR-YRVTQENCYERLKEYYKSLPVN-ALKAFDSYKLL---------------------------------------------------------------------------------------------------------------------------------------------------------------------------------------------------------------------------------------------------------------------------

>Wmill_CP100k_homolog3

----------------------------------------------------------------------------------------------------------------------------------------------------------------------------------------------------------------------------------------------------------------------------------------------------------------------------------------------------------------------------------------------------------------------------------------------------------------------------------------------------------------------------------GEKDYVKNIVNLKPAITAGPVPTYDAFEYKNVLLSSQHMQSIALELEKRFEGLKKPSLRLPLLQILVRADIITDTGDKAAAAFLRLFQGLPKFSRPSSLSYIM------------------------------------------------------------------------------------------------------------------------------------------------------------------------------------------------------------------------------------------------------------------------------------------------------------------------------------------------------------------------------------------------------------------------------------------------------------------------------------------------------------------------------------------------------------------------------------------------------------------------------------------------

**File S6.** Alignment of CP19k homolog sequences within the 10 MEME motif regions.

--------------------------------------------------------------------------------

Motif GSATSSSGHEASSTGDGSFKVDNEGGTEIKL MEME-1 sites sorted by position p-value

--------------------------------------------------------------------------------

Sequence name Start P-value Site

------------- ----- --------- -------------------------------

Cmala_16562_c0_g1_i1.p1 163 1.82e-24 KLPTIIDSSK GAAKSSSGHDASTAGEGTFKTINIGGTEVRL DDLSPDLDIE

Cmala_52758_c0_g1_i1.p1 159 1.02e-23 KTIRLVDAAS GAASSSSGHKASSAAHGTFKIANFGGTEIKL KGPFDF*

Tform_TR66686|c0_g1_i1.p 178 1.67e-23 NLFQVEQVQR AAAKSASGHEASSEGLGSFNVLNLGSTEIKH SDFPSLPPPA

Cmala_60481_c0_g1_i1.p3 66 3.20e-23 GAIRAEQSED GTASSTSGHDASSTGDGRFKTRNEASTEVKQ NAEHAPK*

Cmala_19565_c0_g1_i1.p1 356 7.09e-23 RVVRIVKTDE AKASSNQGHQASSTGDGAFSTINLGGTEIKL DDPLKG*

Aamph_AQA26372.1 397 7.09e-23 PGLDLGSKAK ASGTSSSGHKASSSGPGRFITSNEVGTEIKL TTPELDLETI

Cmala_12977_c0_g1_i1.p1 170 4.42e-22 KVLRVKAAEK ASVSSSSGHKSSTTGSGSFKVINKGGTDIKL ELPELDIEVA

Cmala_34884_c0_g1_i1.p1 80 5.12e-22 KVARIVDVNK GSATSSSGHEASSTGEGRLKTINIGGTKVDL DGLRHDVDIG

Cmala_19296_c0_g1_i4.p1 161 3.79e-21 KKIKILEKQE RSGTSSSGHEASSTGDGTFSVDQKGKTVIKL KGPLVGVISP

Ctest_87774_c0_g1_i1.p1 375 5.00e-21 NLIHVQSVKK GSGTSSSGHKASSTGLGKFGTTNIASTDIKL QTPDLDLSVP

Aamph_AKZ20819.1 167 5.00e-21 EVIKIKEKKQ GTATSSSGHKGSGVGDSLLKVVNEAETELKL KGLKLD

Tform_TR71863|c0_g1_i1.p 166 7.53e-21 GLFKVKEVKR GSATSSNSHKASGSGKSLFKVLNLGETELKL EGTLSDELDD

Majex_16151_c1_g1_i1.p1 384 7.53e-21 PGFNIRSKKK VSGSSSTGHEASSAGDGEFVAKNLAGTEIKL LSPELSLDEI

Tform_TR63530|c0_g1_i1.p 192 1.92e-20 KAVKVRVDKR AGASSSTGHQGSTKGTGAFGIDNFGSTELKR ANPAPEPTNP

Cmala_19565_c0_g1_i1.p1 180 2.86e-20 QLVAVVASEE SKATSSSGHEASSTGPGSFKTINLAGTGIAL TPLPNPLGGG

Cmala_16562_c0_g1_i1.p1 336 3.25e-20 KTVRIVDSGK GSAQSSSGQEASSTGKGSFRALNVGGTDVKL NGVKPGIDLS

Cmala_16562_c0_g1_i1.p1 503 7.04e-20 YPVKSKHSEQ GSASSSSGHDASSSDHGSFQTKNKGKTTIKS GDVKVSPKKG

Tform_TR63530|c0_g1_i1.p 367 1.92e-19 KEVRLVNNKK ATAVSSSGFTGSGSGDSALNVVNEGETEVKL NKIDLNAGIP

Cmala_23239_c0_g1_i1.p2 191 4.01e-19 KAARIADSAQ GSARSTSRHDASSTGDGTFGISNVGGTSLRL GGVGSPDLDF

Tform_TR66686|c0_g1_i1.p 384 4.53e-19 HLVNIQGIQK AAGTSSTGHKASSTGPGKFATTNIAGTDIQL KTPDISLVAP

Tform_TR57313|c0_g1_i1.p 163 6.50e-19 NLLKIKQVKK GSATTSSGHKASGSRESVFGVENRGGTEITL DELDVPKIPT

Aamph_110324_c0_g1_i4.p1 226 1.32e-18 GLVTFSSILR GRGTSSSGHSGSSSGDGELDVSNESGTQIIF SQLTTPRPRG

Mrosa_BAE94409.1 165 2.66e-18 DLFDTKKVEK GTVTSSSSHQGSGAGDSIFEILNEAESKIKK SGD

Aamph_AQA26373.1 363 2.66e-18 KQVQLVNSQK TTASTSSGLAASSKGDGRFRVDDKRETEVRV KPVDLNSEAL

Aamph_102386_c0_g1_i3.p2 129 2.66e-18 KQVQLVNSQK TTASTSSGLAASSKGDGRFRVDDKRETEVRV KPVDLNSEAL

Aamph_AQA26373.1 188 2.98e-18 HEIAVSVDKK AGASSSSGHQSATRGSGSIGVENVGGTELRR VNPARDPTDP

Aamph_Unigene15903_Ba_mi 61 2.98e-18 QEIAVSVDKK AGASSSSGHQSATRGSGSIGVENVGGTELRR VNPARDPTDP

Cmala_12977_c0_g1_i1.p1 340 4.70e-18 KRVRIVGSDR SSAKASSKHEASSSGLGDFKSATEVGTEIKL DPLKL*

Ctest_96511_c0_g1_i1.p1 365 1.15e-17 KEVRLVNNKK ATAVSTSGFSGSGSGKSTLNVVNEGETEVKL AKIDLNTSMP

Ctest_96511_c0_g1_i1.p1 190 1.15e-17 KALKVRVAKK AGASASSGHRGSTKDVGAFTIENLGGTKLRR VNTVLNPTNP

Aamph_AQA26372.1 173 1.15e-17 NLYKVEKVDR TAVKSASGHEASSRRFGTFNVLNLGSTAINN PGLIALPAQA

Majex_16151_c1_g1_i1.p1 177 1.44e-17 NSFQVQHVER TAAESTSGHEASIEGFGTFGVLNFGTTITKK MRVPKVPKSP

Tform_TR63517|c0_g1_i1.p 178 2.00e-17 GLAVIKRVLK GGTTSSSGHKGSGSEDSVFKVANQGGTKIVF DKLTPPTLPP

Majex_56716_c0_g1_i1.p1 194 2.77e-17 LQFMTRVAKR AGASSSTGHQGSTKDNGALTIDNKGATQLRR LKPDLDPTDP

Ctest_86728_c0_g1_i1.p1 202 2.77e-17 GLATIKNILQ GGAKSSSGHKGSGSGNSALDVVNQSGTSIVF DKLSPPAAPP

Cmala_13810_c0_g1_i1.p2 163 2.78e-16 GLVTAVKKVK AQGTSSSGHTGSSTGFGDFKFKGGSVTDIKL PSAL*

Aamph_AQA26371.1 133 4.17e-16 GHAKVAGALK GSATSSSGLDATSSGDSKFKGANAGKTSVKF NVPSPDDALP

Aamph_AQA26371.1 313 5.64e-16 GKFSIKGKHK GAAKGSIGHSASTDGTGSFSAINAVGTDVKF DVPSPDLALP

Majex_56716_c0_g1_i1.p1 371 1.37e-15 KQVRLVNNQK TTAATSSGLSVSSSGNGTFNAQNSRETEVKV VPLNLDANVL

Ctest_87774_c0_g1_i1.p1 188 2.70e-15 NLFQVEQIRK SAAKSGSKHEASTKGLGSFRTLNLGSSSIQG SGLGSLPLPG

Cmala_61689_c0_g1_i1.p1 174 3.96e-15 RKVDIGQAAQ AAGTSSSNHRGSGNGDSKINVKQQSETKVKI KGPLAG*

Cmite_44054_c0_g1_i1.p2 169 6.18e-14 KVVKIVDTGK GTSTGSTGHDGSTTGFGTFHVQQNTTSVIKL KPPLQG*

Cmala_16562_c0_g1_i1.p1 420 8.00e-13 KAVDLKLTEK GAGNAGTSAQAHSAGNGAFKQDAKAKTDIKS NKDGLTVATE

Aamph_AQA26376.1 337 7.49e-12 GADSTISERM SVAEARQSHLASSRGQGNFSVSNEAQMVTEV QKPNA

Cmala_19565_c0_g1_i1.p1 268 2.72e-11 SGGGVQERKA AAASSGAAGTAASAGNGDFFQDTVAKTQIVN SEDGLVVKTG

Aamph_AQA26376.1 169 2.54e-10 EGGAIRRSKR TDEAANIVQSGASKNKGFFTFRNLADSEVKR SAEAGPAPSR

Ctest_86728_c0_g1_i1.p1 113 5.41e-10 RGPQLAAQLA AAGNSGVSGTGVSAGNGIFQQGVKAATEVKS GGNGVDVTTA

Cmala_12977_c0_g1_i1.p1 252 4.69e-09 DLGKIKGSKA TAANAGASATSVSTGLGAFKHRTTGRTRVAS SKDKVKVTSR

--------------------------------------------------------------------------------

--------------------------------------------------------------------------------

Motif GLSIDSKLKQAGKTSGGASVSSTGSTQGS MEME-2 sites sorted by position p-value

--------------------------------------------------------------------------------

Sequence name Start P-value Site

------------- ----- --------- -----------------------------

Tform_TR71863|c0_g1_i1.p 32 4.59e-24 GKPATALPPC DLKIVSKLKQAGITEGGAAVSTTGSTQGS GVIKCVFKSP

Aamph_AQA26371.1 2 5.21e-22 L GLSSKSRQRQTGYTRGGAAVSSTGATQGA GSLDLAIDGP

Tform_TR63530|c0_g1_i1.p 57 8.32e-22 KAPEAPKPDF GFQIRSRQSQSGSTSGGASVSSTGSTQGS ASNSVELSGI

Tform_TR63517|c0_g1_i1.p 43 3.71e-21 TKPPTLPYCD DLKTISKLKQAGFTKGGAAVSSSSSTQGS ASVKCIVRTP

Ctest_96511_c0_g1_i1.p1 55 4.28e-21 APPADLRPDL GVQIRSRQTQTGSTSGGASVSATGSTQGS ASNSIKLAGL

Majex_56716_c0_g1_i1.p1 61 5.70e-21 GKEEKLKPDF GFAIESKQLQTGSTSGGASISSTGSTQGS VSSIMDLSTD

Cmala_13810_c0_g1_i1.p2 29 1.32e-20 ASGGKSKRKC GVSILSKLHQSGSTSGGGAVSAHGKTQGS ARFRCRVQGP

Cmala_19565_c0_g1_i1.p1 46 2.59e-20 PPLPIVVGRS GTAINSKLTQVGHTSGGAVVSSTGSTQGS SRFTRIVAGP

Majex_16151_c1_g1_i1.p1 254 5.00e-20 TAAPTTVSPQ ELSSKSKMKQAGKTSGTGAVTASGATQGS TTSETDVKTP

Cmala_16562_c0_g1_i1.p1 201 5.00e-20 VRLDDLSPDL DIEVKSKQGQAGRTTRGGNVNSHGSTQGS ADSKSGFKAG

Tform_TR63530|c0_g1_i1.p 235 5.69e-20 PAPEPTNPND SLSSDSQLKQVGKTTGTSSLSATGSTQGS GGAKFGFLTP

Tform_TR66686|c0_g1_i1.p 250 6.48e-20 EAPTTASPKF GISTNSKLKQAGKTTGSGAISSTGATQGS ASSKSGLKTR

Aamph_AQA26373.1 53 1.38e-19 EGSGQLKPDF GFEIKSRQSQSGTTSGGASVSSTGSSQGA VTGALNLATE

Ctest_87774_c0_g1_i1.p1 241 2.01e-19 AAPVTLDPKG SISSDSSLKQSGKTTGGGSISSLGATQGS TSLEAAGNSP

Mrosa_BAE94409.1 31 2.27e-19 VDAAPVPPPC DLGIASKVKQKGVTGGGASVSTTSATQGS GTTNCVTRTP

Ctest_96511_c0_g1_i1.p1 233 8.44e-19 TVLNPTNPND SLSSDSGLKQVGKTTGTSSLSATGSTQGS GSAQVGLWTP

Cmala_23239_c0_g1_i1.p2 61 1.06e-18 ELPSGGSARS GVRIKSRLKQEGSTSGSGSVSSKGATRGS SKFKTSLTTP

Majex_56716_c0_g1_i1.p1 237 1.89e-18 PDLDPTDPNE SLSSSSQLKQTGRTTGMSSLSATGSTQDS GGTRLTRWSP

Cmala_19296_c0_g1_i4.p1 31 3.30e-18 RRRPRPKPIC NPSTLSKLGQKGHTSGGGAVSASTSTQGS GSINCVFKGP

Aamph_AQA26372.1 267 3.30e-18 TKPAAPQPKK ELSSESKAKQAGKTTGLGAVSSTGATQGA AQSQTSVETP

Cmala_16562_c0_g1_i1.p1 374 4.12e-18 VKLNGVKPGI DLSIESRQKQAGVTSKGGAVSSQGKSRGA GSDRFKLKAV

Cmala_19565_c0_g1_i1.p1 222 6.37e-18 PLPNPLGGGT DVTITSKQKQGGRTTHGGALSATGATKGS VKTDSSRSGG

Cmala_61689_c0_g1_i1.p1 44 1.35e-17 RRRPKSKPTC NVSTLSKLGQRGHTSGGGAVSGTTSSQGS GSIACVFRGP

Aamph_AQA26373.1 231 1.66e-17 PARDPTDPDD SLSSDSQLRQTGKTTGTSSLTASGSTQGG GGARFNLWSP

Aamph_Unigene15903_Ba_mi 104 1.66e-17 PARDPTDPDD SLSSDSQLRQTGKTTGTSSLTASGSTQGG GGARFNLWSP

Tform_TR57313|c0_g1_i1.p 29 2.05e-17 PSGKRKATPC GLLTISNLTQKAITKGGAAVSSTGATQGS GSVRCITVTP

Cmite_44054_c0_g1_i1.p2 35 3.79e-17 TPVLGTPPNC NMSSHSGLKQSGQTTGTGSVSTTGSTTGS VSGLCAFTGP

Cmala_12977_c0_g1_i1.p1 206 4.20e-17 TDIKLELPEL DIEVASSQQQAGGTTRGGSVSAEGATRGS AVGSSGLDLG

Cmala_52758_c0_g1_i1.p1 24 8.47e-17 ALTVPVPRRP GASVTSRLRQTGRTGGGAVVSSSGSSSGS VSTSSSSRRA

Cmala_12977_c0_g1_i1.p1 36 1.52e-16 TPKPGRRPDS SGAVKSKLSQSGHTTGGATVSTQGSTKGS FRIKATIKGP

Ctest_86728_c0_g1_i1.p1 67 2.47e-16 KRPAALPFCD DLKTSSVVQQGGFTSGGAALSSRSATQGS AAVKCVVRGP

Cmala_34884_c0_g1_i1.p1 118 3.28e-16 VDLDGLRHDV DIGVKSKQGQIGRTADGASLNARGATQGE AVSSTGLN

Cmala_23239_c0_g1_i1.p2 230 1.00e-15 RLGGVGSPDL DFSVVSRRRQAGSTSAGGSVASKGATRGS SKFKTSLTTP

Cmala_16562_c0_g1_i1.p1 31 2.06e-15 SKKGDKDDKS GLTTSSEVSQSGITQGGGVVSSKGSSKGS TKSSSSFKAP

Aamph_AKZ20819.1 32 3.21e-15 TLGAPVPPPC DLKIKSKVGQAAVTKGGAAVSTTGSSGGT GTVHCVVVGP

Aamph_AQA26376.1 38 1.17e-14 APSPAPLPDV RLRVESKQSHRGSTRGGASVSSRSEARGG ASERLRLTDL

Tform_TR66686|c0_g1_i1.p 44 1.51e-14 STAAPARSSR LIDVDSKLTSNVITGGGAVVQTSGSTSGA GSVTRVVSGP

Ctest_87774_c0_g1_i1.p1 54 2.70e-14 KAKVPVASNP LVGVDSKLTSNVVTSGGAKVQTTGSTSGS GSLTTVITGP

Majex_16151_c1_g1_i1.p1 43 4.77e-14 PPLPSSSSES LIGIGSKLSSSIVTGGGAVVNTRGSTSGS ATLHTSYKGP

Aamph_AQA26371.1 369 2.31e-13 PSAPADTPVF KLTSTSDILQKGGSQGGAAVSGAGSTQGS GLGTVDLDTP

Aamph_110324_c0_g1_i4.p1 91 3.14e-13 TSPPALPLCG NLETAAEMKQLGFSRGTGVFSSSSSSRGS ANTKCRSRSD

Aamph_AQA26371.1 181 4.94e-13 ALPSGVAPNI GVDSDSLLKQQGGAVGDASISTNGGTQGG GSLVAGLRAP

Aamph_AQA26372.1 39 6.18e-13 STPPPSSSGP VIDIDSKLASAVVTGGGAQVTTSGGTSGA ASVTSTVRGP

--------------------------------------------------------------------------------

--------------------------------------------------------------------------------

Motif KGPGLKLKKAAVGNSGVSGTGVSSGBGAFKQKAQAKTVVK MEME-3 sites sorted by position p-value

--------------------------------------------------------------------------------

Sequence name Start P-value Site

------------- ----- --------- ----------------------------------------

Tform_TR63530|c0_g1_i1.p 271 1.65e-29 QGSGGAKFGF LTPIVKRKKDFAGNSGVSGNGASSGNGFFVQGVQANTELV STKDGLKVRT

Aamph_AQA26373.1 267 1.19e-28 QGGGGARFNL WSPTFNNSRDFAGNTNVAGNGIASGNGFFVQGVQANTELV STKDGLKVKT

Aamph_102386_c0_g1_i3.p2 33 1.19e-28 QGGGGARFNL WSPTFNNSRDFAGNTNVAGNGIASGNGFFVQGVQANTELV STKDGLKVKT

Ctest_96511_c0_g1_i1.p1 269 2.88e-28 QGSGSAQVGL WTPSLDRKKDVSGNTGVSGNGVSTGNGFFVQGVQAQTELV STKDGLKVKT

Tform_TR63517|c0_g1_i1.p 79 2.96e-24 QGSASVKCIV RTPKSQTKLNAAGNSGVSGAGVSASGGIYKQGVEAATEVK TSNEGVEVKT

Cmala_19296_c0_g1_i4.p1 67 2.96e-24 QGSGSINCVF KGPNLKVDTGAAANSGVAGTGVSAGEGAFGQNVRAGSGVK SVPGITKVTT

Majex_56716_c0_g1_i1.p1 275 7.46e-24 SGGTRLTRWS PSQGLNNSRDLSGNTGVSGNGAATGNGFFVQGVQANTELI STKDSLKVRT

Ctest_87774_c0_g1_i1.p1 277 1.18e-23 QGSTSLEAAG NSPGGGIKVSGVANSGVSGAGVSSGNGVYNQGAQGKTVVD GNKNGGKVTT

Tform_TR71863|c0_g1_i1.p 68 1.47e-23 QGSGVIKCVF KSPTSVVKKAATGNSGVSGASISADNGAFKNLVEALTDVK TTKKGTKVKT

Aamph_AQA26373.1 89 1.32e-22 QGAVTGALNL ATEGYKLDLSAVGNSGVSGSGVSIGDSGFRQKTQTNSEAG SKGTKRAQVV

Cmala_13810_c0_g1_i1.p2 65 2.02e-22 QGSARFRCRV QGPGFLVDSAGVGSAGVSGTGVSSGLGAFDQDAAALTEII SKPGGSKVLT

Tform_TR66686|c0_g1_i1.p 286 7.08e-22 QGSASSKSGL KTRAGGAKQSAVANSGVTGAGVSSGDGAYKQGAEGKTVVA ATKEGVKVTT

Cmala_12977_c0_g1_i1.p1 72 7.08e-22 KGSFRIKATI KGPGKTVDQNSAANAGVSGSSVSAKEGVFAQRSGAKTEIT NKKGRLSAET

Aamph_AQA26371.1 405 7.85e-22 QGSGLGTVDL DTPHLQLDGEVVANSGVSGTAGSQGHGLFGQDANARTDAI ANADGIETRT

Cmala_19565_c0_g1_i1.p1 82 9.63e-22 QGSSRFTRIV AGPGGTVQQAGAGSAGVSGTSVSSGHGVFVQRGQAKTTIK AGPDGIDVKT

Majex_16151_c1_g1_i1.p1 290 1.07e-21 QGSTTSETDV KTPTGNAKQAAVANSGVSGTGVSSGESGFMHVAVGGTVVF KTKDGAKVVT

Mrosa_BAE94409.1 67 2.17e-21 QGSGTTNCVT RTPNSVEKKNVAGNTGVTATSVSAGDGAFGNLAAALTLVE DTEDGLGVKT

Tform_TR57313|c0_g1_i1.p 65 3.94e-21 QGSGSVRCIT VTPVSVEKLAAVGNSGVSGSGVSAGNGILKHIVTSGTVAK RTKDGFKAKS

Tform_TR63530|c0_g1_i1.p 93 4.80e-21 QGSASNSVEL SGIGFVLKKSAVGNGGASGSSVSSGDAAFNQNAATRTVAV SSGTRQAEVL

Cmala_16562_c0_g1_i1.p1 67 7.11e-21 KGSTKSSSSF KAPDVKIKRNLRASSGVSGSGASSGDSAFGQKAGSKSVVS IDKDEIIVRT

Aamph_110324_c0_g1_i4.p1 127 1.05e-20 RGSANTKCRS RSDTSENALAAAGNAAVTGAGNSVGGGAFKQGARANTEVK QTPDGVSVNT

Cmala_52758_c0_g1_i1.p1 61 1.16e-20 GSVSTSSSSR RAAGSSLQVAGAANAGVSGTAVSRGVGAFAQRAKAKTVIK ANKKGLSVKT

Ctest_87774_c0_g1_i1.p1 90 1.87e-20 SGSGSLTTVI TGPGLTSRTTASGSGGVSGTSASAGNGLSKQKSRAKTVVT DSNGGPKVQT

Tform_TR66686|c0_g1_i1.p 80 2.49e-20 SGAGSVTRVV SGPGLVFRTTAVGSGGVSGQSASSGDGLAAQLSRARTVVV DTEDGTKVQT

Cmite_44054_c0_g1_i1.p2 71 5.29e-20 TGSVSGLCAF TGPNSGTQDTGAGNSGVSGSGVSAGNGIFGQTVDAGAGVG TAPDKTVVQT

Ctest_96511_c0_g1_i1.p1 91 9.23e-20 QGSASNSIKL AGLGYLLEKASIGNGGVSGSSVSSGDATFTQKAEADTVSN SAGTRAAKVG

Aamph_AQA26372.1 303 1.01e-19 QGAAQSQTSV ETPNGSAKQAAVVNSGVGLTGVSSGTGLFNQKAHGKTAVT RTGESIDVTS

Aamph_AKZ20819.1 68 1.22e-19 GGTGTVHCVV VGPNKIVKKAAVGNTGVTGAGATAGDGILKNLVKGVTEVK TTKDGTKVKT

Cmala_61689_c0_g1_i1.p1 80 1.33e-19 QGSGSIACVF RGPGLSIDAREAARSGVAGSAASNGHGAFGQTAGANSGVA FVPGLASVTS

Cmala_23239_c0_g1_i1.p2 97 1.46e-19 RGSSKFKTSL TTPDIKIDFSGAANAGLSGNSVSTGRGAFSQKSQSTSGVS VSKTGTDVTT

Majex_56716_c0_g1_i1.p1 97 2.48e-18 QGSVSSIMDL STDIYNLNISAVGNGGLSSSSASSGVGSFSQKAQINTDAG SIGTRRADLG

Aamph_AQA26371.1 217 1.86e-17 QGGGSLVAGL RAPGLQIDGSAAAKSGVSGAGALVGDGGFAQESIAGTSSS GRSDGLSVKS

Aamph_AQA26372.1 75 5.79e-17 SGAASVTSTV RGPGSVSTLTAVGQGGISGSSATAGNSASLQKGRSKTVVE ATTEGTQVKT

Aamph_AQA26371.1 39 1.09e-16 GAGSLDLAID GPGGFKARSKALVNSGVSGAAVAAGNGGFKQKSESETVGT VGLQGLDIST

Aamph_AQA26376.1 243 1.18e-15 NGTAANLASS TEDSPGSRRDLVGRSNVAGNASSIGHGFFRHSVKSGSDLA ANRTALRAQA

Cmala_16562_c0_g1_i1.p1 238 2.49e-15 GSADSKSGFK AGKKVTNRNNAAVNAGAAAQAASTDNGAFRQNAKSFSGAK TDKDGISVRT

Majex_16151_c1_g1_i1.p1 79 2.46e-14 SGSATLHTSY KGPGESSETTTVGKTGLSETSAAASGGVSIQKNRAKTVVD STEEGIEVKT

Aamph_AQA26376.1 74 6.33e-12 RGGASERLRL TDLDLSLEKVARAETRAVTRSTSSGAGAYDQFADTKGDVN SIAARKATSS

--------------------------------------------------------------------------------

--------------------------------------------------------------------------------

Motif TKDGLKVKTGTKGKGTTGGTAGTVQKAGANGGATGKKAVTL MEME-4 sites sorted by position p-value

--------------------------------------------------------------------------------

Sequence name Start P-value Site

------------- ----- --------- -----------------------------------------

Tform_TR63530|c0_g1_i1.p 312 1.28e-31 GVQANTELVS TKDGLKVRTGTRGEGTTGGNAGIVEKAGANGKATDVAIVTL ADGTKEVRLV

Aamph_AQA26373.1 308 1.01e-29 GVQANTELVS TKDGLKVKTGTRGAGTTGGNAGLIEKAGANGKATDVAIITL ADGSKQVQLV

Aamph_102386_c0_g1_i3.p2 74 1.01e-29 GVQANTELVS TKDGLKVKTGTRGAGTTGGNAGLIEKAGANGKATDVAIITL ADGSKQVQLV

Ctest_96511_c0_g1_i1.p1 310 9.29e-29 GVQAQTELVS TKDGLKVKTGTRGEGSTEGDAGIVEKAGADGKATDVAIVTL ADGTKEVRLV

Tform_TR66686|c0_g1_i1.p 121 3.90e-27 LSRARTVVVD TEDGTKVQTGTEGKGTTLGTAAAAQKTGANGGVNTLKAIKF VETDGKNLFQ

Cmala_19565_c0_g1_i1.p1 123 1.17e-26 RGQAKTTIKA GPDGIDVKTGTQGEGFTDGTAGNIQKAGAGGGATQKQTVVT VVPKGHQLVA

Cmala_16562_c0_g1_i1.p1 279 1.20e-25 NAKSFSGAKT DKDGISVRTGNIGEGNTRGHSGVQQKTGANAGANKKQAIII TLPDGKKTVR

Majex_16151_c1_g1_i1.p1 120 4.31e-24 KNRAKTVVDS TEEGIEVKTGTEGKGITDGKAAGTQKAGAEGGAKTVDTTKF VYTDGENSFQ

Tform_TR66686|c0_g1_i1.p 327 4.79e-24 GAEGKTVVAA TKEGVKVTTGSTGFGFTDGTAGTLQNVAANGGFTGLSTLNI KLPTGKHLVN

Aamph_Unigene15903_Ba_mi 4 1.48e-23 GSK GTRRAQVVTGTKGQSISKGNAGTVQKAGANVGFQGAQAVRF TQPGQGQEIA

Cmala_52758_c0_g1_i1.p1 102 2.44e-23 RAKAKTVIKA NKKGLSVKTSTAGRARTRGKAGAVQQAGANGGLTLKRVVIV TLPNGLKTIR

Aamph_AQA26373.1 131 5.38e-23 TQTNSEAGSK GTKRAQVVTGTKGQSISKGNAGTVQKAGANVGFQGAQAVRF TQPGQGHEIA

Aamph_AQA26372.1 116 1.06e-22 KGRSKTVVEA TTEGTQVKTGTQGKGITSGEAVANQKAGAEGGAQRVEAVKY VESDGKNLYK

Majex_56716_c0_g1_i1.p1 316 1.29e-22 GVQANTELIS TKDSLKVRTGIRGGGTTGGSAGIVEKAGAKGKATDVRIVTL ADGSKQVRLV

Ctest_87774_c0_g1_i1.p1 318 5.81e-22 GAQGKTVVDG NKNGGKVTTGSTGFGFTDGTAGALQEVAANGGFTGLGTLDL KLPSGNNLIH

Mrosa_BAE94409.1 108 1.01e-21 LAAALTLVED TEDGLGVKTKNGGKGFSEGTAAISQTAGANGGATVKKAKLD LLTDGEDLFD

Cmala_16562_c0_g1_i1.p1 108 1.59e-21 KAGSKSVVSI DKDEIIVRTGTKGKGFSTGDAGAIQSAGATAGGKQSTIIKL PGGKKLPTII

Cmala_60481_c0_g1_i1.p3 9 2.49e-21 AGKTDVKS TDNGISVSTGSAGKGKTDGDAAVLQKTGANGGARQQRSLSG SQGAGKGAIR

Aamph_AKZ20819.1 109 2.49e-21 LVKGVTEVKT TKDGTKVKTKTAGKAGTGGTATIFQVADANGGVTEKSIKVD HLLTDDFEVI

Tform_TR63517|c0_g1_i1.p 120 3.89e-21 GVEAATEVKT SNEGVEVKTESQGTGGSAGGAAINQNAGANGGAKLNIVGVD LLKNGKLGLA

Tform_TR71863|c0_g1_i1.p 109 6.60e-21 LVEALTDVKT TKKGTKVKTESAGEGATTGKATTLQNAAANGAATLTTAEID LLTSGKGLFK

Ctest_96511_c0_g1_i1.p1 133 1.86e-20 AEADTVSNSA GTRAAKVGTSTKGLATTTGKAATVQKTGANAGFRGSQGVIF NQPNSLKALK

Tform_TR63530|c0_g1_i1.p 135 2.03e-20 AATRTVAVSS GTRQAEVLTGTKGQAVTAGKAATVQKTGANIGFLGTQGIVF NQPNGLKAVK

Cmite_44054_c0_g1_i1.p2 112 2.03e-20 TVDAGAGVGT APDKTVVQTATGGSGGSAGTAGTVQKAGANVGAEKKKTVIV TLSDGTKVVK

Aamph_AQA26371.1 446 2.03e-20 DANARTDAIA NADGIETRTRTGGRGVTEGRAATAQKTGANGAIKARRDIVA QLPGEALGVS

Cmala_12977_c0_g1_i1.p1 113 2.62e-20 RSGAKTEITN KKGRLSAETTSQGTGITGGGAGTMQNTGANGGANQRTTVTR SLPAGKKVLR

Cmala_12977_c0_g1_i1.p1 283 1.38e-19 RTTGRTRVAS SKDKVKVTSRTGGRGSTGGGAGIVEKSAARGRARHRKAIIV TLPDGKKRVR

Majex_56716_c0_g1_i1.p1 139 2.08e-19 AQINTDAGSI GTRRADLGTGTRGKASSRGNAGTVHKTAANVGLQGLETITF AEPGLQFMTR

Cmala_34884_c0_g1_i1.p1 25 3.95e-19 LFMCSTVVGL GRNALVVKTGTKGKSVTTGGAGTVQGAGADAGGTQEVNITL PGGKKVARIV

Majex_16151_c1_g1_i1.p1 331 5.87e-19 VAVGGTVVFK TKDGAKVVTTSDGFGNTKGASGTIQTVAVNGGLSVLDNIDL DLPGFNIRSK

Cmala_13810_c0_g1_i1.p2 106 9.40e-19 DAAALTEIIS KPGGSKVLTLTEGKGSSGGTAGVLQKAGASGGVSKKKIGIL QKNFLKGLVT

Tform_TR57313|c0_g1_i1.p 106 1.75e-18 IVTSGTVAKR TKDGFKAKSGTSGSGASGGGAGILQDAAANGGTKLEIGELA VLTDGKNLLK

Ctest_87774_c0_g1_i1.p1 131 4.05e-18 KSRAKTVVTD SNGGPKVQTATEGQGNTVGPAAATQSAAADGNVNTLKAIKF VQSDGKNLFQ

Cmala_23239_c0_g1_i1.p2 138 4.05e-18 KSQSTSGVSV SKTGTDVTTGTKSLGATSGGAGSISKAGANAAARQKQAVVV GKKAARIADS

Cmala_19565_c0_g1_i1.p1 299 5.89e-18 DTVAKTQIVN SEDGLVVKTGTKGTGKSGGKSGIQQTAGAAGVGALSRVVIV TLPNGKRVVR

Cmala_16562_c0_g1_i1.p1 451 1.07e-17 DAKAKTDIKS NKDGLTVATETGGRGKTAGDTQIAQGTAANGKADQKKDSRQ KYPVKSKHSE

Ctest_86728_c0_g1_i1.p1 144 2.39e-17 GVKAATEVKS GGNGVDVTTASAGTGKSGGGAAINQSSGANVGANLKIVGAG LLQTSELGLA

Aamph_110324_c0_g1_i4.p1 168 3.19e-17 GARANTEVKQ TPDGVSVNTATGGSGVTAGSSATNQTTDANGGVNVRLLRNA LRQGSDGGLV

Cmala_61689_c0_g1_i1.p1 121 1.87e-16 TAGANSGVAF VPGLASVTSGTGGDGTTLGGAAIAQEAGAHAGAKIGSQIDL HLRKVDIGQA

Aamph_AQA26372.1 344 1.87e-16 KAHGKTAVTR TGESIDVTSISSGLGETDGAAGSIQSVATNGGFTAVDTLNL NLPGLDLGSK

Aamph_AQA26371.1 80 9.54e-16 KSESETVGTV GLQGLDISTSSRGAGKSFGTAGVTQKQGANGGVSGAARVGG KYGHAKVAGA

Cmala_19296_c0_g1_i4.p1 108 1.05e-14 NVRAGSGVKS VPGITKVTTGTAAGGATGGGAAVTNKAGANAGAKTSVEFGR RLKKIKILEK

Aamph_AQA26376.1 284 8.74e-10 SVKSGSDLAA NRTALRAQAATSGDGETRRRAILHQSADSGGAGRHAGRTSS TPGADSTISE

--------------------------------------------------------------------------------

--------------------------------------------------------------------------------

Motif LLVCVAVATAVPLP MEME-5 sites sorted by position p-value

--------------------------------------------------------------------------------

Sequence name Start P-value Site

------------- ----- --------- --------------

Tform_TR66686|c0_g1_i1.p 7 5.21e-17 MLSAHL LLVCVAVATAVPLP YKHYTTTTAA

Tform_TR63517|c0_g1_i1.p 6 5.21e-17 MSVRL LLVCVAVATAVPLP SGKLEPPTTK

Tform_TR57313|c0_g1_i1.p 6 1.11e-16 MLSAR LLVCVAIATAVPLP SGKRKATPCG

Majex_16151_c1_g1_i1.p1 7 8.87e-15 MSSARL LLLCAAAASAVPLP SGFRPPTPTT

Ctest_87774_c0_g1_i1.p1 7 2.12e-14 MLLAHL LLACVAFASAVPVP SNYLPRTTTT

Ctest_96511_c0_g1_i1.p1 7 2.38e-14 MLGVRL LLACVAAACAGPVP TKLQTYIPST

Tform_TR63530|c0_g1_i1.p 7 3.03e-14 MLSVRL LLVWVAVATAGPLP SKLRPEEYTK

Cmala_16562_c0_g1_i1.p1 7 4.30e-14 MLLRFT LLLCVALAVAVPTP SKKGDKDDKS

Cmala_52758_c0_g1_i1.p1 7 5.43e-14 LSSHVL LLFCVAVALTVPVP RRPGASVTSR

Aamph_110324_c0_g1_i4.p1 7 2.98e-13 MQLPTV LLVWVAATTAAPWP QDPGTLPLSP

Cmala_12977_c0_g1_i1.p1 7 3.70e-13 MLSARI FLLCMAVAVAVSVP VPGRKTPKPG

Ctest_86728_c0_g1_i1.p1 9 5.09e-13 MLSSAAHL LLAYCTVATAVPLP AFELGLAYSG

Aamph_AQA26372.1 7 5.09e-13 MVSASL LLLCAAAASAVPLK ATVSTTSPST

Cmala_19565_c0_g1_i1.p1 8 8.58e-13 MMRSPLA LLLCAAAVFAAPVP DHKGIIWLPT

Cmala_19296_c0_g1_i4.p1 6 4.18e-12 MRFFL ILLCVAVALAVPTG NRRRPRPKPI

Majex_56716_c0_g1_i1.p1 7 1.86e-11 MLSAHR LIACAAIAAAAALP IEQKYVHYDP

Aamph_AQA26373.1 7 2.67e-11 MLSLHL LTVCAAVAAAAALP VDPKTVEQPP

Cmite_44054_c0_g1_i1.p2 7 6.45e-11 MLSARL FWICLGAAAAVPLQ LPSLTPVLGT

--------------------------------------------------------------------------------

--------------------------------------------------------------------------------

Motif KPAPTPAPTTTPAPTTTPAPE MEME-6 sites sorted by position p-value

--------------------------------------------------------------------------------

Sequence name Start P-value Site

------------- ----- --------- ---------------------

Tform_TR63530|c0_g1_i1.p 422 2.72e-19 VAAPASNPKL PKYPTPSPTTTPAPTTTPAPP TSSPKYLKNP

Tform_TR63530|c0_g1_i1.p 483 1.39e-18 PTNSLKYPKY HKHPSPAPTTTPAPTTTPAPT SAPSPKYSKG

Aamph_AQA26372.1 235 4.08e-17 VYWNPKNPQP EPEPQPEPHPEPEPEPEPHPK PTKPAAPQPK

Tform_TR63530|c0_g1_i1.p 30 6.19e-15 PSKLRPEEYT KYAPTSAPTTTAAPATTKAPE APKPDFGFQI

Ctest_86728_c0_g1_i1.p1 234 7.48e-15 NQSGTSIVFD KLSPPAAPPTTPAPTTTPAPS TPAPDTGLPD

Aamph_102386_c0_g1_i3.p2 205 7.48e-15 GPSPQPKPDP QPKPDPQPKPEPEPEPKPEPE P

Aamph_110324_c0_g1_i4.p1 53 1.09e-14 RRILRPTPQP TPEPTPEPTPQPTPQPTPEPT PQPTPEPTSP

Ctest_96511_c0_g1_i1.p1 423 1.58e-14 ALPPPELPNF PMTPTSMPSTTPEPTLAPLPV YKKGGK*

Tform_TR66686|c0_g1_i1.p 228 9.54e-14 AGPAPTKKPK HWKPTAAPTTTTEAPTTASPK FGISTNSKLK

Tform_TR66686|c0_g1_i1.p 424 3.18e-13 LKTPDISLVA PLIPTPAPTTTAAPSTAAPKL KFHKW*

Aamph_AQA26372.1 207 1.79e-12 GSTAINNPGL IALPAQAPSSEPEPEPTPVYW NPKNPQPEPE

Majex_16151_c1_g1_i1.p1 233 2.10e-12 SAPALSPEPD YWHDEPKPVTTTAAPTTVSPQ ELSSKSKMKQ

Tform_TR63517|c0_g1_i1.p 210 1.96e-11 NQGGTKIVFD KLTPPTLPPTKPAPTTQAPKL KHLKLH*

Tform_TR63530|c0_g1_i1.p 453 2.45e-11 TSSPKYLKNP KKYFAPAPTATSAPTTTLATP TNSLKYPKYH

Ctest_87774_c0_g1_i1.p1 23 2.84e-11 FASAVPVPSN YLPRTTTTTTTAAPATSRAPP KAKVPVASNP

Ctest_96511_c0_g1_i1.p1 28 3.05e-11 PVPTKLQTYI PSTPTAAPTSTATPPTPAPPA DLRPDLGVQI

Aamph_AQA26373.1 27 7.83e-11 AALPVDPKTV EQPPPPPPPTTPAPSKEGSGQ LKPDFGFEIK

Majex_16151_c1_g1_i1.p1 212 9.02e-11 TTITKKMRVP KVPKSPAPAPTSAPALSPEPD YWHDEPKPVT

Cmala_23239_c0_g1_i1.p2 23 9.02e-11 AAAVAAPTVK HDASTVAPATTAAPATTSAPA KAATSDWELP

Ctest_86728_c0_g1_i1.p1 36 1.38e-10 LGLAYSGLHS TPAPPTSAPTTPAPTTQAPTV KRPAALPFCD

Ctest_96511_c0_g1_i1.p1 398 6.22e-10 EGETEVKLAK IDLNTSMPKFPSVPEALPPPE LPNFPMTPTS

Majex_56716_c0_g1_i1.p1 30 7.59e-10 PIEQKYVHYD PPAATTAAPSTAAPSSSPQPD GKEEKLKPDF

Ctest_87774_c0_g1_i1.p1 426 5.23e-09 LLPPVVPATT TTTTTTTTTTTAATTTTHRPY KKYSK

Majex_56716_c0_g1_i1.p1 417 7.13e-09 DANVLKVSVL GSPAEPIPTAMPVVMPTGGPQ YNVRYISTEL

--------------------------------------------------------------------------------

--------------------------------------------------------------------------------

Motif LPDGKKLVKVVKVKK MEME-7 sites sorted by position p-value

--------------------------------------------------------------------------------

Sequence name Start P-value Site

------------- ----- --------- ---------------

Tform_TR57313|c0_g1_i1.p 148 6.03e-14 TKLEIGELAV LTDGKNLLKIKQVKK GSATTSSGHK

Tform_TR66686|c0_g1_i1.p 163 1.03e-13 VNTLKAIKFV ETDGKNLFQVEQVQR AAAKSASGHE

Aamph_AQA26372.1 158 5.20e-13 AQRVEAVKYV ESDGKNLYKVEKVDR TAVKSASGHE

Majex_16151_c1_g1_i1.p1 162 7.40e-13 AKTVDTTKFV YTDGENSFQVQHVER TAAESTSGHE

Cmala_12977_c0_g1_i1.p1 325 2.86e-12 ARHRKAIIVT LPDGKKRVRIVGSDR SSAKASSKHE

Ctest_87774_c0_g1_i1.p1 173 4.88e-12 VNTLKAIKFV QSDGKNLFQVEQIRK SAAKSGSKHE

Cmala_16562_c0_g1_i1.p1 321 1.11e-11 ANKKQAIIIT LPDGKKTVRIVDSGK GSAQSSSGQE

Cmala_19565_c0_g1_i1.p1 341 1.66e-11 GALSRVVIVT LPNGKRVVRIVKTDE AKASSNQGHQ

Tform_TR71863|c0_g1_i1.p 151 3.28e-11 ATLTTAEIDL LTSGKGLFKVKEVKR GSATSSNSHK

Mrosa_BAE94409.1 150 3.28e-11 ATVKKAKLDL LTDGEDLFDTKKVEK GTVTSSSSHQ

Ctest_87774_c0_g1_i1.p1 360 3.61e-11 FTGLGTLDLK LPSGNNLIHVQSVKK GSGTSSSGHK

Tform_TR66686|c0_g1_i1.p 369 9.14e-11 FTGLSTLNIK LPTGKHLVNIQGIQK AAGTSSTGHK

Cmala_12977_c0_g1_i1.p1 155 4.41e-10 ANQRTTVTRS LPAGKKVLRVKAAEK ASVSSSSGHK

Cmite_44054_c0_g1_i1.p2 154 1.18e-09 AEKKKTVIVT LSDGTKVVKIVDTGK GTSTGSTGHD

Aamph_AKZ20819.1 152 1.38e-09 TEKSIKVDHL LTDDFEVIKIKEKKQ GTATSSSGHK

Tform_TR63530|c0_g1_i1.p 177 1.32e-08 FLGTQGIVFN QPNGLKAVKVRVDKR AGASSSTGHQ

Cmala_52758_c0_g1_i1.p1 144 4.60e-08 LTLKRVVIVT LPNGLKTIRLVDAAS GAASSSSGHK

Cmala_19565_c0_g1_i1.p1 165 5.25e-08 ATQKQTVVTV VPKGHQLVAVVASEE SKATSSSGHE

--------------------------------------------------------------------------------

--------------------------------------------------------------------------------

Motif MLSARL MEME-8 sites sorted by position p-value

--------------------------------------------------------------------------------

Sequence name Start P-value Site

------------- ----- --------- ------

Tform_TR66686|c0_g1_i1.p 1 2.89e-09 . MLSAHL LLVCVAVATA

Cmite_44054_c0_g1_i1.p2 1 1.15e-08 . MLSARL FWICLGAAAA

Tform_TR71863|c0_g1_i1.p 1 1.66e-08 . MLSFRL VLVPVAMAIT

Tform_TR63530|c0_g1_i1.p 1 2.72e-08 . MLSVRL LLVWVAVATA

Aamph_AQA26373.1 1 2.72e-08 . MLSLHL LTVCAAVAAA

Cmala_12977_c0_g1_i1.p1 1 3.16e-08 . MLSARI FLLCMAVAVA

Ctest_87774_c0_g1_i1.p1 1 3.48e-08 . MLLAHL LLACVAFASA

Majex_56716_c0_g1_i1.p1 1 6.17e-08 . MLSAHR LIACAAIAAA

Majex_16151_c1_g1_i1.p1 1 3.17e-07 . MSSARL LLLCAAAASA

Ctest_96511_c0_g1_i1.p1 1 5.03e-07 . MLGVRL LLACVAAACA

Aamph_AQA26372.1 1 1.70e-06 . MVSASL LLLCAAAASA

Aamph_AKZ20819.1 1 1.85e-06 . MVSPRI LLAWAAVGIA

--------------------------------------------------------------------------------

--------------------------------------------------------------------------------

Motif DGSKQVRLVNN MEME-10 sites sorted by position p-value

--------------------------------------------------------------------------------

Sequence name Start P-value Site

------------- ----- --------- -----------

Majex_56716_c0_g1_i1.p1 358 2.61e-14 ATDVRIVTLA DGSKQVRLVNN QKTTAATSSG

Tform_TR63530|c0_g1_i1.p 354 1.18e-13 ATDVAIVTLA DGTKEVRLVNN KKATAVSSSG

Ctest_96511_c0_g1_i1.p1 352 1.18e-13 ATDVAIVTLA DGTKEVRLVNN KKATAVSTSG

Aamph_AQA26373.1 350 1.48e-12 ATDVAIITLA DGSKQVQLVNS QKTTASTSSG

Aamph_102386_c0_g1_i3.p2 116 1.48e-12 ATDVAIITLA DGSKQVQLVNS QKTTASTSSG

--------------------------------------------------------------------------------

**File S7.** Alignment of CP20k homolog sequences within the 10 MEME motif regions.

--------------------------------------------------------------------------------

Motif LPCNPKHPCYHCHCK MEME-1 sites sorted by position p-value

--------------------------------------------------------------------------------

Sequence name Start P-value Site

------------- ----- --------- ---------------

Cmala_13935_c0_g1_i1.p2 95 1.39e-13 CDCDCTHEDE LPCNRRHPCFHCHGK GKKYCHCGCE

Chunt_34424_c0_g1_i1.p2 95 1.39e-13 CDCDCTHEDE LPCNRRHPCFHCHGK GKKYCHCGCE

Aamph_Unigene33081_Ba_mi 83 1.70e-12 SHSDCDCNHV KPCNPKHPCWHKYTV KKGHKQHKRI

Aamph_Unigene4945_Ba_mix 110 2.40e-12 GHKQRCNCDH LRCNRKHPCWHRHCD CYCKHQH*

Aamph_Unigene33081_Ba_mi 115 2.40e-12 KRIYGCNCNH LRCNRKHPCWHRHCD CYCKHQH*

Aamph_CL917.Contig1_Ba_m 108 8.69e-12 LTTYDCDCDS IECSIRHPCWHRECG CNCTHTA*

Aamph_101384_c1_g2_i1.p1 58 1.01e-11 HYDCECNHLH DQCKPSHPCYHKHGS DYDCNCNHLK

Aamph_Unigene4945_Ba_mix 83 2.42e-11 SHFDCDCNHV KPCNPKHPCWNKYTV KKGHKQRCNC

Aamph_101384_c1_g2_i1.p1 82 6.22e-11 SDYDCNCNHL KPCNSKHPCWHGHKV KGSKKIVYDC

Mrosa_CP20k_BAB18762.1 147 1.32e-10 HDVCGCECSK LPCNDEHPCYRKEGG VVSCDCKTIT

Aamph_CL917.Contig1_Ba_m 80 2.12e-10 REVECNCNHL TPCDSSHPCWHPTVV NETLTTYDCD

Aamph_101384_c1_g2_i1.p1 36 2.68e-10 GHGHDHGHKS RPCGPGHRCYYCHYD CECNHLHDQC

Aamph_Unigene4945_Ba_mix 36 2.68e-10 DHGHDHGHKS RPCGPGHRCYYCHYD CECNHLHDQC

Mrosa_CP20k_BAB18762.1 80 3.00e-10 NNICKCSCTA IPCNEDHPCHHCHEE DDGDTHCHCS

Mrosa_CP20k_BAB18762.1 170 3.76e-10 GGVVSCDCKT ITCNEDHPCYHSYEE DGVTKSDCDC

Cmala_13935_c0_g1_i1.p2 67 4.20e-10 CKDMKCIGKS TRWYKRHPCYHCHGK KRRCDCDCTH

Chunt_34424_c0_g1_i1.p2 67 4.20e-10 CKDMKCIGKS TRWYKRHPCYHCHGK KRRCDCDCTH

Aamph_92857_c1_g1_i2.p1 74 4.20e-10 TEPSCDCNQL TPCDSSHPCWRTTVV NGTLTDNNCD

Mrosa_CP20k_BAB18762.1 118 6.48e-10 SHDHHDDDTH GECTKKAPCWRCEYN ADLKHDVCGC

Aamph_Unigene4945_Ba_mix 58 7.21e-10 HYDCECNHLH DQCKPSHPCYRKLPG SHFDCDCNHV

Aamph_Unigene33081_Ba_mi 58 7.21e-10 HYDCECNHLH DQCKPSHPCYRKLPG SHSDCDCNHV

Aamph_CL917.Contig1_Ba_m 50 8.89e-10 QDTCDCSSEG LFCTPAHRCFHIHVS NMSTTREVEC

Cmala_13935_c0_g1_i1.p2 32 1.21e-09 YHKKHNPKNH GVCNADAPCWHCKCN KKGVRSCNCS

Chunt_34424_c0_g1_i1.p2 32 1.21e-09 YHKKHNPKNH GVCNADAPCWHCKCN KKGVRSCNCS

Aamph_CL917.Contig1_Ba_m 28 1.64e-09 FVAADARGQK RNCNPGNPCFHCQDT CDCSSEGLFC

Cmite_20195_c0_g1_i1.p1 55 2.42e-09 KEDCNCDCNR MKCDDKHPCYHCYKD ANGKMHFDCD

Aamph_92857_c1_g1_i2.p1 102 2.42e-09 LTDNNCDCDS IACSISHPCRHRQCS CD*

Cmite_20195_c0_g1_i1.p1 83 2.67e-09 KMHFDCDCHH IKCDKRHACYHCHCK GKSCDDCHCD

Aamph_Unigene33081_Ba_mi 36 2.94e-09 DHGHDHGHRS RHCTPSHRCYYCHYD CECNHLHDQC

Cmite_32162_c0_g1_i1.p1 82 6.76e-09 KNECDCDCGH IECDKHHPCYHCHSC FDCHCDCTHS

Mrosa_CP20k_BAB18762.1 24 1.15e-08 AAVVSAHEED GVCNSNAPCYHCDAN GENCSCNCEL

Aamph_92857_c1_g1_i2.p1 47 3.72e-08 CTDSCDCAEL RFCKPAFPCFRLPSG NETEPSCDCN

Aamph_92857_c1_g1_i2.p1 26 1.73e-07 VFYAAGQGQG PPCNPGNPNFNCTDS CDCAELRFCK

Cmite_32162_c0_g1_i1.p1 54 2.00e-07 KDCNCDCDRI TCAKGIHPCYHCHKN GKGKNECDCD

Cmite_32162_c0_g1_i1.p1 26 2.68e-07 ISATAEAHEV PVCNALAKCWNCITK TGKKDCNCDC

--------------------------------------------------------------------------------

--------------------------------------------------------------------------------

Motif MKLSVLVVALA MEME-2 sites sorted by position p-value

--------------------------------------------------------------------------------

Sequence name Start P-value Site

------------- ----- --------- -----------

Chunt_34424_c0_g1_i1.p2 1 5.94e-13 . MKLSVLVVMLS LVVVALAHKD

Cmala_13935_c0_g1_i1.p2 1 1.75e-12 . MKLSVLVVTLS LVVVALAHKD

Aamph_101384_c1_g2_i1.p1 1 9.49e-12 . MKSSVLALCLA TVLAVLYVSV

Aamph_Unigene4945_Ba_mix 1 9.49e-12 . MKSSVLALCLA TVLAVLYVSV

Aamph_Unigene33081_Ba_mi 1 9.49e-12 . MKSSVLALCLA TVLAVLYVSV

Aamph_92857_c1_g1_i2.p1 1 4.95e-11 . MQSTLFVLFLA AASTVFYAAG

Aamph_101384_c1_g2_i1.p1 12 8.41e-10 KSSVLALCLA TVLAVLYVSVD AHEGHGHDHG

Aamph_Unigene4945_Ba_mix 12 8.41e-10 KSSVLALCLA TVLAVLYVSVD AHEDHGHDHG

Aamph_Unigene33081_Ba_mi 12 8.41e-10 KSSVLALCLA TVLAVLYVSVD AHEDHGHDHG

Mrosa_CP20k_BAB18762.1 1 2.81e-09 . MKWFLFLLTTA VLAAVVSAHE

Cmite_32162_c0_g1_i1.p1 7 3.73e-09 MKTVRV LFLLVVVVAIS ATAEAHEVPV

Aamph_CL917.Contig1_Ba_m 1 5.93e-09 . MKRTQLALCLT AVIVAVFVAA

Cmite_20195_c0_g1_i1.p1 9 6.49e-09 MMKTTRVL LLFFVVVAAIS ATAEAHEDHE

Aamph_CL917.Contig1_Ba_m 13 1.32e-08 RTQLALCLTA VIVAVFVAADA RGQKRNCNPG

Aamph_92857_c1_g1_i2.p1 12 1.85e-08 QSTLFVLFLA AASTVFYAAGQ GQGPPCNPGN

--------------------------------------------------------------------------------

--------------------------------------------------------------------------------

Motif DCDCDCNH MEME-3 sites sorted by position p-value

--------------------------------------------------------------------------------

Sequence name Start P-value Site

------------- ----- --------- --------

Aamph_Unigene4945_Ba_mix 74 4.32e-08 HPCYRKLPGS HFDCDCNH VKPCNPKHPC

Aamph_101384_c1_g2_i1.p1 73 1.07e-07 SHPCYHKHGS DYDCNCNH LKPCNSKHPC

Aamph_101384_c1_g2_i1.p1 103 1.67e-07 GHKVKGSKKI VYDCDCDH

Cmala_13935_c0_g1_i1.p2 84 2.16e-07 PCYHCHGKKR RCDCDCTH EDELPCNRRH

Chunt_34424_c0_g1_i1.p2 84 2.16e-07 PCYHCHGKKR RCDCDCTH EDELPCNRRH

Aamph_Unigene33081_Ba_mi 74 4.23e-07 HPCYRKLPGS HSDCDCNH VKPCNPKHPC

Cmite_32162_c0_g1_i1.p1 74 6.59e-07 HCHKNGKGKN ECDCDCGH IECDKHHPCY

Aamph_CL917.Contig1_Ba_m 100 1.51e-06 HPTVVNETLT TYDCDCDS IECSIRHPCW

Mrosa_CP20k_BAB18762.1 189 1.98e-06 YHSYEEDGVT KSDCDCEH SPGPSE

Cmite_20195_c0_g1_i1.p1 75 2.18e-06 HCYKDANGKM HFDCDCHH IKCDKRHACY

Aamph_Unigene33081_Ba_mi 107 2.18e-06 VKKGHKQHKR IYGCNCNH LRCNRKHPCW

Cmite_20195_c0_g1_i1.p1 47 2.37e-06 WNCITKDGKE DCNCDCNR MKCDDKHPCY

Cmite_32162_c0_g1_i1.p1 98 2.84e-06 HPCYHCHSCF DCHCDCTH SPQ*

Cmite_20195_c0_g1_i1.p1 103 2.84e-06 HCHCKGKSCD DCHCDCTH SPN*

Cmite_32162_c0_g1_i1.p1 45 4.53e-06 WNCITKTGKK DCNCDCDR ITCAKGIHPC

Aamph_92857_c1_g1_i2.p1 65 4.53e-06 CFRLPSGNET EPSCDCNQ LTPCDSSHPC

Aamph_CL917.Contig1_Ba_m 71 5.72e-06 IHVSNMSTTR EVECNCNH LTPCDSSHPC

Aamph_92857_c1_g1_i2.p1 94 1.24e-05 RTTVVNGTLT DNNCDCDS IACSISHPCR

Aamph_Unigene4945_Ba_mix 102 1.33e-05 WNKYTVKKGH KQRCNCDH LRCNRKHPCW

--------------------------------------------------------------------------------

--------------------------------------------------------------------------------

Motif GVRSCNCSCKDMKCI MEME-4 sites sorted by position p-value

--------------------------------------------------------------------------------

Sequence name Start P-value Site

------------- ----- --------- ---------------

Cmala_13935_c0_g1_i1.p2 49 9.00e-19 PCWHCKCNKK GVRSCNCSCKDMKCI GKSTRWYKRH

Chunt_34424_c0_g1_i1.p2 49 9.00e-19 PCWHCKCNKK GVRSCNCSCKDMKCI GKSTRWYKRH

--------------------------------------------------------------------------------

--------------------------------------------------------------------------------

Motif AVAEAHED MEME-5 sites sorted by position p-value

--------------------------------------------------------------------------------

Sequence name Start P-value Site

------------- ----- --------- --------

Cmite_20195_c0_g1_i1.p1 20 2.06e-10 LFFVVVAAIS ATAEAHED HECNNKTKCW

Cmala_13935_c0_g1_i1.p2 14 3.43e-09 SVLVVTLSLV VVALAHKD YHKKHNPKNH

Chunt_34424_c0_g1_i1.p2 14 3.43e-09 SVLVVMLSLV VVALAHKD YHKKHNPKNH

Cmite_32162_c0_g1_i1.p1 18 9.87e-09 FLLVVVVAIS ATAEAHEV PVCNALAKCW

Mrosa_CP20k_BAB18762.1 15 6.35e-08 LFLLTTAVLA AVVSAHEE DGVCNSNAPC

--------------------------------------------------------------------------------

--------------------------------------------------------------------------------

Motif NMKTTR MEME-6 sites sorted by position p-value

--------------------------------------------------------------------------------

Sequence name Start P-value Site

------------- ----- --------- ------

Cmite_20195_c0_g1_i1.p1 1 1.04e-09 . MMKTTR VLLLFFVVVA

Aamph_CL917.Contig1_Ba_m 65 8.97e-09 AHRCFHIHVS NMSTTR EVECNCNHLT

--------------------------------------------------------------------------------

--------------------------------------------------------------------------------

Motif AHEDHGHDHGH MEME-7 sites sorted by position p-value

--------------------------------------------------------------------------------

Sequence name Start P-value Site

------------- ----- --------- -----------

Aamph_Unigene4945_Ba_mix 23 3.99e-13 VLAVLYVSVD AHEDHGHDHGH KSRPCGPGHR

Aamph_Unigene33081_Ba_mi 23 3.99e-13 VLAVLYVSVD AHEDHGHDHGH RSRHCTPSHR

Aamph_101384_c1_g2_i1.p1 23 8.83e-13 VLAVLYVSVD AHEGHGHDHGH KSRPCGPGHR

--------------------------------------------------------------------------------

--------------------------------------------------------------------------------

Motif GKKYCHCGCEHSHTG MEME-8 sites sorted by position p-value

--------------------------------------------------------------------------------

Sequence name Start P-value Site

------------- ----- --------- ---------------

Cmala_13935_c0_g1_i1.p2 110 1.08e-17 RHPCFHCHGK GKKYCHCGCEHSHTG *

Chunt_34424_c0_g1_i1.p2 110 1.08e-17 RHPCFHCHGK GKKYCHCGCEHSHTG *

--------------------------------------------------------------------------------

--------------------------------------------------------------------------------

Motif YCKHQH MEME-9 sites sorted by position p-value

--------------------------------------------------------------------------------

Sequence name Start P-value Site

------------- ----- --------- ------

Aamph_Unigene4945_Ba_mix 126 6.32e-08 HPCWHRHCDC YCKHQH *

Aamph_Unigene33081_Ba_mi 131 6.32e-08 HPCWHRHCDC YCKHQH *

--------------------------------------------------------------------------------

--------------------------------------------------------------------------------

Motif ECNHLH MEME-10 sites sorted by position p-value

--------------------------------------------------------------------------------

Sequence name Start P-value Site

------------- ----- --------- ------

Aamph_101384_c1_g2_i1.p1 52 1.77e-07 HRCYYCHYDC ECNHLH DQCKPSHPCY

Aamph_Unigene4945_Ba_mix 52 1.77e-07 HRCYYCHYDC ECNHLH DQCKPSHPCY

Aamph_Unigene33081_Ba_mi 52 1.77e-07 HRCYYCHYDC ECNHLH DQCKPSHPCY

--------------------------------------------------------------------------------

**File S8.** Alignment of CP43k homolog sequences within the 10 MEME motif regions.

--------------------------------------------------------------------------------

Motif DAATEGASVGZATTZADANGRAATICKGTKTDANGGSSSATRGDGKAAG MEME-1 sites sorted by position p-value

--------------------------------------------------------------------------------

Sequence name Start P-value Site

------------- ----- --------- -------------------------------------------------

Cmala_17572_c0_g1_i1.p1 109 8.70e-46 PGFSKGLSKG DAATEGASAGQATTEADANGRAATICKGGKTDANSGSSSATRGDGKAKG VSRSVGQSGS

Chunt_74589_c0_g1_i1.p1 35 8.70e-46 PGFSKGLSKG DAATEGASAGQATTEADANGRAATICKGGKTDANSGSSSATRGDGKAKG VSRSVGQSGS

Aamph_AQA26377.1 128 3.17e-37 AGLNAASSQG DAITETRSTGEATTEADANGKGETLGEGSRTDANGGSSSATISDGSSAG AAQTVGTAGQ

Ctest_101681_c1_g1_i2.p1 144 1.54e-33 KLGSQGESQA ATRAQGASVGQATTKSDATGNAGTVCESTKTDVDTGSSSATRGDGKADA DSKTKSVAKT

Ctest_97391_c0_g1_i7.p1 231 2.17e-33 DGRQHSSSQG DAHTEVRSTGQSSTEADANARGATLLKNAKTDATGGSSSATVNEGTAAG ASETLGSAGR

Tform_TR52047|c0_g1_i1.p 117 2.02e-32 PVGSKGTSQA GTRTSGASVGQATTQSDATGRATTVCKSTKTDADTGTSTATHGAGKAEA DSKTKSTAKT

Ctest_97456_c0_g1_i2.p1 139 4.81e-32 ADAEQASSRG LAAAKGISVGQARTQSDANADAKTIGKGTKTDGSSGISTATEGDGSSSG ISEAIGASSR

Aamph_102661_c1_g6_i1.p2 126 1.13e-31 ANSEQASSDT RAAARGVSVGEARTQADANGAAATRGRGTKTDGNSGISTATESDGASSG LSQALGTASR

Tform_TR64719|c0_g1_i1.p 132 2.93e-31 HGHEHASSQG DAHTETRSTGQASTEADANSSGKTTGSGAKTDASGGSSSATTGEGAAAG ASQTLGTAGK

Majex_22249_c0_g1_i3.p2 147 6.73e-31 PAGLAGSSQG DAITETRSTGEVSTEADAVGGGATLSGGVRSDAHGDSSSATYGDGTAAG ITQTVGTGGQ

Majex_11586_c0_g1_i1.p1 109 5.67e-30 PYGGKGTSVS GQQVSGASAGEATTESDATGGAATVCDSTSTEVEGGASSATRSDGTAAS QTKNTATSET

Aamph_AQA26370.1 121 6.26e-30 PGFSKGASVT GQQTSGASVGEATSTSDATGEAGTICDDTKSDVQGGASTSTISDGKAAS EALNTAASET

Lanti_84167_c0_g1_i1.p1 155 1.70e-28 GFSEGSSSQT DALTNVFSSGQAQGAIDANAAGATTGAATRTDADSGSSSASRGDGEAQG ASQSIGTGGF

Cmite_21715_c0_g1_i1.p2 150 1.73e-27 GGVEDATSQA KTLTQSVSAGQATSQTDAVADAATVGLGTKTKAEGGTSSASRGNGQAGG GSKVVSSAGS

Aamph_73624_c0_g1_i1.p1 119 2.13e-21 PDFKKGVSVT GQEGKSGSIGEATTSTFAGGFANTRCNETRTSAQGQTKTGTSDFGQAGS ESLNKASSKT

--------------------------------------------------------------------------------

--------------------------------------------------------------------------------

Motif VGTKSKAVSSGSIKTTGNAKAVVSGGSKAAEQLPDQKSGANARVKLYSQREGLGEA MEME-2 sites sorted by position p-value

--------------------------------------------------------------------------------

Sequence name Start P-value Site

------------- ----- --------- --------------------------------------------------------

Tform_TR64719|c0_g1_i1.p 53 1.96e-42 YPPGLPPKLS LGTKTKADVAGKVISTGNAQVVVSGGAKAAERLPDQKSGANARVTLRTQENGLGAG SAGSVGRSDV

Aamph_AQA26377.1 51 3.93e-42 PEPVLVEKHS LGTRTKADVSGTIISTGNAKAVVSGGSRATQLLPDQRGGAAARVKLYTQRNGLGSG SAASVGRSDV

Majex_22249_c0_g1_i3.p2 70 3.03e-41 SDIVRVEKRG VGTKTRADVRGSIISIGNAQAVISGGARAAEQMADQNAGADVRLKLDAQRNGLGAG KAAAVGRSDM

Ctest_97391_c0_g1_i7.p1 152 4.17e-40 NAVLPEGKRN LGTKSQTDSAGKIISTGNAKAFASGGARASDMLPDQKSGADVRLTLKTQREGASEG VAGSVGRSDA

Cmala_17572_c0_g1_i1.p1 30 1.69e-39 VGPAVVLNPP VATKSKAKAAGDLKTVGNAGASVSGGSITFETAADQKTDANARVSIKSQEEGIGQA SAASQGTSGV

Ctest_97456_c0_g1_i2.p1 60 9.31e-38 TTTTTTEKPG YGTRSKTEVGGKLKTVGNVIASVGGGSNAIETLADQQSKANSRVVINAQQAAIGEA NAAAKAKSDI

Cmite_21715_c0_g1_i1.p2 71 5.05e-36 PVLPVPAPSA EGTKSKAVTGGSLQTTGNARADVSGGSTSAEQTGDQKTKAHVRTELTSQEHGLAAT KAKSVGQSAK

Aamph_102661_c1_g6_i1.p2 47 4.95e-35 TTTTPAQPEG LATETKALAGGKLKTVGNALAQVAGGSNTVERQESQESAANARVQIDAQRAAIGQA KAASKGRSDV

Majex_11586_c0_g1_i1.p1 30 1.25e-32 PSKSVLPPKV VGTTSDTISTGSIVTGGNSKGTAKGGGVAAQQSDSQRSNTASKVNLYSQRYGKAKA SSASSSGTGT

Tform_TR52047|c0_g1_i1.p 37 2.69e-31 GKKPPLPPPP KGTTTDTVSSGGVRTSGHAKAVVSGGSKAVENAQNQKTGSKAAVKLTSQATGQGEA SAGSTSGTKV

Aamph_5689_c0_g1_i1.p1 17 3.24e-31 AAPAPGKAVT VGTDSSTVATGSVTTGGNANGSAKGGSVTTVQADNQKSNTASKVNIYGQRYSKGEA TAASSSGTGA

Ctest_101681_c1_g1_i2.p1 64 2.69e-30 PPGVSLGQSP GGTGSDTKSSGGVVTTGHARSVVQGGGKAYEKGDDQQTASQSSVKLTSQKEGEAEA AAASTSGSKI

Lanti_84167_c0_g1_i1.p1 76 1.99e-28 PQQEPIDANN IASESTAIGSASISTNGNARSAVSGGTQATQSIPSQRTDAILKLSLESQEEGDVTA AGRSDSRTDI

Aamph_AQA26370.1 42 1.17e-27 PVPPPLPPKR AATDADAVTVGTLKTAGTAIGKSSGGAVSLEQTADQGSKAGVKVDLYSQRAGATEG SAASTSATKV

--------------------------------------------------------------------------------

--------------------------------------------------------------------------------

Motif ASESTANNQLKATRTGAAKAEVFTEGGSTLGLD MEME-3 sites sorted by position p-value

--------------------------------------------------------------------------------

Sequence name Start P-value Site

------------- ----- --------- ---------------------------------

Majex_22249_c0_g1_i3.p2 319 9.02e-31 KSTADQNGTT SSDSVARNQLKASRDGVAKAEVFTTGMSALGLD EFSKDLVVDT

Ctest_97391_c0_g1_i7.p1 403 3.59e-29 KGTAGKDSAT SSDSVARNQLKASRTGRAKADVITEGLSALGLD SFIDDLVVDT

Aamph_140108_Unigene1170 43 8.72e-28 KATATESGTS AAESSANNRLAATRTGRAETSIFTEGGSTLGLD QFSGDVVGAV

Aamph_106445_c0_g2_i1.p2 93 3.64e-27 KATATESGTS ATESSANNRLAATRTGRAETSIFTEGGSTLGLD Q

Aamph_AQA26374.1 81 6.88e-26 KGTAGTNSTT SSDSTASNRLKATRGGVAESDVFTTGLSELGMD SFTKGLAVKT

Ctest_97456_c0_g1_i2.p1 314 4.76e-24 KSTASEKNST ASDSIAQNKLKASRNGRGDATLFSESQSTLGLD QFTGDVVGAV

Tform_TR64719|c0_g1_i1.p 304 6.53e-24 KGTAGKKGTT SSDSVARSQLKASKDGAAKASVFSTGLSALGLD SFTKDLVVDT

Cmala_17572_c0_g1_i1.p1 280 1.22e-23 AQARAGDGVT NSKSQTGQKLKSARDGAAAAKTETEAKSTLGLD AFGGTLFGTT

Chunt_221884_c0_g1_i1.p4 90 1.22e-23 AQARAGDGVT NSKSQTGQKLKSARDGAAAAKTETEAKSTLGLD AFGGTLFGTT

Ctest_101681_c1_g1_i2.p1 319 8.05e-21 EATANQGSNV ASESEANESVKATRTGAAKAAVGTGGSGVLGKD GQDIVGDSRA

PECT_74810_c0_g1_i1.p3 84 8.24e-20 KGRARAGDSL TSKSTANTQLRSTNTGAASVEVKSSSGSTVASD DLESFVTATQ

Cmite_21715_c0_g1_i1.p2 325 8.24e-20 KGRARAGDSL TSKSTANTQLRSTNTGAASVEVKSSSGSTVASD DLESFVTATQ

Tform_TR52047|c0_g1_i1.p 292 2.63e-19 GATAGQGDNV AAESDANEGVQTTRTGVAAAKVGSKASAVLGKD GKDIVGDSQA

Lanti_84167_c0_g1_i1.p1 331 3.89e-18 QATAERDGLQ TSKSTTSNQLNSAGDGAAVAETGSIGASALSLD DIGAPNTRTQ

Majex_11586_c0_g1_i1.p1 284 7.17e-18 KATANKGSVV AAESKTNEEVGTTRTGSASSDVKAGSGSVVGKD GSDIVGDSQA

Aamph_AQA26370.1 296 4.44e-16 GGTANQGSNV AAESDSNQKIRSTRTGSSAVDAKSGSAAALGAI KDKLVGKSDA

--------------------------------------------------------------------------------

--------------------------------------------------------------------------------

Motif PVWPCVLPSKPPKPY MEME-4 sites sorted by position p-value

--------------------------------------------------------------------------------

Sequence name Start P-value Site

------------- ----- --------- ---------------

Tform_TR52047|c0_g1_i1.p 425 1.18e-17 WYKKYGTIKH PIWPCVLPIKPIKPF KPKPKKDPFK

Aamph_AQA26370.1 402 2.01e-15 SASGSVTLKR PVWPCRLPSKAPKDW LHGWVPGTKL

Aamph_AQA26370.1 426 3.14e-15 WLHGWVPGTK LVWHCVFPHKIPAKY SQLYKPKW

Tform_TR52047|c0_g1_i1.p 401 5.45e-15 GRVTPAKKPK HVWPCKFPTKAPKGW YKKYGTIKHP

Aamph_73624_c0_g1_i1.p1 434 5.45e-15 WLDDWPITPN PVWPCELPLSPIDPF KPKASKSKDM

Aamph_73624_c0_g1_i1.p1 410 3.00e-14 IASVPTRLKK PKWPCEKPSAPPHGW LDDWPITPNP

Ctest_101681_c1_g1_i2.p1 444 4.09e-14 PKAGWQFKDG DVWPCVFPVNFKAPF KPKVAFKG*

Majex_22249_c0_g1_i3.p2 464 1.25e-13 YVPPQPSPEP YVPPYVPPQPSPAPY VLPFVRSRPN

Majex_22249_c0_g1_i3.p2 492 3.02e-13 FVRSRPNPAL HVPPYVRPQPNPAPY VPPYVRPQPS

Majex_11586_c0_g1_i1.p1 392 1.05e-12 QGRTTVADKK PKWPCQLPSAPSKDW LKLLPNVKGT

Tform_TR64719|c0_g1_i1.p 452 1.52e-12 YVPPHPSPKP YVPPYVPPPPSPSPY VPPYAPPGPR

Majex_11586_c0_g1_i1.p1 416 5.48e-12 WLKLLPNVKG TDWPCVVPGKAEEPY KAVALGSLF*

Aamph_AQA26374.1 261 3.16e-11 PPYSHKQSTG PYIPPYAHKQSPEPY TPPYVPKQS

Aamph_AQA26374.1 235 3.44e-11 PAPEPYVPPV PAPEPYVPRPRPEPY TPPYSHKQST

Aamph_AQA26370.1 25 5.26e-11 LSAPAPGVTP PVSPPLPPVPPPLPP KRAATDADAV

Cmite_21715_c0_g1_i1.p2 44 6.22e-11 PGPPVVPPPP PPPPVVVPVPPPPPP VVPVLPVPAP

Ctest_101681_c1_g1_i2.p1 424 1.21e-10 AEAQGRQKPV VQWPCSLPGKPKAGW QFKDGDVWPC

Tform_TR64719|c0_g1_i1.p 38 2.96e-10 RPSAVVVVAT PVRPPYPPGLPPKLS LGTKTKADVA

Ctest_101681_c1_g1_i2.p1 31 9.64e-10 GPVGPVHPVY PVSPGQPPVYIPPPP PTTRQPPPPP

Tform_TR64719|c0_g1_i1.p 434 1.53e-09 IKKPGPPPYA PPHPAPKPYVPPHPS PKPYVPPYVP

Aamph_73624_c0_g1_i1.p1 26 2.24e-09 CAPAPGESYP TVPPTVPPTKPPKKD IAKTNAYTGG

Aamph_140108_Unigene1170 192 7.87e-09 GKGKGPRPTQ PPPPPTLPSPPRGKH *

Ctest_97391_c0_g1_i7.p1 612 5.27e-08 APRASPKKSY GYYPSKKDRRVPQPW ELH*

Lanti_84167_c0_g1_i1.p1 34 1.35e-07 RQLPTVPPRV PVAPVKIQIQVPTPV QPAAPQIQQP

--------------------------------------------------------------------------------

--------------------------------------------------------------------------------

Motif GTTSISGSQTKVDTIGDGKAAARZRGGA MEME-5 sites sorted by position p-value

--------------------------------------------------------------------------------

Sequence name Start P-value Site

------------- ----- --------- ----------------------------

Majex_22249_c0_g1_i3.p2 243 1.47e-25 GRGRGVAIGS DTTSASGSQTKVDVMGDGKAQAFERGTA RGELAAGGPV

Tform_TR64719|c0_g1_i1.p 228 2.31e-24 GRGRGAALGG GTTSVSGSHTRVDVIGDGKAQAVQRGGA GGELAGDALV

Ctest_97391_c0_g1_i7.p1 327 9.77e-23 GRGRGAAFGG GTTSLAGSQSETDTIGDGKAQSVQRGGA GGELDGNGVL

Aamph_AQA26374.1 9 1.60e-22 GRGAAFGS DTTSLSGSQTRSDVIGDGQARSVETGGA AGQKGDGISQ

Ctest_97456_c0_g1_i2.p1 238 7.49e-22 ARGRGASVNS GTTSVSGSQTSVDVIGDGKAAAIEGGGA TGESGSSSIL

Aamph_106445_c0_g2_i1.p2 17 1.33e-21 GRARSAAVQA NTTALSGSQTKVDVIGDGRATAAEAGGA LGQSGGSASR

Cmala_17572_c0_g1_i1.p1 205 5.42e-20 RGRAAAIGAR GSTSISGSQTEGGVIGDGKVKSRQVAGA ATRTDRGPLG

Chunt_221884_c0_g1_i1.p4 15 5.42e-20 RGRAAAIGAR GSTSISGSQTEGGVIGDGKVKSRQVAGA ATRTDRGPLG

PECT_74810_c0_g1_i1.p3 8 2.30e-18 ASAGVTG GTSSISRAQTGSDTLGDGVAAARKTVYS KGAVGGDGAG

Cmite_21715_c0_g1_i1.p2 249 2.30e-18 GRGASAGVTG GTSSISRAQTGSDTLGDGVAAARKTVYS KGAVGGDGAG

Tform_TR52047|c0_g1_i1.p 216 9.86e-17 SRARAGGVGD GTTAQSGSQVKTETTGDGAATGSQKGEA GGTTAVGVGA

Aamph_AQA26370.1 220 9.86e-17 GKASSRGIGD GTTSRADSQTKTSTTGDGRSEADQRSTG TGTTGRKRGA

Majex_11586_c0_g1_i1.p1 208 1.13e-15 GRAGATGTGD GVTSNTKSQTKISTTGDGRSAADQKAIG SASQGSGVGS

Lanti_84167_c0_g1_i1.p1 255 3.14e-15 RGRAVSTGGQ DTTAAAGAQTGVNTIGDGAGTSQETSLS SGISVGDGVG

Ctest_101681_c1_g1_i2.p1 243 1.69e-14 GRARGASIGK SGTSKSGSQTQTETTGDGSAVGGQQGTA TGTTGKGPNL

--------------------------------------------------------------------------------

--------------------------------------------------------------------------------

Motif RGAETKAGSKGEVQTVGPGASSAGESSSKATAGEGGT MEME-6 sites sorted by position p-value

--------------------------------------------------------------------------------

Sequence name Start P-value Site

------------- ----- --------- -------------------------------------

Aamph_106445_c0_g2_i1.p2 55 3.22e-25 LGQSGGSASR RASRTEAANKGEVETRGDGAGAVGESSSKATATESGT SATESSANNR

Aamph_140108_Unigene1170 5 3.22e-25 SASR RASRTEAANKGEVETRGDGAGAVGESSSKATATESGT SAAESSANNR

Cmala_17572_c0_g1_i1.p1 243 7.64e-24 ATRTDRGPLG KSAQSRAASEGEVQTAGPGASGGGESNAQARAGDGVT NSKSQTGQKL

Chunt_221884_c0_g1_i1.p4 53 7.64e-24 ATRTDRGPLG KSAQSRAASEGEVQTAGPGASGGGESNAQARAGDGVT NSKSQTGQKL

Majex_22249_c0_g1_i3.p2 281 5.79e-23 RGELAAGGPV RCGSTQVGSEGDVKTLGDGASGAGETTVKSTADQNGT TSSDSVARNQ

Ctest_97456_c0_g1_i2.p1 276 1.23e-22 TGESGSSSIL RSSRTEAGSKGSVRTQGPGAGATGESSSKSTASEKNS TASDSIAQNK

Tform_TR64719|c0_g1_i1.p 266 4.80e-22 GGELAGDALV RVGHTHAGSQGDVKTQGPGASGTGGSASKGTAGKKGT TSSDSVARSQ

Aamph_AQA26374.1 43 4.80e-22 TGGAAGQKGD GISQTQATSKGKVHTLGNGASGDGETSAKGTAGTNST TSSDSTASNR

Tform_TR52047|c0_g1_i1.p 254 2.78e-20 GGTTAVGVGA IGAETNADAKGESSSVGGGSSSAGETTAGATAGQGDN VAAESDANEG

Ctest_97391_c0_g1_i7.p1 365 6.27e-20 GGELDGNGVL RVGGSKARSQGDVKTLGSGASGSGESTSKGTAGKDSA TSSDSVARNQ

PECT_74810_c0_g1_i1.p3 46 1.16e-19 KGAVGGDGAG VGTATKAGAKGDVATNGPGAASAAEGNTKGRARAGDS LTSKSTANTQ

Cmite_21715_c0_g1_i1.p2 287 1.16e-19 KGAVGGDGAG VGTATKAGAKGDVATNGPGAASAAEGNTKGRARAGDS LTSKSTANTQ

Aamph_AQA26370.1 258 3.02e-19 TGTTGRKRGA LGAETSAQTTGSSATVGGGSDSKGESSAGGTANQGSN VAAESDSNQK

Ctest_101681_c1_g1_i2.p1 281 1.16e-18 TGTTGKGPNL AGADTSTTAVGESNTVGTGSSSGGETSSEATANQGSN VASESEANES

Lanti_84167_c0_g1_i1.p1 293 5.34e-17 SGISVGDGVG QAVSSDSSTIGAVQTFGPGAEALGEGTGQATAERDGL QTSKSTTSNQ

Majex_11586_c0_g1_i1.p1 246 1.39e-16 SASQGSGVGS AGVETDSRSTGSSQSVGGGSSSEGGSASKATANKGSV VAAESKTNEE

Aamph_73624_c0_g1_i1.p1 256 1.12e-13 TATLARVAET LGAESSTGTIGAARSAGDKSSITGNAAATGEANEGGD VRANTKSNQE

Aamph_AQA26370.1 342 3.85e-12 LVGKSDAASG GSAESVGSAKTDFNTGGSAGHSAGEGSGFAETSVGGQ TRQTGAVEGS

--------------------------------------------------------------------------------

--------------------------------------------------------------------------------

Motif VADSTFRGKTAGTGAVKGSLZSRGQGRV MEME-7 sites sorted by position p-value

--------------------------------------------------------------------------------

Sequence name Start P-value Site

------------- ----- --------- ----------------------------

Tform_TR64719|c0_g1_i1.p 380 7.50e-28 AGSSLVGGDA VADSTFQGKTSGSGHIKGILESRGQGRV ADGKPVVQPQ

Ctest_97456_c0_g1_i2.p1 390 5.15e-22 ATNALGGGAA AADSTFNAKSAGDGSVAGTIESRGQNRI KSQSSPVQLS

Aamph_140108_Unigene1170 119 2.54e-21 AQNALGGGAA LGDSTFRAQTSGTGAVSGTLETRAQNRL KADAQPAQLS

Majex_11586_c0_g1_i1.p1 358 9.46e-21 GGHSIGSGSG VADSTIGGESAGTGFVKGSQESRAQGRT TVADKKPKWP

Tform_TR52047|c0_g1_i1.p 366 1.31e-20 GAHNLSGGSG AADTTIKAKTAGTGAAEGSHQARAQGRV TPAKKPKHVW

Majex_22249_c0_g1_i3.p2 395 3.05e-20 SGSTLVGAGT VSDTTFTGKTSQAGHIKAQLTARGQGRV TDGEPVVVAQ

Ctest_97391_c0_g1_i7.p1 479 3.86e-18 AGSSLTGGAA VADTTVQGKTSKAGEIRSVLETNGQARV GDGKPVVQPS

Lanti_84167_c0_g1_i1.p1 406 8.11e-18 GTVLTPLGGS TSDATFRGVTAGDGSIEGSILTEGGNRL DLALPGSLPG

Cmala_17572_c0_g1_i1.p1 356 6.39e-17 GVSKAGGGGT KSDSAYKGKTAGDGAVSGSQQSQGEGVV KLGDNKASSI

Ctest_101681_c1_g1_i2.p1 393 4.56e-15 AGHSLGLGSG VAQSRSEATTAGTGTASGTHQAEAQGRQ KPVVQWPCSL

Cmite_21715_c0_g1_i1.p2 400 4.56e-15 SGVRTPSGGG VADSAYRAETGGTAAVKGGQEAGGVTVV RRGPSVVDDL

Aamph_AQA26374.1 158 7.34e-15 GSTLLGAGAA ITDTTFRGESVTAGSIKGALQSSGVAGV ADGQPVVQAA

--------------------------------------------------------------------------------

--------------------------------------------------------------------------------

Motif FTGDLVVDTKSKTSGSQTTLGSANSAANGASTAGSSLGGG MEME-8 sites sorted by position p-value

--------------------------------------------------------------------------------

Sequence name Start P-value Site

------------- ----- --------- ----------------------------------------

Tform_TR64719|c0_g1_i1.p 338 1.86e-35 TGLSALGLDS FTKDLVVDTKSKTTGTQTTLGSGSSAANGASTAGSSLVGG DAVADSTFQG

Aamph_140108_Unigene1170 77 1.16e-30 EGGSTLGLDQ FSGDVVGAVKSLTSGSQSSLGSANSKVNAASSAQNALGGG AALGDSTFRA

Majex_22249_c0_g1_i3.p2 353 4.26e-30 TGMSALGLDE FSKDLVVDTKSTTRGSQTTAGSAAAAANGGSTSGSTLVGA GTVSDTTFTG

Ctest_97391_c0_g1_i7.p1 437 1.88e-29 EGLSALGLDS FIDDLVVDTKSKSASNQQSVGSASSAVNGGSTAGSSLTGG AAVADTTVQG

Ctest_97456_c0_g1_i2.p1 348 1.44e-26 ESQSTLGLDQ FTGDVVGAVGSLTSASQSTSGSANSAQNGASSATNALGGG AAAADSTFNA

Aamph_AQA26374.1 115 2.61e-26 TGLSELGMDS FTKGLAVKTKSVTTGEQTTAGSARAKSNGASAVGSTLLGA GAAITDTTFR

Cmala_17572_c0_g1_i1.p1 314 2.01e-23 EAKSTLGLDA FGGTLFGTTKTKASGKQQSLGNANSDANAASTGVSKAGGG GTKSDSAYKG

--------------------------------------------------------------------------------

--------------------------------------------------------------------------------

Motif MLFWILLLAAL MEME-9 sites sorted by position p-value

--------------------------------------------------------------------------------

Sequence name Start P-value Site

------------- ----- --------- -----------

Cmala_17572_c0_g1_i1.p1 1 3.05e-15 . MNVWILLLAAL GAVNAAPLVG

Aamph_AQA26377.1 1 3.96e-15 . MHCWILAVACL GAAAAAPCIN

Tform_TR64719|c0_g1_i1.p 1 7.14e-15 . MSFWIVLVAAL SAAGAAPCHK

Ctest_97391_c0_g1_i7.p1 2 4.49e-13 M MTCWIALSAAL GALCAYTNGA

Aamph_102661_c1_g6_i1.p2 1 4.76e-12 . MKFVVLLAAAL SVASAVPTGF

Majex_22249_c0_g1_i3.p2 1 1.45e-11 . MHPWVVVLAAT LGVATAAPCV

Majex_11586_c0_g1_i1.p1 1 1.81e-11 . MLFAVVLVATL AGLHAAPAPS

Aamph_AQA26370.1 1 3.94e-10 . MLPAAILLLSL GAALSAPAPG

Aamph_73624_c0_g1_i1.p1 1 1.63e-09 . MLAVVLLLLVA GGALCAPAPG

--------------------------------------------------------------------------------

--------------------------------------------------------------------------------

Motif VPVGPVGPVNP MEME-10 sites sorted by position p-value

--------------------------------------------------------------------------------

Sequence name Start P-value Site

------------- ----- --------- -----------

Ctest_97391_c0_g1_i7.p1 113 4.24e-13 VGPVVPVNPG VPVGPVVPVNP GVPSVVTPGH

Ctest_97391_c0_g1_i7.p1 101 4.24e-13 VGPVGPVNPG VPVGPVVPVNP GVPVGPVVPV

Ctest_101681_c1_g1_i2.p1 18 5.00e-13 VGPVHPVGPV HPVGPVGPVHP VYPVSPGQPP

Ctest_97391_c0_g1_i7.p1 89 1.05e-12 GGPVGPGYPG VPVGPVGPVNP GVPVGPVVPV

Ctest_101681_c1_g1_i2.p1 6 3.28e-12 PVGPV HPVGPVHPVGP VHPVGPVGPV

Ctest_97391_c0_g1_i7.p1 53 1.26e-10 LGTYPVVTGT VPQVPVAPVYP GVPGGPVVPG

Ctest_97391_c0_g1_i7.p1 77 1.18e-09 GGPVVPGQPG VPGGPVGPGYP GVPVGPVGPV

Majex_22249_c0_g1_i3.p2 446 3.63e-09 NPDEDEPEPP VPVEHPVPYVP PQPSPEPYVP

Ctest_97391_c0_g1_i7.p1 136 5.67e-09 PSVVTPGHPG VPDVPVNAVLP EGKRNLGTKS

--------------------------------------------------------------------------------

**File S9.** Alignment of CP52k homolog sequences within the 10 MEME motif regions.

--------------------------------------------------------------------------------

Motif GLPALSRPRASLGGLVSYLKSAKISRTTF MEME-1 sites sorted by position p-value

--------------------------------------------------------------------------------

Sequence name Start P-value Site

------------- ----- --------- -----------------------------

BAL22342.1 312 6.42e-25 YDMITIFRGF GLPKLYQPRYTLGGLVSYLKVAKISQPTF IGQIKKYAKK

Ctest_99265_c0_g2_i11.p1 188 3.87e-23 YELLTVLKSA GLPGFHNPRFSLGGLISYMKITKISHTTF IVGIRAHISS

Aamph_106899_c1_g1_i1.p1 320 6.39e-22 YSLLPVLRGI GLPKLYRPKYTLGGIVAYLKIKKISQVTF IARIKKYRLK

Aamph_106899_c1_g1_i1.p1 33 6.39e-22 FTLKPVFSEI GLPSLYKPQYSLGGLYGYLQQADIPKTQF VSRVRKYGPY

Aamph_106899_c1_g1_i1.p1 192 1.46e-21 YEILATLKSL GLPPLVNPRASLGGIVAYLKVVGVSKTTF ISRIQVYRQA

Ctest_99265_c0_g2_i11.p1 316 4.77e-21 YKMAPLLQRI GLPGLKYPQYTLSGIVSYLKVVRISQTTF ISNIQTHADS

Cmite_21231_c0_g1_i2.p1 546 5.43e-21 SSLLPIWSRI GLPALSQPQATLGGLRSYLASTNIPRPTF VQYISSLKPH

Aamph_106899_c1_g1_i1.p1 448 7.00e-21 YNLIRVFRGI GLPKLYRPKYSVGGLVAYLKVVKIKQVTF ISQINKYSTS

Ctest_87731_c0_g3_i2.p1 129 3.08e-20 SSLQSLVFSI GLPAFSRPRASLGGLEQFLRSADIPYSQF LSRASQVGRR

Ctest_99265_c0_g2_i11.p1 444 7.06e-20 YKMAPILQRI GLPGLKYPQYTLSGIVSYLKIVKIGQTTF INSIQAHAAS

Cmala_24475_c0_g1_i1.p1 234 7.06e-20 TKLIYILHNM GLPRLYKPRTSLAGLVAYLDRVGIPQTRF VDRVRKLRGD

Cmala_24475_c0_g1_i1.p1 371 1.26e-19 HHLEKVLSSL GLPRLYRPRESLSGLEEYLQHAQINHDNF VDRIRKMRQR

BAL22342.1 184 3.86e-19 YGLLTVLQSV GLPALTNPRMSLGGVVAYLQLANIQQAVF ISRIRSQRKA

Ctest_99265_c0_g2_i11.p1 42 8.25e-19 GNLRHVLASM GLPEFAQPNDSLNGLMSYLQQVHVSRAEF LGRVQMYAPF

Cmite_14519_c0_g1_i2.p1 222 8.25e-19 SSLLPIWSGI GLPALSQPQATLGGLYSYLASANIRTVDF IRHCRTIRLP

Mlong_27848_c0_g1_i2.p1 90 9.18e-19 ISLQATLADI GLPSFHNPAISLSGLKSYLQRQGVTTEQF NRSLLKHRAT

Cmala_24475_c0_g1_i1.p1 506 1.02e-18 HGLARALRRL GLPRFSQPDASLGGLIVFLRGQRIPRHQF VARLATLSSN

Mlong_24074_c5_g7_i1.p1 162 1.14e-18 LGLLTELTNI GLPTLKQQENSLNGLLSYLDSARIDRQMF VDSISSNGDN

Mlong_27848_c0_g1_i2.p1 235 1.92e-18 SELLATMRSI GLPTLTNKQASLGGVDAYLSSAGITMETF ARRITLFGED

Ctest_97758_c3_g2_i5.p1 291 2.13e-18 YKLTPILKKM GLPALKDVETSLGGVVVYLKAAKIRMATF INFIQAFSIG

Tform_TR5993|c0_g1_i1.p1 39 3.95e-18 RSLQRVVQQI GLPAFSRPQASLGGLNVLLRSANIPHRRF YSRVSQVGGQ

Cmala_16752_c0_g1_i1.p1 261 8.81e-18 ETLPLVLSRL GLPKFYRPHTSLAGLLRVLKSASISQHRF VAHLRQLQGQ

Mlong_15653_c0_g1_i1.p1 72 1.31e-17 HELLSTLTEM GLPALEYPETSLGGIVAHLKLASVSLKRF IGAIKQYGND

Cmite_12453_c0_g1_i3.p1 264 1.75e-17 EAIAPVISRI GLPRLLQPAVTLGGLADYLHVQHVPVRAF VRHIKVYRAP

Mlong_26441_c1_g5_i3.p1 59 5.49e-17 HEYLSSIKSL GMDSLNYPSISLGGWAKYMDQYEYCRTQF EDKIAAKKTE

Mlong_30790_c11_g1_i1.p1 188 6.62e-17 YELISVLQEI GLHALTYPRPSLGGLVGRLNVAQISRETF NSAVRQYNST

Aamph_106899_c1_g1_i1.p1 584 1.81e-16 YQLKPLLTTV GLPKLTKPQYSLPGLVGYLQSNKYPLPSL VGRIQKYGPK

Mlong_6982_c0_g2_i1.p1 35 3.10e-16 EEYLQNLQSL KLPALVNPRATLGGWKKYMVKYGYSKAAF ETEIASAKKL

Mlong_24074_c5_g7_i1.p1 33 6.83e-16 ASLQPALTTA GLSSFSSTTVALGGLASFLQQKEISLTTF NSRVTQYETK

Mlong_32972_c3_g29_i1.p1 32 1.05e-15 HEYGKILKEA GLPDLIHRNKTLEGWVDYMNEHAVDATTA QANARGNSSN

BAL22342.1 436 1.05e-15 YNLKPVLLQA GLPKLSMPQYSLSGLMSYIHGNKYSDSSL IGLIRVYGPK

Ctest_97758_c3_g2_i5.p1 37 2.07e-15 RRLERVMADI RLPKLSRPYTSLNGLLEYLQQTHVPEFKF LSRIQLHGAD

Syats_lar_TR23764|c0_g1_ 218 2.66e-15 RLLSNILPSI GLPTPSLPAVTLPGLVRYLGSIGIPFDQF VSTARRLNIN

Mlong_27848_c0_g1_i2.p1 359 6.04e-15 YSLRSRLVSA GLPVMSAPHICLPGLISYLQSNSYSRDTL VSRIQTQSSA

Cmite_67826_c0_g1_i1.p1 129 1.15e-14 GSLLPTLSKI GLPVPVQPTVTIPGLVDYLVGQRVPVQRF ISRVRVIRIH

Mlong_62463_c0_g1_i1.p1 37 1.24e-14 KPLAQILDQF DMSKLKYQMVSLQGLERALTEEDISHQQF VDLFKRYKIG

Cmala_9305_c0_g1_i1.p1 167 5.88e-14 RTLRPVLARA GLSQLLHPATVIGGLADYLGAQRIPVSRF AHCVSLLRIP

Wmill_16510_c0_g1_i1.p1 36 1.94e-13 EQLRTALKSI EMPDFIRPERALAGLDFFMTDNGITYQSF VSRIKKWRYQ

Mlong_11150_c0_g1_i1.p1 46 3.47e-13 DSYWLKKSLY GFLPLHHYNVSLQGLESVLLEEEVFANDF HNRISAYKHA

Mlong_24434_c0_g1_i1.p1 49 3.73e-13 YLSSLSVLNE ETRTLLFPREMLGAWASYMREFNENKTEF DNRLAIATVI

Mlong_23544_c0_g1_i1.p1 98 4.01e-13 GSEYLPILTE IQVDLHDEEPALGGWKRYMDEHSINKDSF ETQLRNYQHF

Mlong_62806_c0_g1_i1.p1 72 7.60e-13 DTLLVKKSLY GFLPFHHYNASLEGLEDAIFEEDVFPNDF HNRISAYEHT

Mlong_20508_c0_g1_i1.p1 30 7.60e-13 SISSAVGQIT PLSSFSDSTTSLEGLESFVKQSTVSESQF IANIQGSSTQ

Ctest_97758_c3_g2_i5.p1 163 1.33e-12 HKLLAVLRRV GLLHFHNTANAVGGTVAYLELANVNYAAF LRRISTYRSI

BAL22342.1 38 1.75e-12 SSLKPVLSGI GLPAFYKPDYALSGLVGYLNTRPKIVTQA QFTARIQKYA

Mlong_14009_c0_g1_i1.p1 40 2.15e-12 YLSSLSGLND ETKTLLLSREMLGAWASYMEEFGHSKTTF DTRLSEDSVI

Mlong_1830_c0_g1_i1.p1 185 2.64e-12 SQLASTLSDL GLYPLKRQKASYNGYSDYLKSKKRTEASA VYVLKQYSNQ

Mlong_22409_c0_g1_i2.p1 46 4.22e-12 DHYWLKRGFA LFYPFSNEDATLNGFDDFLMKYNWSAQSF YQRLHEYQIN

Ctest_97758_c3_g2_i5.p1 419 8.19e-12 VGLEPHLKII VPSGLDYAKITLMGLDSYLKDTEVKRTPF INRIRARDTR

Mlong_25582_c0_g1_i1.p1 39 1.47e-11 DVYWLQLSIS GFQTFYHIANSLEGLRRFLVKEEVTPRQI YDNIRAYKGD

Mlong_1830_c0_g1_i1.p1 53 4.31e-11 SVDNLESALS SLGMSYHYRSSLSGLISYLKEKKLPQAKY IAGVRKYKER

Mlong_56954_c0_g1_i1.p1 52 1.56e-10 SHFASTLEEV GLYPLRRQKASFNGYSDYLKGKKSKRVSP INILKQYANQ

Mlong_30790_c11_g1_i1.p1 42 2.10e-10 SDISTILSAT PLPTLSFRTVSTTTLAKHLQANGASLHTF RSILQRYGKV

Ctest_94376_c0_g9_i3.p1 341 2.06e-09 RVFGAVQRAG QPAAAEAVTTSIGGLDQFLHCTRVARSQM SSSVSRAGGH

Mlong_20086_c0_g1_i1.p1 42 3.02e-09 FNFIDAVEEI GLPSLECGRISSAGFNNHLGSSSRQNQVA GEMRKHKEAI

--------------------------------------------------------------------------------

--------------------------------------------------------------------------------

Motif NKLIDPLSLVDIRLASYLYRPVSVQTFNQFFVRQILKYNGK MEME-2 sites sorted by position p-value

--------------------------------------------------------------------------------

Sequence name Start P-value Site

------------- ----- --------- -----------------------------------------

Tform_TR47370|c0_g1_i2.p 91 2.15e-36 LRTLNFPYSQ DKLIVPLSLTDIRLAAYQYQPLNVQRFNQFFIRNVLKFNGK VFQDIPPLNK

Tform_TR47370|c2_g2_i2.p 24 3.74e-35 LRTLNFPYNQ NSLIVPLSLTDIRLATYQYRPLSVQSFNQFFARFVLKYNGK VFQGIPPLRT

Cmite_14519_c0_g1_i2.p1 132 2.60e-33 LSRYHFKYNP SSLLAPLAFCDIRLASHLYRPVSVSSFNGFFGRHILGFNGK VFRGFPSLGR

Ctest_94376_c0_g8_i1.p1 141 7.10e-33 LRTLKVPYNL DKLIDPMGLVDIRLACYQYQPPTVEDVGQFLIKYVLKYNGV KGVRLLRT

Ctest_87731_c0_g3_i2.p1 32 1.45e-31 LRELKFPYSK EELVDPLSLIDIRLVSYKYRTLSVKTFNEFFVINILKYDGE RFQRIPPLQT

Ctest_56201_c0_g2_i1.p1 78 4.62e-31 LKQLNIAYDQ NKLITSLSLIDIRLAVNRRKPVSPQTFNQFFVQHILKAGGE NNGGIRPLNE

Ctest_82489_c0_g1_i2.p1 71 5.19e-31 LRTIKAPYDM HKLIDPMGLVDIRLACNQYQPPSIEDCSKFVLKNVLKHHKD NFEGVRPLRT

Cmala_16752_c0_g1_i1.p1 164 6.04e-30 LRRLHFPHKY SYLIRPLSLLDFHLACHFYRPVSKLKFNKFFVRYILRFNGK VFPPLPRPSK

Ctest_87731_c0_g1_i1.p1 4 7.50e-30 YRK NELLAPLSLTDIRLATYIYKPRSIQNFSRFFTRFILKIRGR PFPEFPPLNK

Ctest_194197_c0_g1_i1.p1 89 1.04e-29 LKALHLPHDL DKLIDPMALVDIRLASYQYQTPRVEDFAHFLVVYALKYNGN KFEDVQPQET

Ctest_94376_c0_g9_i3.p1 266 3.73e-29 LKALSFPHSK NKLIMPLSLLDIRLAAGGHYPVDMDSFNQFFIRKLMKFNGK TFQALPSADQ

Cmala_24475_c0_g1_i1.p1 134 4.60e-29 LKKYGFSHGA NALVAPLALLDMRLGSHLYRPVNSHYFNKFFARMILGSNGK YVGAIPPLQK

Cmite_21231_c0_g1_i2.p1 253 5.10e-29 LAGLKIRTSP SDLLAPLALSDIYLASNLVRPTSLSLFNRFFLRNILGIKGR VVGRPSLPSI

Ctest_94376_c0_g6_i1.p1 32 7.74e-29 LETLDFPYDV DRLIDHLSVVDIRLASYQYQIPSTQAFGQFVIKQIFKYSGN EFRAIRPVGA

Cmite_21231_c0_g1_i2.p1 50 8.59e-29 LAGLKIRTSP SDLLAPLAISDIYLASNLVRPTSPSLFNRFFLRNILGIKGR VAGRPSLPSI

Ctest_94376_c0_g2_i1.p1 23 2.65e-28 LRTVNFYDNI NKLIDHMSLVDIRLVCQQYQTPSVEEFGRFITKYALQYRQR EFEGIRPLKT

Cmite_21231_c0_g1_i2.p1 457 4.85e-28 LQRHGLRTSA AALTAPLAIIDIHLACQLLQPVSLSAFRDFFIGQILGISGK IIGSQYSLPS

Cmite_67826_c0_g1_i1.p1 19 9.95e-27 GSHLGLQIPV GALVDPLSLADIYFSGHLVYPVSVAAFNKFFVSHIWGGHGG SVYDHVLGIG

Ctest_94376_c0_g9_i3.p1 65 2.55e-26 FKRLNVPYNK DEIMIPLSFIDNRLISYQYQPLNAQTFVRFFVKYVLKYGGE DLQDISTISK

Cmite_21231_c1_g1_i1.p1 142 1.45e-25 LAGHGLRVPA AALLSPLAVTDIRLVANLLKPVSPQVFGGFFRSKILGLGGK IVGTPTLPSI

Cmala_9305_c0_g1_i1.p1 72 4.05e-24 VRRLGVHVTP QALVEPLALIDARLAASFVTPVSVPTFCNFVGSQILHAVGG RRVSLPPLHT

Cmite_12453_c0_g1_i3.p1 168 4.92e-22 LGALGLHVPR AALVDPLWLTDFRVAGSLLAPISPPVFSKFFVNRVLNVHRG LPSHAVPVIP

Chunt_14710_c0_g1_i1.p1 118 2.63e-18 LGARGIHLPR YRLVDSLALSDYFLGLNAVRPVSTGLFSQFYTGQLLGLTQP FYNSFTFPAY

Syats_lar_TR23764|c0_g1_ 142 5.94e-17 FLSRGLRISR SDLINRLPLFDTVFARSLRSPMTVGQFGDFFFDQFSGISRI PTIRTISSRL

--------------------------------------------------------------------------------

--------------------------------------------------------------------------------

Motif VHKYKKQYSGVRADLLQLAALRYYGL MEME-3 sites sorted by position p-value

--------------------------------------------------------------------------------

Sequence name Start P-value Site

------------- ----- --------- --------------------------

Aamph_106899_c1_g1_i1.p1 491 1.17e-23 NKYSTSIRQL VLKHKKQYSGFRVDLLQLAALRYYGI KQDYSVKFIV

BAL22342.1 479 2.11e-23 RVYGPKIKRI VHQYKSRYSGIQADLLQLCAIRYYSL PVVFRSSYSF

Ctest_99265_c0_g2_i11.p1 359 3.74e-23 QTHADSIKKM VLKYRKHFTGYRLDLLQVAVLRYYGM SQTSQYAVSF

Aamph_106899_c1_g1_i1.p1 235 4.31e-23 QVYRQAIIKV VLKYKKIYTGYRLDLLQVAALRYYGL KKNTKYEVSF

Ctest_99265_c0_g2_i11.p1 487 6.57e-23 QAHAASIKTM VYKYRKHFTGYRLDLLQVAVLRYYGM SQTSQYAVSF

Ctest_99265_c0_g2_i11.p1 85 2.56e-22 QMYAPFISGI VHSLKKRYRGVAGDLLQLAALRHYGC EPQVGNHLWL

Mlong_27848_c0_g1_i2.p1 402 7.28e-22 QTQSSAIVAT VNQYGAQYSGVRGDLLQLCVIRYYAM PAAAREEVTF

BAL22342.1 227 1.07e-21 RSQRKAIRRL VSKYRSRYSGVQLDLLCLAALRYYGV PRTARYVVDF

Ctest_99265_c0_g2_i11.p1 231 1.76e-21 RAHISSIKQM IIKYRTQYTGYKADFLQLAALRYYGM PRTSPYLVSF

Ctest_97758_c3_g2_i5.p1 462 1.76e-21 RARDTRIQKL VDKHRKNYSGNKYDLLQVAALRYYGI SETSEYPISF

Aamph_106899_c1_g1_i1.p1 363 3.26e-21 KKYRLKIKKL VLKYKRQYKGYQLDLVQIAALRYYCI SKKSKYSVSF

Tform_TR5993|c0_g1_i1.p1 82 9.57e-21 SQVGGQIRSL VHSMQGQYTGVESDLLKLAALRYYAL PISIQRRVEF

BAL22342.1 355 2.70e-20 KKYAKKIKKF IRKYKKKYSGYRSDLLQLAAIRYCLY PRSYPIKFST

Aamph_106899_c1_g1_i1.p1 76 3.39e-20 RKYGPYISRT VLPLTKQYKGVLADLLQLAVVRYYGC EPVINKKVPL

Cmala_24475_c0_g1_i1.p1 414 5.30e-20 RKMRQRIKGI IHNIRKHYDGVRADLMQLSALRYYQL PEDTRSDISF

Aamph_106899_c1_g1_i1.p1 627 5.30e-20 QKYGPKIRST VYRYRPNYSGVLTDLLQLCAIRYYSL PLVIRSNVGF

Cmala_16752_c0_g1_i1.p1 304 8.22e-20 RQLQGQLQRL IHRLTKQYSGLRRDLLKLCALRYYAF PLRIRRNVHF

Mlong_27848_c0_g1_i2.p1 278 9.17e-20 TLFGEDMNRL VMQHAEQYSGSRGDLLQVAALRYFGA GGSYSVQFSV

Ctest_97758_c3_g2_i5.p1 206 9.17e-20 STYRSIIVQL VHKHRHSARGYQTDLLQLAAMRYFAV PDSPPHIVSF

Ctest_97758_c3_g2_i5.p1 334 1.27e-19 QAFSIGIKKI VSKYIKRYSGYQADLLQLAVMRHFIV YETDHYAVSF

Cmala_24475_c0_g1_i1.p1 277 1.27e-19 RKLRGDIQRL IHNAKKQYDGVRSDLHMLAALRYYQF PKTTRVNIRF

BAL22342.1 83 4.05e-19 QKYAPVIKRI VLPMRRKYSGILGDLIQVAVIRYYGC EPVIGSSIHL

Ctest_97758_c3_g2_i5.p1 80 1.37e-18 QLHGADISHI VHSNKGQYTGHRADLWQLSALFFYGV PKASRTVSFE

Mlong_30790_c11_g1_i1.p1 231 2.37e-17 RQYNSTIVSK VAQYQNQYQGVKRDLWKLVVLEYYGT TSEPNVNAAL

Mlong_15653_c0_g1_i1.p1 113 3.41e-17 AIKQYGNDIA EKVAESQYHGIQRDLWQLVVLDYYCG GQQNSDNFPL

Ctest_87731_c0_g3_i2.p1 172 3.25e-16 SQVGRRIRAA FDTMKSLYTGVQSDLLKLAALQYFAM SASTKSHVEF

Mlong_3921_c0_g1_i1.p1 41 2.30e-15 AIKQYGNDIA EKVKEKKYKGIQGDLWQLVVLDHFSR RQQNSENIRL

Cmala_24475_c0_g1_i1.p1 549 2.30e-15 ATLSSNVRSL ISGLTPSCRGLRSDLLKLSAMRYYGL PKRIRVNVRF

Mlong_1830_c0_g1_i1.p1 96 1.45e-14 RKYKERIRSG LASAKDQYNGVSGDLLQLSALVYNSG EDVGSYKVSL

Mlong_56954_c0_g1_i1.p1 93 1.82e-14 ILKQYANQIK MVVNQYKYNNYNRDLLQLSAIEYYSL PFLTRRKVSL

Mlong_20086_c0_g1_i1.p1 84 2.44e-14 RKHKEAIKEL VSKHMDTYVDSDRDLLILSAIRYYLI PADTRSRVNF

Cmite_14519_c0_g1_i2.p1 267 1.37e-12 IRLPNINRLI HSLLKLYSGVYRTDLLPLLALRYYSF PTGVRRNLNF

Cmite_21231_c0_g1_i2.p1 591 2.97e-12 LKPHSINALI HPLQKLYSGAYRTDLLPLLALRYYSL PLRIQRNVNF

--------------------------------------------------------------------------------

--------------------------------------------------------------------------------

Motif YSASYVSQFLSGFSKYJ MEME-4 sites sorted by position p-value

--------------------------------------------------------------------------------

Sequence name Start P-value Site

------------- ----- --------- -----------------

Aamph_106899_c1_g1_i1.p1 130 6.65e-14 KKTKIPSPYR YNTKYVRTFLDGFKVYL RRHLQRPQKI

Mlong_3921_c0_g1_i1.p1 95 2.01e-13 KTTYIASPFA YNSVYVHSFLSGFKSYL QTSH

Mlong_15653_c0_g1_i1.p1 22 2.30e-13 KTANIASSFA YNSIYVHTFLSGFKSYL QTFHILQKQL

Cmala_24475_c0_g1_i1.p1 329 2.30e-13 YLGQSQSPSR YSPEYVSAFLRGFSDYV RRYRPQKPAH

BAL22342.1 137 8.13e-13 KRQSMPSPYK YNAKYVTGFIRGFMGYM HKNYKPSQLV

Mlong_22409_c0_g1_i2.p1 148 9.18e-13 DNTTVPTPED YTENYVTSFLEEFKKHI LPDPIPPLSS

BAL22342.1 530 2.62e-12 QYLGSQNLKV YNASTVKRFINGFVSYI RKRQSNKYSG

Ctest_87731_c0_g3_i2.p1 224 2.62e-12 YIEETQIPDS YSADYASRFLTGFEDHV HQLHLKNHVH

Ctest_99265_c0_g2_i11.p1 410 4.10e-12 QALKVHTLDQ YSVTYVSQFLVHFSKIL ITPPYPGYKM

Mlong_1830_c0_g1_i1.p1 150 5.11e-12 KKNKAPLAKK YKASYVKSFLKSFKKHL GELNKKQESQ

BAL22342.1 278 5.11e-12 HSLKSTAIVH YNPSYVRTFLSRFSTKL VTLPYPGYDM

Aamph_106899_c1_g1_i1.p1 286 8.73e-12 KILKPSIRIK YSVTYVRQFLVRFSRVL VAVPYPGYSL

Aamph_106899_c1_g1_i1.p1 679 9.69e-12 YIRSQKLSRQ YSPALVKKFLSGFIKYI RKNNFQKYSG

Mlong_27848_c0_g1_i2.p1 186 1.08e-11 TTTKTPSSAD FNVRYITRFLNGFKGYV SKNAGQQSVV

Tform_TR5993|c0_g1_i1.p1 134 1.99e-11 YLDRTQIPSR YTTQFTSSFLTGFERYI QRYRLKNHNY

Mlong_27848_c0_g1_i2.p1 325 1.99e-11 FEEALTPISE YTVSYVTSFIDRFGQYL STPMYAGYSL

Mlong_15653_c0_g1_i1.p1 167 2.43e-11 KIADIPSPPA YNNLYVQVFLDGFESYL RVSTKLLRSS

Cmala_24475_c0_g1_i1.p1 466 2.43e-11 YLSQRKNPGQ YSRHYVRDFLQGFGSFI RQRRRSAPIT

Ctest_97758_c3_g2_i5.p1 385 3.28e-11 QALKANPIRQ YSVSYVRQFLVSFSRIH ITQWYPGVGL

Wmill_16510_c0_g1_i1.p1 137 7.77e-11 RPKKKGEIFL YSAANVKLFMNGFIEFI NSKQTGR*

Mlong_27848_c0_g1_i2.p1 453 9.37e-11 AYFRTVTLTQ FNAAFVSEMLSGFVEYI QNQLDDYSGE

Cmala_16752_c0_g1_i1.p1 359 9.37e-11 SIKVPTVYTK YTPTYVYRFIIRFERYV RTHALKKYSG

Lanti_24997_c0_g1_i1.p1 110 1.03e-10 SGSLLSSYRT YDSRFVSAALNSFDKYC RGMSHGRYRG

Ctest_99265_c0_g2_i11.p1 139 1.13e-10 QRMKMPHSKN YNIKFVISFLNGLKGYM SKLRQPPKVD

Chunt_14710_c0_g1_i1.p1 327 1.35e-10 TGNLLTGHST YSTQFVSQTLESFDTFC RGKSDSRYSG

Mlong_24074_c5_g7_i1.p1 129 1.94e-10 AEKQNAATLV YTVENVKNFLLSFDGEL AETFPPLGLL

Aamph_106899_c1_g1_i1.p1 414 2.13e-10 KTIKLSIRQK YSIKYARIFLERFSKVL VTLPYPGYNL

Cmite_21231_c0_g1_i2.p1 643 3.30e-10 YLSSIKVPQR LSASYVRGFLRGYSDYI RHRVPKKYTG

Ctest_97758_c3_g2_i5.p1 257 5.54e-10 KALQVYEPTK YTVRYATQFLVRFNHTL TAPPYPGYKL

Cmite_14519_c0_g1_i2.p1 319 1.27e-09 FLRSIKIPGR LSSRYVRGFLGRYSKYI RHHYLKKYSG

Cmite_12453_c0_g1_i3.p1 372 1.27e-09 GSSLIPGGHS LSRGFVSGLLNGFDSYI HGLLNDYDGS

BAL22342.1 402 1.38e-09 FQRALSSYST YSVSSVSSFLGLFTQYL KKPAYVGYNL

Mlong_62463_c0_g1_i1.p1 147 3.59e-09 DTEKKKLSTT YTVDDAYKFVSRFITYM RDDPDYFYKM

Cmala_9305_c0_g1_i1.p1 275 3.88e-09 ASLRVKRAVV FDRSFVSGLLTSFDSYL HSHVKGFRGR

Mlong_30790_c11_g1_i1.p1 146 4.19e-09 SLDASALGSD YTEEYALARLSGFWSFL LANADLSPFR

Syats_lar_TR23764|c0_g1_ 331 4.19e-09 SQDRFGDLRT FNIDFVSRLLLNFDNYL RRLLHGNYLP

Mlong_20086_c0_g1_i1.p1 137 4.53e-09 FAEQRATTYR YNAEIARRFCVLYIRYI KRSLQIRGTL

Aamph_106899_c1_g1_i1.p1 538 4.53e-09 FRKALVPSFK ISTRSIKAFLVRFSKYL LKPVYISPVY

Ctest_99265_c0_g2_i11.p1 282 5.27e-09 KSLAGPSQFS NGIAFVSQFLIKFSKTL ITPPYPGYKM

Cmala_24475_c0_g1_i1.p1 601 6.14e-09 YLRRYKIPRH WNSDAAYQYLTGFDKFV RTHKLRKYSG

Mlong_23544_c0_g1_i1.p1 190 2.14e-08 SVLASAPAGQ YTADSVFQHLQSFREEI AKDIDKVNEK

Mlong_32972_c3_g29_i1.p1 138 2.65e-08 LFAVQVKTDE YSAYIVKTLLNLFRKVL ECAGEEEDDD

Mlong_56954_c0_g1_i1.p1 12 2.85e-08 ASERIPSPES YGASIVPKYMKCFKTHV EKLNEKMADH

Mlong_30790_c11_g1_i1.p1 285 2.85e-08 TGRTIPAASL YTDVYVLTFLQGFASPS GGK*

Mlong_25582_c0_g1_i1.p1 136 4.64e-08 FLDTTEPLSP YGPGTVEDYLDAFFKYM EKSDLA*

Mlong_62806_c0_g1_i1.p1 170 9.13e-08 FLNKVVAPIT YNDEYFSTFVANFLSIV RGKKTP*

Mlong_24074_c5_g7_i1.p1 252 1.04e-07 FSEAVPSSDE NSPAITKNFLDSFVTRL NTPLPELPDL

--------------------------------------------------------------------------------

--------------------------------------------------------------------------------

Motif DYGPAIRLVQDRYPGLSRLAAAYIJRYLLTNKQLPIYGRIKY MEME-5 sites sorted by position p-value

--------------------------------------------------------------------------------

Sequence name Start P-value Site

------------- ----- --------- ------------------------------------------

Ctest_94376_c0_g8_i1.p1 53 5.94e-42 AMLNGLLPTT AYHAAIKLVTDRYHGLSRIAAAYILRFLLTNKQLPVFGRIKY NEAPTLPGRL

Ctest_94376_c0_g2_i1.p1 136 2.49e-38 AILNGLLPTT AYPAAIKLVTDRYTGLSRMAAAYILRFLITNKQLPIYRQMKY NAVPVSSGRL

Tform_TR47370|c0_g1_i2.p 3 5.37e-38 TS AYEVAIRLVLDRYSGLSRMGAAYVLRFLLTNRQLPIYSRIKY QAVPVLSGRL

Ctest_194197_c0_g1_i1.p1 1 5.54e-37 . ASQEAIKLVTNRYNGLSKIAAAYILRFLLTNKQLPIFNGMKY RSVPVLASRL

Ctest_94376_c0_g9_i3.p1 178 3.55e-35 VILKGLLPAN SYGAAIRLVVNRYPGLSRTGAAYVIRFLLTNKQLPVYRHTKY GAVPAMPGRL

Cmite_21231_c0_g1_i2.p1 368 2.76e-34 KILGGLLSPR DIVPATRLVQTRYPGVSSLAAAQIISYILRSQQLPIFGKIKL KKVPAVAARL

Cmite_14519_c0_g1_i2.p1 44 9.77e-34 PILGGLLNPS DYGVAIRLVQRRYSNLQSVAAAHILRYILRPQRLPIYGRIKY RTVPIISRRV

Cmala_24475_c0_g1_i1.p1 47 2.82e-33 LVLTDILAPA HRGKAIVLVQRRYPGLSRLAAAHGIRYLLSQHRLPIYQRIKM QGVPPLSDRL

Cmala_16752_c0_g1_i1.p1 76 6.18e-33 VILRGLLPRK HYRLALKLVLPRYPGLDRDGAAYVIRYLLTHRRLPIFGRVKY HTVPALPLRL

Cmite_21231_c0_g1_i2.p1 165 7.57e-31 KILGGLLSPR DIVPATRLVQTRYPGVSSLAAAQIISYILRSKQLPVFGQLGI GQIPAVSGRL

Cmite_12453_c0_g1_i3.p1 80 2.09e-25 LDLGGLLSAG DVGQAIELVQTRYSGISSPAAVQIIRLAVSQPPAAILPRIHF RTVPSLGRRL

Chunt_14710_c0_g1_i1.p1 30 1.01e-23 HSLYTYLLPS DIAPALSLVRSRYVGISQPCAYSIIEHALTHPHNGFLGRINL SFPSLRSQRL

--------------------------------------------------------------------------------

--------------------------------------------------------------------------------

Motif PALSGRLEFLRLLFRALPPVF MEME-6 sites sorted by position p-value

--------------------------------------------------------------------------------

Sequence name Start P-value Site

------------- ----- --------- ---------------------

Tform_TR47370|c0_g1_i2.p 48 2.37e-22 IYSRIKYQAV PVLSGRLEFLQLLFRALPPVF YSGQYQSDLF

Ctest_94376_c0_g9_i3.p1 223 1.11e-20 VYRHTKYGAV PAMPGRLQFLQHLFRALPPVF YTGQYQRNLL

Ctest_56201_c0_g2_i1.p1 35 2.17e-20 FNRHMKRHTI PALAGRLEFLQLLFRALPAVF YSGRYQSSLT

Ctest_94376_c0_g2_i1.p1 181 1.57e-19 IYRQMKYNAV PVSSGRLQFLRQLFEALPPVF YFSQYQKKLT

Ctest_94376_c0_g9_i3.p1 22 6.09e-19 VFNRISYNAV PALSGRMKFLQLLFKSLPPVF YHGQFQANML

Ctest_94376_c0_g8_i1.p1 98 1.89e-18 VFGRIKYNEA PTLPGRLDFLKQLFHELPPVF YSDKYHNKLV

Cmala_9305_c0_g1_i1.p1 29 4.32e-18 LLRAVRLQTV PVLEQRLAFLRAVFGALPPVF YRANYRRALV

Cmite_21231_c0_g1_i2.p1 210 2.05e-17 VFGQLGIGQI PAVSGRLEFLRLYFSRLPVVV YNQGISNDLA

Cmite_21231_c0_g1_i2.p1 7 2.05e-17 LGIGQI PAVSGRLEFLRLYFSRLPVVV YNQGISNDLA

Ctest_82489_c0_g1_i2.p1 28 2.81e-17 VFGRIKYNGV PILSRRLNFLKLLFPQLPPVF YSGKYRKSLI

Ctest_194197_c0_g1_i1.p1 46 6.41e-17 IFNGMKYRSV PVLASRLKFLEELFLELPPVF YSGKYQRNLI

Cmite_21231_c0_g1_i2.p1 413 3.13e-15 IFGKIKLKKV PAVAARLPFLRSVFRALPAPV LVSRVYSGGL

Cmite_14519_c0_g1_i2.p1 89 4.40e-15 IYGRIKYRTV PIISRRVAFLRSYFGALPGVF YHPTYYGGLR

Cmala_24475_c0_g1_i1.p1 92 9.29e-15 IYQRIKMQGV PPLSDRLPYLQRLVAAMPPVF YTRYQNGLLH

Cmala_16752_c0_g1_i1.p1 121 1.19e-14 IFGRVKYHTV PALPLRLAFIRKVWPLLPPVF YRGKYHASLL

Cmite_21231_c1_g1_i1.p1 98 1.78e-14 IFSRLALGPI PAISGRLAYLTSVFSRLPAPV VFGVRFSGAL

Cmite_12453_c0_g1_i3.p1 125 2.11e-12 ILPRIHFRTV PSLGRRLVYLRRLIAALPPPI ALQSYLPSVE

Syats_lar_TR23764|c0_g1_ 99 2.25e-12 NIPGLRVDAP VPAAQRLKFLRQSFRLFPRVF FDDHFVNSLQ

Chunt_14710_c0_g1_i1.p1 75 9.01e-12 FLGRINLSFP SLRSQRLGYLRTLFSALPSQV FYTSYIDPVS

--------------------------------------------------------------------------------

--------------------------------------------------------------------------------

Motif MLRAVLLAALAAAAAAAQGS MEME-7 sites sorted by position p-value

--------------------------------------------------------------------------------

Sequence name Start P-value Site

------------- ----- --------- --------------------

Aamph_106899_c1_g1_i1.p1 1 1.98e-16 . MFRPVLLLALAAAAAAVGTY PGFTLKPVFS

Cmite_14519_c0_g1_i2.p1 1 2.71e-16 . MFRAFLVVALAAVAAAHGGH HPGPIYVRPN

Cmala_24475_c0_g1_i1.p1 1 2.50e-15 . MLRSVLLAVLALAAAAHNDD DFFSSPVVVR

Mlong_11150_c0_g1_i1.p1 1 5.70e-15 . MFRPVLFAVLMAGICSGTGD SRVPGAGDPT

Mlong_23404_c0_g1_i1.p1 1 2.09e-14 . MLRASLLTALAAGMAAAQNI TTLKTVFSSV

Mlong_62806_c0_g1_i1.p1 1 1.79e-13 . MLRPVLFAVLMAGISSGTED NGVPEAGDPT

Cmite_21231_c1_g1_i1.p1 4 2.00e-13 MAD VLRALLLTALAAAAAAHFGG GDGGGTVVVR

Wmill_16510_c0_g1_i1.p1 1 2.00e-13 . MLRVVLLVTLMVVASAKRDE RAKQKEQLRT

Cmala_16752_c0_g1_i1.p1 1 2.80e-13 . MLRVLVLTWLAAAAAGCRFK SCHPPHPHPH

Mlong_22409_c0_g1_i2.p1 1 1.02e-12 . MFRPVLFALLTAISCSQRGH NGVPGARELV

Mlong_26441_c1_g5_i3.p1 1 1.71e-12 . MLPALLLFALTAAAAGAPQP VTVTGVLRPS

Chunt_14710_c0_g1_i1.p1 1 2.09e-12 . MFRILLLAAVLAVATPHGYH SLYTYLLPSD

Mlong_32972_c3_g29_i1.p1 1 5.11e-12 . MFGAALLLSLTATAAVATYP GHEYGKILKE

Mlong_27848_c0_g1_i2.p1 48 6.82e-12 TAPPVRISQT MMRSMLLLALSVTVAIVASS PATTRLKDRV

Ctest_99265_c0_g2_i11.p1 1 6.82e-12 . MLRTVLVAALTVTAIVSAGN GVKQASHPYH

Mlong_30790_c11_g1_i1.p1 1 9.99e-12 . MFRFTFLTVLSAAASLAPSS ASPVQQPVIT

Mlong_25582_c0_g1_i1.p1 1 1.21e-11 . MLRLTVLCVLATTVICVASE QSPFKPDGDV

Mlong_62463_c0_g1_i1.p1 1 1.75e-11 . MLQPLLLVLLWAAATVADGA PENYAGKPLA

Syats_lar_TR23764|c0_g1_ 1 2.30e-11 . MIRAVLFSALICVAFATPYG KGLKGKLFLE

Cmite_12453_c0_g1_i3.p1 1 2.30e-11 . MLRLVLVAALVARSLATFGP GYGFHDVLGG

Mlong_24074_c5_g7_i1.p1 1 3.30e-11 . MLGPMLMMVLSGAASAQTSL TGASLQPALT

BAL22342.1 2 3.30e-11 M LLRPVLLLAALAALAAATGS RPYFPVSSLK

Mlong_20508_c0_g1_i1.p1 1 4.71e-11 . MFHAAFVFVLVAVAAGQQTS ISSAVGQITP

Mlong_14009_c0_g1_i1.p1 1 5.62e-11 . MLRSSLLLVVSAVVTLGQDS APESFPGSEY

Ctest_97758_c3_g2_i5.p1 1 2.20e-10 . MLCTVALITVLTATAASQKS YAPGFDRRLE

Mlong_6982_c0_g2_i1.p1 1 4.58e-10 . MLRALLRVMLLLSLTAAKKE EYYGEEYLQN

Mlong_1830_c0_g1_i1.p1 1 6.30e-10 . MSRLLLLIALVTITGAWSPP YKRKTPTSFG

Mlong_24434_c0_g1_i1.p1 1 1.18e-09 . MMRAAVLISVSVAIVLGQEY PLPAGCDCQT

Mlong_23544_c0_g1_i1.p1 1 3.79e-08 . MFLLVLLLGLSLSTITTASP AGKDNKTPLK

--------------------------------------------------------------------------------

--------------------------------------------------------------------------------

Motif YSGQYQKSLLSYLRTLNFPYN MEME-8 sites sorted by position p-value

--------------------------------------------------------------------------------

Sequence name Start P-value Site

------------- ----- --------- ---------------------

Tform_TR47370|c2_g2_i2.p 2 5.29e-22 F YSGQYQSGLFAYLRTLNFPYN QNSLIVPLSL

Tform_TR47370|c0_g1_i2.p 69 7.08e-21 LLFRALPPVF YSGQYQSDLFAYLRTLNFPYS QDKLIVPLSL

Ctest_194197_c0_g1_i1.p1 67 3.56e-19 ELFLELPPVF YSGKYQRNLISYLKALHLPHD LDKLIDPMAL

Ctest_94376_c0_g9_i3.p1 244 1.31e-17 HLFRALPPVF YTGQYQRNLLTYLKALSFPHS KNKLIMPLSL

Ctest_94376_c0_g2_i1.p1 202 1.43e-17 QLFEALPPVF YFSQYQKKLTAYLEALNFPYA VTMLIVPLSF

Ctest_94376_c0_g2_i1.p1 1 3.12e-17 . YSVKYQKNLTSYLRTVNFYDN INKLIDHMSL

Ctest_87731_c0_g3_i2.p1 10 3.40e-17 LFPALPPVF YPSQYQGSLASYLRELKFPYS KEELVDPLSL

Cmala_16752_c0_g1_i1.p1 142 3.70e-17 KVWPLLPPVF YRGKYHASLLSYLRRLHFPHK YSYLIRPLSL

Ctest_82489_c0_g1_i2.p1 49 6.15e-17 LLFPQLPPVF YSGKYRKSLISYLRTIKAPYD MHKLIDPMGL

Ctest_94376_c0_g9_i3.p1 43 2.91e-16 LLFKSLPPVF YHGQFQANMLAHFKRLNVPYN KDEIMIPLSF

Ctest_94376_c0_g8_i1.p1 119 3.41e-16 QLFHELPPVF YSDKYHNKLVYYLRTLKVPYN LDKLIDPMGL

Ctest_56201_c0_g2_i1.p1 56 1.09e-15 LLFRALPAVF YSGRYQSSLTARLKQLNIAYD QNKLITSLSL

Ctest_94376_c0_g6_i1.p1 10 1.27e-15 LFQVLPPVF YVSPYRESLASYLETLDFPYD VDRLIDHLSV

Cmite_14519_c0_g1_i2.p1 110 2.07e-12 SYFGALPGVF YHPTYYGGLRSYLSRYHFKYN PSSLLAPLAF

--------------------------------------------------------------------------------

--------------------------------------------------------------------------------

Motif TPPYPGYELLPVLRS MEME-9 sites sorted by position p-value

--------------------------------------------------------------------------------

Sequence name Start P-value Site

------------- ----- --------- ---------------

Ctest_99265_c0_g2_i11.p1 428 1.43e-14 FLVHFSKILI TPPYPGYKMAPILQR IGLPGLKYPQ

Ctest_99265_c0_g2_i11.p1 300 2.59e-13 FLIKFSKTLI TPPYPGYKMAPLLQR IGLPGLKYPQ

Ctest_97758_c3_g2_i5.p1 275 3.25e-13 FLVRFNHTLT APPYPGYKLTPILKK MGLPALKDVE

Mlong_30790_c11_g1_i1.p1 172 4.06e-13 LLANADLSPF RPAYPGYELISVLQE IGLHALTYPR

BAL22342.1 168 7.77e-13 KPSQLVVPVV KPTYPGYGLLTVLQS VGLPALTNPR

Aamph_106899_c1_g1_i1.p1 304 1.18e-12 FLVRFSRVLV AVPYPGYSLLPVLRG IGLPKLYRPK

Ctest_99265_c0_g2_i11.p1 172 1.97e-12 PKVDIAPVPT PAPYPGYELLTVLKS AGLPGFHNPR

Mlong_15653_c0_g1_i1.p1 56 2.17e-12 KQLVPVTPLY KPAYPGHELLSTLTE MGLPALEYPE

Aamph_106899_c1_g1_i1.p1 176 1.10e-11 IFPIFPVRPP KPSYPGYEILATLKS LGLPPLVNPR

Mlong_27848_c0_g1_i2.p1 219 3.48e-11 QSVVFPDPPP APSYPGSELLATMRS IGLPTLTNKQ

BAL22342.1 296 1.03e-10 FLSRFSTKLV TLPYPGYDMITIFRG FGLPKLYQPR

Ctest_97758_c3_g2_i5.p1 147 1.12e-10 FLGRFSQALV TPTYPAHKLLAVLRR VGLLHFHNTA

Cmite_14519_c0_g1_i2.p1 206 1.42e-10 PRIFQSIVSV SVSYPGSSLLPIWSG IGLPALSQPQ

Aamph_106899_c1_g1_i1.p1 432 1.42e-10 FLERFSKVLV TLPYPGYNLIRVFRG IGLPKLYRPK

BAL22342.1 420 1.96e-10 FLGLFTQYLK KPAYVGYNLKPVLLQ AGLPKLSMPQ

Ctest_97758_c3_g2_i5.p1 403 5.31e-10 FLVSFSRIHI TQWYPGVGLEPHLKI IVPSGLDYAK

Aamph_106899_c1_g1_i1.p1 568 5.31e-10 VYISPVYKPR PATYPGYQLKPLLTT VGLPKLTKPQ

Mlong_23544_c0_g1_i1.p1 83 1.19e-09 AEDTPPDNAA APTFSGSEYLPILTE IQVDLHDEEP

Mlong_27848_c0_g1_i2.p1 343 2.99e-09 FIDRFGQYLS TPMYAGYSLRSRLVS AGLPVMSAPH

Cmite_21231_c0_g1_i2.p1 530 4.83e-09 YSSFVPAVFQ RIGYAGSSLLPIWSR IGLPALSQPQ

BAL22342.1 22 9.39e-09 LAALAAATGS RPYFPVSSLKPVLSG IGLPAFYKPD

Ctest_99265_c0_g2_i11.p1 26 2.03e-08 VSAGNGVKQA SHPYHGGNLRHVLAS MGLPEFAQPN

--------------------------------------------------------------------------------

--------------------------------------------------------------------------------

Motif RYNVSFGDAFRKYLK MEME-10 sites sorted by position p-value

--------------------------------------------------------------------------------

Sequence name Start P-value Site

------------- ----- --------- ---------------

Ctest_99265_c0_g2_i11.p1 517 3.67e-13 LRYYGMSQTS QYAVSFSVAFRQALE VHTLHQY

Ctest_99265_c0_g2_i11.p1 389 4.18e-13 LRYYGMSQTS QYAVSFSVAFRQALK VHTLDQYSVT

Cmala_24475_c0_g1_i1.p1 579 1.38e-11 MRYYGLPKRI RVNVRFPDAFRGYLR RYKIPRHWNS

Ctest_97758_c3_g2_i5.p1 364 1.52e-11 MRHFIVYETD HYAVSFRVAFRQALK ANPIRQYSVS

Cmala_16752_c0_g1_i1.p1 334 4.01e-11 LRYYAFPLRI RRNVHFHDVFRRYLS SIKVPTVYTK

Ctest_87731_c0_g3_i2.p1 202 7.63e-11 LQYFAMSAST KSHVEFSDAFASYIE ETQIPDSYSA

Aamph_106899_c1_g1_i1.p1 265 8.34e-11 LRYYGLKKNT KYEVSFGYALRKILK PSIRIKYSVT

Mlong_27848_c0_g1_i2.p1 306 1.09e-10 AALRYFGAGG SYSVQFSVAFEEALT PISEYTVSYV

Aamph_106899_c1_g1_i1.p1 519 3.30e-10 AALRYYGIKQ DYSVKFIVAFRKALV PSFKISTRSI

Tform_TR5993|c0_g1_i1.p1 112 4.21e-10 LRYYALPISI QRRVEFADAFASYLD RTQIPSRYTT

Cmala_24475_c0_g1_i1.p1 444 4.21e-10 LRYYQLPEDT RSDISFDQAFSKYLS QRKNPGQYSR

Ctest_99265_c0_g2_i11.p1 261 4.95e-10 LRYYGMPRTS PYLVSFITAFKKSLA GPSQFSNGIA

Cmala_24475_c0_g1_i1.p1 307 4.95e-10 LRYYQFPKTT RVNIRFPDAFSRYLG QSQSPSRYSP

Mlong_56954_c0_g1_i1.p1 123 5.37e-10 IEYYSLPFLT RRKVSLGYAFQGYLS SHRTKTYN

Ctest_97758_c3_g2_i5.p1 236 5.37e-10 MRYFAVPDSP PHIVSFPEAFEKALQ VYEPTKYTVR

Mlong_1830_c0_g1_i1.p1 126 6.29e-10 LVYNSGEDVG SYKVSLLYAFKEYLK KNKAPLAKKY

Wmill_16510_c0_g1_i1.p1 110 6.81e-10 EYYYYRPTTY KAKISFIDAFKQYSE TLRPKKKGEI

Aamph_106899_c1_g1_i1.p1 393 8.62e-10 LRYYCISKKS KYSVSFGYALKKTIK LSIRQKYSIK

BAL22342.1 257 3.33e-09 LRYYGVPRTA RYVVDFDYALEHSLK STAIVHYNPS

Mlong_27848_c0_g1_i2.p1 432 4.44e-09 IRYYAMPAAA REEVTFGDTFPAYFR TVTLTQFNAA

Aamph_106899_c1_g1_i1.p1 657 6.79e-09 IRYYSLPLVI RSNVGFGSTFQRYIR SQKLSRQYSP

BAL22342.1 509 1.26e-08 IRYYSLPVVF RSSYSFGTIFQQYLG SQNLKVYNAS

BAL22342.1 383 1.44e-08 AAIRYCLYPR SYPIKFSTVFQRALS SYSTYSVSSV

Cmite_21231_c0_g1_i2.p1 621 1.54e-08 LRYYSLPLRI QRNVNFGAVTRAYLS SIKVPQRLSA

Ctest_97758_c3_g2_i5.p1 109 1.65e-08 ALFFYGVPKA SRTVSFEAAFKRSFI EPVLRYAKYS

Mlong_20086_c0_g1_i1.p1 114 1.88e-08 IRYYLIPADT RSRVNFDTTFSAHFA EQRATTYRYN

Ctest_94376_c0_g9_i3.p1 439 1.18e-07 NTKMSDMSTP QPQSRFQDAFEGYF*

Cmite_21231_c1_g1_i1.p1 119 1.09e-06 SVFSRLPAPV VFGVRFSGALASHLA GHGLRVPAAA

--------------------------------------------------------------------------------

**File S10.** Alignment of CP100k homolog sequences within the 10 MEME motif regions.

--------------------------------------------------------------------------------

Motif PSVSQCGKIAGYLQKAVPELAGGGVNIDLKSLVASAAVILHQRGVTVNLDQLNILLKTGLSGYLQSTAYQSSYSMLSQLIATLDHIDHBLPNILDQEELIAVRRALEKRFNLDSKIFDKRYRQAIKAFEANRRRJLESFNTLAYRGPDYE MEME-1 sites sorted by position p-value

--------------------------------------------------------------------------------

Sequence name Start P-value Site

------------- ----- --------- ------------------------------------------------------------------------------------------------------------------------------------------------------

Tform_TR54333|c0_g1_i1.p 106 1.52e-149 SLKELTDEVV PSAPTCGKIADYLQGAVPELAGGGVNIDLKSLVAASAVILHQRGVAIQLSQLNILLKTGLSGYLQSTAYKTSYSALSQLIATLDHIDHNLPNILDQDLLIAVRRKLETRFNLDSKIFDQRFQQAIRVFEANRQRILESFNSLAFRGPDYE VTIQVVIRET

Aamph_AGS19349.1 106 4.78e-149 SLNTLTDAVV PSVPQCGKIAGYLQKSVPGLAAGGVSLDLRSLVASASVILHQRGVTVNLEQLNVLLKSGLAGYLQSTAYQSSYGSLIQLISALDHIDHNLPNILDQESLIVVRRALESRFNLDREIFDKRYKLAIKAFEENRRRLLASFNTLAYRGPNYE TNVQLVIKQM

Majex_524_c0_g1_i1.p1 73 6.09e-148 SLSSLTDEVL PSVSQCGQIAGYLQQSVPELIGGRVNIDLKSLVASASVILHQRGVKVSLAQLNILLKTGLMAYLKSTAYQSSYSSLIQLISSLDHIDHNLPNILDQKSLLIVRRALENRFNLDREIFDKRYKQAVKSFEANRRRILASFNALAYRGPDYE VNMQAVIRTM

Ctest_109122_c0_g1_i4.p1 106 6.25e-144 SLNELTDKVV PSVSQTGEITGYLNGAVPELAGGGVNIDLDSLVVAVSTILHQRGVALPLDRLNILLKTGLSGYLQSPAYHTNYGRLSQLVATLDHIDHNLPNILDQDLLIDVRRKLETRFNLDSKIFDKRYHQAIKAFEANRARLMESFNSLAFRGPDYE VTIQTVIREV

Mrosa_BAB12269.1 107 1.15e-133 SLGSLTDSVV PSVSQCGQIAGYLQKSVPALAQGGFNVDLKSLVSSASVLLHQRGVTVNTDELNIFLKYGLINYLKSTVYQSSYSMLRQLIVTLDYLDHELPVILDYEELIAVRLALKKKFDTSVDIFKNRYQLAIQSYKANRNLLLDSFRTMAYRGPKYE MYLQEAIRET

Majex_14369_c0_g1_i1.p1 106 6.03e-131 SFGSLTDSVV PSVAQCDQIAGYLKKSVPALVGAGVSIDLRSLVSSAAVLLHQRGVTVNTDELNIFLKYGLINYLKSTVYQSSYSMLRQLIVTLDYLDNDLPIILDYKELIAVRLALKKRFNTSVNIFKNRYQLAIQSYKANRNLLLASFRTLAYRGPKYE IYLQQVIRET

Aamph_AKZ20818.1 107 2.29e-121 SFSSLTDAVL PSVSQCGKYGDYLHKATPLVISGDRKFDMCSLVASYAVILHQREVTVNFDQLNVILKLGLQKYLKSTAYQSSYSMLTQLLTSLDFIDHDLPTILDYEELIAVRRALLLRYNIKRARFDNRFRLAIEEFKLNRHRLLSTFNTIAFRGPYYE IVVQEVIREI

Cmala_14904_c0_g1_i1.p1 103 5.40e-116 GLSELTDKVV PGADVVAKIVRYLGGAVPELKGSQLNVDLSSLVPACAVVLHQRGVQLTHQQLQVFLRSALSGYLQSPAYKADYSPQSQLVACLDHFDHQLPSILELAALLPVRRQLEKRFNLESDIFDHRFRQAVKAFEANRRRILESFNTIAFRGSRYE ITIQQVIAKV

Cmite_22920_c0_g1_i3.p1 106 2.58e-111 GLRELSDSVV PSQKQVDEVVTYLGGNVPELAAGKVQLDLPSLVGSGAVVLHQRGVGISQQQLGLVLGNGLSGYLQSPAYKVDYNGQSQLVATLDSIDHAIPSILELEQLKPVRAKLESRFNLDSKIFDSRFRQSVESFERNRQRILDSFNTLASRGAGFE VSIQTVISKV

--------------------------------------------------------------------------------

--------------------------------------------------------------------------------

Motif GIQPLNLQATYEAFIYHTQRFFLSTRTYTVEAYILYVIRVVVPSIPRGSKHFRIHLFDSSVVIDNILVPEPLKSIYEEGRDTIIKRIVGLQGSSDQITRRLIEGEGEKPYVKN MEME-2 sites sorted by position p-value

--------------------------------------------------------------------------------

Sequence name Start P-value Site

------------- ----- --------- -----------------------------------------------------------------------------------------------------------------

Aamph_130017_c0_g1_i1.p1 2 1.86e-115 Q GIQPLNQMATYELFIYHSTIYFRSSCAYTVDDYFLFISRVVRPNIPLGSKHFKIISFDHSVVIENILVPEPWRSNYEKSRDTIIKRLVGLQGSSDQITKRLIEGGGEKGYIKN IVNLKP

Majex_14369_c0_g1_i1.p1 353 1.25e-114 FLFLVSILSQ GIQPLNQVATYELFIYHSTSYFQSSCTYTVDDYFLFISRVVRSNIPLGSKHFKIISFDHSVVIENILVPEPWTSIYKEGRDTIIKRLVGLQGSSDQITKRLIEGGGEKDYVKN IVNLKPAITA

Mrosa_BAB12269.1 354 8.14e-114 FLFLVGILSQ GIQPLNQMAIYELFIYHSTIYFRSSCAYTVDDYFLFISRVVRPNIPLGSKHFKIISFDHSVVIENILVPEPWRSNYEKSRDTIIKRLVGLQGSSDQITKRLIEGGGEKGYIKN IVNLKPAITP

Tform_TR54333|c0_g1_i1.p 354 5.01e-112 VLFIIGLASQ GVQPVQLQACHEAFVWHTQRFFLGTRSYTVEAYILYVIRVVVPSIPRGSVGFRLHLFDASIVIDNVLVPEPLQSIYKEGRQTIIERVRGLQGSSEDITRRLLTGEGEKPFVKN IVDLRPPIKT

Ctest_109122_c0_g1_i4.p1 354 7.50e-110 ALFIIGLASQ GVQPLKLQAAHEAFVWHTQRFFLGTRTYTVEAYILYVIRVVVPSIPRGSSAFRLHLFDSSVVINNVLVPEPLKSIYKEGRQTIIARIQGLQGSSADITRRLVRGEGEKAVVEN IVDLRPPIKN

Aamph_AGS19349.1 354 3.91e-106 SIFIIGLASQ GVQPLQLEATYEAFIWHTQRFFLATRIYSVQAYLLYVMRVVVPLIPRGSQSFRLHIFDSSVVIDNILVPEGLTSIYEEGRQTIIKRIRGLQGSSSDITNRIIGGQGEKGVIGN DLKFQTIVPA

Cmite_22920_c0_g1_i3.p1 353 6.64e-95 ALFIVGLASQ GITPLRLDETHASFIFHTQRFFLASSVFTPEAYILYVIRVVVPSIPRGSPAFSIRLFDPTIVVDEVLVPEPLRSIYEEGRKTIIPRVFGIQGNSIQITRRLAQGEGDKPPVEN PVVLRPPIVN

Cmala_14904_c0_g1_i1.p1 350 9.42e-95 ALYIIGLLSQ GVQPAKLSIVHRLFVEHTQRFFLGVPQYNVESYILYVVRVVVPSIPRGSPGFIIRLFENTVVIDNVLVPEPLKSIYQEGRDTIIPRIFGLQGNSIQISRRLARGEGAKPDVEN IVNLRPRIVS

Majex_464_c0_g1_i1.p1 89 2.67e-94 TLFIMALMSQ GIRPVDQLKTFEAFIKYTQWFFLGNHVYSVEAYLLYALRVVIAYIPRGSEFFRSHIFDSTIVVDNILVPKPWKSIYEEGRDSIIQRIRSLQGSSDQITNRLKSSIGERGYVKN IVNLKPTITA

Aamph_AKZ20818.1 355 1.87e-85 PTFLISLISQ GIQPVNTIVTYKTFYYYLQAYFQSTSSYSVESMTTFFLRTVVTSIPRGSPHFRINIFQSTVVIDNILVPQPWTSIYRKGKASILKRIVGPQGNSRNIIIRLKTGRGEKPVIQN DLKFKNIVPA

--------------------------------------------------------------------------------

--------------------------------------------------------------------------------

Motif RLQLTRTQJLAGLQQFYVASRCLGYVIPQQAJPSVFVYTVRQYLSTLPTIPAQPFDYGFLEYLYLRLASIIEQ MEME-3 sites sorted by position p-value

--------------------------------------------------------------------------------

Sequence name Start P-value Site

------------- ----- --------- -------------------------------------------------------------------------

Majex_14369_c0_g1_i1.p1 567 6.84e-71 SYIMTQLSAY RLQATKAQIKAALDQFFVATKCLGYVIPQQQIPSIFVAVVGQYLSTLPTIPKQPFDYNFLEFLHFRLASIIEQ LSAVGSQSVI

Tform_TR54333|c0_g1_i1.p 568 8.99e-71 AGIITQLSER RLQLTRTQVLAGLQQFFVASRCLGHVIPPKSLPGVFVYTITQYIQTLSKIPAQPFDYRFLQYLYQRLASIIQQ VVLINRSVPV

Ctest_109122_c0_g1_i4.p1 565 1.18e-70 DGLITQLSQR RLQLTREQLLAGLQQFYVASRCLGIVIPSSSLPGVFIYTVTQYLQTLTTIPAQPFDYRFLQYVYERLASIIEQ AVLINRSVPV

Aamph_AGS19349.1 567 2.32e-70 SFVLTQLSEN RLQLTETQLLAGIQQFYVASRCLGYVIPQQTIPSVFLYTVREYLSTLASVPAQPFGDGFLEFLYLRLAGIIRQ VTVVDQKVPI

Mrosa_BAB12269.1 568 4.15e-69 SFIVEQLREY RLQTTKAQIKAALDQFFVATKCLGYVIPQQKIPSIFLATVGRYLSTLPTIPKQPFDYNFLEYLRYSLASIIEH LPAVGSQSVI

Cmala_14904_c0_g1_i1.p1 563 2.01e-62 DVLVTQLSRK RLQLTRPQLLAGLQQFYVCSRALGHVIPRKALPGVFAAALGRYVQTLPKIPAQPFDIGFLRYLQKRLVSIIQR VVLVGDSVPV

Majex_5038_c0_g1_i1.p1 53 3.76e-62 RYILTQLSEH RLQLTESQLEAALDQFYVFSRCFGYIIPKPAIPSIFMYAVRQYLLTQSKIPAQPFDDDFLTFLDSHLPGIIRQ VAVIDNQVPI

Aamph_AKZ20818.1 568 1.76e-61 TFVFSQLTEL NLQLTETQIRGALQQFYVVTRSLGYVIPQETIHSVFVYSVREYLSTLTSIPTQPFGDGFLEFLYLRLEVIIKK VVVIEQHVPI

Cmite_22920_c0_g1_i3.p1 565 2.54e-54 DLSPLVAELR RLKLTQEQLLAGLQQFFVTTRSLGHIIPAGALPGVFSASVRRFIDTVPRPPKQPFNVDYLRYLRRRLPAIIER TVLVGNSVPV

--------------------------------------------------------------------------------

--------------------------------------------------------------------------------

Motif AISGVDIDALLKEIDERLVDATISGTGIQSGLVELYJHMHYLKLPJPSPEVRDEFFSFVIGAYGQVQVRRQLPFGKPFYEFLSGFLPKLPGYLKPFPLFAAPKLFDAFSSQLK MEME-4 sites sorted by position p-value

--------------------------------------------------------------------------------

Sequence name Start P-value Site

------------- ----- --------- -----------------------------------------------------------------------------------------------------------------

Tform_TR54333|c0_g1_i1.p 898 2.67e-119 SYYSSLSKNL AISGVDIDALLKTIDERLVDATISGTGIQSGLVELFLHMSFLKMPIPGPEVRNEFFSFCIGAYGQVQVRRQLPFGKSFYEFLSGFLPKLPGYLKPFPLFSGPKIFDAFSAQLK TRIAPSDIRL

Ctest_109122_c0_g1_i4.p1 891 3.75e-116 QYYGGLPKGI SIKGVDIDALLTSIDERLVDATISGNGIRSGLVEMYIHMHFLGLPLPGAEVRDEFFSFIIGAYGQVQVRRQLPFGKYFYKFLSSFLSKLPGYLKPFPLFAGPKLFDAFSSQLK VRIAPADIPL

Aamph_AGS19349.1 895 8.28e-107 RYYRGLPKSI SVGEFDIEDLVKEIDDQLKDATISGTGVQSALVELYLHMYYLKMPLPSVKVRDGFLSFVIGAYGKVQVRRQLPFGKLFYDFLQGFLPKLPGYLKPFPIFAGPQVYKVFHSTLK TPVYPSDIPL

Ctest_86401_c0_g1_i1.p1 150 3.64e-106 RYYSGLPRSL AISGVNIDTLLRTIDERLVDATISGAGIRSGLVELYLHMHFLGLPIPTPAVRDQFFSFILSAYGQMQVRRQLPFGRYFFTFLNSFLGKLPGYVKPFPLFAAPKIYDAFSSQLG VRISPADMPL

Majex_38903_c0_g1_i1.p1 40 5.12e-106 RYFRSLPKSI AIGALDIDPLVKEIDDQLVDATISGTGIQAAMVELYIHMYYLKLPIPSVEVRDKFLSFVLGAYGKIHVRRQLPFGKPFYEFLSGFLPRLPDYLQPFPLFAAPQLHSMLYSKLK TPIYASDIPL

Cmala_14904_c0_g1_i1.p1 891 3.12e-89 VVYITALPKF PVKGVDLAALLKRIDEQLVDVTITGIGIQSAFCELYLSLHALKLPLPSAAVRDDFFTLVVTMYGRTFIRQQIRFGVPFYEFLGKRLPKLVDYVKPFPLFAAPKLFFAFQRELK PAPVPVADIR

Cmite_22920_c0_g1_i3.p1 894 1.52e-78 SFVSSLPADL AVSALDVEALRKAIDGSIHDATVTGNGIQAGVVQLLISMHGLALPLPKGADRDSFFTFVIVQFARTVRRQQLRFGAPYHAFLGGFLPKLTGLVRPYPLFSAPQLFSLFEKQLS PTLLRIDDLP

--------------------------------------------------------------------------------

--------------------------------------------------------------------------------

Motif FEYKNVLLSSVQLQSVAAELEKRFEGLKQPSLQLPLLRILVRABVISDTGDKAAAA MEME-5 sites sorted by position p-value

--------------------------------------------------------------------------------

Sequence name Start P-value Site

------------- ----- --------- --------------------------------------------------------

Wmill_88483_c0_g1_i1.p1 27 8.67e-58 TAGPVPTYDA FEYKNVLLSSQHMQSIALELEKRFEGLKKPSLRLPLLQILVRADIITDTGDKAAAA FLRLFQGLPK

Majex_14369_c0_g1_i1.p1 484 8.67e-58 TAGPVPTYDA FEYKNVLLSSQHMQSIALELEKRFEGLKKPSLRLPLLQILVRADIITDTGDKAAAA FLRLFQGLPK

Mrosa_BAB12269.1 485 6.38e-56 TPRPQPTYDA FEYQNVLLSSQHMQRVAFELAKRFEGLKEPSFRLPLLKILVRANLVTDTGDKAAAA FLRLFQGLPV

Aamph_124352_c0_g1_i1.p1 9 6.38e-56 RPQPTYDA FEYQNVLLSSQHMQRVAFELAKRFEGLKEPSFRLPLLKILVRANLVTDTGDKAAAA FLRLFQGLPV

Aamph_AGS19349.1 484 7.20e-46 VPADVPGYDQ FEYQNVILSAVQMREIASVLIQRFNQLKQPSLQLPLMRIMIHANVIPNSGAAAAAA FRRLFRGLPA

Tform_TR54333|c0_g1_i1.p 485 9.10e-46 KTGPLPTYSK FEYEGVILSVAHLQAIAYELQRRFDQLKQPQLQLPLLRVLIRAHVVRGSGDKAAAA FRRLFSGLPR

Mlong_56399_c0_g1_i1.p1 46 1.92e-42 VPGSVPTYNT FEYEGVILSALELQEVAGTLARRFHYLKKPSLQLPLLRILVRAKIVTDTDTRAAVA FQRLFQGLPL

Aamph_AKZ20818.1 485 4.31e-38 VPADVPGYDQ FEYQNVILSAIQLSQVASALIQRFNLLKQPSLQLSTLRIMIRAGLIKGTGVQAANA FSTLFQGLPA

Majex_14369_c0_g1_i1.p1 903 2.05e-36 DSYKLLETLE VRPKRAAISSAGIQSAMAELYMHMHHLKMPFPSDDEVRIAVLRDCLSAYSSKGMYR SVPFGRRFHG

Mrosa_BAB12269.1 902 2.05e-36 DAYNLLQTLR VQPKRAAISSVGIQSAMAELYMHMRHLQMPFPSDNDVRITVLRNCLSAYSSKGMYR NVPFGRRFFA

Aamph_134975_c0_g1_i1.p1 19 2.05e-36 DAYNLLQTLR VQPKRAAISSVGIQSAMAELYMHMRHLQMPFPSDNDVRITVLRNCLSAYSSKGMYR NVPFGRRFFA

Cmite_22920_c0_g1_i3.p1 484 5.80e-35 VNGRLPTAKP FSYGGVALSGVQFQTVSAVLSSRFGALSARSLQEPILQALIEAGVVSGTGDAAAAA LSRLFTGLPS

Ctest_109122_c0_g1_i4.p1 485 3.32e-34 KNGPLPTYSK FEYDGVVLSATQQRAIFLELQRRFNRLRQPQLQLPLLRVLIRANVVRRNPAAAFRR LFSGLPSFQA

Cmala_14904_c0_g1_i1.p1 480 5.16e-33 IVSGPLPTED FSYSGVLISAAQLRAVSAVLEARFAALKSRRLYAPIIKVLIDADIIGGSGEKAAAS LRRLFDRLPD

Syats_lar_TR59509|c1_g1_ 489 9.11e-20 PTASQVPTNL IVLGNFSLTPEQLSQLESIITSRFSLLNSNEFNIPLIQFFLESNVGSSSSNVAQRV KALVSAINAL

--------------------------------------------------------------------------------

--------------------------------------------------------------------------------

Motif PIYILPGJTLPVEQVRQLVTVJRPRFPFVSLDNVQSIVAHTILJLRAR MEME-6 sites sorted by position p-value

--------------------------------------------------------------------------------

Sequence name Start P-value Site

------------- ----- --------- ------------------------------------------------

Mrosa_BAB12269.1 817 3.16e-47 LVQYRRTPKR LCYIVPGIVLYREQLRQLVTLIRPRFTFVSMRNIRSIVAHTILILRAR YSITQNNCYG

Aamph_12011_c0_g1_i1.p1 223 3.16e-47 LVQYRRTPKR LCYIVPGIVLYREQLRQLVTLIRPRFTFVSMRNIRSIVAHTILILRAR YSITQNNCYG

Wmill_86332_c0_g1_i1.p2 25 4.22e-47 LIQYRRKPER PCYIIPGIILYKEQLRQLVTVILPRFAFVNMYNIRSIVAHTILILRAR YRVTQENCYE

Majex_14369_c0_g1_i1.p1 818 4.22e-47 LIQYRRKPER PCYIIPGIILYKEQLRQLVTVILPRFAFVNMYNIRSIVAHTILILRAR YRVTQENCYE

Ctest_109122_c0_g1_i4.p1 819 3.57e-43 PPVIQAPVPP PIYILSGLTLTVTQVHEVVSVLRVRFPFVSLDNVQGIIAHTVLLLRAK NKVVNQQNAH

Tform_TR54333|c0_g1_i1.p 826 5.05e-42 IQTPKVYVPP PIYILRGLTLTVVQVREIVAVLRVRFTFVSLDNVQAILAHTVLLLRAN GKPVDQKNAY

Aamph_AGS19349.1 823 1.54e-41 VIQYPKYTRA PIYILSGISLPVKQVEQLVVILRTRFVFVSIENVQSILAHTVLLLRAS GQQIVQKNCY

Ctest_86401_c0_g1_i1.p1 78 2.67e-41 YPVIQAPVSP PIYILRGLTLPVRQVHEIVSALRSRFPFVSLDNAQAIIAHTVLLLRSR NSNFNYARAH

Aamph_AKZ20818.1 823 3.69e-38 VEQHKVNIQR DIYILPDIYLPVRSVHQLVVILQKRFVFVSIDNVQTIIVHTILILRAN GVTITSDNCY

Cmala_14904_c0_g1_i1.p1 820 1.38e-36 YQQPKPYVPP PIMLVPGLSLTVKKVEYMVSLLRRRFSFVSLDNVQPILAHIISILRSR GEKITQAYLE

Cmite_22920_c0_g1_i3.p1 822 1.12e-31 HQEPRPPTVP PIPIIDDILLPVADVDRVISLLRGRFPFVSFDNVQPILQHVVLARRAA GVSITAGNLL

--------------------------------------------------------------------------------

--------------------------------------------------------------------------------

Motif IKIFPGISASTVRIVLNILQLTNTGGGKASPKDLLAMITVPKLDASLRSIT MEME-7 sites sorted by position p-value

--------------------------------------------------------------------------------

Sequence name Start P-value Site

------------- ----- --------- ---------------------------------------------------

Tform_TR54333|c0_g1_i1.p 266 5.44e-52 VTIQVVIRET IRLFPGISKTTLRSVLNILQLTNTRGGKATPKDLLAMITIPHLDKSLRTIT DVIANRIFLK

Ctest_109122_c0_g1_i4.p1 266 4.36e-50 VTIQTVIREV IRLFPGVSAGTLRSVLNILQLTNTGGGRATPKDLLAMITIPHLDSSIRSIT DAVANRIYLK

Aamph_AGS19349.1 266 1.47e-47 TNVQLVIKQM LTIFSGISAKTVRIILNILQLTNSAGGKATPKDLLAMITVPKLDVSIRKIT EAAANRVYLK

Majex_14369_c0_g1_i1.p1 266 1.84e-47 IYLQQVIRET IKIFPSISAATVRKVFNSLQLSNTGSGMGSPKDLLTMITVPSLDASLRSIT RMYANNLYKK

Majex_464_c0_g1_i1.p1 1 2.06e-47 . VAHFPGLSSSTARSVLNLLQLTNSAGGKATPKDLLAMITVPKLDASLRSIS EVYAQRALLK

Mrosa_BAB12269.1 267 4.99e-47 MYLQEAIRET INIFPSISPSTVRIVFNNLQLSNTGSGMVSPLDLLAMVTTPVLDDDLKSIT KVYAERLYNK

Aamph_130724_c0_g1_i1.p1 54 4.99e-47 MYLQEAIRET INIFPSISPSTVRIVFNNLQLSNTGSGMVSPLDLLAMVTTPVLDDDLKSIT KVYAERLYNK

Cmala_14904_c0_g1_i1.p1 263 7.76e-44 ITIQQVIAKV VKIFPGTSQRTIRAVLNILQLTNTKGGQASPRDLLAMIAFPAVDKSLRDIS VEIAHRWRLY

Aamph_AKZ20818.1 267 5.89e-40 IVVQEVIREI IKIFPGLSASSVRIILDVLQLTNAPGGKASPRDLLALITVPRLDAELYVIQ EYYIQKYVAS

Cmite_22920_c0_g1_i3.p1 266 4.45e-38 VSIQTVISKV VELFPGLSSASARTILNILQLTNSAGGRARPRNLISMIAIPPIDPSLRVIT DVVVNRFQLH

--------------------------------------------------------------------------------

--------------------------------------------------------------------------------

Motif KHYGALSDRALQAIFRFNLLNNFPDVVPSSRSGVLQVISESLSSLTD MEME-8 sites sorted by position p-value

--------------------------------------------------------------------------------

Sequence name Start P-value Site

------------- ----- --------- -----------------------------------------------

Ctest_109122_c0_g1_i4.p1 56 1.22e-49 LRGYIQQRGV HHYKAFTDRALQAIFRFNLLNNFPDVVPSSRAGVLQVVSESLNELTD KVVPSVSQTG

Aamph_AGS19349.1 56 1.02e-46 LRGYVKERGV KHYDVLSNDALQAIFRFNLINNFPDVVPATRTGVLQIISESLNTLTD AVVPSVPQCG

Tform_TR54333|c0_g1_i1.p 56 2.31e-46 LRGYIKQRGV THYEAFTDYSLQAIYRFNLLNNFPDVVPSSRSGVIQVVSESLKELTD EVVPSAPTCG

Mrosa_BAB12269.1 57 3.08e-45 LREYVKKQGV MHYESLSDISLKAIFRNKLLNNFPEEVPATRDGVLQVITESLGSLTD SVVPSVSQCG

Majex_524_c0_g1_i1.p1 23 2.46e-44 LRGYLQQRGV KHYGALSDLALKAMFHFNLINNFPNVVPATRSGMIQVVSGSLSSLTD EVLPSVSQCG

Majex_14369_c0_g1_i1.p1 56 3.13e-44 LRGYVKERGV KHYESLSDISLKAIFRNKLINNFPDEVPATRDGLLQVIAESFGSLTD SVVPSVAQCD

Mlong_37885_c0_g1_i1.p2 40 6.23e-43 LRGLVRQQGV THYRTLSDRALQAVFRFSLLNNFPAVVPSSRSGVLQLVAESLSSLTD AVVPSQNQVS

Cmite_22920_c0_g1_i3.p1 56 1.38e-38 VRKYIGGLGV KHVGSLTDQALEAVFRFNLLNNFPGVVPSNRAGVIEVILRGLRELSD SVVPSQKQVD

Cmala_14904_c0_g1_i1.p1 53 1.16e-37 LRGYVYNRGV KHADVLTDACLQGVFKFSILNNFPDVVPVRRAGMIQIIEQGLSELTD KVVPGADVVA

Aamph_AKZ20818.1 57 7.51e-35 LRVYIQQLGI KNHPVLTDDVINAIFRFNLNNNYQGQVPSKRSTLLQIFSESFSSLTD AVLPSVSQCG

Syats_lar_TR59509|c1_g1_ 63 9.65e-34 IGRGFHALQG SQVGLLSDRVLQLIFRFMLLNDFPGGVARSRSEILHIFHGALGKLSP SQFPDQSSFK

Syats_ext_TR19508|c0_g1_ 63 9.65e-34 IGRGFHALQG SQVGLLSDRVLQLIFRFMLLNDFPGGVARSRSEILHIFHGALGKLSP SQFPDQSSFK

--------------------------------------------------------------------------------

--------------------------------------------------------------------------------

Motif AAGGDIMFPRNGCGCLRNPVAADLTNEEISHLRGYIKQRGV MEME-9 sites sorted by position p-value

--------------------------------------------------------------------------------

Sequence name Start P-value Site

------------- ----- --------- -----------------------------------------

Tform_TR54333|c0_g1_i1.p 15 8.19e-43 PLALTLLLAA FASGSIMFPRNGCGCLRNPVAAELKTEEITQLRGYIKQRGV THYEAFTDYS

Ctest_109122_c0_g1_i4.p1 15 2.06e-41 PLALALLLVA SANGSFMFPRNGCGCIRNPVAAQLKPEEIAHLRGYIQQRGV HHYKAFTDRA

Aamph_AGS19349.1 15 2.80e-41 PLALALLLAA SAYGNVLFARSGCGCLRNPVAAKLTGEEISHLRGYVKERGV KHYDVLSNDA

Majex_14369_c0_g1_i1.p1 15 7.93e-38 LVAVLLLVAS ANGYRPSFARRGCGCLRSPVAADLDDKEIAILRGYVKERGV KHYESLSDIS

Cmala_14904_c0_g1_i1.p1 12 1.70e-37 KLALLLALLG AASGTIMFPRNGCGCGRNINARPLTPPEIKHLRGYVYNRGV KHADVLTDAC

Cmite_22920_c0_g1_i3.p1 15 1.56e-36 ALLTLLLAAA AAGGDLHFNRNGCGCLRNPVAGPLTAAELAQVRKYIGGLGV KHVGSLTDQA

Mrosa_BAB12269.1 16 4.18e-34 LVAVLLVTVS VTGHRPSFERRCCGCLRSPVAADLDDDEIGMLREYVKKQGV MHYESLSDIS

Syats_lar_TR59509|c1_g1_ 17 8.95e-33 IATVVVTLLA ANSADAGFARSGCGCLRNRLAGKLSNDELSSIRQLLIGRGF HALQGSQVGL

Syats_ext_TR19508|c0_g1_ 17 8.95e-33 IATVVVTLLA ANSADAGFARSGCGCLRNRLAGKLSNDELSSIRQLLIGRGF HALQGSQVGL

Aamph_AKZ20818.1 16 2.68e-30 LALAVLLAVS AAGDKYPISRFGCGCNRNIIAADLTVQEISQLRVYIQQLGI KNHPVLTDDV

--------------------------------------------------------------------------------

--------------------------------------------------------------------------------

Motif PKEPQJVZYQQLIESVVSKCSVNSFILNEKQLTSIQSDLYKSKGIKIELSLLIDINYMAYFAVCQSGAYRPVMNRYLYRS MEME-10 sites sorted by position p-value

--------------------------------------------------------------------------------

Sequence name Start P-value Site

------------- ----- --------- --------------------------------------------------------------------------------

Majex_14369_c0_g1_i1.p1 685 4.76e-93 IKYINEYEIL PKEAQIAEYQQLIKSVMTKCSVNSLILNEKQLNSILSNLYKSKGIKIELSMLIDINYMAYFAVCQSGSYRPAMHNYLYRS IISYNLTVRK

Wmill_41843_c0_g1_i1.p1 81 1.38e-91 IKYINEYEIL PKEAQIAEYQQLIKSVMTKCSVNSLILNEKQLNSILSNLYKSKGIKIELSMLIDINYMAYFAVCQSGSYRPAMHNYLYRR S

Mrosa_BAB12269.1 690 1.70e-87 NEYKLLPKAA QNVPLLVQYQQLMESMVSKCSVSSFILSKKQLTTIQSDLYKSRRIRIELSLLVDINYMAYFAVCQSGAYTAVMNRYVYQS IISYTQTVRK

Aamph_12011_c0_g1_i1.p1 96 1.70e-87 NEYKLLPKAA QNVPLLVQYQQLMESMVSKCSVSSFILSKKQLTTIQSDLYKSRRIRIELSLLVDINYMAYFAVCQSGAYTAVMNRYVYQS IISYTQTVRK

--------------------------------------------------------------------------------
