# Supplementary material for: Histology and transcriptomic analyses of barnacles with different base materials and habitats shed lights on the duplication and chemical diversification of barnacle cement proteins
Source: BMC Genomics. 2021 Nov 1;22:783. doi: 10.1186/s12864-021-08049-4 (PMC8561864; doi:10.1186/s12864-021-08049-4)
Supplement: Supplementary file 5 — Additional file 5 [file 12864_2021_8049_MOESM5_ESM.docx]

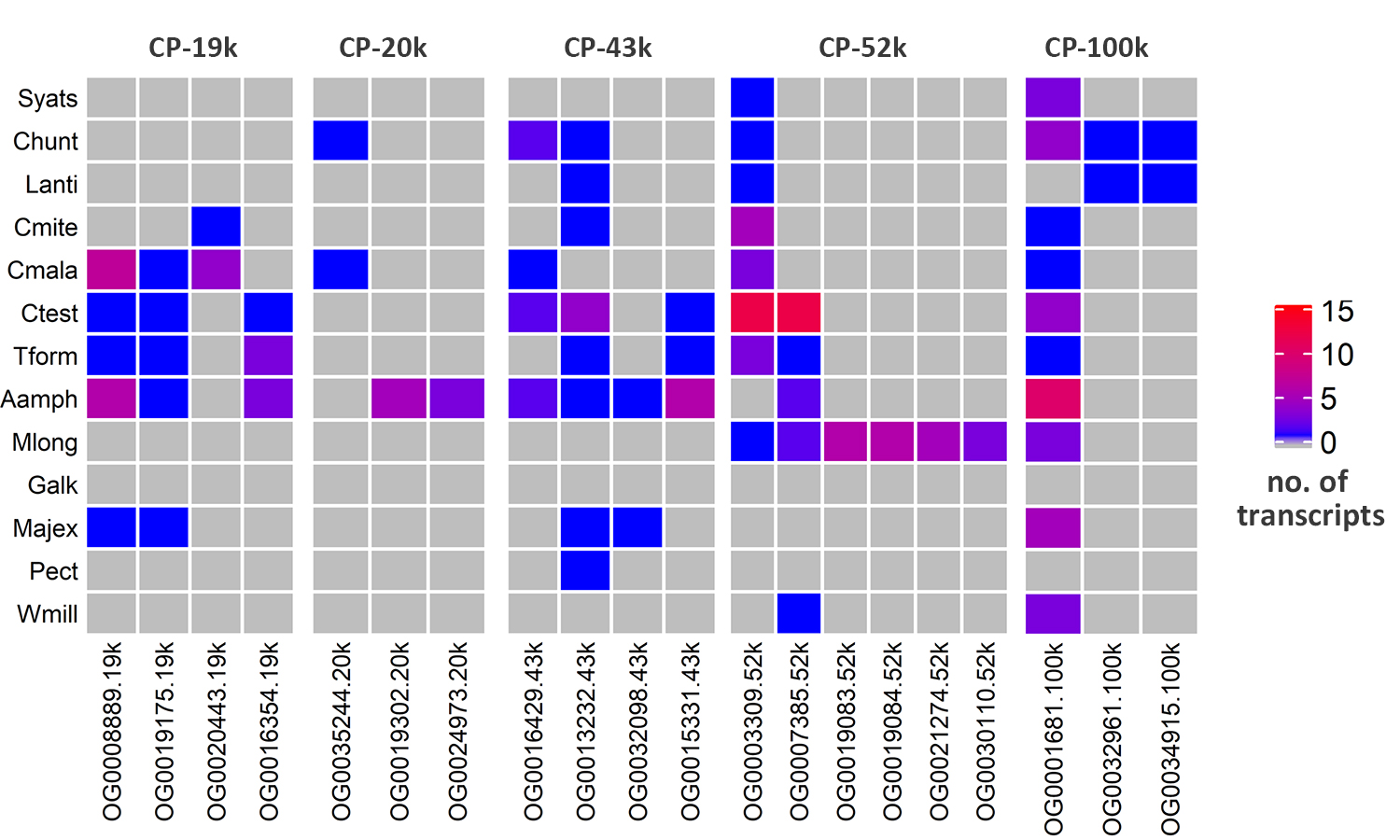


**Additional file 5. Summary of orthogroup assignments for CP homologs.** Heatmap summarizing the number of CP homologs assigned to different orthogroups.
