# Supplementary material for: Histology and transcriptomic analyses of barnacles with different base materials and habitats shed lights on the duplication and chemical diversification of barnacle cement proteins
Source: BMC Genomics. 2021 Nov 1;22:783. doi: 10.1186/s12864-021-08049-4 (PMC8561864; doi:10.1186/s12864-021-08049-4)
Supplement: Supplementary file 6 — Additional file 6 [file 12864_2021_8049_MOESM6_ESM.docx]

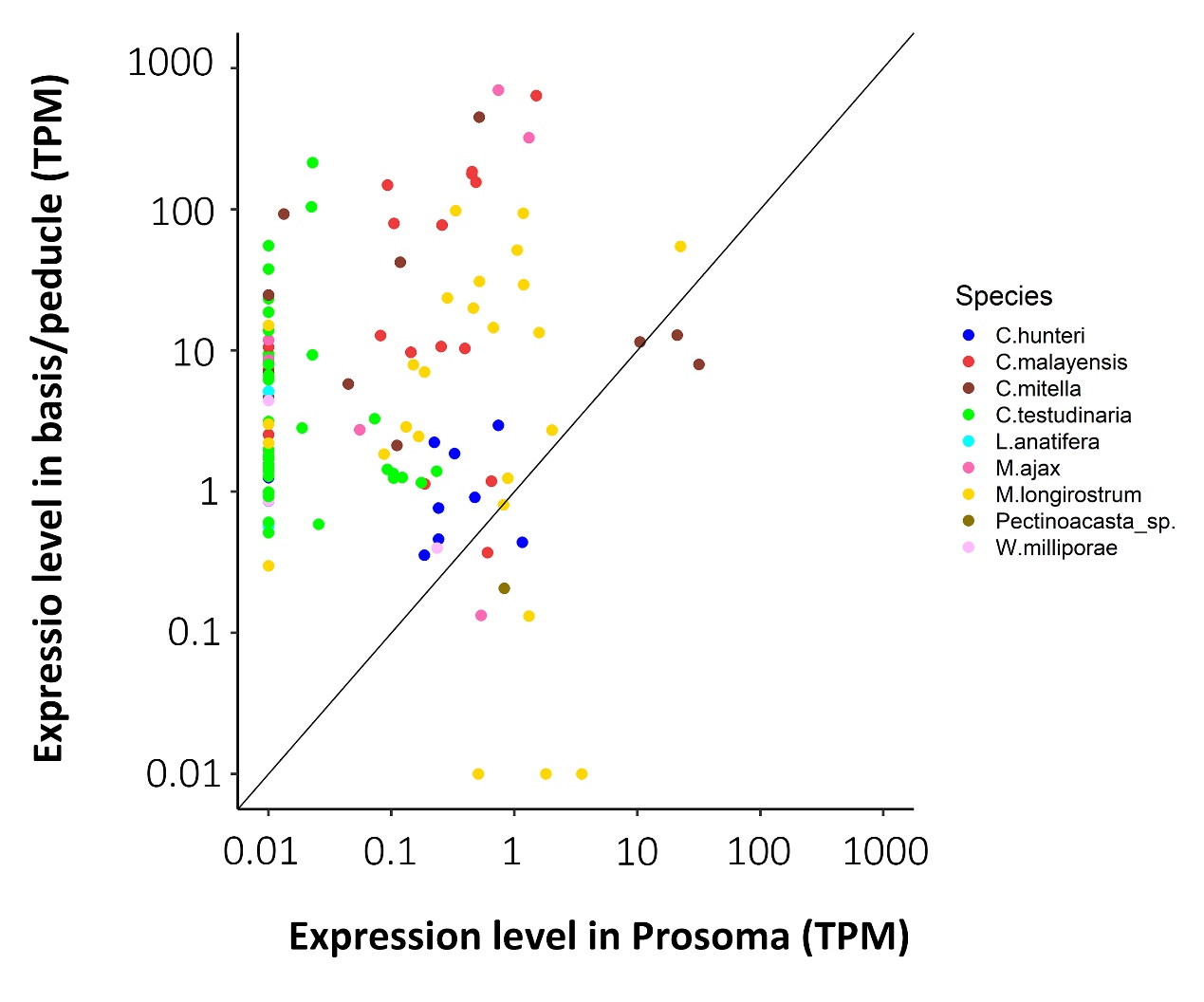


**Additional file 6.** **mRNA expression level of CP homologs in the prosoma against the base or the peduncle.** mRNA expression level is presented as log10-transformed transcripts per million read (TPM) value.
