# Supplementary material for: Histology and transcriptomic analyses of barnacles with different base materials and habitats shed lights on the duplication and chemical diversification of barnacle cement proteins
Source: BMC Genomics. 2021 Nov 1;22:783. doi: 10.1186/s12864-021-08049-4 (PMC8561864; doi:10.1186/s12864-021-08049-4)
Supplement: Supplementary file 7 — Additional file 7 [file 12864_2021_8049_MOESM7_ESM.docx]

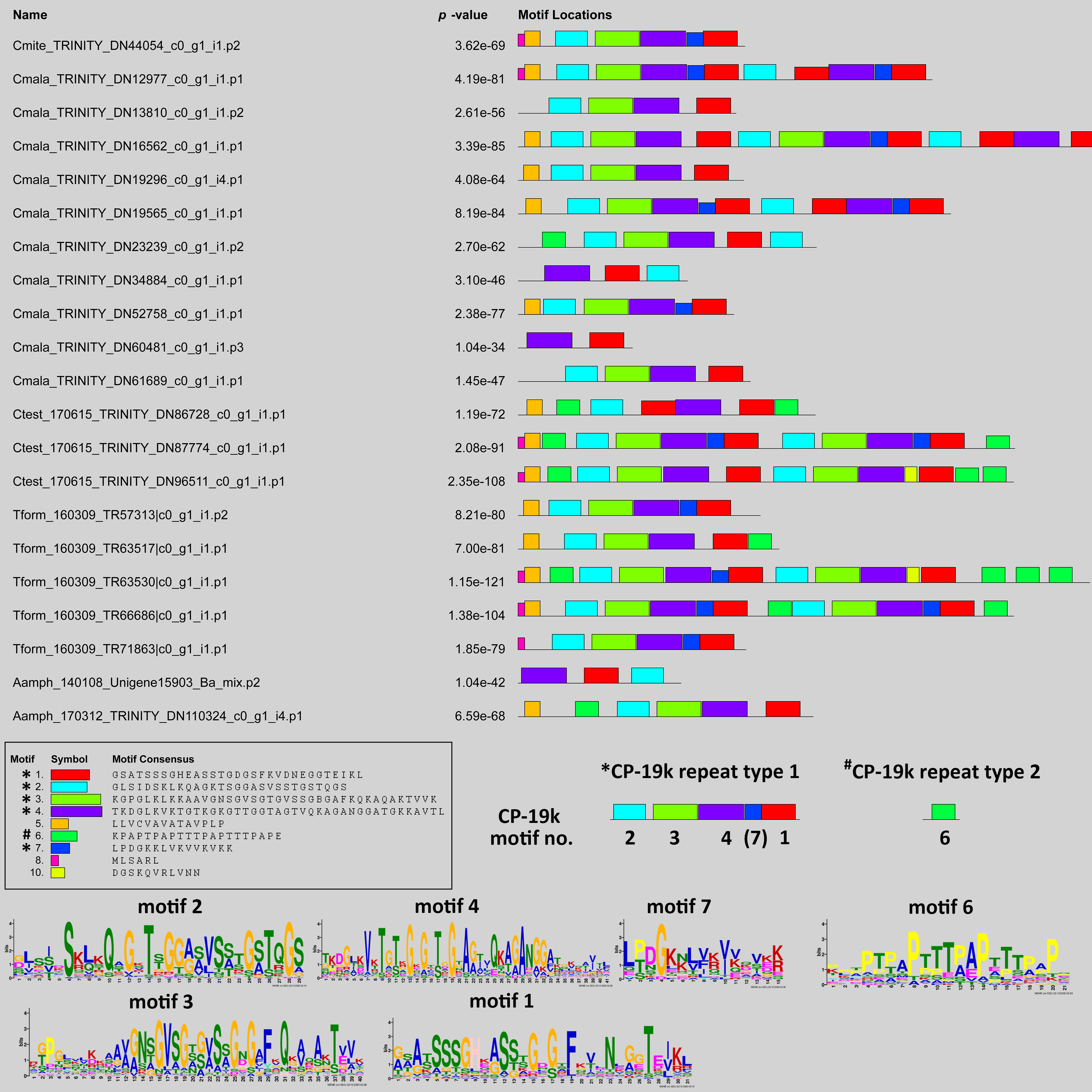


**Figure S1. MEME motif structure of all CP19k homologs.**


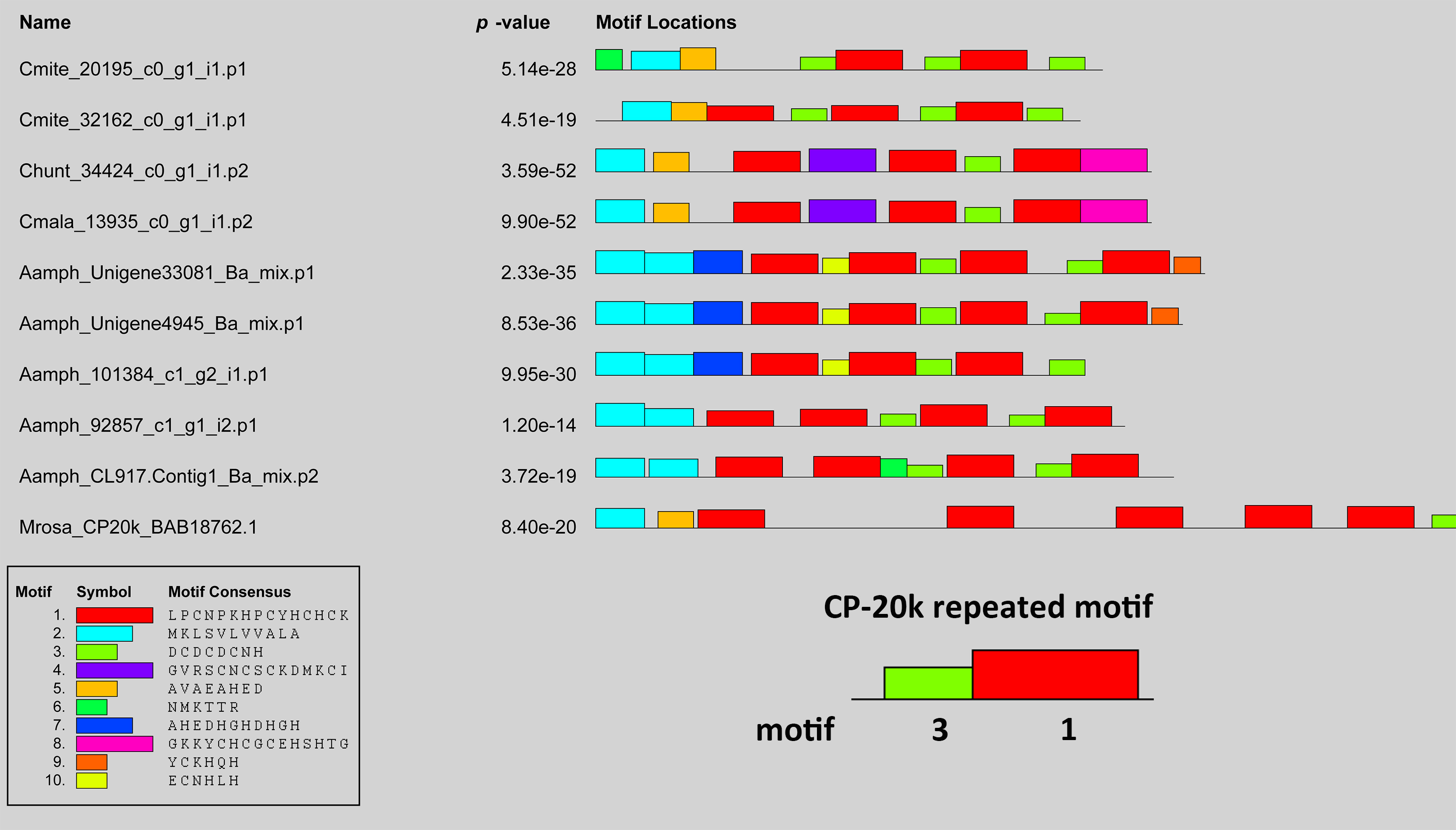


**Figure S2. MEME motif structure of all CP20k homologs.**


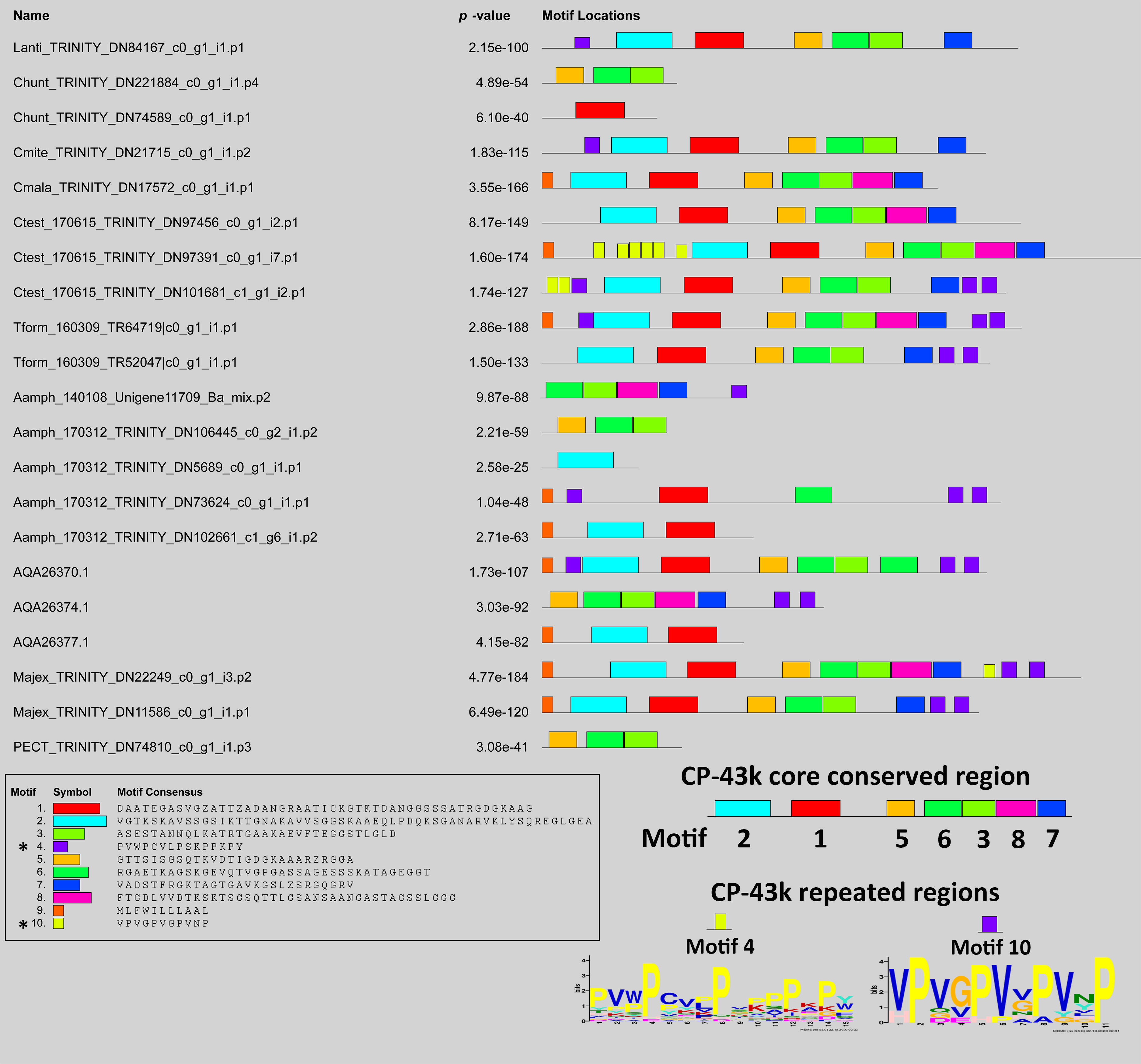


**Figure S3. MEME motif structure of all CP43k homologs.**


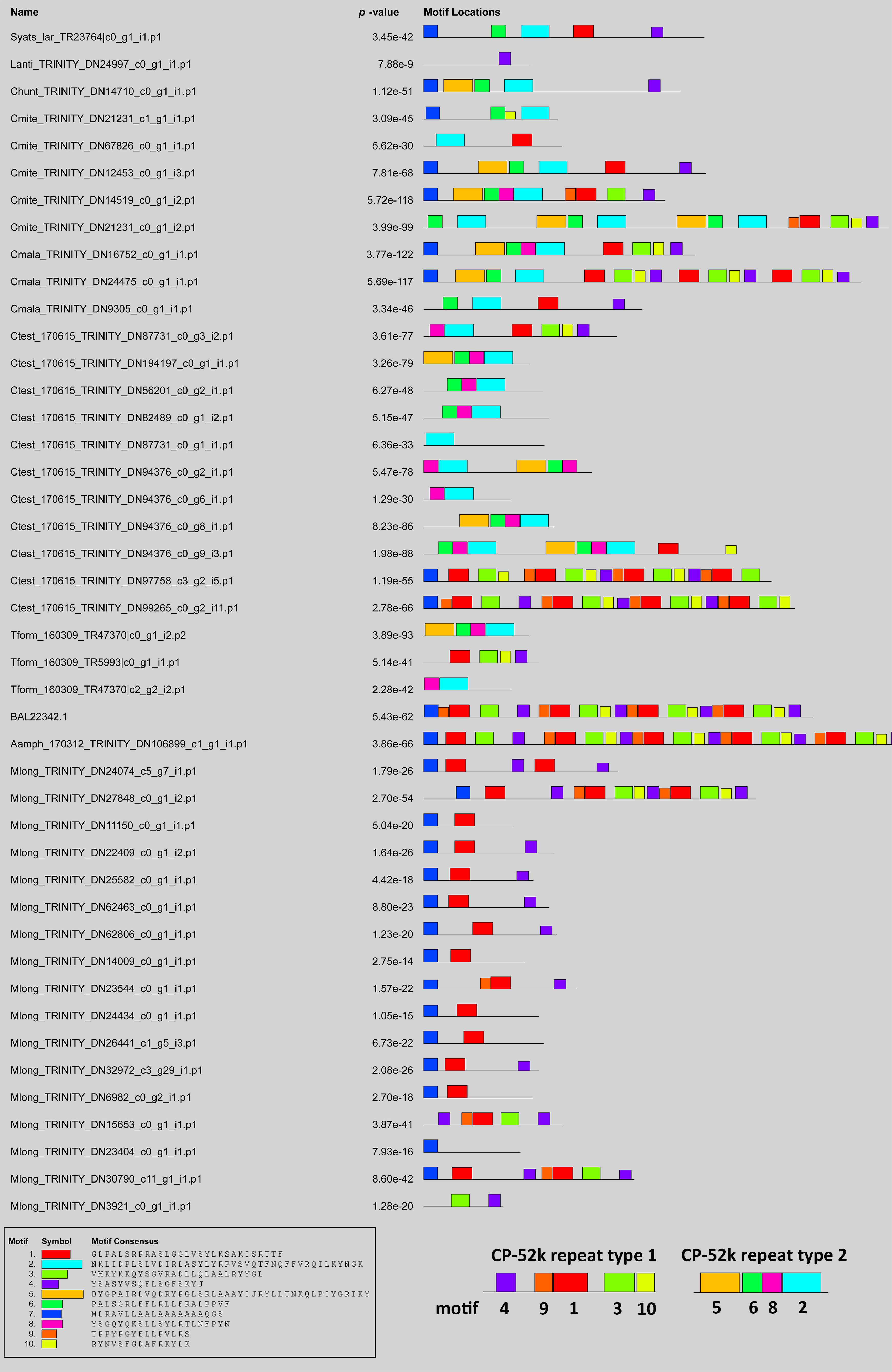


**Figure S4. MEME motif structure of all CP52k homologs.**


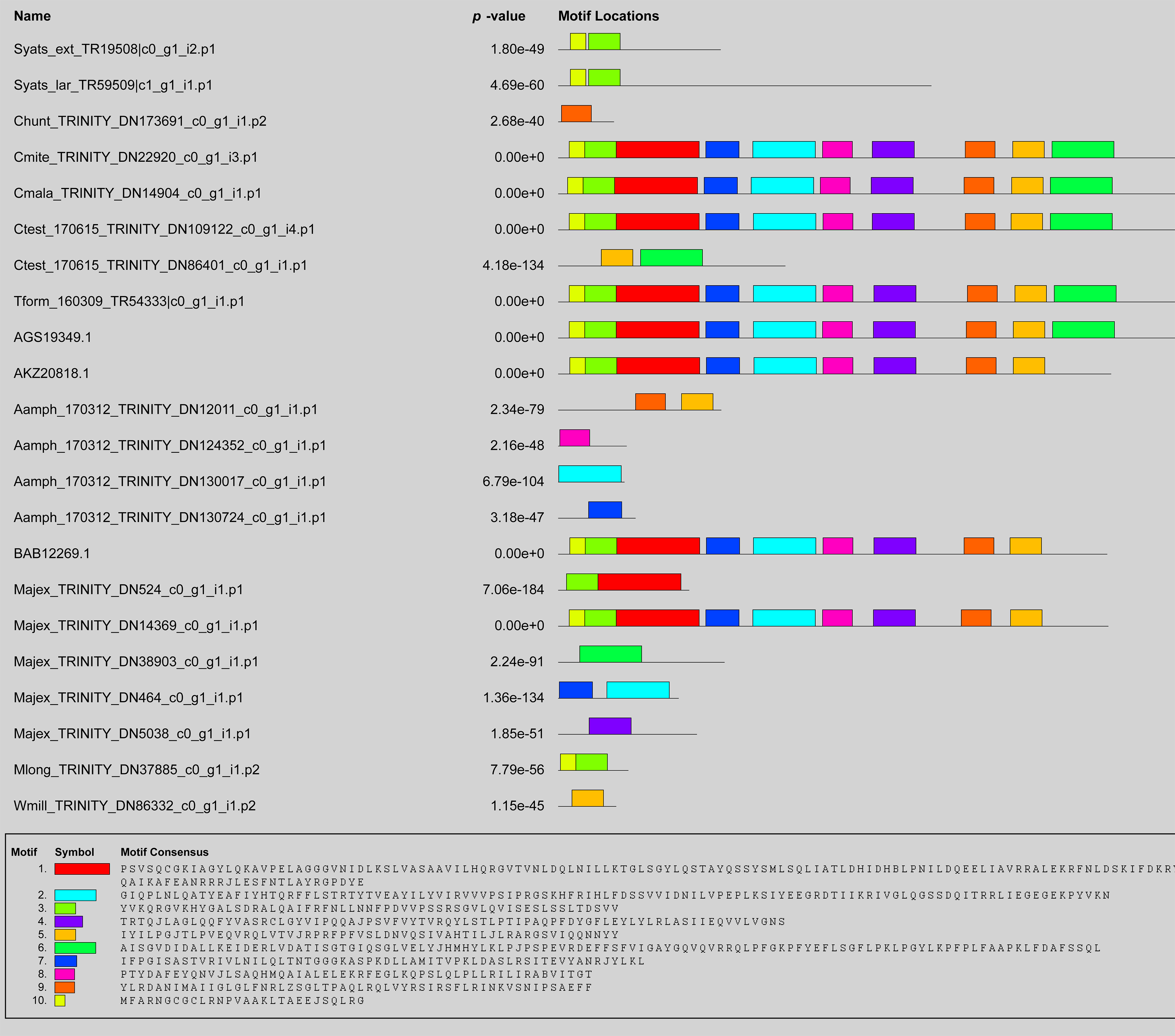


**Figure S5. MEME motif structure of all CP100k homologs.**


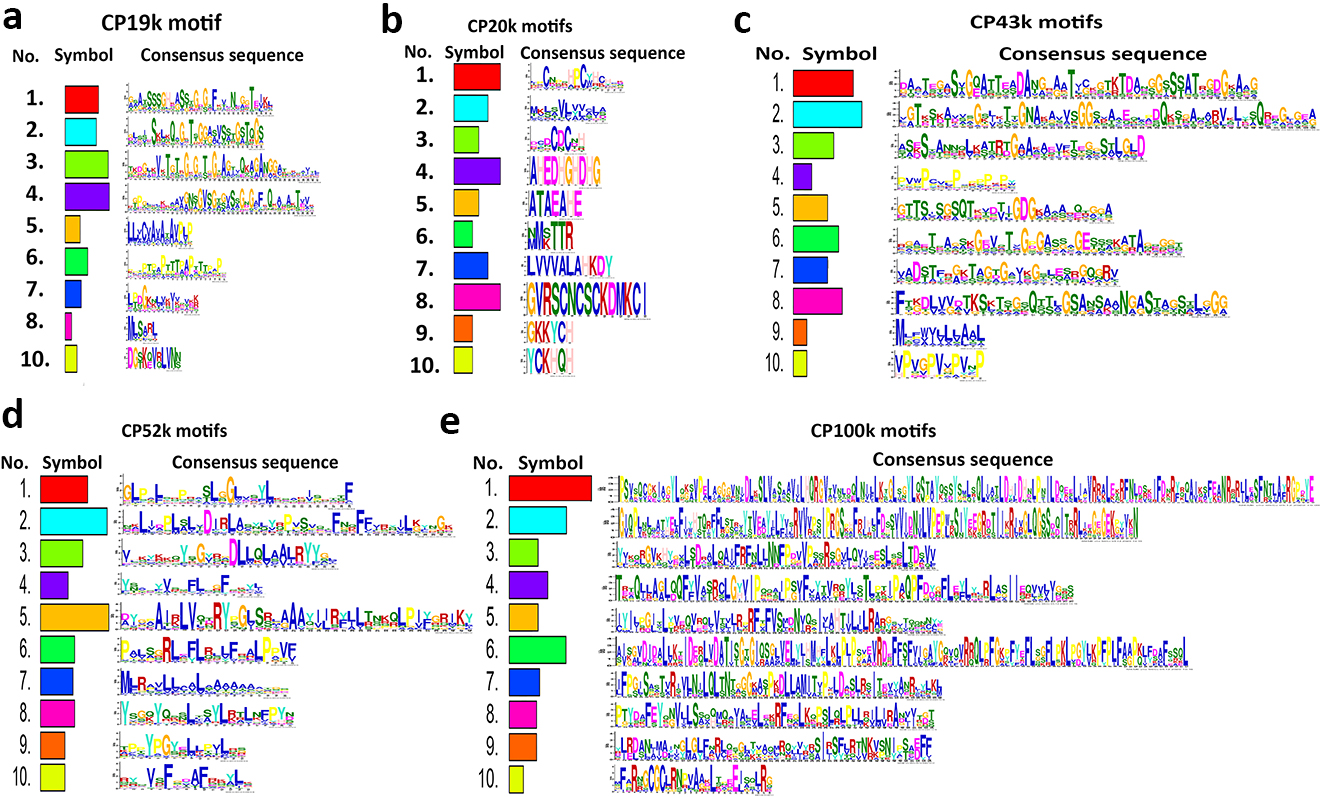


**Figure S6. MEME motif consensus sequences of all CPs.** Motif consensus sequences of **a.** CP19k homologs, **b.** CP20k homologs, **c.** CP43k homologs, **d.** CP52k homologs, and **e.** CP100k homologs.
